# Weekly dose-dense chemotherapy in first-line epithelial ovarian, fallopian tube, or primary peritoneal cancer treatment (ICON8): overall survival results from an open-label, randomised, controlled, phase 3 trial

**DOI:** 10.1016/S1470-2045(22)00283-2

**Published:** 2022-07

**Authors:** Andrew R Clamp, Elizabeth C James, Iain A McNeish, Andrew Dean, Jae-Won Kim, Dearbhaile M O'Donnell, Dolores Gallardo-Rincon, Sarah Blagden, James Brenton, Tim J Perren, Sudha Sundar, Rosemary Lord, Graham Dark, Marcia Hall, Susana Banerjee, Rosalind M Glasspool, C Louise Hanna, Sarah Williams, Kate M Scatchard, Helena Nam, Sharadah Essapen, Christine Parkinson, Lucy McAvan, Ann Marie Swart, Babasola Popoola, Francesca Schiavone, Jonathan Badrock, Fuad Fananapazir, Adrian D Cook, Mahesh Parmar, Richard Kaplan, Jonathan A Ledermann

**Affiliations:** a The Christie NHS Foundation Trust and University of Manchester, Manchester, UK; b MRC Clinical Trials Unit at UCL, Institute of Clinical Trials and Methodology, University College London, London, UK; c Department of Surgery and Cancer, Imperial College London, London, UK; d Oncology, St John of God Hospital, Subiaco, WA, Australia; e Department of Obstetrics and Gynecology, Seoul National University, Seoul, South Korea; f Cancer Trials Ireland, Dublin, Ireland; g Department of Medical Oncology, Instituto Nacional de Cancerologia, Mexico City, Mexico; h Department of Oncology, Churchill Hospital, University of Oxford, Oxford, UK; i Li Ka Shing Centre, Cancer Research UK Cambridge Institute, Cambridge, UK; j Leeds Institute of Medical Research, St James' University Hospital, Leeds, UK; k Institute of Cancer and Genomics, University of Birmingham, Birmingham, UK; l Department of Oncology, Clatterbridge Cancer Centre, Wirral, UK; m Northern Centre for Cancer Care, Freeman Hospital, Newcastle, UK; n Department of Medical Oncology, Mount Vernon Cancer Centre, Northwood, UK; o Gynaecological Unit, The Royal Marsden NHS Foundation Trust and Institute of Cancer Research, London, UK; p Beatson West of Scotland Cancer Centre, Glasgow, UK; q Department of Oncology, Velindre Cancer Centre, Cardiff, UK; r Birmingham City Hospital, Birmingham, UK; s North Devon District Hospital, Barnstaple, UK; t Exeter Oncology Centre, Royal Devon and Exeter Hospital, Exeter, UK; u Broomfield Hospital, Chelmsford, UK; v Southend University Hospital, Southend, UK; w St Luke's Cancer Centre, Royal Surrey County Hospital, Guildford, UK; x Cambridge Cancer Centre, Addenbrooke's Hospital, Cambridge, UK; y Department of Oncology, University Hospital Coventry and Warwickshire, Coventry, UK; z Norwich Clinical Trials Unit, Norwich Medical School, University of East Anglia, Norwich, UK; aa Cancer Research UK and UCL Cancer Trials Centre, UCL Cancer Institute and UCL Hospitals, London, UK

## Abstract

**Background:**

Standard-of-care first-line chemotherapy for epithelial ovarian cancer is carboplatin and paclitaxel administered once every 3 weeks. The JGOG 3016 trial reported significant improvement in progression-free and overall survival with dose-dense weekly paclitaxel and 3-weekly (ie, once every 3 weeks) carboplatin. However, this benefit was not observed in the previously reported progression-free survival results of ICON8. Here, we present the final coprimary outcomes of overall survival and updated progression-free survival analyses of ICON8.

**Methods:**

In this open-label, randomised, controlled, phase 3 trial (ICON8), women aged 18 years or older with newly diagnosed stage IC–IV epithelial ovarian, primary peritoneal, or fallopian tube carcinoma (here collectively termed ovarian cancer, as defined by International Federation of Gynecology and Obstetrics [FIGO] 1988 criteria) and an Eastern Cooperative Oncology Group performance status of 0–2 were recruited from 117 hospitals with oncology departments in the UK, Australia and New Zealand, Mexico, South Korea, and Ireland. Patients could enter the trial after immediate primary surgery (IPS) or with planned delayed primary surgery (DPS) during chemotherapy, or could have no planned surgery. Participants were randomly assigned (1:1:1), using the Medical Research Council Clinical Trials Unit at University College London randomisation line with stratification by Gynecologic Cancer Intergroup group, FIGO disease stage, and outcome and timing of surgery, to either 3-weekly carboplatin area under the curve (AUC)5 or AUC6 and 3-weekly paclitaxel 175 mg/m^2^ (control; group 1), 3-weekly carboplatin AUC5 or AUC6 and weekly paclitaxel 80 mg/m^2^ (group 2), or weekly carboplatin AUC2 and weekly paclitaxel 80 mg/m^2^ (group 3), all administered via intravenous infusion for a total of six 21-day cycles. Coprimary outcomes were progression-free survival and overall survival, with comparisons done between group 2 and group 1, and group 3 and group 1, in the intention-to-treat population. Safety was assessed in all patients who started at least one chemotherapy cycle. The trial is registered on ClinicalTrials.gov, NCT01654146, and ISRCTN registry, ISRCTN10356387, and is closed to accrual.

**Findings:**

Between June 6, 2011, and Nov 28, 2014, 1566 patients were randomly assigned to group 1 (n=522), group 2 (n=523), or group 3 (n=521). The median age was 62 years (IQR 54–68), 1073 (69%) of 1566 patients had high-grade serous carcinoma, 1119 (71%) had stage IIIC–IV disease, and 745 (48%) had IPS. As of data cutoff (March 31, 2020), with a median follow-up of 69 months (IQR 61–75), no significant difference in overall survival was observed in either comparison: median overall survival of 47·4 months (95% CI 43·1–54·8) in group 1, 54·8 months (46·6–61·6) in group 2, and 53·4 months (49·2–59·6) in group 3 (group 2 *vs* group 1: hazard ratio 0·87 [97·5% CI 0·73–1·05]; group 3 *vs* group 1: 0·91 [0·76–1·09]). No significant difference was observed for progression-free survival in either comparison and evidence of non-proportional hazards was seen (p=0·037), with restricted mean survival time of 23·9 months (97·5% CI 22·1–25·6) in group 1, 25·3 months (23·6–27·1) in group 2, and 24·8 months (23·0–26·5) in group 3. The most common grade 3–4 adverse events were reduced neutrophil count (78 [15%] of 511 patients in group 1, 183 [36%] of 514 in group 2, and 154 [30%] of 513 in group 3), reduced white blood cell count (22 [4%] in group 1, 80 [16%] in group 2, and 71 [14%] in group 3), and anaemia (26 [5%] in group 1, 66 [13%] in group 2, and 24 [5%] in group 3). No new serious adverse events were reported. Seven treatment-related deaths were reported (two in group 1, four in group 2, and one in group 3).

**Interpretation:**

In our cohort of predominantly European women with epithelial ovarian cancer, we found that first-line weekly dose-dense chemotherapy did not improve overall or progression-free survival compared with standard 3-weekly chemotherapy and should not be used as part of standard multimodality front-line therapy in this patient group.

**Funding:**

Cancer Research UK, Medical Research Council, Health Research Board in Ireland, Irish Cancer Society, and Cancer Australia.

## Introduction

Epithelial ovarian cancer is the leading cause of death related to gynaecological cancer in high-income countries, and accounts for more than 180 000 deaths annually worldwide.^1^ Most women with epithelial ovarian cancer present with advanced International Federation of Gynecology and Obstetrics (FIGO) stage III–IV disease and, despite primary multimodality treatment with cytoreductive surgery and chemotherapy, many have disease relapse and a median survival of less than 4 years.^1^ Platinum–paclitaxel doublet chemotherapy administered once every 3 weeks for six to eight cycles has been the reference standard of care, first-line systemic treatment for ovarian cancer for the past 27 years.^2^, ^3^, ^4^ Although chemotherapy is commonly administered after primary surgery, delayed surgery after three or four primary neoadjuvant chemotherapy cycles has been widely adopted in patients for whom complete primary cytoreduction is deemed unlikely. This approach is supported by three randomised trials that each found that overall survival with neoadjuvant chemotherapy and delayed primary surgery is not inferior to upfront surgery and is associated with reduced perioperative morbidity.^5^, ^6^, ^7^, ^8^

There has been substantial interest in the assessment of weekly dose-dense paclitaxel schedules. Preclinical studies suggest that administration of metronomic taxane improves drug delivery, increases tumour cell apoptosis, and reduces angiogenesis.^9^, ^10^ Weekly paclitaxel treatment is an efficacious and well tolerated approach in recurrent platinum-resistant epithelial ovarian cancer and confers a survival advantage compared with 3-weekly (ie, once every 3 weeks) scheduling in both the adjuvant and metastatic setting for breast cancer.^11^, ^12^

The JGOG 3016 trial randomly assigned 637 Japanese women with newly diagnosed epithelial ovarian cancer to receive standard of care conventional 3-weekly carboplatin area under the curve [AUC]6 with paclitaxel 180 mg/m^2^ chemotherapy or an investigational group in which weekly dose-dense paclitaxel at a dose of 80 mg/m^2^ was administered with 3-weekly carboplatin AUC6.^13^, ^14^ In this trial, weekly treatment resulted in an 11-month extension in median progression-free survival and a corresponding 38-month improvement in median overall survival (hazard ratio [HR] 0·76 [95% CI 0·63–0·99]; p=0·039). However, weekly dose-dense treatment caused an increase in haematological toxicity, which compromised physicians' ability to deliver at least six cycles of treatment. By contrast, the assessment of weekly scheduling of both carboplatin and paclitaxel in phase 2 trials showed both excellent tolerability and promising efficacy.^15^, ^16^

**Research in context**

**Evidence before this study**

For more than 20 years, the cornerstone of systemic treatment for first-line epithelial ovarian cancer has been carboplatin and paclitaxel administered once every 3 weeks. We searched MEDLINE to identify any relevant phase 3 trials assessing weekly dose-dense paclitaxel scheduling published in English between Jan 1, 1990, and Dec 21, 2020, using the terms “ovarian neoplasm” AND (“chemotherapy” OR “drug therapy”) AND “paclitaxel” AND “progression-free survival” AND “clinical trial”. In addition to the previously reported progression-free survival results of ICON8, three relevant trials were identified. The preliminary results of ICON8 showed no significant difference in progression-free survival between standard 3-weekly (ie, once every 3 weeks) chemotherapy, 3-weekly carboplatin and dose-dense weekly paclitaxel, and weekly carboplatin and dose-dense weekly paclitaxel. The JGOG-3016 study of 3-weekly carboplatin and paclitaxel versus 3-weekly carboplatin with weekly dose-dense paclitaxel found a significant improvement in both progression-free and overall survival in Japanese women; however, because of increased toxicity in the weekly paclitaxel group, treatment delivery rates were lower than expected. The MITO-7 study of 3-weekly carboplatin and paclitaxel versus weekly carboplatin and paclitaxel (not dose dense) found no benefit in progression-free survival, but did find that weekly chemotherapy was associated with improved quality of life. GOG-0262 assessed 3-weekly carboplatin and paclitaxel versus weekly paclitaxel with 3-weekly carboplatin (identical to JGOG 3016); however, 84% of patients in the GOG-0262 trial opted to additionally receive bevacizumab alongside chemotherapy. No benefit in progression-free survival was observed in the whole trial population; however, a benefit was observed in the small underpowered chemotherapy only subgroup. Neither MITO7 nor GOG-0262 were adequately powered or had sufficient follow-up to assess the effect of weekly chemotherapy scheduling on overall survival.

**Added value of this study**

To our knowledge, ICON8 is the largest study to assess weekly chemotherapy in newly diagnosed epithelial ovarian cancer and was designed with appropriately powered coprimary endpoints of progression-free and overall survival. In this study, we provide mature overall survival and long-term progression-free survival results that show no significant difference for weekly dose-dense chemotherapy compared with standard 3-weekly chemotherapy.

**Implications of all the available evidence**

In this cohort of predominantly European women, we found that weekly dose-dense paclitaxel should not be used as part of standard multimodality epithelial ovarian cancer treatment in the front-line setting. There might be potential racial and ethnic pharmacogenomics differences in efficacy of dose-dense paclitaxel in Japanese populations compared with the predominantly European ICON8 population.

The Gynecologic Cancer InterGroup (GCIG) International Collaboration on Ovarian Neoplasms 8 (ICON8) trial was designed to assess whether the incorporation of dose-dense weekly paclitaxel into first-line treatment of epithelial ovarian cancer in a predominantly European patient group would improve survival outcomes, and also to determine whether weekly scheduling of carboplatin would reduce haematological adverse events, improve deliverability, and maintain the efficacy of dose-dense paclitaxel compared with weekly paclitaxel 80 mg/m^2^ with 3-weekly carboplatin AUC6.

In 2019, we reported the ICON8 primary progression-free survival analysis, which showed that although weekly treatment regimens were tolerable and could safely deliver increased paclitaxel dose density, there was no significant difference in progression-free survival restricted mean survival time between standard 3-weekly carboplatin–paclitaxel and either of two experimental weekly dose-dense regimens.^17^ Subsequently, in 2020, we published the outcome of the quality-of-life substudy to ICON8 that confirmed the tolerability of the weekly schedules overall, but highlighted worse neurological toxic effects and fatigue during treatment for patients receiving weekly treatment.^18^ In this Article, we report the mature analysis of overall survival, one of ICON8's coprimary endpoints, and an updated progression-free survival analysis.

## Methods

### Study design and participants

ICON8 was an international, open-label, randomised, phase 3 trial of weekly dose-dense chemotherapy as first-line treatment in patients with histologically confirmed invasive epithelial ovarian, primary peritoneal, or fallopian tube carcinoma (here collectively termed ovarian cancer). Patients were recruited at hospitals with oncology departments in the UK (88 sites), Australia and New Zealand (19 sites), Mexico (two sites), South Korea (three sites), and Ireland (five sites); all 117 sites recruited at least one patient (appendix pp 6–9) Full details of methods for ICON8 have been reported elsewhere.^17^ Patients were eligible if they were aged 18 years or older and had newly diagnosed FIGO (1988) stage IC–IV cancer (with mandatory high-risk histological subtype for patients with FIGO [1988] stage IC or IIA disease), an Eastern Cooperative Oncology Group (ECOG) performance status of 0–2, life expectancy of more than 12 weeks, and adequate haematological (absolute neutrophil count ≥1·5 × 10^9^ per L, platelet count of ≥100 × 10^9^ per L, and haemoglobin concentration of ≥9 g/dL), renal (radioisotopic glomerular filtration rate of ≥30 mL/min, or calculated creatinine clearance of ≥60 mL/min), and hepatic function (bilirubin concentration of ≤1·5 × the upper limit of normal [ULN] and serum transaminase concentration of ≤3 × ULN in the absence of parenchymal liver metastases or ≤5 × ULN in the presence of parenchymal liver metastases).^19^ A 10% cap to recruitment was placed on the number of patients with early stage high-risk disease. Exclusion criteria included receipt of previous systemic therapy for ovarian cancer and plans to receive maintenance treatment after completion of protocol therapy. Full exclusion criteria are in the protocol (appendix). Patients gave written, informed consent.

Patients could enter the trial after upfront or immediate primary cytoreductive surgery (IPS) or could receive primary or neoadjuvant chemotherapy with a plan for delayed primary cytoreductive surgery (DPS) or no surgery at the decision of the local multidisciplinary gynaecological oncology team. Chemotherapy was required to start within 8 weeks of IPS.

Ethical approval was granted in the UK by the London-Chelsea research ethics committee. Ethical approval was also granted by the appropriate national or local institutional review boards in other jurisdictions. All protocol amendments were approved by relevant ethics committees and regulatory bodies and are listed in the protocol (appendix).

### Randomisation and masking

Participants were randomly assigned (1:1:1) to 3-weekly carboplatin and paclitaxel (control; group 1), 3-weekly carboplatin and weekly paclitaxel (group 2), or weekly carboplatin and paclitaxel (group 3). Patients were randomly assigned using the Medical Research Council Clinical Trials Unit (MRC CTU) at University College London (UCL) randomisation line. Randomisation was done using minimisation stratified by GCIG group, disease stage (FIGO stage IC–IIA high grade serous, clear cell, or grade III carcinoma *vs* FIGO stage IIB–IV), and outcome and timing of surgery (IPS plus FIGO stage IC–III with no visible residual disease *vs* IPS plus FIGO stage IC–III with residual disease of ≤1 cm in diameter *vs* IPS plus FIGO stage IV or FIGO stage IC–III with residual disease of >1 cm in diameter *vs* no surgery planned *vs* DPS planned). Due to the nature of the interventions, neither patients nor clinicians were masked to treatment allocation.

### Procedures

Patients in the control group (group 1) received carboplatin AUC5 or AUC6 by intravenous infusion over 30–60 min and paclitaxel 175 mg/m^2^ by intravenous infusion over 3 h on day 1 of each 21-day cycle for six cycles. Patients in the weekly paclitaxel group (group 2) received carboplatin as in group 1 and dose-fractionated paclitaxel 80 mg/m^2^ by intravenous infusion over 1 h on days 1, 8, and 15 of each 21-day cycle for six cycles. Patients in the weekly dose-fractionated carboplatin–paclitaxel group (group 3) received carboplatin AUC2 by intravenous infusion over 30–60 min and paclitaxel 80 mg/m^2^ by intravenous infusion over 1 h on days 1, 8, and 15 of each 21-day cycle for six cycles. Carboplatin dose was calculated using the Calvert formula with starting AUC determined by the method used to calculate renal function at trial entry. AUC5 was used when radioisotopic glomerular filtration rate, measured 24 h urinary creatinine clearance, and Wright formula estimation had been used, and AUC6 was used when the modified Cockcroft-Gault or Jelliffe formula estimation had been used.

In all groups, treatment proceeded on day 1 of each cycle if absolute neutrophil count was at least 1·0 × 10^9^ per L and platelet count was at least 75 × 10^9^ per L. In group 2, day 8 and day 15 paclitaxel was administered if absolute neutrophil count was at least 0·5 × 10^9^ per L and platelet count was at least 50 × 10^9^ per L; paclitaxel was omitted if these haematological parameters were not met. In group 3, day 8 and day 15 weekly carboplatin and paclitaxel were administered if absolute neutrophil count was at least 1·0 × 10^9^ per L and platelet count was at least 75 × 10^9^ per L; both drugs were deferred if these values were not met. Protocol-defined dose alterations (ie, delay, reduction, or omission) were allowed for haematological and other adverse events if deemed clinically necessary by the treating physician. Single agent carboplatin was accepted as protocol treatment if patients were unable to tolerate paclitaxel. In the event of carboplatin hypersensitivity, trial management guidelines were followed that allowed continuation of carboplatin with increased hypersensitivity prophylaxis, the use of a formal carboplatin desensitisation regimen, or a switch to cisplatin for severe or recurrent hypersensitivity. Assigned treatment was discontinued if any of the following occurred: progression (as defined by Response Evaluation Criteria in Solid Tumours [RECIST] version 1.1^20^) while on therapy, unacceptable toxicity, intercurrent illness that prevented further treatment, withdrawal of consent for treatment by the patient, or any alterations in the patient's condition that justified the discontinuation of treatment in the investigator's opinion. Adverse events were graded according to the National Cancer Institute Common Toxicity Criteria for Adverse Events (version 4.03), and were collected at baseline and at the beginning of each chemotherapy cycle, to reflect adverse events that had occurred since the last cycle.

Surgery was done either as IPS before randomisation, followed by six cycles of chemotherapy; or as planned DPS, with three cycles of chemotherapy, followed by surgery, then by three further cycles of chemotherapy. However, surgery could be done at a later date if deemed clinically appropriate by the local multidisciplinary team. Day 15 treatment was omitted from the chemotherapy cycle before DPS to prevent chemotherapy-related adverse events affecting surgical timing and the protocol recommended that surgery be done within 32 days of the start of the pre-surgery chemotherapy cycle.

During chemotherapy, patients were seen before administration on day 1 of each chemotherapy cycle, and an end-of-treatment visit was carried out 6 weeks after day 1 of the last cycle of protocol-defined chemotherapy. Thereafter, patients were followed up every 6 weeks from the end of treatment visit until 9 months after randomisation, then once every 3 months until 2 years after randomisation, then once every 6 months until 4 years after randomisation, and annually thereafter until trial closure. After disease progression, patients were followed up every 6 months.

In all patients, baseline disease assessment was done with CT of the abdomen and pelvis, and chest radiograph, and scans were repeated 6 weeks after the final cycle of assigned chemotherapy. In those with planned DPS, two additional CT scans were done, one after three cycles of chemotherapy to allow surgical planning and then another at 4 weeks after DPS. All imaging was reported using RECIST version 1.1.^20^ Serum CA125 tumour marker measurements were done at baseline, on day 1 of each treatment cycle, and at each follow-up visit. Serum CA125 concentrations were processed in local laboratories at each trial site using commercially available and validated immunoassays. During follow-up, routine imaging was not mandatory unless clinical symptoms suggestive of disease progression occurred or if the GCIG criteria for CA125 progression were met.^21^ In women in whom CA125 progression was noted in the absence of radiological progression as defined by RECIST version 1.1, repeat imaging was mandated once every 3 months until progression per RECIST version 1.1 was noted.

### Outcomes

The trial had two coprimary outcomes, progression-free survival and overall survival. For both outcomes, two comparisons were to be made: group 2 versus group 1 and group 3 versus group 1. Progression-free survival was calculated from the date of randomisation to the date of the first indication of disease progression or death from any cause, whichever occurred first. Disease progression was defined by RECIST version 1.1 on the basis of radiological, clinical, or symptomatic indicators of disease progression and did not include isolated, asymptomatic CA125 progression. No central review of the primary endpoint was done. Patients were censored on the date last seen, defined as either the date of last assessment when patient was confirmed to be alive with no progression or the date a patient was confirmed to be lost to follow-up or withdrawn from the trial. Overall survival was calculated from date of randomisation to the date of death from any cause.

Secondary outcomes were safety and quality of life, which have previously been reported in detail,^17^, ^18^ and health economics, which is not described here.

### Statistical analysis

ICON8 was powered to detect a hazard ratio (HR) of 0·75 in both progression-free survival and overall survival. For both these analyses, the main comparisons were between the control group (group 1) and each of the weekly experimental groups (ie, group 2 *vs* group 1 and group 3 *vs* group 1). For both progression-free survival and overall survival, the timing of primary analyses was event-driven. To determine the sample size, we estimated that 70% of patients would enter the trial follow the IPS pathway with an estimated median overall survival of 36 months, and 30% of patients would follow the DPS pathway with an estimated median overall survival of 30 months, giving an overall median overall survival of 34·2 months. Assuming exponential survival and comparing the groups using an unadjusted log-rank test, 602 events were required for each comparison for overall survival using a two-sided 97·5% CI to achieve 90% power, to take the multiple comparisons into account, giving a total sample size of 1485 patients.

We did efficacy analyses on an intention-to-treat basis, including all patients randomly assigned to treatment, unless consent for data to be used in analyses was withdrawn. We assessed safety in all patients who started at least one chemotherapy cycle. We used the unadjusted log-rank test as the primary test to determine if there was a difference between the Kaplan-Meier survival curves. We used a Cox proportional hazards model as the primary estimation of treatment effect alongside median survival with 95% CIs calculated using the Kaplan-Meier approach) if the proportional hazards assumption was satisfied. However, if we found evidence of non-proportional hazards (assessed using Schoenfeld residuals), we used the restricted mean survival time with 97·5% CIs as the primary measure of the estimation of the size of treatment effect.

An exploratory analysis with limited power comparing group 2 and group 3 was preplanned, in the event that both experimental groups were found to perform better than group 1 using the aforementioned tests. Subgroup survival analyses were preplanned to investigate the effect of weekly chemotherapy on survival in patients treated with IPS or who were planned for DPS (patients who had no surgery planned were included in the DPS group; overall survival and progression-free survival) and also to account for the randomisation stratification factors of FIGO stage, ECOG performance status, and histological subtype (overall survival), and were done in the same way as in the primary efficacy analyses.

p values of 0·025 or less were deemed to be statistically significant for purposes of the primary analysis. An independent data monitoring committee met annually and had overall oversight of all emerging data within the study. Early feasibility and safety analyses were done and have reported previously.^22^

We did all analyses using Stata (version 16.1). This study is registered with ClinicalTrials.gov, NCT01654146, and the ISRCTN registry, ISRCTN10356387.

### Role of the funding source

The funders had no role in the study design, data collection, data analysis, data interpretation, or writing of the report.

## Results

**Figure 1:**
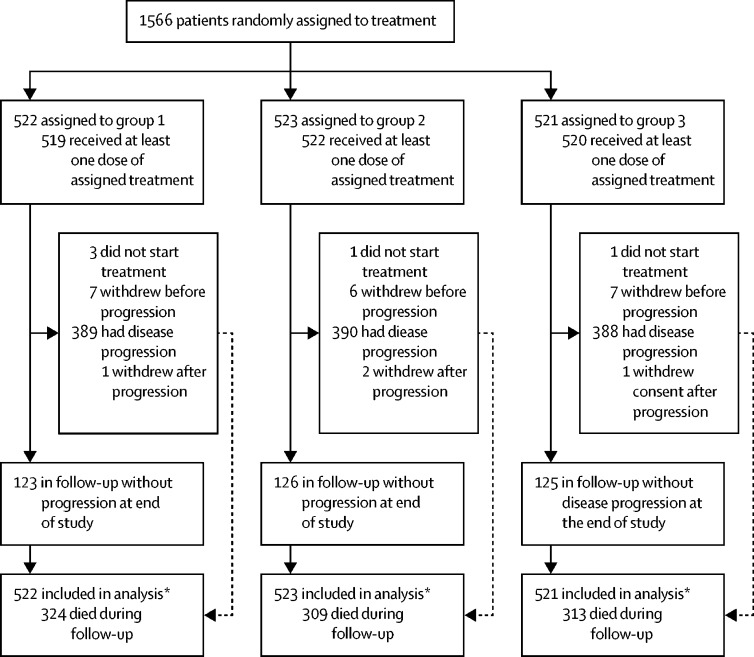
Trial profile End of study was March 2, 2020. *Follow-up was censored at time of withdrawal if patient withdrew consent for future data to be used.

Between June 6, 2011, and Nov 28, 2014, 1566 patients were eligible and randomly assigned to standard treatment (group 1; n=522), 3-weekly carboplatin and weekly paclitaxel (group 2; n=523), or weekly carboplatin and paclitaxel (group 3; n=521; figure 1). Demographic and disease characteristics are in table 1. Median age at randomisation was 62 years (IQR 54–68), 1073 (69%) of 1566 patients had high-grade serous carcinoma and 1119 (71%) had stage IIIC–IV disease. 745 (48%) of 1566 had debulking surgery before randomisation (ie, IPS) and commenced trial chemotherapy a median of 36 days (IQR 30–43) after their operation. 780 (50%) of 1566 were planned to undergo delayed surgery, while 41 (3%) were not considered candidates for future surgical intervention after multidisciplinary team review. Patients who had planned delayed primary surgery or had no surgery planned had more advanced disease at diagnosis than did those who had immediate surgery (773 [94%] of 821 DPS patients *vs* 346 [47%] of 745 IPS patients had stage IIIC–IV disease whereas 44 [5%] DPS patients *vs* 399 [54%] IPS had FIGO stage IC–IIIB disease; appendix p 1). Chemotherapy delivery by treatment group has been reported previously.^17^

**Table 1:** Baseline characteristics (intention-to-treat population)

		**Group 1 (n=522)**	**Group 2 (n=523)**	**Group 3 (n=521)**
Age, years		63 (55–68)	61 (54–67)	62 (53–68)
Participating group				
	UK	465 (89%)	468 (89%)	464 (89%)
	Australia and New Zealand	24 (5%)	23 (4%)	23 (4%)
	Mexico	14 (3%)	13 (2%)	16 (3%)
	South Korea	10 (2%)	12 (2%)	10 (2%)
	Ireland	9 (2%)	7 (1%)	8 (2%)
Location of cancer				
	Ovary (epithelial)	420 (80%)	424 (81%)	433 (83%)
	Fallopian tube	24 (5%)	27 (5%)	21 (4%)
	Primary peritoneal	77 (15%)	70 (13%)	65 (12%)
	Missing data	1 (<1%)	2 (<1%)	2 (<1%)
Histological type				
	High-grade serous	365 (70%)	346 (66%)	362 (69%)
	Low-grade serous	11 (2%)	12 (2%)	11 (2%)
	Serous (unspecified)	6 (1%)	7 (1%)	6 (1%)
	Clear cell	32 (6%)	41 (8%)	34 (7%)
	Endometrioid	26 (5%)	19 (4%)	22 (4%)
	Carcinosarcoma	2 (<1%)	7 (1%)	3 (1%)
	Mixed or other type	80 (15%)	91 (17%)	83 (16%)
FIGO 1988 stage				
	IC–IIA	56 (11%)	56 (11%)	52 (10%)
	IIB–IIC	47 (9%)	47 (9%)	37 (7%)
	IIIA–IIIB	43 (8%)	55 (11%)	54 (10%)
	IIIC	273 (52%)	266 (51%)	272 (52%)
	IV	103 (20%)	99 (19%)	106 (20%)
ECOG performance status				
	0	246 (47%)	250 (48%)	235 (45%)
	1	237 (45%)	230 (44%)	246 (47%)
	2	37 (7%)	40 (8%)	39 (7%)
	Missing data	2 (<1%)	3 (<1%)	1 (<1%)
Timing of surgery				
	Immediate	250 (48%)	247 (47%)	248 (48%)
	Delayed	258 (49%)	263 (50%)	259 (50%)
	Inoperable	14 (3%)	13 (2%)	14 (3%)

Data are n (%) or median (IQR). Group 1 was the control group, receiving carboplatin AUC5 or AUC6 and paclitaxel 175 mg/m^2^ every 3 weeks, group 2 received carboplatin AUC5 or AUC6 once every 3 weeks and weekly paclitaxel 80 mg/m^2^, and group 3 received carboplatin AUC2 and paclitaxel 80 mg/m^2^ once a week, all for a total of six 21-day cycles. AUC=area under the curve. ECOG=Eastern Cooperative Oncology Group. FIGO=International Federation of Gynecology and Obstetrics.

Five (<1%) of 1566 patients did not commence assigned treatment due to rapid clinical deterioration, and another 24 (2%) patients withdrew from trial follow-up before the final analysis, of whom 20 withdrew before disease progression and four after progression-free survival had been reached (figure 1). All patients were included in final analyses, with follow-up censored at the date of withdrawal if consent was withdrawn for further data collection.

**Figure 2:**
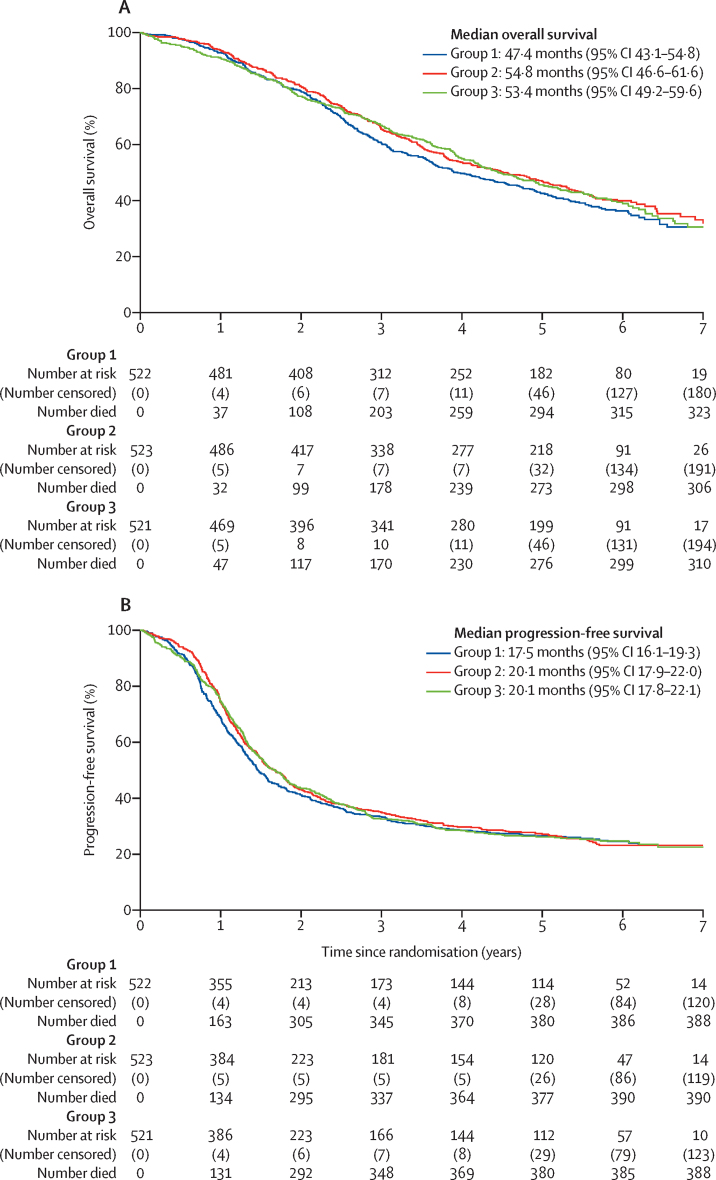
Overall survival (A) and progression-free survival (B) in the intention-to-treat population

The final follow-up visit took place on March 2, 2020, and the database was locked for analysis on March 31, 2020, when median follow-up was 69 months (IQR 61–75). As of database lock, 324 (62%) patients in group 1, 309 (59%) in group 2, and 313 (60%) in group 3 had died (figure 1; table 2). There was no evidence for non-proportional hazards for overall survival (p=0·84). Median overall survival was 47·4 months (95% CI 43·1–54·8) in group 1, 54·8 months (46·6–61·6) in group 2, and 53·4 months (49·2–59·6) in group 3 (table 2, figure 2A), but did not differ significantly between treatment groups (HR 0·87 [97·5% CI 0·73–1·05], p=0·092 for group 2 *vs* 1; HR 0·91 [0·76–1·09], p=0·24 for group 3 *vs* 1; table 2, figure 2A).

**Table 2:** Overall survival and updated progression-free survival results for all trial participants, intention-to-treat population

		**Group 1 (n=522)**	**Group 2 (n=523)**	**Group 3 (n=521)**
Number of deaths		324 (62%)	309 (59%)	313 (60%)
Median overall survival, months		47·4 (43·1–54·8)	54·8 (46·6–61·6)	53·4 (49·2–59·6)
	Unadjusted log-rank p value (*vs* group 1)	NA	0·16	0·35
	Hazard ratio (97·5% CI; p value)	1·0 (ref)	0·87 (0·73–1·05; p=0·092)	0·91 (0·76–1·09; p=0·24)
Patients with disease progression		389 (75%)	390 (75%)	388 (74%)
Median progression-free survival, months		17·5 (16·1–19·3)	20·1 (17·9–22·0)	20·1 (17·8–22·1)
	Unadjusted log-rank p value (*vs* group 1)	NA	0·39	0·46
	Hazard ratio (97·5% CI; p value)	1·0 (ref)	0·92 (0·78–1·09; p=0·39)	0·94 (0·80–1·10; p=0·49**)**
	Restricted mean survival time (97·5% CI)	23·9 (22·1–25·6)	25·3 (23·6–27·1)	24·8 (23·0–26·5)

Data are either n (%) or median (95% CI), unless otherwise stated. NA=not applicable.

As of data cutoff, progression had occurred in 389 (75%) patients in group 1, 390 (75%) in group 2, and 388 (74%) in group 3. The updated progression-free survival analysis (figure 2B) also showed no significant difference between randomised groups (table 2). Evidence of non-proportional hazards (p=0·037) was observed, and restricted mean survival time values were used as the primary estimate of treatment effect for progression-free survival (table 2). Because neither group 2 nor group 3 performed better than group 1, we did not do the exploratory analysis comparing group 2 and group 3.

**Figure 3:**
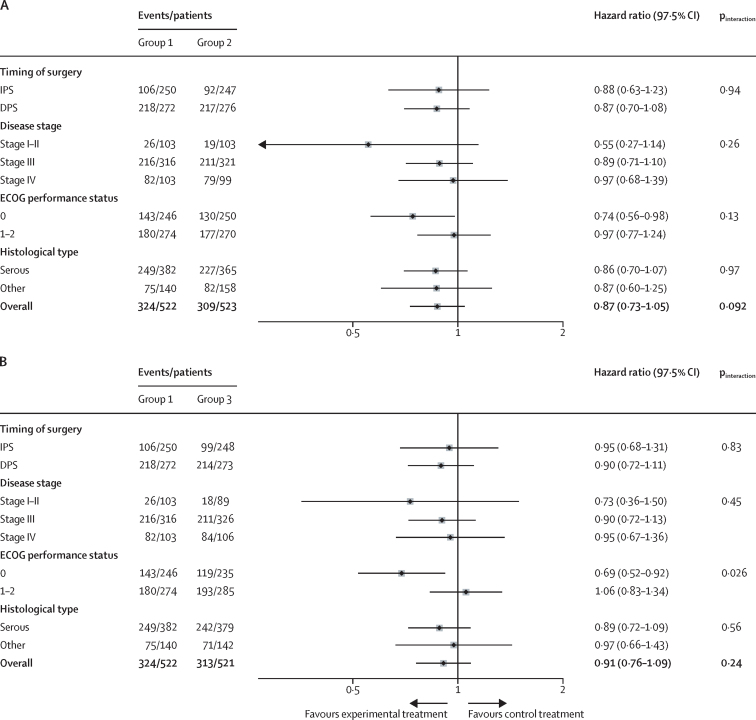
Subgroup analyses of overall survival in group 1 *vs* group 2 (A) and group 1 *vs* group 3 (B) DPS=delayed primary cytoreductive surgery. ECOG=Eastern Cooperative Oncology Group. IPS=immediate primary cytoreductive surgery.

In the preplanned subgroup analysis to assess the effect of weekly chemotherapy on survival in patients treated with IPS, or those who were planned for DPS (including no surgery planned), longer overall survival and progression-free survival were observed among patients who underwent IPS than among those managed through a DPS approach (appendix p 2). For patients who were managed using the DPS approach and those who had no planned surgery, median overall survival was 32·0 months (95% CI 30·3 to 35·5) in group 1, 38·6 months (35·8 to 42·3) in group 2, and 37·2 months (32·6 to 43·6) in group 3, and for patients managed via IPS median overall survival was 78·6 months (71·0 to not reached) in group 1, 93·6 months (77·1 to not reached) in group 2, and 85·8 months (72·9 to not reached) in group 3 (appendix p 2). 420 (56%) of patients who had IPS and 747 (91%) of patients who had DPS (or no planned surgery) had progression during the follow-up period (appendix p 2). Among both subgroups, no significant differences were observed between treatment groups for either overall survival or progression-free survival (figure 3; appendix pp 2–3).

In additional preplanned subgroup analyses, no significant difference in overall survival was observed in subgroups defined by timing of surgery, disease stage, or histological type (figure 3). Patients with an ECOG performance status of 0 benefitted from weekly treatment more than did those with an ECOG status of 1 or 2 (figure 3).

Safety and tolerability data, including dose alterations, have been previously reported.^17^ These data have been updated to include the small number of additional events reported after the database lock of the primary progression-free survival analysis (Feb 20, 2017), and are summarised in the appendix (pp 4–5). 511 patients in group 1, 514 in group 2, and 513 in group 3 started at least one cycle of assigned treatment. 215 (42%) of 511 patients in group 1 had at least one grade 3 or worse adverse event, compared with 320 (62%) of 514 patients in group 2 and 271 (53%) of 513 patients in group 3. The most common grade 3 or worse events were decreased neutrophil count (78 [15%] in group 1, 183 [36%] in group 2, and 154 [30%] in group 3), reduced white blood cell count (22 [4%] in group 1, 80 [16%] in group 2, and 71 [14%] in group 3), and anaemia (26 [5%] in group 1, 66 [13%] in group 2, and 24 [5%] in group 3). Incidence of sensory neuropathy of grade 2 or worse was similar across randomised groups (data not shown). No new drug-related serious adverse events occurred. Seven treatment-related deaths were reported (two in group 1, four in group 2, and one in group 3).

## Discussion

We found in our final analysis of the ICON8 study that, in a predominantly European population with high-risk epithelial ovarian cancer, the incorporation of weekly dose-dense paclitaxel in combination with either 3-weekly or weekly carboplatin into first-line multimodality treatment does not improve either progression-free survival or overall survival.

ICON8 was developed after the results of the Japanese JGOG 3016 trial were reported.^13^, ^14^ Although three subsequent phase 3 studies addressing the incorporation of treatment with weekly paclitaxel into first-line treatment for epithelial ovarian cancer have been done outside of Japan,^17^, ^23^, ^24^ ICON8 is the only study to our knowledge that has assessed overall survival as a coprimary outcome measure and provides the largest and most mature dataset to date. ICON8 was adequately powered to detect a clinically relevant survival difference between either of the two dose-dense weekly paclitaxel-containing experimental regimens and standard treatment scheduling. However, neither 3-weekly carboplatin and weekly dose-dense paclitaxel (group 2) nor weekly carboplatin and weekly dose-dense paclitaxel (group 3) improved progression-free survival or overall survival compared with standard 3-weekly chemotherapy scheduling in women with ovarian cancer who had undergone IPS or received neoadjuvant chemotherapy with a plan for DPS or with no planned surgery.

Previously, in the European MITO-7 trial,^23^ which was designed with coprimary endpoints of progression-free survival and quality of life, 822 women were randomly assigned to standard 3-weekly carboplatin AUC6 with paclitaxel 175 mg/m^2^ or a weekly carboplatin (AUC2)–paclitaxel (60 mg/m^2^) schedule without paclitaxel dose intensification. No significant difference in median progression-free survival was observed between the two administration schedules, although quality of life during chemotherapy was superior on weekly treatment. Only 22% of trial participants had died at the time of the database lock, restricting the validity of the overall survival analysis.^23^ Concurrently, investigators of the Gynecologic Oncology Group (GOG) 0262 trial^24^ recruited 692 women with stage II–IV epithelial ovarian cancer who were randomly assigned to receive standard 3-weekly chemotherapy AUC6 plus 3-weekly paclitaxel 175 mg/m^2^ or 3-weekly carboplatin AUC6 plus weekly paclitaxel 80 mg/m^2^. Additionally, 84% of trial participants opted to receive 3-weekly bevacizumab 15 mg/kg for up to 15 months alongside their cytotoxic treatment. At the time of the primary efficacy analysis, weekly treatment was not associated with an improvement in progression-free survival in the intention-to-treat population. However, in the 16% of women who elected not to receive bevacizumab, a 3·7 month increase in progression-free survival was noted (HR 0·62 [95% CI 0·40–0·95]; p=0·03).^24^ Overall survival was analysed as a secondary endpoint after an additional 26 months of follow-up to capture 304 events (44% total) with no difference detected between the two treatment groups (HR 0·94 [95% CI 0·72–1·23]).^24^

The absence of a survival benefit is also supported by real-world assessment of weekly dose-dense paclitaxel in the OPAL study, a national prospective cohort study of 634 women receiving first-line treatment for ovarian cancer conducted at 18 major oncology centres in Australia.^25^ This study showed that, after adjustment for age, FIGO stage, and histological subtype, dose-dense paclitaxel did not improve progression-free survival, but was associated with increased haematological and neurological toxicity.

This disparity in survival outcomes associated with dose-dense paclitaxel containing chemotherapy regimens between the Japanese JGOG 3016 trial and three phase 3 trials and a real-world evaluation study conducted predominantly in White women emphasise the potential importance of racial and ethnic differences in ovarian cancer biology or pharmacogenomic factors. Population-based assessment of the National Cancer Institute Surveillance, Epidemiology and End Results database identified better 5-year survival for Asian women than for White women with ovarian cancer.^26^ A subsequent pooled analysis of 7914 patients who entered ten first-line randomised NRG ovarian cancer systemic therapy trials identified 273 participants who were of Asian origin.^27^ These women were younger, with better performance status, and more likely to have cancers of early stage disease or with clear cell histology than their Caucasian counterparts. However, even after adjustment for these factors, Asian race was still a significant positive prognostic factor for overall survival (HR 0·84 [0·72–0·99]; p=0·04). Notably, pharmacogenomic studies have shown lower incidence of polymorphisms in drug-metabolising enzymes that might negatively affect paclitaxel activity in Asian populations than in Caucasian populations,^28^ and a post-hoc analysis of three phase 3 advanced lung cancer trials studying 3-weekly carboplatin–paclitaxel in US and Japanese patients determined that the racially associated single nucleotide polymorphisms CYP3A4*1B and *ERCC2K751Q* were associated with improved outcomes and greater haematological toxicity in Japanese patients than in US patients.^29^ The relatively low number of participants recruited from Korea and the fact that data on race and ethnicity were not collected prospectively in ICON8 prevents a meaningful subgroup analysis to determine whether race and ethnicity affects the efficacy of weekly dose-dense paclitaxel and was a weakness of our trial design. Further assessment of the role of race and ethnicity via a meta-analysis of the aforementioned phase 3 trials might be possible; however, we recommend that detailed race and ethnicity data, capturing sufficient information to allow granular analyses, should be incorporated into routine baseline demographic data collection in future phase 3 ovarian cancer trials.

Although the broad eligibility criteria adopted in ICON8 were considered a strength at the time of study design in 2009 by allowing wide participation and rapid recruitment, in retrospect and with our current knowledge of ovarian cancer biology, the inclusion of women with stage IC–IV disease and low-grade histologies (in particular low-grade serous) could be seen as a limitation on the ability of the trial to define the effect of dose-dense treatments. However, the small numbers of women with low-grade serous carcinoma and the 10% cap placed on the proportion of women with early stage high-risk disease mitigates these concerns. Additionally, at the time of ICON8 recruitment (2011–14), *BRCA* mutation testing was only available for women with a strong family history of breast or ovarian cancer, and so we are unable to assess the interaction between *BRCA* mutation status, other biomarkers of homologous recombination deficiency, and dose-dense chemotherapy administration within ICON8.

Notably, in ICON8 our reported overall survival is substantially longer than was anticipated at trial design. Our power calculation assumed a median overall survival of 36 months for patients who had IPS and 30 months for those treated with neoadjuvant chemotherapy and DPS on the basis of mature overall survival data from the ICON3, GOG0182-ICON5, and EORTC 55791 trials, which predominantly recruited patients with advanced stage disease.^6^, ^30^, ^31^ Our reported median overall survival was substantially longer than this estimate. This finding is reflective of the improvements in the number and efficacy of treatment options both in the maintenance setting and beyond disease progression for patients with ovarian cancer that have occurred in the past decade. In particular, we report a long median overall survival for patients who had IPS. 54% of patients who had IPS patients had FIGO stage IC–IIIB disease, and only 56% had disease progression during trial follow-up compared with 91% of patients who had DPS planned. These data reinforce that patients with early stage high-risk ovarian cancer have a very different prognosis to those with high-stage disease and we recommend that these groups should no longer be included in the same interventional trials. Additionally, although the longer than expected overall survival time in our trial meant that the analysis was done later than initially projected, because this analysis was triggered by a required number of events (602 in each comparison of group 1 *vs* group 2, and group 1 *vs* group 3), the analysis was still conducted with full information and was adequately powered to detect a target HR of 0·75.

Notably, a higher proportion of women entering ICON8 received neoadjuvant chemotherapy with a plan for DPS than was predicted in our power calculations. The results of the EORTC 55971 trial,^6^ published shortly before the start of the ICON8 enrolment period, showed that overall survival with neoadjuvant chemotherapy and DPS was non-inferior to IPS and was associated with less perioperative morbidity. Therefore, DPS rapidly became a more widely adopted standard approach for women with advanced epithelial ovarian cancer for whom complete primary cytoreduction is deemed unlikely in many UK centres.

This shift in real-world surgical practice is unlikely to have affected our ability to assess the effect of dose-dense chemotherapy because randomisation in ICON8 was stratified by surgical timing, and so the proportion of patients planned for DPS was balanced across all groups.

Although this study has clearly shown that dose-dense paclitaxel is not more efficacious than once every 3 week dosing when given in combination with carboplatin as part of the first-line management of ovarian cancer, it does not address whether modulating paclitaxel dose frequency might interact with maintenance treatment approaches that are now part of standard of care. This factor is particularly relevant in the context of anti-angiogenic therapy. Bevacizumab, an anti-VEGF monoclonal antibody, given concurrently with first-line 3-weekly carboplatin–paclitaxel and as subsequent maintenance therapy, has been shown to improve overall survival in women with high-risk FIGO stage III–IV ovarian cancer in subgroup analyses of two international phase 3 trials.^32^, ^33^ However, some research suggests that weekly paclitaxel might be a better backbone for targeted anti-angiogenic therapies than 3-weekly paclitaxel In an exploratory subgroup analysis of the AURELIA phase 3 trial of women with platinum-resistant recurrent ovarian cancer, a higher response rate was seen in the group randomly assigned to weekly paclitaxel plus bevacizumab than in those assigned to weekly paclitaxel alone (53% *vs* 30%) with superior overall survival (22·4 months *vs* 13·2 months; HR 0·65 [95% CI 0·42–1·02]), despite 38% of women receiving weekly paclitaxel alone crossing over to bevacizumab at disease progression.^34^ Although this was an exploratory analysis, and so not sufficiently powered, it is hypothesis generating and supports further assessment of this combination strategy in clinical trials. An exploratory biomarker assessment has also suggested that differential amplification of angiogenesis-related pathway genes is seen in women with recurrent ovarian cancer who have exceptionally durable responses to weekly paclitaxel.^35^ Whether the integration of both weekly paclitaxel and bevacizumab into the first-line management of disease in women with high-risk stage III–IV ovarian cancer will improve survival is being assessed in our follow-on ICON8B trial, which completed accrual in 2020. Although the first-line GOG 0262 trial, which did not show a survival benefit for weekly dose-dense paclitaxel compared with 3-weekly dosing both given with 3-weekly carboplatin, allowed women to receive bevacizumab, its use was based on investigator and patient choice rather than mandated per protocol.^24^ Moreover, not all participants met the criteria for high-risk disease, which is the population who typically gain most benefit from anti-angiogenic therapy, as identified retrospectively from the ICON7 trial.^33^

In summary, the mature overall survival results of the ICON8 trial support that weekly dose-dense paclitaxel should not be used as part of standard multimodality ovarian cancer treatment for women of predominantly European descent in the front-line setting. Whether the potential synergy between dose-dense paclitaxel and bevacizumab seen in the setting of platinum-resistant recurrent ovarian cancer will improve outcomes in first-line treatment is being investigated in the ICON8B trial.

## Data sharing

Data will be shared according to the MRC CTU's controlled access approach, based on the following principles: no data should be released that would compromise an ongoing trial or study; there must be a strong scientific or other legitimate rationale for the data to be used for the requested purpose; and investigators who have invested time and effort into developing a trial or study should have a period of exclusivity in which to pursue their aims with the data before key trial data are made available to other researchers. The resources required to process requests should not be underestimated, especially successful requests that lead to data being prepared for release. Therefore, adequate resources must be available to comply in a timely manner or at all, and the scientific aims of the study must justify the use of such resources. Data exchange complies with Information Governance and Data Security Policies in all of the relevant countries. Researchers wishing to access ICON8 data should contact mrcctu.icon8and8b@ucl.ac.uk in the first instance.

## Declarations of interests

ARC reports institutional funding from Cancer Research UK for the present Article, consulting fees from AstraZeneca, payment or honoraria for lectures and presentations from Clovis Oncology and AstraZeneca, and support for attending meetings from GSK/Tesaro. IAM reports consulting fees from Clovis Oncology, AstraZeneca, GSK/Tesaro, and Roche; honoraria for lectures of presentations from AstraZeneca and GSK; travel support for attending meetings from AstraZeneca and GSK; and participation on an Independent Data Monitoring Committee for Transgene. SBl reports consulting fees from Ellipses Pharma, Theolytics, Amphista, and RApport Global Strategic Services; honoraria for lectures or presentations from Nucana and the Norwegian Cancer Society; has a planned patent (WO1999062548A90); being a member of advisory boards for the UCB6114, TORCH, AVAT-M, MROC, and OCTOVA studies; being founder and treasurer of La-Related Protein Society; and has received institutional support to conduct clinical trials from Nucana, UCB, Nurix, Astex, BergenBio, MSD, Redx, and MiNA Therapeutics. JBr reports royalties from Inivata; consulting fees from AstraZeneca; payment or honoraria for lectures or presentations from GSK and AstraZeneca; holding two patents that have been issued (1818159.5 and 1818159.4); being a cofounder and shareholder of Tailor Bio; and being a previous cofounder and shareholder of Inivata. TJP reports consulting fees from AstraZeneca, Exact Health, and MSD, and support for attending meetings from Gilead. SS reports honoraria for lectures or presentations from AstraZeneca and MSD, participation in advisory board meetings for AstraZeneca, and is current President of the British Gynaecological Cancer Society. RL reports advisory works for and honoraria for educational events and support for travel and enrolment at conferences from AstraZeneca and GSK. MH reports consulting fees and payment or honoraria for lectures or presentations from GSK and Clovis Oncology. SBa reports grants from AstraZeneca, Tesaro, and GSK; consulting fees from Amgen, AstraZeneca, Genmabs, GSK, Immunogen, MSD, Merck Sereno, Mersana, Oncxerna, Seagen, and Shattuck Labs; payment or honoraria for lectures from Amgen, AstraZeneca, Clovis Oncology, GSK, Immunogen, MSD, Mersana, Pfizer, Roche, and Takeda; and is European Society for Medical Oncology (ESMO) Director of Membership. RMG reports grants from Clovis Oncology, Boehringer Ingelheim, and Lily/Ignyta; consulting fees from Clovis Oncology, AstraZeneca, MSD, GSK, Sotio, Immunogen, and Novartis; payment or honoraria for lectures or presentations from Clovis Oncology, AstraZeneca, and GSK; support for attending conferences from GSK; being a member of the Independent Data Monitoring Committee for the Glasgow Cancer Research Trials Unit and Swiss GO Trials Group; and receipt of equipment (drugs) to institution from GSK. CLH reports royalties from Cambridge University Press, support for attending conferences from Tesaro, being chair of the Wales Cancer Network Gynaecological Cancer Site Group, and a member of British Gynaecological Society Guidelines Group. JAL reports institutional research grants from AstraZeneca and MSD/Merck; payment or honoraria for lectures or presentations from Pfizer, AstraZeneca, GSK, MSD, Clovis Oncology, Eisai, Bristol Myers Squibb, Artios Pharma, VBL Therapeutics, and Neopharm; being chair of an Independent Data Monitoring Committee for Regeneron; being editor of the Gynaecological Clinical Practice Guidelines (for ESMO); and former Vice President of European Society of Gynaecological Oncology. All other authors declare no competing interests.
