# Supplementary material for: Weekly dose-dense chemotherapy in first-line epithelial ovarian, fallopian tube, or primary peritoneal cancer treatment (ICON8): overall survival results from an open-label, randomised, controlled, phase 3 trial
Source: Lancet Oncol. 2022 Jul;23(7):919–30. doi: 10.1016/S1470-2045(22)00283-2 (PMC9630160; doi:10.1016/S1470-2045(22)00283-2)
Supplement: Supplementary appendix [file mmc1.pdf]

THE LANCET
Oncology

Supplementary appendix

This appendix formed part of the original submission and has been peer reviewed. We post it as supplied by the authors.

Supplement to: Clamp AR, James EC, McNeish IA, et al. Weekly dose-dense chemotherapy in first-line epithelial ovarian, fallopian tube, or primary peritoneal cancer treatment (ICON8): overall survival results from an open-label, randomised, controlled, phase 3 trial. *Lancet Oncol* 2022; published online June 8. https://doi.org/10.1016/S1470-2045(22)00283-2.

Weekly dose-dense chemotherapy in first-line epithelial ovarian, fallopian tube, or primary peritoneal cancer treatment (ICON8): overall survival results from a GCIG open-label, randomised, controlled, phase 3 trial

Supplementary appendix

Supplementary Table 1. Baseline characteristics, split by timing of surgery

	IPS n=745		DPS n=821*		All n=1566	
Age (Years)	60	(52-66)	64	(56-69)	62	(54-68)
Participating group						
UK	635	(85%)	762	(93%)	1397	(89%)
Australia and New Zealand	50	(7%)	20	(2%)	70	(4%)
Mexico	21	(3%)	22	(3%)	43	(3%)
Korea	26	(3%)	6	(1%)	32	(2%)
Ireland	13	(2%)	11	(1%)	24	(2%)
Origin						
Ovary (epithelial)	658	(88%)	619	(76%)	1277	(82%)
Fallopian tube	63	(8%)	9	(1%)	72	(5%)
Primary peritoneal	23	(3%)	189	(23%)	212	(14%)
Missing data	1		4		5	
Histological type						
High grade serous	467	(63%)	606	(74%)	1073	(69%)
Low grade serous	14	(2%)	20	(2%)	34	(2%)
Serous (unspecified)	0	(0%)	19	(2%)	19	(1%)
Clear cell	94	(13%)	13	(2%)	107	(7%)
Endometroid	60	(8%)	7	(1%)	67	(4%)
Carcinosarcoma	10	(1%)	2	(<1%)	12	(1%)
Mixed or other type	100	(13%)	154	(19%)	254	(16%)
FIGO stage						
IC or IIA	160	(21%)	4	(<1%)	164	(10%)
IIB or IIC	123	(17%)	8	(1%)	131	(8%)
IIIA or IIIB	116	(16%)	36	(4%)	152	(10%)
IIIC	288	(39%)	523	(64%)	811	(52%)
IV	58	(8%)	250	(30%)	308	(20%)
ECOG performance status						
0	404	(54%)	327	(40%)	731	(47%)
1	313	(42%)	400	(49%)	713	(46%)
2	25	(3%)	91	(11%)	116	(7%)
Missing data	3		3		6	

* including 41 patients with no surgery planned

Supplementary Table 2. Overall Survival and Updated Progression Free Survival Results split by Immediate Primary and Delayed Primary Surgery Patients

	IPS						DPS					
	Group 1		Group 2		Group 3		Group 1		Group 2		Group 3	
	N=250		N=247		N=248		N=272		N=276		N=273	
Overall Survival												
Patients Died	106	(42%)	92	(37%)	99	(40%)	218	(80%)	217	(79%)	214	(78%)
Median OS (months)	78.6	(71.0,-)	93.6	(77.1,-)	85.8	(72.9,-)	32.0	(30.3,35.5)	38.6	(35.8,42.3)	37.2	(32.6,43.6)
p (vs. group 1)	-		0.26		0.59		-		0.13		0.26	
HR	1.0		0.88	(0.63,1.23)	0.95	(0.68,1.31)	1.0		0.87	(0.70,1.08)	0.90	(0.72,1.11)
p (proportional hazards)	0.19						0.12					
RMST	44.9	(44.0,45.7)	45.3	(44.4,46.1)	45.3	(44.4,46.1)	38.0	(36.5,39.5)	39.1	(37.7,40.6)	38.0	(36.5,39.5)
Progression Free Survival												
Patients progressed	147	(59%)	137	(55%)	136	(55%)	242	(89%)	253	(92%)	252	(92%)
Median PFS (months)	37.5	(12.9, -)	46.7	(17,3.-)	43.9	(17.7,-)	13.8	(9.0,23.1)	14.6	(10.7,22.6)	15.3	(9.0,23.0)
p (vs. group 1)	-		0.26		0.25		-		0.88		0.86	
HR	1.0		0.83	(0.63,1.09)	0.83	(0.63,1.09)	1.0		0.97	(0.79,1.19)	1.0	(0.81,1.22)
p (proportional hazards)	0.14		-		-		0.05		-		-	
RMST	38.0	(36.4,39.5)	39.6	(38.0,41.1)	39.4	(37.8,40.9)	25.9	(24.3,27.5)	26.9	(25.3,28.5)	26.3	(24.6,27.9)

Supplementary Figure 1A. Overall Survival for DPS population

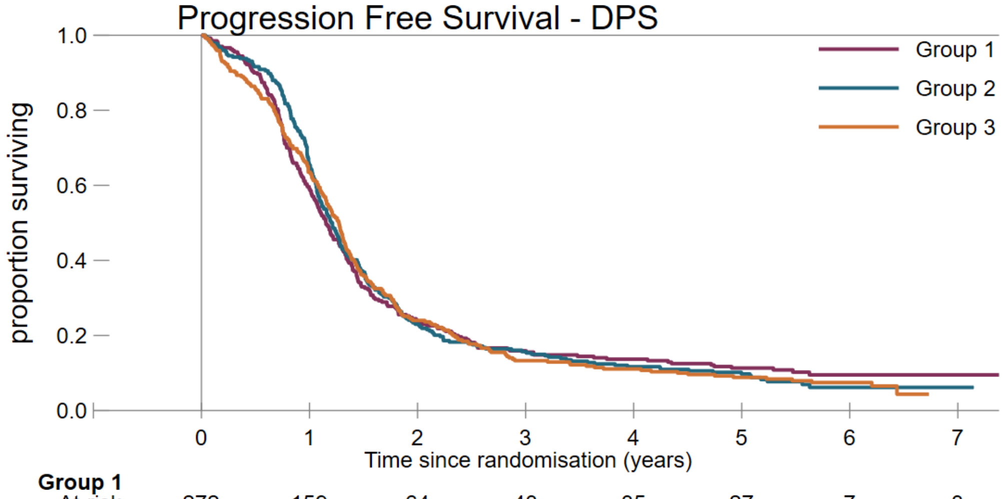

	0	1	2	3	4	5	6	7
**Group 1**								
At-risk	272	159	64	43	35	27	7	3
Censored	0	2	2	2	4	6	23	27
Died	0	111	206	227	233	239	242	242
**Group 2**								
At-risk	276	179	63	43	32	23	5	1
Censored	0	2	2	2	2	6	18	22
Died	0	95	211	231	242	247	253	253
**Group 3**								
At-risk	273	172	65	36	30	22	12	0
Censored	0	2	2	2	2	4	11	21
Died	0	99	206	235	241	247	250	252

Supplementary Figure 1B. Overall Survival for IPS population

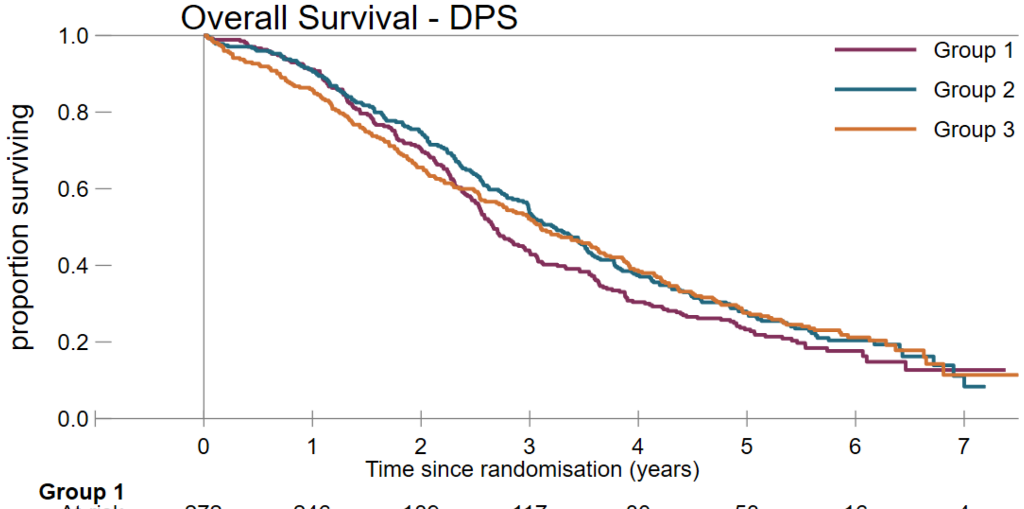

	0	1	2	3	4	5	6	7
**Group 1**								
At-risk	272	246	189	117	80	53	16	4
Censored	0	2	3	3	5	14	41	50
Died	0	24	80	152	187	205	215	218
**Group 2**								
At-risk	276	249	204	146	102	70	26	4
Censored	0	2	3	3	3	9	39	56
Died	0	25	69	127	171	197	211	216
**Group 3**								
At-risk	273	231	176	140	104	66	32	3
Censored	0	3	4	4	4	13	34	57
Died	0	39	93	129	165	194	207	213

Supplementary Table 3. Toxicity

	Group 1				Group 2				Group 3			
	Grades 1-2	Grade 3	Grade 4	Grade 5	Grades 1-2	Grade 3	Grade 4	Grade 5	Grades 1-2	Grade 3	Grade 4	Grade 5
Blood and lymphatic System disorders												
Anaemia	314 (61.4%)	25 (4.9%)	1 (0.2%)	0	369 (71.8%)	66 (12.8%)	0	0	358 (69.9%)	24 (4.7%)	0	0
Cardiac disorders												
Other	30 (5.9%)	3 (0.6%)	0	0	38 (7.4%)	4 (0.8%)	0	0	36 (7%)	2 (0.4%)	2 (0.4%)	0
Ear and labyrinth disorders												
Ear and labyrinth	44 (8.6%)	1 (0.2%)	0	0	36 (7.0%)	1 (0.2%)	0	0	36 (7%)	2 (0.4%)	0	0
Gastrointestinal disorders												
Constipation	361 (70.6%)	4 (0.8%)	2 (0.4%)	0	323 (62.8%)	2 (0.4%)	0	0	331 (64.5%)	6 (1.2%)	0	0
Diarrhoea	170 (33.3%)	12 (2.3%)	0	0	218 (42.4%)	10 (1.9%)	0	0	211 (41.1%)	14 (2.7%)	0	0
Dry mouth	91 (17.8%)	0	0	0	97 (18.9%)	0	0	0	109 (21.2%)	0	0	0
Mucositis oral	147 (28.8%)	1 (0.2%)	0	0	188 (36.6%)	0	0	0	176 (34.3%)	1 (0.2%)	0	0
Nausea	323 (63.2%)	13 (2.5%)	0	0	309 (60.1%)	13 (2.5%)	0	0	303 (59.1%)	5 (1%)	0	0
Vomiting	156 (30.5%)	16 (3.1%)	1 (0.2%)	0	147 (28.6%)	18 (3.5%)	0	0	116 (22.6%)	6 (1.2%)	0	0
Other	122 (23.9%)	7 (1.4%)	2 (0.4%)	0	150 (29.2%)	5 (1.0%)	0	0	156 (30.4%)	4 (0.8%)	0	1 (0.2%)
General disorders and administration site conditions												
Fatigue	442 (86.5%)	15 (2.9%)	0	0	441 (85.8%)	26 (5.1%)	0	0	449 (87.5%)	17 (3.3%)	0	0
Pain	266 (52.1%)	17 (3.3%)	0	0	257 (50.0%)	9 (1.8%)	0	0	252 (49.1%)	12 (2.3%)	0	0
Immune system disorders												
Allergic reaction	55 (10.8%)	8 (1.6%)	1 (0.2%)	0	47 (9.1%)	4 (0.8%)	2 (0.4%)	0	86 (16.8%)	4 (0.8%)	2 (0.4%)	0
Infections and Infestations												
Infection	103 (20.2%)	18 (3.5%)	3 (0.6%)	0	138 (26.8%)	25 (4.9%)	0	0	148 (28.8%)	18 (3.5%)	4 (0.8%)	0
Injury, poisoning and procedural complication												
Injury, poisoning and procedural complication	12 (2.3%)	1 (0.2%)	0	0	9 (1.8%)	2 (0.4%)	0	0	18 (3.5%)	2 (0.4%)	0	0
Investigations												
ALT or AST elevation	111 (21.7%)	4 (0.8%)	0	0	155 (30.2%)	4 (0.8%)	0	0	144 (28.1%)	5 (1%)	1 (0.2%)	0
Creatinine increased	50 (9.8%)	3 (0.6%)	0	0	74 (14.4%)	1 (0.2%)	0	0	34 (6.6%)	1 (0.2%)	0	0
Neutrophil count decreased	175 (34.2%)	61 (11.9%)	17 (3.3%)	0	194 (37.7%)	148 (28.8%)	35 (6.8%)	0	192 (37.4%)	135 (26.3%)	19 (3.7%)	0
Platelet count decreased	146 (28.6%)	17 (3.3%)	4 (0.8%)	0	193 (37.5%)	43 (8.4%)	5 (1%)	0	134 (26.1%)	11 (2.1%)	5 (1%)	0
Weight loss	71 (13.9%)	0	0	0	65 (12.6%)	1 (0.2%)	0	0	79 (15.4%)	1 (0.2%)	0	0
White blood cell count decreased	216 (42.3%)	20 (3.9%)	2 (0.4%)	0	265 (51.6%)	71 (13.8%)	9 (1.8%)	0	260 (50.7%)	69 (13.5%)	2 (0.4%)	0
Other	67 (13.1%)	8 (1.6%)	1 (0.2%)	0	95 (18.5%)	16 (3.1%)	3 (0.6%)	0	82 (16%)	10 (1.9%)	0	0
Metabolism and nutrition disorders												
Anorexia	137 (26.8%)	6 (1.2%)	0	0	118 (23.0%)	2 (0.4%)	0	0	122 (23.8%)	3 (0.6%)	0	0
Dehydration	21 (4.1%)	6 (1.2%)	0	0	16 (3.1%)	4 (0.8%)	0	0	17 (3.3%)	6 (1.2%)	0	0
Hypokalaemia	27 (5.3%)	4 (0.8%)	0	0	59 (11.5%)	7 (1.4%)	1 (0.2%)	0	35 (6.8%)	3 (0.6%)	1 (0.2%)	0

	Group 1				Group 2				Group 3			
	**Grades 1-2**	**Grade 3**	**Grade 4**	**Grade 5**	**Grades 1-2**	**Grade 3**	**Grade 4**	**Grade 5**	**Grades 1-2**	**Grade 3**	**Grade 4**	**Grade 5**
Other	59 (11.5%)	4 (0.8%)	0	0	72 (14.0%)	11 (2.1%)	1 (0.2%)	0	56 (10.9%)	9 (1.8%)	1 (0.2%)	0
Musculoskeletal/Soft tissue												
Arthralgia	174 (34.1%)	7 (1.4%)	0	0	107 (20.8%)	2 (0.4%)	0	0	97 (18.9%)	0	0	0
Muscle weakness	63 (12.3%)	0	0	0	57 (11.1%)	3 (0.6%)	0	0	58 (11.3%)	0	0	0
Myalgia	139 (27.2%)	6 (1.2%)	0	0	93 (18.1%)	1 (0.2%)	0	0	97 (18.9%)	0	0	0
Nervous system disorders												
Other	63 (12.3%)	4 (0.8%)	0	0	57 (11.1%)	3 (0.6%)	0	0	49 (9.6%)	2 (0.4%)	0	0
Renal and urinary disorder												
Renal and urinary disorder	57 (11.2%)	2 (0.4%)	0	0	61 (11.9%)	4 (0.8%)	0	0	77 (15%)	5 (1%)	0	1 (0.2%)
Respiratory, thoracic and mediastinal disorder												
Respiratory, thoracic and mediastinal disorder	95 (18.6%)	11 (2.2%)	0	1 (0.2%)	133 (25.9%)	4 (0.8%)	1 (0.2%)	0	120 (23.4%)	7 (1.4%)	1 (0.2%)	0
Skin and subcutaneous tissue disorder												
Alopecia	466 (91.2%)	0	0	0	469 (91.2%)	0	0	0	452 (88.1%)	0	0	0
Rash	104 (20.4%)	1 (0.2%)	0	0	166 (32.3%)	3 (0.6%)	1 (0.2%)	0	163 (31.8%)	5 (1%)	0	0
Surgical and medical procedure												
Surgical and medical procedure	19 (3.7%)	24 (4.7%)	3 (0.6%)	0	31 (6.0%)	20 (3.9%)	1 (0.2%)	0	36 (7%)	16 (3.1%)	0	0
Vascular disorders												
Thromboembolic event	14 (2.7%)	11 (2.2%)	2 (0.4%)	1 (0.2%)	20 (3.9%)	20 (3.9%)	1 (0.2%)	0	19 (3.7%)	20 (3.9%)	2 (0.4%)	0
Other	29 (5.7%)	4 (0.8%)	0	0	41 (8.0%)	2 (0.4%)	0	0	24 (4.7%)	1 (0.2%)	1 (0.2%)	0

Collaborators

List of all Recruiting Sites and PIs

Note: If a principal investigator for a site has changed over the course of the study, all principal investigators are listed.

Centre	Principal Investigator	No. patients recruited
Christie Hospital	Dr. Andrew Clamp	105
Hammersmith Hospital	Dr. Jonathan Krell<br>Prof. Hani Gabra	70
Clatterbridge Cancer Centre	Dr. Rosemary Lord	57
UCLH	Prof. Jonathan Ledermann	52
Freeman Hospital	Dr. Graham Dark	45
Addenbrooke's Hospital	Dr. Christine Parkinson<br>Dr. Helena Earl	43
University Hospital Coventry & Warwickshire	Mrs. Lucy McAvan<br>Prof. Chris Poole	43
Mount Vernon Hospital	Dr. Marcia Hall	41
Instituto Nacional de Cancerologia	Dr. Dolores Gallardo-Rincon	39
Beatson	Dr. Rosalind Glasspool	38
St Barts Hospital	Dr. Melanie Powell<br>Dr. Rowan Miller	35
Velindre Hospital	Dr. Rachel Jones<br>Dr. Louise Hanna	33
City Hospital (Birmingham)	Dr. Sarah Williams	33
Royal Surrey County Hospital	Dr. Sharadah Essapen	31
Bristol Haemtaology & Oncology Centre	Dr. Axel Walther	28
Seoul National University Hospital	Prof. Jae-Won Kim	27
Royal Devon & Exeter Hospital	Dr. Kate Scatchard	26
Maidstone Hospital	Dr. Jeff Summers	25
Nottingham University Hospital	Dr. Anjana Anand<br>Dr. Christopher Kent<br>Dr. Stephen Chan	25
Royal Shrewsbury Hospital	Dr. Abel Zachariah	25
Royal Derby Hospital	Dr. Mojca Persic	23
Broomfield Hospital	Dr. Helena Nam<br>Prof. Saad Tahir	22
Royal Marsden Hospital (Sutton)	Dr. Susana Banerjee	21
James Cook University Hospital	Dr. Louise Li	21
Royal Marsden Hospital (London)	Dr. Susana Banerjee	19
Guy's Hospital (London)	Dr. Ana Montes	19
Cheltenham General Hospital	Dr. Audrey Cook	18
St James University Hospital	Prof. Tim Perren	18
St Helens Hospital	Dr. Rosemary Lord	18
North Devon District Hospital	Dr. Kate Scatchard	17
Royal United Hospital	Dr. Rebecca Bowen	15
Royal Stoke University Hospital	Dr. Rajanee Bhana	15
Weston Park Hospital	Dr. Simon Pledge	14

Centre	Principal Investigator	No. patients recruited
Wexham Park Hospital	Dr. Marcia Hall	14
St George's Hospital	Dr. Fiona Lofts	13
Great Western Hospital	Dr. Omar Khan	13
Mater Misericordiae University Hospital	Dr. John McCaffrey	13
Singleton Hospital	Dr. Gianfilippo Bertelli<br>Dr. Rachel Jones	12
Queen Elizabeth the Queen Mother	Dr. Justin Waters	12
Musgrove Park Hospital	Dr. Clare Barlow	12
St John of God Hospital Subiaco	Dr. Andrew Dean	12
Royal Cornwall Hospital	Dr. John McGrane	11
Southend University Hospital	Dr. Helena Nam	11
Aberdeen Royal Infirmary	Dr. Trevor McGoldrick	11
Glan Clwyd Hospital	Dr. Anna Mullard	11
Churchill Hospital	Dr. Shibani Nicum	11
Warwick Hospital	Dr. Denise Hrouda	11
Royal Berkshire Hospital	Dr. Madhumita Bhattacharyya	10
Dorset County Hospital	Dr. Maxine Flubacher	10
Peterborough City	Dr. Sarah Ayers	10
Northampton General Hospital	Dr. Roshan Agarwal	10
Queen's Hospital (Romford)	Dr. Mary Quigley	10
Huddersfield Royal Infirmary	Mr. Deivasikamani Ramanujam	9
Ninewells Hospital	Dr. Michelle Ferguson	9
Airedale General Hospital	Dr. Dan Lee<br>Dr. Shazza Rehman	9
Liverpool Women's Hospital	Dr. Rosemary Lord	9
Westmead Hospital	Dr. Alison Brand	9
Yeovil District Hospital	Dr. Clare Barlow<br>Dr. Urmila Barthakur<br>Dr. Erica Beaumont	8
Royal Preston Hospital	Dr. Geraldine Skailes	8
Belfast City Hospital	Dr. Sarah McKenna	8
County Hospital, Stafford	Dr. Rjanee Bhana	8
Norfolk & Norwich University Hospital	Dr. Daniel Epurescu	8
Royal Lancaster Infirmary	Dr. Sarah Moon	8
Sir Charles Gairdner Hospital	Dr. Tarek Meniawy	8
Leicester Royal Infirmary	Dr. David Peel<br>Dr. Joanna Wood	7
Royal Sussex County Hospital	Dr. Rebecca Herbertson	7
Hinchingbrooke Hospital	Dr. Li Tee Tan	7
Auckland City Hospital	Dr. Kathryn Chrystal	7
Calvary Mater Newcastle	Dr. Janine Lombard	7
Queen Alexandra Hospital	Dr. Cheng Yeoh	7
Cumberland Infirmary	Dr. Sandeep Singhal	6
Torbay District General Hospital	Dr. Nangi Lo	6
Bedford Hospital	Dr. Sarah Smith	6

Centre	Principal Investigator	No. patients recruited
Victoria Hospital (Blackpool)	Dr. Andrew Hindley<br>Dr. Dennis Yiannakis	6
Doncaster Royal Infirmary	Dr. Simon Pledge	6
George Eliot Hospital	Dr. Vicky Kaur Sangha<br>Dr. Mark Hocking	6
Weston General Hospital	Dr. Waheeda Owadally	6
Withybush General Hospital	Dr. David Mark Davies<br>Dr. Maung Moe	5
Queen's Hospital Burton	Dr. Dorai Ramanathan<br>Dr. Lalith Seneviratne	5
Waterford regional Hospital	Dr. Paula Calvert	5
Ysbyty Gwynedd	Dr. Anna Mullard	4
Southampton General Hospital	Dr. Clare Green	4
Worthing Hospital	Dr. Rebecca Herbertson	4
Castle Hill Hospital	Dr. Georgios Bozas	4
New Cross Hospital	Dr. Margaret King<br>Dr. Rozenn Allerton	4
Centro Oncologico Estatal	Dr. Eva Maria Gomez	4
Royal Women's Hospital	Dr. Sumitra Ananda	4
ASAN Medical Center	Dr. Joo-hyum	4
York District Hospital	Dr. Angela Darby	3
Diana, Princess of Wales, Grimsby	Dr. Georgios Bozas	3
Ipswich Hospital	Dr. Jamey Morgan<br>Dr. Liz Sherwin<br>Mr. Pugazhenthi Pattu	3
Beaumont Hospital	Dr. Patrick Morris	3
Hereford County Hospital	Dr. Audrey Cook<br>Dr. Nina Reeve	3
Kettering General Hospital	Dr. Choi Iut-Mak	3
Border Medical Oncology	Dr. Christopher Steer	3
Bradford Royal Infirmary	Dr. Clara Sentamans<br>Dr. Daniel Lee<br>Dr. Chris Bradley	2
Poole Hospital	Dr. Maxine Flubacher<br>Dr. Richard Osborne	2
Bankstown Hospital	Dr. Sandra Harvey	2
Queen Elizabeth Hospital (Kings Lynn)	Dr. Margaret Daly	2
Royal Blackburn Hospital	Dr. Martin Hogg	2
Canberra Hospital	Dr. Sayed Ali	2
Mater Private Hospital	Dr. John McCaffrey	2
Royal Brisbane & Women's Hospital	Dr. Alison Hadley	2
Prince of Wales Hospital	Prof. Michael Friedlander	2
Peninsula Health	Dr. Yoland Antill	2
Townsville Hospital	Dr. Zulfiquer Otty	2
St George Hospital	Dr. Chee Lee	2
Mercy Hospital for Women	Prof. Linda Mileshkin	2
Manor Hospital	Dr. Indrakit Nalinika<br>Dr. Christos Mikropoulos	1
Lister Hospital	Dr. Marcia Hall	1

Centre	Principal Investigator	No. patients recruited
Chris O'Brien Lifehouse	Dr. Philip Beale	1
Christchurch Hospital	Dr. Michelle Vaughan	1
James Paget Hospital	Dr. Debashis Biswas	1
Western Hospital	Dr. Sumitra Ananda	1
St James's Hospital (Dublin)	Dr. Dearbhaile O'Donnell	1
Monash Health	Dr. Geraldine Goss	1
Gangnam Severance Hospital	Dr. Jae Hoon	1

MRC CTU Trial Staff

Janet Cairns, Kirsty Brown, Monique Tomiczek, Jennifer Petrie, Monica Mascarenhas, Gosala Gopalakrishnan, Emma Kent, Shabinah Ali, Aziza Mirza, Wendi Quan, Suzanne Freeman, Christopher Jarvis, Ann Marie Swart, Sally Stenning, Stephen Townsend, Chiara Borg, Dominic Mounsey, Katharine Goodall, Timothy Brush, Cheryl Jones, Lyndsey Castle, Ann Gelstharp, Anna Thomason, Tom Lazenby, Aishah Ahmed, Daniel King, Tatiana Sarfati, Kiran Peddireddi, Azizat Oyegoke, Bartlomiej Przybl, Christopher Coyle, Laura Farrelly, Rahela Choudhury, Jonathan Badrock, Babasola Popoola, Fuad Fananapazir, Elizabeth James, Adrian Cook, Richard Kaplan, Carlos Diaz, Zaheer Islam, Francesca Schiavone

IDMC Members

Graham Dunn, Alistair Ring, Simon Bach, Jan Bogaerts

ICON8 Trials Programme

ICON8: An international phase III randomised trial of dose-fractionated chemotherapy compared to standard three-weekly chemotherapy, following immediate primary surgery or as part of delayed primary surgery, for women with newly diagnosed epithelial ovarian, fallopian tube or primary peritoneal cancer

and

ICON8B: A phase III randomised trial investigating the combination of dose-fractionated chemotherapy and bevacizumab compared to standard three weekly chemotherapy and bevacizumab for the first-line treatment of women with newly diagnosed high-risk stage III-IV epithelial ovarian, fallopian tube or primary peritoneal cancer

Version: V7.0
Date: 26th June 2017

ISRCTN #:	ISRCTN10356387
EUDRACT #:	2010-022209-16
CTA #:	2010-022209-16
ENGOT #:	OV-13
MREC #:	11/LO/0043

Authorised by:

Name: **Dr Andrew Clamp**

Role**: Chief Investigator**

Signature:

Date:

Name: **Prof Richard Kaplan**

Role: **Programme Lead**

Signature:

Date:

GENERAL INFORMATION

This document was constructed using the MRC CTU Protocol Template Version 3.0. The MRC CTU endorses the Standard Protocol Items: Recommendations For Interventional Trials (SPIRIT) initiative. It describes the ICON8 trial, coordinated by the Medical Research Council (MRC) Clinical Trials Unit (CTU) at UCL (University College London), and provides information about procedures for entering patients into it. The protocol should not be used as an aide-memoire or guide for the treatment of other patients. Every care has been taken in drafting this protocol, but corrections or amendments may be necessary. These will be circulated to the registered investigators in the trial, but sites entering patients for the first time are advised to contact the Cancer Group, MRC CTU at UCL, London, UK, to confirm they have the most up-to-date version. MRC CTU at UCL may be referred to as MRC CTU throughout this document.

COMPLIANCE:

The trial will be conducted in compliance with the approved protocol, the Declaration of Helsinki 1996, the principles of Good Clinical Practice (GCP), Commission Directive 2005/28/EC and by Statutory Instrument 2004/1031 and subsequent amendments, the UK Data Protection Act (DPA number: Z5886415), and the National Health Service (NHS) Research Governance Framework for Health and Social Care (RGF).

International sites shall be responsible for the operational management of the ICON8 Trials Programme at their participating Clinical Sites and shall do so in compliance with the principles of Good Clinical Practice (GCP) as laid down by the ICH topic E6 (Note for Guidance on GCP), Commission Directive 2005/28/EC (the European Directive 2001/20/EC [where applicable])and in accordance with applicable local laws or national regulations.

SPONSOR:

The MRC is the trial sponsor and has delegated responsibility for the overall management of the ICON8 Trials Programme to the MRC CTU at UCL. Queries relating to MRC sponsorship of this trial should be directed to: Director of MRC CTU at UCL, Aviation House, 125 Kingsway, London WC2B 6NH, UK.

FUNDING:

The trial has public funding from Cancer Research UK through the UK Clinical Trials Awards and Advisory Committee (C1489/A12127) and CA1489/A17092), and will also be supported by Medical Research Council core funding.

AUTHORISATIONS AND APPROVALS

This trial was approved by the London-Chelsea research ethics committee and is part of the UK National Cancer Research Network (NCRN) portfolio.

TRIAL REGISTRATION

This trial has been registered with the EU Clinical Trials Register, where it is identified as ICON8.

RANDOMISATIONS

To randomise, call MRC CTU, Monday to Friday 9:00 – 17:00, UK time

Tel: +44 (0) 20 7670 4777

SAE REPORTING

Within 24 hours of becoming aware of an SAE, please fax a completed SAE form to the MRC CTU on:

Fax: +44 (0) 20 7670 4818

Recruitment into the ICON8 pathway is now complete.

The original ICON8B pathway was a phase III randomised trial investigating the combination of dose-fractionated chemotherapy and bevacizumab compared to either strategy alone for the first-line treatment of women with newly diagnosed high-risk stage III-IV epithelial ovarian, fallopian tube or primary peritoneal cancer.

As of 5th May 2017, following analysis of mature Progression-free survival data from the ICON8 trial,recruitment to arm B2 of ICON8B is suspended and participants will be randomised into a 2-arm comparison study (arm B1 vs arm B3) as described in section B.

TRIAL ADMINISTRATION

Please direct all queries to the Trial Managers at MRC CTU at UCL in the first instance; clinical queries will be passed to the Chief Investigator OR Trial Physician via the Trial Manager.

COORDINATING TRIALS UNIT

MRC Clinical Trials Unit
Aviation House
125 Kingsway
London
WC2B 6NH
UK

Switchboard: 020 7670 4700
Fax: 020 7670 **4818**
For ICON8 email: mrcctu.icon8@ucl.ac.uk
For ICON8B email: mrcctu.icon8b@ucl.ac.uk

MRC CTU STAFF

ICON8 Trial Manager:	Gosala Gopalakrishnan	Tel:	+44 (0) 207 670 4630
ICON8B Trial Manager:	Emma Kent	Tel:	+44 (0) 207 670 4857
ICON8 Data Manager:	Daniel King	Tel:	+44 (0) 207 670 4864
ICON8B Data Manager	Aishah Ahmed	Tel:	+44 (0) 207 670 4731
Statistician:	Adrian Cook	Tel:	+44 (0) 207 670 4639
Statistician:	Liz James	Tel:	+44 (0)20 7670 4682

CHIEF INVESTIGATOR AND PROJECT LEAD

Lead Chief Investigator
Dr Andrew Clamp
Cancer Research UK Department of Medical Oncology
Christie Hospital
Wilmslow Road
Manchester
M20 4BX

Tel: +44 (0) 161 446 3391
Fax: +44 (0) 161 446 3461
Email: Andrew.Clamp@christie.nhs.uk

MRC CTU Programme Lead
Prof Richard Kaplan
MRC CTU at UCL,
Aviation House,
125 Kingsway,
London
WC2B 6NH,
UK.

Tel: +44 (0) 207 670 4734
Fax: +44 (0) 207 670 4818
Email: r.kaplan@ucl.ac.uk

For full details of all trial committees, please see **Appendix 1: Trial Management Group**

SUMMARY OF TRIAL

Summary Information Type	SUMMARY DETAILS
Short Title of Trial	ICON8 trials programme, including ICON8 and ICON8B
Long Title of Trial	**ICON8:** An international phase 3 randomised trial of dose-fractionated chemotherapy compared to standard three-weekly chemotherapy, following immediate primary surgery or as part of delayed primary surgery, for women with newly diagnosed epithelial ovarian, fallopian tube or primary peritoneal cancer<br><br>And<br><br>**ICON8B:** A phase 3 randomised trial investigating the combination of dose-fractionated chemotherapy and bevacizumab compared to standard three weekly chemotherapy and bevacizumab for the first-line treatment of women with newly diagnosed high-risk stage III-IV epithelial ovarian, fallopian tube or primary peritoneal cancer
Protocol Version	7.0
Date	26th June 2017
ISRCTN #	ISRCTN10356387
NCT #	NCT01654146
EudraCT #	2010-022209-16
CTA #	2010-022209-16
MREC #	11/LO/0043
ENGOT ID	OV-13
Programme Design	The ICON8 trials programme encompasses two trial pathways for patients with newly diagnosed epithelial ovarian, fallopian tube or primary peritoneal cancer, and ovarian carcinosarcoma.<br><br>ICON8 is investigating dose-dense weekly chemotherapy scheduling in women with FIGO stage IC-IV disease. The original ICON8B pathway was investigating the combination of dose-fractionated chemotherapy and bevacizumab compared to either strategy alone for the first-line treatment of women with newly diagnosed high-risk stage III-IV epithelial ovarian, fallopian tube or primary peritoneal cancer.<br><br>As of 5th May 2017, following analysis of mature Progression-free survival data from the ICON8 trial, recruitment to arm B2 of ICON8B is suspended and participants will be randomised into a 2-arm comparison study arm B1 vs arm B3, investigating the combination of dose-dense chemotherapy and targeted anti-angiogenic therapy (bevacizumab) in a sub-group of women with high-risk stage III-IV ovarian cancer[1] to see if it is superior to standard three weekly chemotherapy and bevacizumab. High-risk is defined as women with FIGO (2013) stage IIIA1(ii), stage IIIA2 with positive retroperitoneal lymph nodes >1cm in diameter, stage IIIB or IIIC disease with >1cm residual disease following immediate primary surgery

[1] Unless otherwise specified in the text, where mentioned ovarian cancer refers to epithelial ovarian cancer, primary peritoneal cancer or fallopian tube cancer

	(IPS) or those receiving primary chemotherapy with or without delayed primary surgery (DPS), and all stage IV disease.
ICON8 Study Design	ICON8 is a randomised (1:1:1 ratio), three-arm, three-stage Gynaecologic Cancer InterGroup (GCIG) phase III trial designed to evaluate the safety and efficacy of dose-dense, dose-fractionated carboplatin-paclitaxel chemotherapy in the first-line treatment of ovarian cancer[2].<br><br>Patients eligible to join the ICON8 cohort will be randomised following IPS or following histological diagnosis if DPS or treatment with primary chemotherapy alone is planned.<br><br>ICON8 patients will be randomly assigned to:<br><br>• **Arm 1 (Control arm):** Carboplatin (AUC5[3] by intravenous infusion over 30-60 minutes) and paclitaxel (175mg/m^2 by intravenous infusion over 3 hours) on day 1 of a 21-day cycle. This is an internationally accepted control arm for the first-line treatment of ovarian cancer<br><br>• **Arm 2 (Research arm):** Carboplatin (AUC5[3] by intravenous infusion over 30-60 minutes) on day 1 and dose-fractionated weekly paclitaxel (80mg/m^2 by intravenous infusion over 1 hour) on day 1, 8 and 15 of a 21-day cycle<br><br>• **Arm 3 (Research arm):** Dose-fractionated weekly carboplatin (AUC2 by I.V infusion over 30-60 minutes) and weekly paclitaxel (80mg/m^2 by intravenous infusion over 1 hour) on day 1, 8 and 15 of a 21-day cycle<br><br>In patients who have undergone IPS:<br>• Chemotherapy must start within 8 weeks after surgery, and they will receive six cycles of chemotherapy.<br><br>In patients undergoing DPS:<br>• Should receive three cycles of chemotherapy initially<br>• Surgery should then take place within 10 days after cycle 3 day 22<br>• Following recovery from surgery, a further three cycles of chemotherapy should be given (six cycles in total)<br>• For patients in the research arms 2 and 3 undergoing DPS, cycle 3 day 15 chemotherapy should be omitted to reduce the likelihood of surgery being delayed for myelosuppression.<br><br>Following completion of chemotherapy, no anti-cancer treatment is permitted prior to protocol defined disease progression.

[2] Unless otherwise specified in the text, where mentioned ovarian cancer refers to epithelial ovarian cancer, primary peritoneal cancer or fallopian tube cancer
[3] In ICON8 the recommended dose of three-weekly carboplatin is AUC5 with a measured GFR or a GFR calculated by the Wright method. If the Cockcroft-Gault or Jelliffe formulae are used to calculate the GFR, the carboplatin dose is AUC6.

ICON8B Study Design	The original ICON8B pathway was a randomised three-arm, two-stage, Gynaecologic Cancer InterGroup (GCIG) phase III trial designed to evaluate the safety and efficacy of bevacizumab in combination with dose-dense, dose-fractionated carboplatin-paclitaxel chemotherapy compared to either strategy alone for the first-line treatment of high-risk stage III-IV ovarian cancer.<br><br>As of 5th May 2017, following analysis of mature Progression-free survival data from the ICON8 trial, recruitment to arm B2 of ICON8B is suspended and participants will be randomised into a 2-arm comparison study arm B1 vs arm B3, investigating the combination of dose-dense chemotherapy and targeted anti-angiogenic therapy (bevacizumab) in a sub-group of women with high-risk stage III-IV ovarian cancer to see if it is superior to standard three weekly chemotherapy and bevacizumab.<br><br>Patients eligible to join ICON8B will be randomly assigned to:<br><br>• **Arm B1 (Control arm):** Carboplatin (AUC5[4] by intravenous infusion over 30-60 minutes) and paclitaxel (175mg/m2 by intravenous infusion over 3 hours) plus bevacizumab (7.5mg/kg by intravenous infusion over 30-90 minutes) on day 1 of a 21-day cycle for 6 cycles followed by bevacizumab (7.5mg/kg by intravenous infusion over 30-90 minutes) as maintenance therapy to complete 18 cycles in total<br><br>• **Arm B3 (Research arm):** Carboplatin (AUC5[5] by intravenous infusion over 30-60 minutes) on day 1 and dose-fractionated weekly paclitaxel (80mg/m2 by intravenous infusion over 1 hour) on day 1, 8 and 15 of a 21-day cycle plus bevacizumab (7.5mg/kg by intravenous infusion over 30-90 minutes) on day 1 of a 21-day cycle for 6 cycles followed by bevacizumab (7.5mg/kg by intravenous infusion over 30-90 minutes) as maintenance therapy to complete 18 cycles in total.<br><br>In patients who have undergone IPS:<br>• Chemotherapy must start within 8 weeks after surgery<br>• If chemotherapy is started less than 28 days after surgery then bevacizumab must be omitted from cycle 1 in Arms B1 and B3.<br><br>Patients undergoing DPS:<br>• Should receive 3 cycles of chemotherapy initially, with<br>○ Arms B1 & B3, bevacizumab omitted from cycle 3<br>○ ArmB3, cycle 3 day 15 paclitaxel omitted to reduce the likelihood of surgery being delayed for myelosuppression

[4] In ICON8 and ICON8B the recommended dose of three-weekly carboplatin is AUC5 with a measured GFR or a GFR calculated by the Wright method. If the Cockcroft-Gault or Jelliffe formulae are used to calculate the GFR, the carboplatin dose is AUC6.

<table>
<tr><td></td><td>Surgery should then take place within 10 days after cycle 3 day 22
Following recovery from surgery, a further 3 cycles of chemotherapy will be given (6 cycles of carboplatin + paclitaxel in total)
If chemotherapy is started less than 28 days after surgery then bevacizumab will be omitted from cycle 4 in Arms B1 and B3.
Following completion of chemotherapy, no other anti-cancer treatment is permitted prior to protocol defined disease progression.</td></tr>
<tr><td>Type of Participants to be Studied</td><td>Patients with newly diagnosed epithelial ovarian, fallopian tube or primary peritoneal carcinoma (including ovarian carcinosarcoma) who meet the eligibility criteria for one of the following study cohorts:

ICON8
Patients with newly diagnosed, histologically confirmed, FIGO (1988) stage IC/IIA (high-risk histology) - IV disease
Patients may be randomised either following IPS or following histological diagnosis if DPS or treatment with primary chemotherapy alone is planned.
ICON8B
Patients with newly diagnosed, histologically confirmed high-risk stage III-IV ovarian cancer:
FIGO (2013) stage IIIA1(ii), stage IIIA2 with positive retroperitoneal lymph nodes >1cm in diameter, stage IIIB or IIIC disease with >1cm residual disease following immediate primary surgery (IPS) or those planned to undergo primary chemotherapy with or without DPS
FIGO (2013) stage IV disease either following IPS (any volume of residual disease permitted) or those planned to receive primary chemotherapy with or without DPS
No contraindications to receiving bevacizumab.</td></tr>
<tr><td>Study Hypothesis</td><td>The primary objectives of the ICON8 Trials Programme are to test:
Whether dose-fractionated chemotherapy is more effective than standard 3-weekly treatment for women with FIGO stage IC-IV ovarian cancer (ICON8).
Whether the combination of bevacizumab plus dose-fractionated chemotherapy prolongs progression-free survival compared to standard 3-weekly bevacizumab for women with high-risk stage III-IV ovarian cancer (ICON8B).</td></tr>
<tr><td>ICON8 Outcome Measures and Analysis</td><td>ICON8: will have three planned staged analyses. The outcome measures for each stage are summarised in Table 1

Table 1: Outcome Measures of ICON8 Stages<table><tr><th></th><th>Stages of ICON8</th></tr></table></td></tr>
</table>

<table>
<tr>
<td></td>
<td>
<table>
<tr><th></th><th>Stage 1</th><th>Stage 2</th><th>Final stage</th></tr>
<tr><td>Primary</td><td>Feasibility and safety of protocol treatment</td><td>9-month progression-free survival rate</td><td>Progression-free survival (PFS)and overall survival (OS)</td></tr>
<tr><td>Secondary</td><td>--</td><td>--</td><td>Toxicity
Quality of life
Health Economics</td></tr>
</table>
In stage 1, if pre-defined levels of deliverability are not met and/or pre-defined levels of toxicity exceeded, the research arms will be reconsidered. If a pre-defined level of activity is not demonstrated in stage 2, the research arms will be reconsidered. Assuming activity is demonstrated, accrual will continue into the final stage to assess efficacy.
</td>
</tr>
<tr>
<td>ICON8B Outcome Measures and Analysis</td>
<td>
ICON8B: Two planned staged analyses. The primary outcome measures for each stage are summarised in Table 2.

Table 2: Outcome Measures of ICON8B Stages
<table>
<tr><th></th><th>STAGE 1</th><th>STAGE 2</th></tr>
<tr><td>Primary</td><td>Safety of neo-adjuvant bevacizumab in patients undergoing DPS</td><td>Progression-free survival (PFS) and overall survival (OS)</td></tr>
<tr><td>Secondary</td><td>--</td><td>Toxicity
Quality of life
Health Economics</td></tr>
</table>
In Stage 1, if pre-defined levels of safety are not met the use of neo-adjuvant bevacizumab will be reconsidered. In Stage 2, there will be one primary superiority comparison: Arm B1 vs. Arm B3.
</td>
</tr>
<tr><td>Randomisation allocation ratio</td><td>ICON8: 1:1:1
ICON8B: 1:1</td></tr>
<tr><td>No. of Participants to be Studied</td><td>ICON8: 1485
ICON8B: 660</td></tr>
<tr><td>Duration</td><td>ICON8: 6 years (3.5 year recruitment period)
ICON8B: 9 years (4 year recruitment period)</td></tr>
<tr><td>Ancillary Studies/Sub studies</td><td>Translational Research Sub study - TRICON8 and TRICON8B
Health Economics
QoL Study</td></tr>
<tr><td>Sponsor</td><td>Medical Research Council</td></tr>
<tr><td>Funder</td><td>CRUK and MRC</td></tr>
<tr><td>Chief Investigator</td><td>Dr Andrew Clamp</td></tr>
<tr><td>MRC CTU Project Leader</td><td>Prof. Rick Kaplan</td></tr>
</table>

ICON8 TRIALS PROGRAMME STRUCTURE AND SCHEMA

Figure 1: Original ICON8 trials programme schema

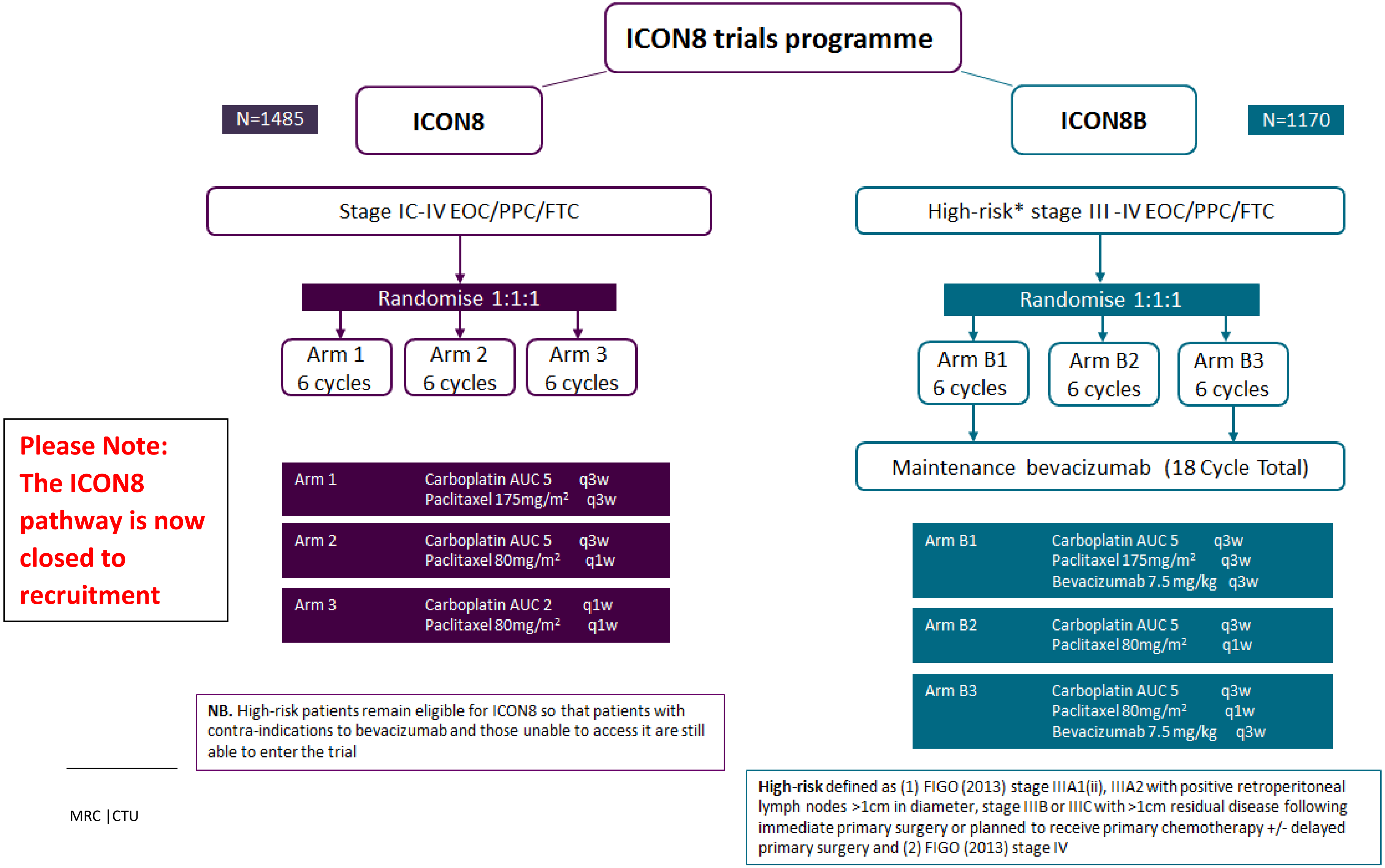

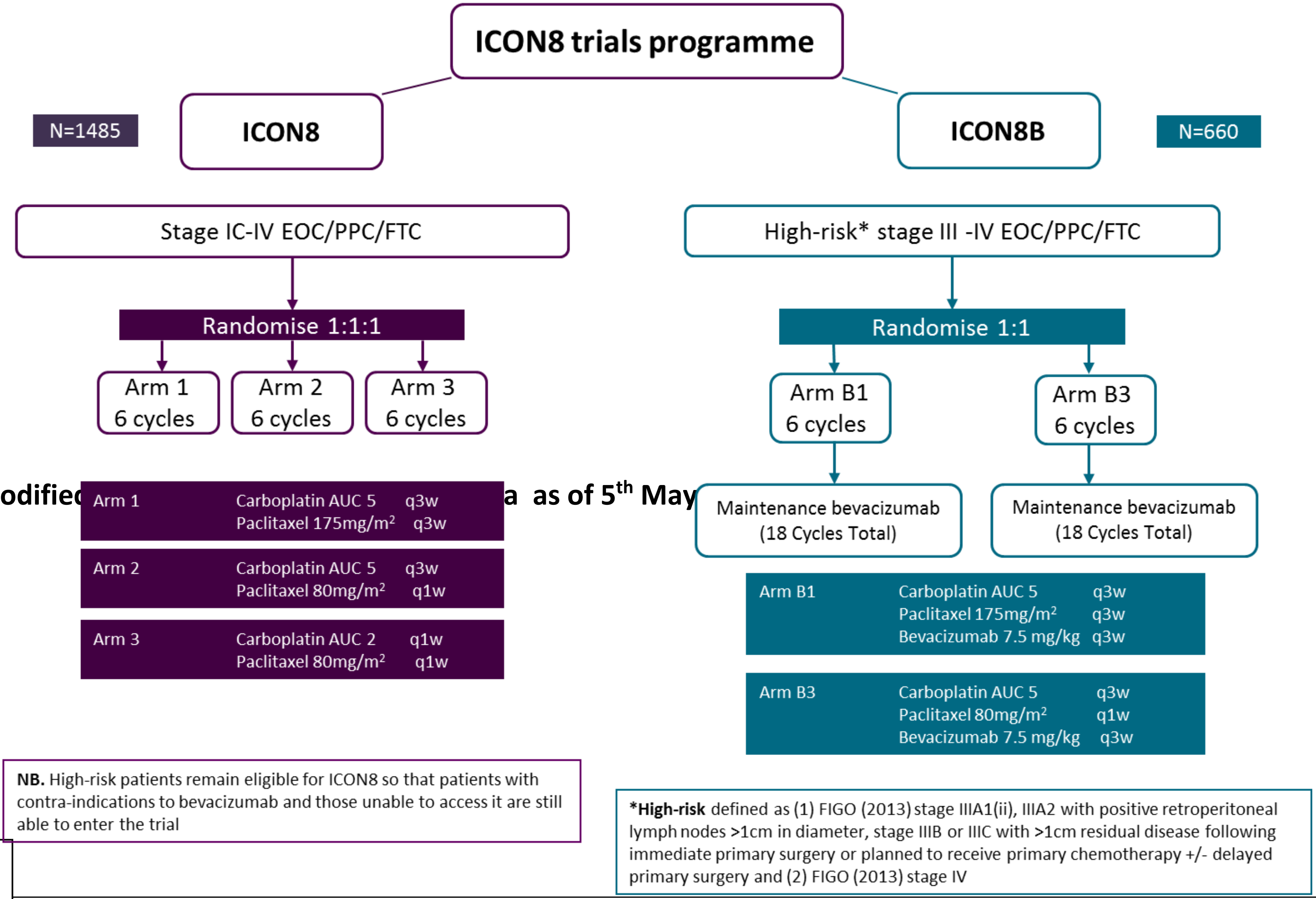

Figure 2: Modified [illegible] a as of 5th May [illegible]

Please Note: The ICON8 pathway is now closed to recruitment

Figure 3: ICON8 Trial Schema

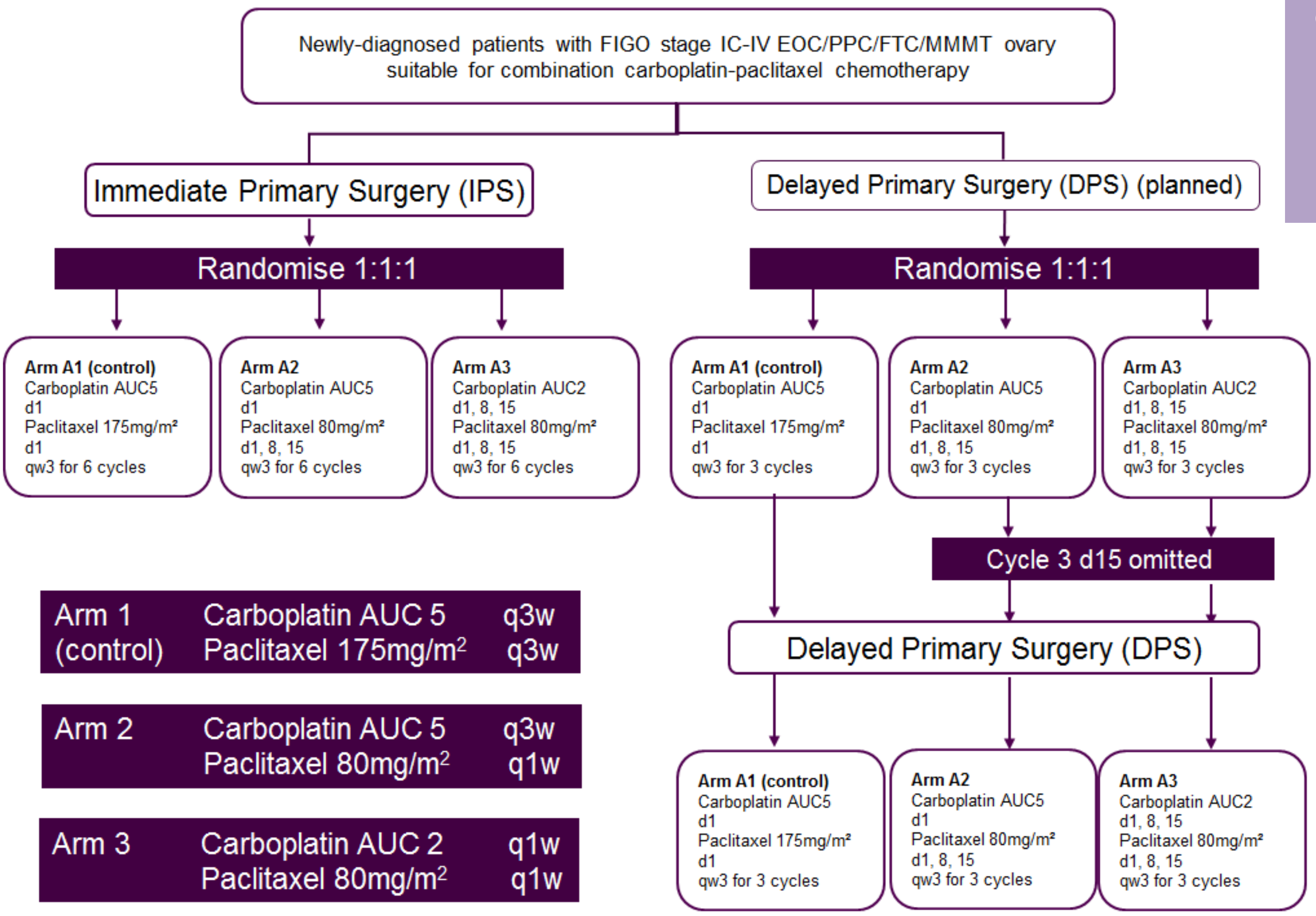

NB. Patients with inoperable Stage III/IV disease at diagnosis or in whom no operation is planned may also enter the trial

See Section A for details of ICON8 eligibility, investigations, treatment, follow-up and assessments. **Please note the ICON8 pathway closed to recruitment in Nov 2014.**

Table 3: ICON8 Outcome Measures

**Stage 1:**	
**Stage 1A**	Primary outcome measure: Feasibility and Safety in first 50 patients randomised per arm (approximately 150 patients)
**Stage 1B**	Primary outcome measure: Feasibility and Safety in first 50 patients randomised per arm with planned DPS (approximately 150 patients)
**Stage 2:**	Primary outcome measure: 9-month Progression Free Survival in first 62 patients randomised per arm (approximately 186 patients)
**Stage 3:**	Primary outcome measures: Progression Free Survival and Overall Survival<br>Secondary outcome measures: Toxicity, Quality of Life and Health Economics
**Ancillary studies:**	Translational Research (TRICON8) Health economics and QoL study

Teal Coloured tab denotes ICON8B specific page

Figure 4: ICON8B Trial Schema

Patient with newly diagnosed high-risk EOC/FTC/PPC
FIGO (2013) stage IIIA1(ii), IIIA2 with positive retroperitoneal lymph nodes >1cm in diameter, stage IIIB or IIIC with >1cm residual disease following IPS or planned to receive primary chemotherapy +/- DPS
FIGO (2013) stage IV after IPS with any volume of residual disease *or* planned to undergo primary chemotherapy +/- DPS
Suitable for combination carboplatin-paclitaxel chemotherapy and no contraindications to bevacizumab

Randomise 1:1

Arm B1 (Control arm)
Carboplatin AUC5* d1
Paclitaxel 175mg/m² d1
Bevacizumab 7.5mg/kg d1**
q3w for 6 cycles

Arm B3 (Research arm)
Carboplatin AUC5* d1
Paclitaxel 80mg/m² d1, 8, 15
Bevacizumab 7.5mg/kg d1**
q3w for 6 cycles

Chemotherapy phase
18 weeks

In DPS patients:
- Surgery after cycle 3
- Arm B1 & B3: Omit bevacizumab from cycle 3 (& cycle 4 if re-start <28 days after surgery)
- Arm B3: Omit d15 paclitaxel from cycle 3

Bevacizumab 7.5mg/kg d1
q3w to complete 18 cycles in total

Bevacizumab 7.5mg/kg d1
q3w to complete 18 cycles in total

Clinic assessment every 6 weeks (i.e. with every other cycle of bevacizumab) until completion of bevacizumab***

Maintenance phase
Up to 42 weeks

Follow-up visits
- End of chemotherapy to week 66 post-randomisation: 6-weekly follow-up in all Arms
- Week 66 to end of Year 2: 3-monthly follow-up
- Years 3,4,5 6: 6- monthly follow-up; annually thereafter
- Post-progression: trial follow-up 6-monthly in Year 1-6 then annually

Follow-up phase

* AUC6 if GFR is calculated by Cockcroft-Gault or Jeliffe formulae
** Omit bevacizumab from cycle 1 if chemotherapy commences within 28 days after IPS
*** If bevacizumab course is not completed by week 66 post-randomisation, continue 6-weekly visits until completion of course

See Section B for details of ICON8B eligibility, investigations, treatment, follow-up and assessments

Table 4: ICON8B Outcome Measures

**Stage 1:**	Safety of bevacizumab in combination with DPS
**Stage 2:**	Primary outcome measures: Progression-free Survival and Overall Survival<br><br>Secondary outcome measures: Toxicity, Quality of Life and Health Economics
**Ancillary studies:**	Translational Research (TRICON8B)<br>Health economics<br>QoL study

CONTENTS

GENERAL INFORMATION II

SUMMARY OF TRIAL V

ICON8 TRIALS PROGRAMME STRUCTURE AND SCHEMA X

CONTENTS 16

ABBREVIATIONS 23

1 BACKGROUND 27
1.1 STANDARD THERAPY FOR OVARIAN CANCER 27
1.1.1 Surgical treatment of ovarian cancer 27
1.1.2 Standard chemotherapy for ovarian cancer 27
1.2 DOSE-FRACTIONATED PACLITAXEL IN THE MANAGEMENT OF OVARIAN CANCER 28
1.2.1 Evidence from pre-clinical studies 28
1.2.2 Evidence in recurrent ovarian cancer 29
1.2.3 Evidence in first-line treatment 29
1.3 DOSE-FRACTIONATED CARBOPLATIN & PACLITAXEL IN THE MANAGEMENT OF OVARIAN CANCER 30
1.3.1 Evidence in recurrent ovarian cancer 30
1.3.2 Evidence in first-line treatment 30
1.4 INTEGRATING BEVACIZUMAB WITH DOSE-FRACTIONATED PACLITAXEL AND NEOADJUVANT CHEMOTHERAPY IN THE FIRST-LINE MANAGEMENT OF OVARIAN CANCER 32
1.5 RATIONALE FOR THE SUSPENSION OF ICON8B ARM B2 AND CONTINUATION OF THE 2-ARM (B1 VS B3) COMPARISON 33
1.6 INCORPORATION OF FIGO 2013 STAGING SYSTEM INTO ICON8 AND ICON8B 34

2. SELECTION OF SITES/CLINICIANS 35
2.1 GCIG GROUP APPROVAL 35
2.2 SITE/INVESTIGATOR INCLUSION CRITERIA 35
2.2.1 PI's Qualifications & Agreements 35
2.2.2 Adequate Resources 36
2.3 SITE APPROVAL 38

SECTION A: ICON8 40

A1 SELECTION OF PATIENTS 41
A1.1 PATIENT INCLUSION CRITERIA 41
A1.2 PATIENT EXCLUSION CRITERIA 42
A1.3 CONCOMITANT MEDICATIONS 43
A1.4 NUMBER OF PATIENTS 43
A1.5 SCREENING PROCEDURES AND PRE-RANDOMISATION INVESTIGATIONS 43
A1.5.1 Additional pre-chemotherapy tests 45
A1.6 INITIAL TUMOUR ASSESSMENT 45
A1.6.1 Immediate Primary Surgery Patients 45
A1.6.2 Delayed Primary Surgery Patients 45

A2 REGISTRATION AND RANDOMISATION 48
A2.1 INFORMATION REQUIRED FOR RANDOMISATION 48
A2.2 RANDOMISATION PROCEDURE 49
A2.2.1 Timing of Treatment Following Randomisation 49
A2.3 CO-ENROLMENT GUIDELINES 49

A3 TREATMENT OF PATIENTS 52
A3.1 GENERAL PRINCIPLES 52
A3.2 TREATMENT ARMS 52
A3.3 SURGERY 52
A3.3.1 Immediate Primary Surgery (IPS) 52
A3.3.1.1 Interval Debulking Surgery (IDS) 53
A3.3.2 Delayed Primary Surgery (DPS) 53
A3.3.2.1 Timing of delayed primary surgery 53
A3.3.2.2 Modifications to chemotherapy for patients undergoing DPS 53
A3.4 SPECIFIC DRUG INFORMATION 54
A3.4.1 Paclitaxel 55
A3.4.1.1 Three-weekly administration (Arm 1 only) 55
A3.4.1.2 Weekly administration (Arm 2 and 3) 56
A3.4.2 Carboplatin 57
A3.4.2.1 Carboplatin Dose 57
A3.4.2.2 GFR Limitations 58
A3.4.2.3 Requirements for re-assessment of GFR during chemotherapy 59
A3.5 TRIAL TREATMENT RECORDING 59
A3.6 CONCOMITANT THERAPY 59
A3.7 TREATMENT FOR PROGRESSION 60
A3.8 PROTOCOL TREATMENT DISCONTINUATION 60
A3.9 FOLLOW-UP 60

A4 DRUG SAFETY INFORMATION 62
A4.1 GENERAL INFORMATION ON PACLITAXEL AND CARBOPLATIN 62
A4.1.1 Expected Adverse Events with Paclitaxel and Carboplatin 62
A4.2 DOSE MODIFICATIONS, DELAYS AND OMISSIONS OF CARBOPLATIN AND PACLITAXEL: GENERAL PRINCIPLES 62
A4.3 ARM 1: 3-WEEKLY CARBOPLATIN-PACLITAXEL 65
A4.3.1 Haematological Toxicity 65
A4.3.2 Non-haematological toxicity 68
A4.3.2.1 Renal toxicity 68
A4.3.2.2 Neuropathy 68
A4.3.2.3 Mucositis 68
A4.3.2.4 Hypersensitivity 68
A4.3.2.5 Liver toxicity 69
A4.3.2.6 Other 69
A4.3.3 Stopping Paclitaxel or Carboplatin for toxicity: Alternative regimens 70
A4.3.3.1 Paclitaxel-specific toxicity 70
A4.3.3.2 Carboplatin-specific toxicity 70
A4.4 ARM 2: 3-WEEKLY CARBOPLATIN WITH WEEKLY PACLITAXEL 71
A4.4.1 Haematological Toxicity 71
A4.4.2 Non-haematological toxicity 72
A4.4.2.1 Renal toxicity 72
A4.4.2.2 Neuropathy 72

A4.4.2.3 Mucositis ... 72
A4.4.2.4 Hypersensitivity ... 72
A4.4.2.5 Liver toxicity ... 73
A4.4.2.7 Other ... 74
A4.4.3 Stopping Paclitaxel or Carboplatin for toxicity: Alternative regimens ... 74
A4.4.3.1 Paclitaxel-specific toxicity ... 74
A4.4.3.2 Carboplatin-specific toxicity ... 74
A4.5 ARM 3: WEEKLY CARBOPLATIN-PACLITAXEL ... 75
A4.5.1 Haematological Toxicity ... 75
A4.5.2 Non-haematological toxicity ... 75
A4.5.2.1 Renal toxicity ... 75
A4.5.2.2 Neuropathy ... 75
A4.5.2.3 Mucositis ... 76
A4.5.2.4 Hypersensitivity ... 76
A4.5.2.5 Liver toxicity ... 77
A4.5.2.6 Other ... 77
A4.5.3 Stopping Paclitaxel or Carboplatin for toxicity: Alternative regimens ... 77
A4.5.3.1 Paclitaxel-specific toxicity ... 77
A4.5.3.2 Carboplatin-specific toxicity ... 78

A5 ASSESSMENTS AND PROCEDURES ... 79
A5.1 VISIT SCHEDULE ... 79
A5.2 PROCEDURES FOR ASSESSING EFFICACY ... 79
A5.2.1 Tumour imaging ... 79
A5.2.1.1 Stage 2 analysis ... 80
A5.2.2 CA125 ... 80
A5.3 PROCEDURES FOR ASSESSING SAFETY ... 80
A5.4 TRANSLATIONAL RESEARCH SAMPLES ... 80
A5.5 PROCEDURES FOR ASSESSING QUALITY OF LIFE ASSESSMENTS ... 81
A5.6 HEALTH ECONOMICS ASSESSMENTS ... 82
A5.7 EARLY STOPPING OF FOLLOW-UP ... 82
A5.8 PATIENT TRANSFERS ... 82
A5.9 LOSS TO FOLLOW-UP ... 83

A6 TRIAL ASSESSMENTS SCHEDULES ICON8 ... 84
A6.1 Table 9: Trial Assessment Schedule for IPS Patients: ICON8 Arm 1 ... 84
A6.2 Table 10: Trial Assessment Schedule for IPS Patients: ICON8 Arm 2 ... 86
A6.3 Table 11: Trial Assessment Schedule for IPS Patients: ICON8 Arm 3 ... 88
A6.4 Table 12: Trial Assessment Schedule for DPS Patients: ICON8 Arm 1 ... 90
A6.5 Table 13: Trial Assessment Schedule for DPS Patients: ICON8 Arm 2 ... 92
A6.6 Table 14: Trial Assessment Schedule for DPS Patients: ICON8 Arm 3 ... 94

SECTION B: ICON8B ... 96

B1 SELECTION OF PATIENTS ... 97
B1.1 PATIENT INCLUSION CRITERIA ... 97
B1.2 PATIENT EXCLUSION CRITERIA ... 98
B1.3 CONCOMITANT MEDICATIONS ... 100
B1.3.1 Excluded concomitant medications ... 100
B1.4 NUMBER OF PATIENTS ... 101

B1.5 SCREENING PROCEDURES & PRE-RANDOMISATION INVESTIGATIONS 101
B1.5.3 Additional pre-chemotherapy tests 103
B1.6 INITIAL TUMOUR ASSESSMENT 103
B1.6.1 Immediate Primary Surgery Patients 103
B1.6.2 Delayed Primary Surgery Patients 104

B2 REGISTRATION & RANDOMISATION 107
B2.1 INFORMATION REQUIRED FOR RANDOMISATION 107
B2.2 RANDOMISATION PROCEDURE 108
B2.3 CO-ENROLMENT GUIDELINES 108

B3 TREATMENT OF PATIENTS 113
B3.1 INTRODUCTION 113
B3.1.1 Commencement of therapy 113
B3.1.2 Anticipated length of treatment course 114
B3.1.3 Use of bevacizumab around surgery 114
B3.2 TREATMENT ARMS 114
B3.2.1 Arm B1: 3-weekly carboplatin + paclitaxel + bevacizumab 114
B3.2.2 Arm B2: 3-weekly carboplatin + weekly paclitaxel 115
B3.2.3 Arm B3: 3-weekly carboplatin + weekly paclitaxel + bevacizumab 115
B3.3 SURGERY 116
B3.3.1 Immediate Primary Surgery (IPS) 116
B3.3.2 Delayed Primary Surgery (DPS) 117
B3.3.3 Modifications of SACT Required Around Surgery 119
B3.4 SPECIFIC DRUG INFORMATION 119
B3.4.1 Paclitaxel 120
B3.4.2 Carboplatin 122
B3.4.2.2 GFR Limitations 123
B3.4.2.3 Requirements for Reassessment of GFR During Chemotherapy 123
B3.4.3 Bevacizumab 124
B3.5 TRIAL TREATMENT RECORDING 125
B3.6 CONCOMITANT THERAPY DURING PROTOCOL TREATMENT 125
B3.7 TREATMENT AFTER PROGRESSION 126
B3.8 PROTOCOL TREATMENT DISCONTINUATION 126
B3.9 FOLLOW-UP 127
B3.10 GUIDANCE FOR PATIENTS RANDOMISED TO ARM B2 127

B4 DRUG SAFETY INFOMATION 128
B4.1 GENERAL INFORMATION ON PACLITAXEL, CARBOPLATIN AND BEVACIZUMAB 128
B4.1.2 DOSE MODIFICATIONS, DELAYS AND OMISSIONS OF CARBOPLATIN , PACLITAXEL AND BEVACIZUMAB: GENERAL PRINCIPLES 128
B4.2 ARM B1: 3-WEEKLY CARBOPLATIN-PACLITAXEL PLUS 3-WEEKLY BEVACIZUMAB 130
B4.2.1 Haematological Toxicity 130
B4.2.2 Non-Haematological Toxicity 133
B4.2.3 Stopping Paclitaxel or Carboplatin for Toxicity: Alternative Regimens 135
B4.3 ARM B2: 3-WEEKLY CARBOPLATIN WITH WEEKLY PACLITAXEL / ARM B3: 3-WEEKLY CARBOPLATIN AND BEVACIZUMAB WITH WEEKLY PACLITAXEL 136
B4.3.1 Haematological Toxicity 136
B4.3.2 Non-haematological Toxicity 137
B4.3.3 STOPPING PACLITAXEL OR CARBOPLATIN FOR TOXICITY: ALTERNATIVE REGIMENS 139
B4.4 BEVACIZUMAB MODIFICATIONS: DELAYS AND OMISSIONS DUE TO ADVERSE EVENTS (ARMS B1 AND B3) .. 140

B4.4.1 Bevacizumab: General Principles 140
B4.4.2 Bevacizumab Notable Events (DPS Patients Only) 141
B4.4.4 Specific Adverse Events of Bevacizumab and Recommendations for Treatment 142

B5 ASSESSMENTS & PROCEDURES 150
B5.1 VISIT SCHEDULE 150
B5.2 PROCEDURES FOR ASSESSING EFFICACY 151
B5.2.1 Tumour Imaging Assessments 151
B5.2.2 CA125 152
B5.3 PROCEDURES FOR ASSESSING SAFETY 152
B5.4 PROCEDURES FOR ASSESSING QUALITY OF LIFE 153
B5.5 HEALTH ECONOMICS ASSESSMENT 154
B5.6 TRANSLATIONAL RESEARCH SAMPLES 154
B5.7 EARLY STOPPING OF FOLLOW-UP 155
B5.8 PATIENT TRANSFERS 155
B5.9 LOSS TO FOLLOW-UP 155

B6 TRIAL ASSESSMENTS SCHEDULE FOR ICON8B 156
B6.1 TABLE 19: TRIAL ASSESSMENTS SCHEDULE FOR IPS PATIENTS ICON8B ARM B1: 3-WEEKLY CARBOPLATIN + PACLITAXEL + BEVACIZUMAB 156
B6.2 TABLE 20: TRIAL ASSESSMENTS SCHEDULE FOR IPS PATIENTS ICON8B ARM B2: 3-WEEKLY CARBOPLATIN + WEEKLY PACLITAXEL 160
B6.3 TABLE 21: TRIAL ASSESSMENTS SCHEDULE FOR IPS PATIENTS ICON8B ARM B3: 3-WEEKLY CARBOPLATIN + BEVACIZUMAB + WEEKLY PACLITAXEL 164
B6.4 TABLE 22: TRIAL ASSESSMENTS SCHEDULE FOR DPS PATIENTS ICON8B ARM B1: 3-WEEKLY CARBOPLATIN + PACLITAXEL + BEVACIZUMAB 169
B6.5 TABLE 23: TRIAL ASSESSMENTS SCHEDULE FOR DPS PATIENTS ICON8B ARM B2: 3-WEEKLY CARBOPLATIN + WEEKLY PACLITAXEL 174
B6.6 TABLE 24: TRIAL ASSESSMENTS SCHEDULE FOR DPS PATIENTS ICON8B ARM B3: 3-WEEKLY CARBOPLATIN + BEVACIZUMAB + WEEKLY PACLITAXEL 180
B6.7 ARM B1 AND B3 LEVEL 3 TRANSLATIONAL SAMPLE TIMEPOINTS (PLASMA SAMPLING) 185
B6.8 ARM B2 LEVEL 3 TRANSLATIONAL SAMPLE TIMEPOINTS (PLASMA SAMPLING) 186

3 SAFETY REPORTING 188
3.1 DEFINITIONS OF ADVERSE EVENTS AND ADVERSE REACTIONS 188
3.1.1 Medicinal Products 189
3.1.2 Adverse Events 189
3.1.3 Exempted Serious Adverse Events 189
3.2 ICON8B SPECIFIC NOTABLE EVENTS 190
3.2.1 ICON8B Specific Notable Adverse Events (All Arms) 190
3.2.2 ICON8B Specific Additional Notable Events Requiring Expedited Reporting 190
3.3 OTHER NOTABLE EVENTS (ICON8 AND ICON8B, ALL ARMS) 190
3.3.1 Pregnancy 190
3.4 CLINICAL TRIAL SITE/INVESTIGATOR RESPONSIBILITIES 191
3.4.1 Investigator Assessment 191
3.4.2 Notification 192
3.4.3 SAE Reporting Period 192
3.4.4 Notification Procedure (ICON8 and ICON8B): 192
3.5 MRC CTU RESPONSIBILITIES 193

4 STATISTICAL CONSIDERATIONS ... 195
4.1 ICON8 STATISTICAL CONSIDERATIONS ... 195
4.1.1 Method of Randomisation ... 195
4.1.2 Outcome Measures ... 195
4.1.3 Sample Size ... 195
4.1.4 Stage 1 ... 195
4.1.5 Stage 2 ... 196
4.1.6 Final stage ... 197
4.1.7 Interim Monitoring and Analyses ... 197
4.1.8 Brief Analysis Plan ... 197
4.2 ICON8B STATISTICAL CONSIDERATIONS ... 198
4.2.1 Method of Randomisation ... 198
4.2.8 Analysis Plan (Brief) ... 201
4.3 QUALITY OF LIFE ANALYSIS ... 201

5. QUALITY ASSURANCE & CONTROL ... 203
5.1 RISK ASSESSMENT ... 203
5.2 MONITORING AT THE MRC CTU ... 203
5.3 ON-SITE MONITORING ... 203
5.4 DATA QUALITY ASSURANCE ... 204
5.4.1 Confidentiality of Trial Documents and Patient Records ... 204
5.4.2 Direct Access to Patient Records ... 204
5.4.3 Patient Confidentiality ... 205

6 REGULATORY & ETHICAL ISSUES ... 206
6.1 COMPLIANCE ... 206
6.1.1 Regulatory Compliance ... 206
6.1.2 Site Compliance ... 206
6.1.3 Data Collection & Retention ... 206
6.2 ETHICAL CONDUCT OF THE STUDY ... 206
6.2.1 Ethical Considerations ... 206
6.2.2 ICON8 Ethical Considerations ... 207
6.2.3 ICON8B Ethical Considerations ... 207
6.3 ETHICAL APPROVAL ... 207
6.4 COMPETANT AUTHORITY APPROVALS ... 208
6.5 OTHER APPROVALS ... 208
6.6 REGULATORY REQUIREMENTS FOR PATIENT ENROLMENT ... 208
6.7 TRIAL CLOSURE ... 209
6.8 ARCHIVING ... 209
6.9 DESTRUCTION OF ESSENTIAL DOCUMENTS ... 209

7 SPONSORSHIP AND INDEMNITY ... 210

8 ANCILLARY STUDIES ... 211
8.1 TRANSLATIONAL RESEARCH ... 211

9 FINANCE ... 212

10 OVERSIGHT & TRIAL COMMITTEES ... 213
10.1 TRIAL MANAGEMENT TEAM (TMT) ... 213

10.2 GCIG ICON8 TRIAL MANAGEMENT GROUP (TMG) ... 213
10.3 INDEPENDENT TRIAL STEERING COMMITTEE (TSC) ... 213
10.4 INDEPENDENT DATA MONITORING COMMITTEE (IDMC) ... 213

11 PUBLICATION ... 215

12 PROTOCOL AMENDMENTS ... 216

13 REFERENCES ... 217

14 APPENDICES ... 222
APPENDIX 1 TRIAL MANAGEMENT GROUP ... 223
APPENDIX 2 1988 FIGO STAGING ... 225
APPENDIX 2.1 FIGO STAGING: CARCINOMA OF THE OVARY ... 225
APPENDIX 2.2 FIGO STAGING: CARCINOMA OF THE FALLOPIAN TUBE[47] ... 226
APPENDIX 2.3 PRIMARY PERITONEAL CARCINOMA STAGING ... 228
APPENDIX 2.4 OVARIAN CARCINOSARCOMA STAGING ... 228
APPENDIX 3 FIGO 2013 STAGING ... 229
APPENDIX 4 ECOG PERFORMANCE STATUS[48] ... 230
APPENDIX 5 CALCULATION AND MEASUREMENT OF GFR TO DETERMINE CARBOPLATIN DOSE ... 231
APPENDIX 5.1 CALCULATION OF GFR USING THE WRIGHT FORMULA[49] ... 231
APPENDIX 5.2 CALCULATION OF GFR USING THE JELLIFFE FORMULA[50] ... 232
APPENDIX 5.3 CALCULATION OF GFR USING THE COCKCROFT-GAULT FORMULA[51] ... 232
APPENDIX 6 GCIG CONSENSUS STATEMENT ON MINIMUM SURGICAL STANDARDS[5] ... 233
APPENDIX 7 COMMON TERMINOLOGY CRITERIA FOR ADVERSE EVENTS ... 234
APPENDIX 8 EVALUATION OF RESIDUAL DISEASE, EVALUATION OF AND DEFINITION OF PROGRESSION ... 241
APPENDIX 8.3.4 ROLE OF SERUM CA125 IN ASSESSMENT OF PROGRESSION ... 243
APPENDIX 9 PATIENT INFORMATION SHEETS AND INFORMED CONSENT FORMS GUIDANCE ... 245
APPENDIX 10 QUALITY OF LIFE - INFORMATION SHEET FOR CLINICIANS ... 246
APPENDIX 11 TRICON8 ... 247
APPENDIX 12 TRICON8B ... 249
APPENDIX 13 CHEMOTHERAPY RESPONSE SCORE ... 251
APPENDIX 14 MULTI-OMICS IMAGING ANALYSIS ... 251

ABBREVIATIONS

Abbreviation	Expansion
**ADL**	Activities of Daily Living
**AE**	Adverse Event
**ALP**	Alkaline Phosphatase
**ALT**	Alanine Aminotransferase/Serum Glutamic Pyruvic Transaminase / SGPT
**ANC**	Absolute Neutrophil Count
**APTT**	Activated ProThrombin Time
**AR**	Adverse Reaction
**AST**	Aspartate Aminotransferase/Serum Glutamic Oxaloacetic Transaminase /SGOT
**AUC**	Area under the plasma drug concentration versus time curve
**BSA**	Body Surface Area
**BUN**	Blood Urea Nitrogen
**Ca**	Calcium
**CA125**	Cancer Antigen 125
**CHF**	Congestive Heart Failure
**CIs**	Chief Investigator's
**CI**	Confidence Intervals
**Cr**	Serum Creatinine
**CrCl**	Creatinine Clearance
**CRF**	Case Report Form
**CTCAE**	Common Toxicity Criteria: Adverse Events
**CT Scan**	Computed Tomographic Scan
**CTU**	Clinical Trials Unit
**CXR**	Chest X-ray
**d**	Day
**DPS**	Delayed Primary Surgery
**ECOG**	Eastern Cooperative Oncology Group

Abbreviation	Expansion
**EOC**	Epithelial Ovarian Cancer
**EORTC**	European Organisation for Research and Treatment of Cancer
**EPO**	Erythropoietin
**FBC**	Full Blood Count
**FIGO**	International Federation of Gynaecology and Obstetrics
**FFPE**	Formalin-Fixed Paraffin Embedded
**FTC**	Fallopian Tube Carcinoma
**GCIG**	Gynaecologic Cancer InterGroup
**GCP**	Good Clinical Practice
**GFR**	Glomerular Filtration Rate
**GPIL**	General Practitioner Information Letter
**Hb**	Haemoglobin
**HE**	Health Economics
**HRQL**	Health-related quality of life
**IB**	Investigator's Brochure
**ICH**	International Conference of Harmonisation
**ICON**	International Collaborative Ovarian Neoplasm
**IDMC**	Independent Data Monitoring Committee
**IDMS**	Isotope Dilution Mass Spectrometry
**IDS**	Interval Debulking Surgery
**IMP**	Investigational Medical Product
**INR**	International Normalised Ratio
**IPS**	Immediate Primary Surgery
**K**	Potassium
**LFT**	Liver Function Test
**Mg**	Magnesium
**MHRA**	Medicines and Healthcare Products Regulatory Authority
**MMMT**	Malignant Mixed Müllerian Tumour
**MRC**	Medical Research Council

Abbreviation	Expansion
**MRI**	Magnetic Resonance Imaging
**N Saline**	Normal Saline
**Na**	Sodium
**NCRI**	National Cancer Research Institute
**NYHA**	New York Heart Association
**OS**	Overall Survival
**PFS**	Progression-Free Survival
**PI**	Principal Investigator
**PIS**	Patient Information Sheet
**PLT**	Platelets
**PPC**	Primary Peritoneal Cancer
**PS**	Performance Score
**PT**	ProThrombin Time
**PVC**	Polyvinyl Chloride
**QoL**	Quality of Life
**R&D**	Research and Development
**REC**	Research Ethics Committee
**RECIST**	Response Evaluation Criteria in Solid Tumours
**SACT**	Systemic Anti-Cancer Therapy
**SAE**	Serious Adverse Events
**SAR**	Serious Adverse Reaction
**SCr**	Serum Creatinine
**SOP**	Standard Operating Procedure
**SPC**	Summary of Product Characteristics
**SUSAR**	Suspected Unexpected Serious Adverse Reaction
**TNM**	Tumour, Node Metastases (staging system)
**TMG**	Trial Management Group
**TR**	Translational Research
**TRM**	Translation Research Manual

Abbreviation	Expansion
**TRT**	Treatment Related Toxicity
**TSC**	Trial Steering Committee
**TSH**	Thyroid Stimulating Hormone
**U&E**	Urea and Electrolytes
**UPCR**	Urine Protein to Creatinine Ratio
**ULN**	Upper Limit of Normal
**wk**	Week
**Wt**	Weight

1 BACKGROUND

Ovarian carcinoma is the fifth most common cancer in women in the United Kingdom (UK) and the most lethal gynaecological malignancy. Worldwide, there are an estimated 225,000 cases and 140,000 ovarian cancer related-deaths annually[1]. While there have been some improvements in survival in the last twenty years due to more aggressive surgical techniques and the routine use of platinum-taxane combination chemotherapy, the 5 year overall survival (OS) rate remains approximately 30% for patients with FIGO stage III/IV disease. There is clearly a significant need to develop more effective first-line treatment strategies for patients with ovarian carcinoma.

1.1 STANDARD THERAPY FOR OVARIAN CANCER

The recommended treatment for all but low-risk stage I ovarian carcinoma is immediate primary surgery (IPS) followed by systemic platinum-based chemotherapy.

1.1.1 Surgical Treatment of Ovarian Cancer

Primary surgery is required for definitive histopathological diagnosis and FIGO staging. Surgical debulking of ovarian cancer that achieves maximal cytoreduction with no visible residual disease (optimal debulking) has a positive effect on prognosis in advanced ovarian cancer. There are internationally accepted GCIG consensus guidelines on the standards of surgery for ovarian cancer, which should comprise total abdominal hysterectomy, bilateral salpingo-oophorectomy, omentectomy and debulking aiming for no residual disease. However, it is clear that optimal debulking is not achieved in a substantial minority of patients who undergo IPS. Indeed, 47% of patients randomised within ICON3[2] and 25% included in GOG0182-ICON5[3] had suboptimally debulked disease. This is either due to the extent of the disease or because of severe morbidity engendered by the presence of ascites, pleural effusions, venous thromboembolism or hypoalbuminaemia. In such patients, there is often significant post-operative morbidity delaying or preventing the use of post-operative chemotherapy. This has increasingly led to the adoption of an approach to treatment using primary chemotherapy to achieve a reduction in tumour burden, which is often associated with an improvement in performance status of the patient, with delayed primary surgery (DPS) performed after three cycles of chemotherapy.

Importantly, the results of EORTC 55971[4] which randomised 718 patients with operable IIIC-IV ovarian cancer to IPS or DPS, has shown similar outcomes with respect to PFS and OS, irrespective of whether IPS or DPS was employed; median OS was 29 months for IPS and 30 months for DPS (ITT analysis HR 0.98; 95% CI 0.84-1.13). Perioperative morbidity and mortality, however, were lower with DPS. It is apparent that, following the recent publication of these results, the current clinical trend towards DPS will increase further to include more women with advanced operable disease. It is, therefore, essential that any new front-line trial in ovarian cancer such as ICON8 is open to patients in whom a clinical decision has been made to perform DPS.

1.1.2 Standard Chemotherapy for Ovarian Cancer

The 3rd International GCIG Ovarian Cancer Consensus Conference[5] recommended that the control arm in first-line clinical trials of systemic treatment for ovarian cancer should be 6 cycles of

combination intravenous (IV) chemotherapy with carboplatin (AUC5 to AUC7.5) and paclitaxel (175mg/m^2 given as a three hour infusion) administered every three weeks. This is considered an international standard of care and the addition of a third cytotoxic[3] or substitution of an alternative drug for paclitaxel[6, 7] has not improved survival outcomes.

Two phase III randomised controlled trials have now been published that show improvement in PFS with the addition of concurrent and maintenance three-weekly bevacizumab to 6 cycles of three-weekly carboplatin-paclitaxel. GOG-218 is a randomized double-blind placebo controlled trial of 1873 women with stage III-IV ovarian cancer with residual disease following debulking surgery. It reported a statistically significant 3.8 month improvement in PFS (10.3 to 14.1 months, HR 0.72; 95%CI 0.63-0.82) with concurrent and maintenance bevacizumab at a dose of 15mg/kg every 3 weeks for up to 15 months.[8] ICON7 is a randomized controlled trial of 1528 women with high-risk early stage or advanced ovarian cancer that used a lower dose of bevacizumab (7.5mg/kg) given concurrently and as maintenance for up to 12 months. Overall it reported a 1.5 month improvement in PFS (20.3 to 21.8 months, HR 0.81; 95% CI 0.70-0.94)[9] at the expense of a small but clinically significant decrease in quality of life.[10] In a pre-planned sub-group analysis of high-risk patients (stage IV or stage III with >1cm residual disease), a more clinically meaningful improvement in PFS of 3.6 months (14.5 to 18.1 months) was seen. On the basis of these trials, the European Medicines Agency has granted a marketing authorisation approving bevacizumab use in combination with carboplatin-paclitaxel for the first-line treatment of Stage IIIB-IV ovarian cancer at a dose of 15mg/kg and for up to 15 months duration of maintenance therapy.

Recently, the final OS analysis of the ICON7 trial has been presented (ECCO 2013). While no effect on OS associated with the addition of bevacizumab was seen for the whole study group, a 4.8 month improvement in OS by restricted means analysis (34.5 to 39.3 months) and a 9.4 month increase in median survival (30.3 to 39.7 months, HR 0.78; 95% CI 0.63-0.97) was seen in the high-risk patient cohort.

However, no OS improvement has been noted in the GOG218 trial.[8] One of the notable differences between ICON7 and GOG218 is the rate of cross-over to bevacizumab after disease progression which was substantially higher in GOG218. This fact, together with improvements in PFS observed in trials of bevacizumab in combination with chemotherapy in both platinum-sensitive and resistant relapsed ovarian cancer[11, 12] indicate that although bevacizumab is an important new option in the management of ovarian cancer, it's exact positioning in the disease pathway is not yet clear. The use of carboplatin-paclitaxel chemotherapy alone therefore remains a standard treatment option in the first-line setting.

1.2 DOSE-FRACTIONATED PACLITAXEL IN THE MANAGEMENT OF OVARIAN CANCER

Dose-dense weekly administration of paclitaxel is an intriguing potential treatment strategy that may enhance antitumour activity and prolong survival in the first-line treatment of ovarian cancer.

1.2.1 Evidence from pre-clinical studies

Preclinical studies including in ovarian cancer models have demonstrated that fractionated paclitaxel dosing is associated with increased anti-angiogenic and pro-apoptotic effects, as well as reduced leukopenia.[13-17] It is also apparent that the duration of exposure to paclitaxel is a key determinant of its cytotoxic activity provided that a certain threshold concentration is achieved.[18] A reduction in time

between drug dosing has also been hypothesised to reduce the development of clones of paclitaxel-resistant cells.[19]

1.2.2 Evidence in recurrent ovarian cancer

In clinical studies in patients with platinum-resistant recurrent ovarian cancer, weekly paclitaxel alone, at doses between 40 and 100mg/m^2, is well-tolerated and has demonstrated activity[20-22] with a 25% response rate to 80mg/m^2 in tumours previously resistant to three-weekly paclitaxel treatment .[23] One randomised study demonstrated no difference in response rate between weekly and three-weekly paclitaxel but weekly paclitaxel at a median dose intensity of 77.6mg/m^2/wk was better tolerated.[24] The combination of weekly paclitaxel with three-weekly carboplatin has also demonstrated impressive response rates. One study investigated carboplatin AUC5 three-weekly and paclitaxel 80mg/m^2 weekly in 28 patients with platinum-sensitive recurrent ovarian cancer and achieved a 77% response rate with a median PFS of 14.5 months. A median of 6 cycles of therapy was administered and although 89% of patients required paclitaxel dose reductions during treatment to 60mg/m^2, 72% planned paclitaxel dose intensity was achieved.[25]

1.2.3 Evidence in first-line treatment

Importantly, weekly paclitaxel (80mg/m^2) has recently been compared to three-weekly paclitaxel (175mg/m^2), in combination with three-weekly carboplatin (AUC6), in the first-line treatment of ovarian cancer in the phase III Japanese Gynae Oncology Group (JGOG) trial 3016.[26] This trial randomised 637 women with FIGO stage II-IV ovarian cancer, 89% of whom had undergone primary debulking surgery. After a median follow-up of 29 months, median PFS was 17.2 months in the standard three-weekly paclitaxel arm compared to 28 months in the weekly paclitaxel arm (HR 0.71; 95% CI 0.58-0.88) and 3 year OS (at 42 months follow-up) was 65.1% and 72.1% respectively (HR 0.75; 95% CI 0.57-0.98). These improvements in survival exceed any benefit previously seen in, or anticipated from, any phase III trial in ovarian cancer. Updated survival results presented at ASCO 2012 after 6.4 years median follow-up show that these benefits have been sustained with the 5-year overall survival rate of patients treated with weekly paclitaxel being 58.6% compared with 51.0% in the standard arm (HR 0.75; 95% CI 0.62-0.91).[27]

Notably, this improvement was seen despite the fact that only 61.5% of patients in the weekly dosing arm received at least 6 cycles of treatment (compared with 72.7% in the three-weekly arm). The reason for the higher rate of early treatment discontinuation in the weekly arm was treatment-related toxicity (TRT). This was predominantly myelosuppression; 36.2% of patients in the weekly arm and 21.6% of patients in the three-weekly arm stopped early because of TRT (p=0.03). Of note, 48% of subjects in the weekly paclitaxel arm required at least one dose reduction (compared to 35% in the three-weekly arm) and 76% required at least one delay in treatment administration (67% in the three-weekly arm). However, the mean delivered dose intensity of paclitaxel achieved in the weekly arm 63.0mg/m^2/wk (planned 80mg/m^2/wk) was still higher than in the three-weekly arm, where it was 51.7mg/m^2/wk (planned 60mg/m^2/wk).

The findings of this study are highly provocative and there is international enthusiasm to further assess this regimen and determine whether the efficacy results can be extrapolated worldwide. There is, however, an open question with respect to the tolerability of this regimen. Because of this, in ICON8, we plan to evaluate the feasibility of the research regimens at an early stage by performing an interim

analysis after the first 50 patients randomised to each arm could have completed therapy. If either or both regimens fails to meet a pre-defined cut-off for treatment completion and toxicity, the starting dose of paclitaxel will be reduced from 80mg/m^2/wk to 60mg/m^2/wk.

In addition, the combination of dose-fractionated chemotherapy with DPS has not previously been assessed in a clinical trial. An additional feasibility analysis is, therefore, planned specifically in the sub-group of patients undergoing DPS. This interim analysis will take place after the first 50 patients randomised to each arm with a plan to undergo DPS could have completed therapy.

1.3 DOSE-FRACTIONATED CARBOPLATIN & PACLITAXEL IN THE MANAGEMENT OF OVARIAN CANCER

Although the JGOG3016 trial investigated weekly paclitaxel, there are strong arguments for investigating the administration of both carboplatin and paclitaxel weekly in a randomised phase III study. The dose-fractionated combination is active and many small phase II studies with variable regimens of weekly carboplatin and paclitaxel have yielded promising results. The regimens appear to be deliverable; combined weekly carboplatin and paclitaxel is widely used in Europe as first-line treatment for women who are too frail to receive full dose three-weekly chemotherapy. The fractionation of carboplatin may also improve the toxicity profile of weekly chemotherapy enabling both drugs to be delivered with fewer dose reductions and delays.

1.3.1 EVIDENCE IN RECURRENT OVARIAN CANCER

Weekly combination regimens are highly active, even in women with platinum- resistant/refractory disease, and a number of different doses and schedules have been tested in phase II studies. Delivery of carboplatin AUC4 and paclitaxel 90mg/m^2 on d1 and 8 of a 3 week cycle has been achieved safely in recurrent ovarian cancer with a response rate of 66%. 73% of patients received 6 cycles despite the fact that the majority of patients had received at least two previous lines of chemotherapy, although the incidence of grade ≥3 neutropenia was 94%.[28] Response rates of 60% (RECIST), 76% (CA125) and a median PFS of 8 months were seen in a series of 20 women with platinum-resistant/refractory ovarian cancer treated with weekly carboplatin AUC3 and paclitaxel 70mg/m^2 given on d 1, 8 and 15 of a 4 week cycle. Toxicity was manageable with 34% incidence of grade ≥3 neutropenia and 19% incidence of grade ≥2 peripheral neuropathy.[29] Studies utilising a lower dose regimen, carboplatin AUC2 and paclitaxel 60mg/m^2 have also shown acceptable toxicity although outcomes appear somewhat inferior in patients with platinum-resistant disease.[30] Continuous weekly therapy with carboplatin AUC2 and paclitaxel 80mg/m^2 has also been studied in a series of 27 patients with platinum-sensitive recurrent ovarian cancer and demonstrated a better therapeutic index and toxicity profile when compared with monthly platinum-containing chemotherapy regimens.[31] Other studies have investigated carboplatin AUC2 and paclitaxel 80mg/m^2 given weekly for 3 out of 4 weeks and demonstrated good response rates (50-70%) with manageable toxicity.[32, 33]

1.3.2 EVIDENCE IN FIRST-LINE TREATMENT

Weekly carboplatin and paclitaxel regimens have also been tested in the first-line setting. A phase II study of 64 patients with stage IC-IV ovarian cancer utilised carboplatin AUC2 and paclitaxel 80mg/m^2

given weekly for 3 out of 4 weeks and demonstrated encouraging activity and tolerability. Median PFS was 25.7 months, 25% of patients suffered grade ≥3 neutropenia with only one febrile episode. The incidence of grade ≥2 neuropathy was 17%.[34] In an elderly population (MITO-5), carboplatin AUC2 and paclitaxel 60mg/m^2 given 3 out of 4 weeks was shown to have a favourable toxicity profile although response rate was only 38% and PFS 13.1 months.[35] When compared (in a non-randomised study) to a monthly carboplatin and paclitaxel regimen, the weekly regimen (carboplatin AUC2 and paclitaxel 60mg/m^2 given weekly without a break) demonstrated fewer treatment delays, improved tolerability and cost-effectiveness and comparable efficacy.[36]

Higher dose weekly regimens also appear tolerable. Carboplatin AUC2 and paclitaxel 100mg/m^2 (given in 3 blocks of 6 out of 8 weeks) was administered to 129 women with stage IIB-IV ovarian cancer with a median of 12 doses received and a median PFS of 21 months. However, significant rates of anaemia and neuropathy were noted.[37]

The use of a 6 week induction protocol with weekly platinum/paclitaxel (cisplatin 70mg/m2 or carboplatin AUC4 and paclitaxel 90mg/m^2) followed by 6 conventional three-weekly cycles (cisplatin 75mg/m^2 or carboplatin AUC6 and paclitaxel 175mg/m^2) has been compared to 8 conventional three-weekly cycles in a first-line phase III randomised trial that recruited 270 patients with stage II-IV ovarian cancer.[38] While there was no significant improvement in PFS and OS, the weekly induction phase was well tolerated with no increase in toxicity compared to three-weekly treatment. The short duration of the weekly phase which would abrogate any anti-angiogenic effect and the small size of this study may have contributed to the failure to detect benefit.

Notably, the incidence of platinum hypersensitivity does not appear to be increased by weekly fractionation. Sehouli et al (2008) did not report any allergic reactions in 129 patients who received 14.3 +/- 4.3 cycles of weekly carboplatin/paclitaxel while Pignata et al (2008) only reported a single grade 1 hypersensitivity reaction in 26 patients receiving a mean of 14.2 doses. Although Safra et al (2009) noted 20 grade 1 allergic reactions in 64 patients, these all responded to simple measures and did not complicate further carboplatin administration.

While it is difficult to draw direct comparisons between these multiple phase II studies, it is clear that weekly regimens containing doses of paclitaxel up to 100mg/m^2 in combination with carboplatin at doses greater than or equal to AUC2 are deliverable. Encouraging activity has been shown for carboplatin AUC2 and paclitaxel 80mg/m^2 using both a continuous weekly schedule and a schedule administering treatment for 3 weeks out of 4 in both the first-line and recurrent setting. For comparability with the other arms of the study, we propose to use a continuous regimen in ICON8.

It should, however, be noted that weekly carboplatin-paclitaxel has never been examined in the phase III setting in ovarian cancer although weekly carboplatin AUC2 and paclitaxel 100mg/m^2 was of equivalent efficacy to three-weekly treatment in an 883 patient randomized controlled trial in stage IIB-IV non-small cell lung cancer.[39] An interim analysis assessing 9-month PFS rate (as a measure of activity) will, therefore, be conducted in the first 62 patients randomised to each arm with the aim of excluding markedly reduced activity in either of the two experimental arms. This assessment of activity is also important for the weekly paclitaxel arm as early confirmation of the findings of the JGOG study in a different study population.

1.4 INTEGRATING BEVACIZUMAB WITH DOSE-FRACTIONATED PACLITAXEL AND NEOADJUVANT CHEMOTHERAPY IN THE FIRST-LINE MANAGEMENT OF OVARIAN CANCER

Given that the incorporation of bevacizumab and weekly dose-dense paclitaxel into first-line management of ovarian cancer has improved survival in phase III clinical trials, both of these approaches can be considered new standards-of-care. They do however have markedly different economic implications for healthcare providers and place distinct burdens on patients with respect to treatment-related toxicity and duration/intensity of therapy. There is therefore an urgent need to compare these treatment approaches in a randomised trial. This trial should also evaluate a combined bevacizumab-dose dense paclitaxel treatment strategy as clinical efficacy data suggest the benefits from these two approaches may be additive in recurrent disease.[55] These are the goals of the ICON8B comparison.

The published results of the GOG262 trial give us some limited insight into these questions. In this trial 692 women with stage II-IV ovarian cancer were randomised to 3-weekly carboplatin-paclitaxel or 3-weekly carboplatin and dose-dense paclitaxel. Bevacizumab at a dose of 15mg/kg administered once every 3 weeks and continued until disease progression was allowed at physician's discretion and was given to 84% of trial participants. PFS was equivalent in both arms (14.0 vs 14.7 months; HR 0.89 95%CI 0.74-1.06). Notably, in the 16% of patients who did not receive bevacizumab, an improvement in PFS in favour of weekly dose-dense paclitaxel was seen (10.3 vs 14.2 months; HR 0.62 95% CI 0.40-0.95).[56] The use of bevacizumab in a non-randomised fashion in this trial makes interpretation of these data difficult. However, the results suggest that while the combination of bevacizumab and dose-dense paclitaxel might not substantially improve PFS, weekly dose-dense paclitaxel alone could be as efficacious a strategy as adding bevacizumab to 3-weekly chemotherapy.

The GOG262 trial is also weakened by the fact that inclusion criteria allowed some patients with lower risk disease to enter the trial and only 13% of participants underwent neoadjuvant chemotherapy. It is potentially this patient group that would have most to gain from the addition of bevacizumab to dose-dense chemotherapy.

One of the key determinants of prognosis in advanced ovarian cancer is the achievement of complete macroscopic debulking (CMD) at primary surgery. This has been shown in both the IPS[64] and DPS settings.[4] A systemic therapy that increases CMD rates when used in the neoadjuvant setting may therefore have a positive impact on patient survival. RECIST response (RR) may be a marker of the likelihood of achieving CMD and notably, the addition of bevacizumab to 3-weekly carboplatin-paclitaxel in ICON7 increased RR in patients with measurable disease (n=520) from 48 to 67% (19% increase; 95% CI 11-28%)[9] and in the OCTAVIA trial- a single arm phase II evaluation of the combination of bevacizumab and weekly dose-dense paclitaxel[57],a RR of 85% was reported. Of note, 2 phase III RCTs have also shown significant increases in pathological complete response in triple- negative breast cancer associated with the addition of bevacizumab to neoadjuvant chemotherapy.[58 59]

A key concern that needs to be addressed in ICON8B is the potential for increased surgical morbidity associated with the use of pre-operative anti-angiogenic therapy, in particular haemorrhage, bowel anastomotic complications, delayed wound healing and post-operative thrombo-embolic events. This is particularly relevant given the long half-life for bevacizumab clearance. Although data in ovarian cancer are limited, two recent publications- one a small case series[60] and the second a phase Ib trial[61] conclude that the approach is safe. A French phase II efficacy evaluation of neoadjuvant bevacizumab, the ANTHALYA trial that has completed recruitment of over 90 women with no adverse safety signal detected at DMC review.[62 63]

The safety of this approach has also been demonstrated in other disease settings, 2 post-marketing prospective observational cohort studies evaluating the use of bevacizumab in metastatic colorectal cancer did not document higher than anticipated rates of surgical complications in 546 patients

undergoing surgery during bevacizumab therapy although wound-healing complication rates were higher in those operated on <2 weeks after last bevacizumab dose (9.7% vs 4.4%).[65 66] Retrospective case series also show no increase in complications in patients undergoing resection of colorectal cancer hepatic metastases after bevacizumab therapy[67] and the recently published phase II analysis (n=200) of the ST03 trial which randomised patients with locally-advanced gastro-oesophageal cancer to 3 cycles of neoadjuvant chemotherapy with or without bevacizumab did not show a negative impact on surgical outcomes including wound healing, anastomotic leaks and gastrointestinal bleeding associated with neoadjuvant bevacizumab.[68]

In order to rigorously address this issue an early interim safety analysis of ICON8B will be conducted after the first 50 patients receiving neoadjuvant chemotherapy in each arm have undergone surgery and the trial will exclude patients with extensive bowel involvement who may be predicted to have higher rates of gastrointestinal complications, in paticular perforation.

1.5 RATIONALE FOR THE SUSPENSION OF ICON8B ARM B2 AND CONTINUATION OF THE 2-ARM (B1 VS B3) COMPARISON

Data from the PFS analysis of the ICON8 trial matured in 2017 and the main analysis was presented on 20th April 2017 to the ICON8 Independent Data Monitoring Committee (IDMC). Importantly, all three arms of the trial demonstrated PFS that was fully consistent with the results of recent similar international studies, for patients in both immediate primary surgery (IPS) and delayed primary surgery (DPS) cohorts. However, the superiority of dose-dense (weekly) chemotherapy could not be confirmed: neither of the experimental arms was statistically superior to standard 3-weekly carboplatin and paclitaxel, the control arm. Arm B2 in ICON8B is identical to arm 2 in ICON8 (3-weekly carboplatin and dose dense weekly paclitaxel), and is associated with more intensive patient visits.

The final survival analysis from the ICON7 trial demonstrated a clinically significant survival advantage for bevacizumab + 3-weekly carboplatin-paclitaxel chemotherapy compared to 3-weekly carpoplatin-paclitaxel chemotherapy alone in the group of patients with high-risk stage III and IV ovarian cancer who are eligible for ICON8B. It is therefore possible that arm B2, which was not more efficacious than 3-weekly carboplatin-paclitaxel chemotherapy in ICON8 could be inferior to the bevaiczumab-containing treatment arms B1 and B3 in ICON8B.. Hence the TMG in collaboration with input from the IDMC, Trial Steering Committee (TSC), and trial funder made the decision to suspend arm B2 to recruitment and continue ICON8B as a 2-arm randomised study comparing arm B1 and arm B3. This change was implemented on 5th May 2017.

The TMG and principal investigators feel strongly that exploring the integration of weekly dose-dense paclitaxel and bevacizumab, particularly in the neoadjuvant setting is still and important and relevant research question as;

1- In platinum-resistant EOC, the addition of bevacizumab to weekly paclitaxel was associated with a greater impact on clinical outcomes, including survival than when pegylated liposomal doxorubicin or topotecan were used as ‘partner’ chemotherapy in the phase III AURELIA trial (Poveda et al 2015)
2- A RECIST response rate of 85% was documented in 91 patients with advanced ovarian cancer and measurable residual disease after primary surgery who were treated with bevacizumab- carboplatin-weekly paclitaxel (equivalent to Arm B3) in the OCTAVIA phase

II trial. A response rate of 67% was seen in a similarly-defined patient population treated with bevacziumab and 3-weekly chemotherapy in the ICON7 trial.

3- Mature outcome data from the ANTHALYA and GEICO-NOVA studies, two randomised phase II trials comparing bevacizumab-carboplatin-paclitaxel to chemotherapy alone prior to cytoreductive surgery in advanced EOC indicate that the use of neoadjuvant bevacziumab is safe and associated with higher operability rates compared to chemotherapy alone (Rouzier et al 2017; Garcia et al 2017).

4- The rigorous longitudinal collection of ovarian cancer tissue and blood sampling for DNA and plasma incorporated into ICON8B offers a unique opportunity to understand the biology of ovarian cancer in the context of combined anti-antiangiogenic and cytotoxic chemotherapy.

1.6 INCORPORATION OF FIGO 2013 STAGING SYSTEM INTO ICON8 AND ICON8B

Since the launch of the ICON8 trial, an updated staging system for ovarian cancer has been adopted by FIGO.[69] This is delineated in **Appendix 3**. Key changes are;

1) Subdivision of stage Ic dependent on the timing of ovarian capsular rupture and the presence of malignant cells in ascites
2) Incorporation of isolated retroperitoneal lymph nodal metastatic disease into stage IIIA given the better prognosis of this pattern of spread
3) Subdivision of stage IV into IVA (isolated malignant pleural effusion) and IVB (parenchymal or extrabdominal metstatic disease)

As recruitment to ICON8 was more than 50% complete at the time of publication of the revised staging, a decision was made to continue to utilise the 1988 staging for all patients entering the ICON8 trial pathway. However, for patients entering ICON8B, the new staging system will be adopted. This distinction is reflected in the eligibility criteria for the two studies and investigators are referred to the staging **Appendix 2** for clarification.

2. SELECTION OF SITES/CLINICIANS

The trial sponsor has overall responsibility for site and investigator selection.

In order to participate in the ICON8 Trials Programme, investigators and sites must be affiliated with a participating GCIG group and must fulfil a set of basic criteria which have been agreed by participating groups. Each site must complete a site accreditation form (separate for ICON8 and ICON8B) which verifies that the site is willing, and able, to comply with the requirements of the trial. This will be signed by the Principal Investigator (PI) for that site on behalf of all staff who will be working on the ICON8 trial.

2.1 GCIG GROUP APPROVAL

Prior to group approval a contract must be signed between MRC and the participating GCIG group (or their legal entity). In addition, each participating group is required to send the following documents to the MRC CTU before they can activate their sites and start to randomise patients into the trial:

1. Confirmation of regulatory approval

2. Confirmation of national or local ethics approval of protocol, Patient Information Sheet and Informed Consent Form

3. Copy of the nationally approved Patient Information Sheet

4. Certificate of trial insurance or equivalent if applicable to national groups

5. Copy of any group specific operating instructions.

2.2 SITE/INVESTIGATOR INCLUSION CRITERIA

To participate in the ICON8 trials programme, investigators and clinical trial sites must fulfil a set of basic criteria that have been agreed by the ICON8 Trial Management Group (TMG) and are defined below.

Sites where a previous serious protocol breach has occurred will be visited and thoroughly reviewed before allowing participants to enter the trial.

Criteria for site accreditation are outlined below in **sections 2.2.1 and 2.2.2.**

2.2.1 PI's Qualifications & Agreements

1. The investigator has appropriate experience of conducting trials according to the principles of Good Clinical Practice (GCP) and should be qualified by education, training, and experience to assume responsibility for the proper conduct of the trial at their site. The investigator should provide evidence of such qualifications through an up-to-date curriculum vitae and/or other relevant documentation requested by the Sponsor, the REC, the IRB, and/or the regulatory authority(ies)

2. The investigator and clinical trial site staff should be thoroughly familiar with the appropriate use of the investigational products, as described in the protocol, in the current Investigator

Brochure, in the product information and in other information sources provided by the Sponsor

3. The investigator should be aware of, and should comply with, the principles of ICH GCP and the applicable regulatory requirements. A record of GCP training should be accessible for all investigators

4. The clinical trial site permits monitoring by the MRC Clinical Trials Unit (MRC CTU) and/or qualified third-parties employed by MRC CTU for monitoring purposes. The clinical trial site also permits inspection by the appropriate regulatory authorities. Direct access to all trial related sites, documents, reports and data must be available

5. The investigator should maintain a delegation log of appropriately-qualified persons to whom the investigator has delegated significant trial-related duties

6. The investigator should be able to demonstrate a potential for recruiting the required number of suitable subjects within the agreed recruitment period (that is, the investigator regularly treats the target population)

7. The investigator should have sufficient time to properly conduct and complete the trial within the agreed trial period

8. The investigator should sign an investigator statement (separate statements are required for ICON8 and ICON8B), which verifies that the site is willing and able to comply with the requirements of the trial.

2.2.2 ADEQUATE RESOURCES

1. The clinical trial site regularly undertakes the treatment of ovarian cancer

2. Clinical trial site staff are familiar with the use of the experimental regimens, as described in the protocol and in the relevant Summary of Product Characteristics (SPCs)

3. The clinical trial site has an adequate number of qualified staff and adequate facilities, for the foreseen duration of the trial, to conduct the trial properly and safely

4. The site will have adequate cover from other senior colleagues at time of PI absences to maintain best practice whilst managing trial patients. Delegation of responsibilities to junior staff should only be done after assurance of familiarity with trial protocol and GCP

5. The investigator should have available an adequate number of qualified staff and adequate facilities for the foreseen duration of the trial to conduct the trial properly and safely

6. The investigator should ensure that all persons assisting with the trial are adequately informed about the protocol, the investigational product(s), and their trial-related duties and functions

7. The site should have sufficient data management resources to allow prompt data return to the MRC CTU (refer to the Data Management Plan for timelines). Sites that have previously participated in MRC CTU-coordinated trials should have a proven track record of good data return

8. All trial data must be submitted in a timely manner, and as described in the protocol. Individual clinical trial sites may be suspended on the recommendation of the Trial Management Group (TMG) if data return does not comply with the ICON8 or ICON8B data return policy or if trial conduct is violated in other ways

9. The clinical trial site has experienced surgeons accredited in gynae-oncology and who specialise in the management of patients with gynaecological malignancies

10. The clinical trial site has appropriate pathologists who specialise in the reporting of gynae-oncology specimens

11. The clinical trial site has trained oncologists to deliver chemotherapy, who specialise in the treatment of ovarian cancer, and who are integrated into the gynae-oncology multi-disciplinary team and experienced in the care of patients receiving carboplatin and paclitaxel

12. Chemotherapy prescribing conforms to best local practice, including computerised prescribing, where available

13. The site will have appropriate radiologists who have the capability and capacity to report all trial protocol scans using RECIST v1.1

14. All staff assisting with the trial are to be adequately informed about the protocol and their trial related duties

15. The study must be conducted in accordance with the current protocol and changes will only be made when necessary to protect the safety, rights and welfare of patients

16. Formal protocols for the management of acute medical or acute surgical complications of treatment are to be in place at the clinical trial site

17. The clinical trial site runs a 24 hour specialised oncology on call service (an off-site referral service able to provide advice over the telephone is acceptable as a minimum standard) to which patients experiencing treatment toxicities will be referred

18. The trial is conducted in compliance with the principles of GCP and applicable regulatory requirements

19. The clinical trial site must maintain an Investigator Site File, which will contain documents essential for the conduct of the trial

20. A record of the method used by the clinical trial site to analyse serum creatinine is included in the site's Investigator Site File, and updated if laboratory methods change

21. All Serious Adverse Events (SAE) must be reported immediately to the MRC CTU, except for those that the protocol identifies as not requiring immediate reporting

22. Initial SAE reports must be promptly followed by detailed written reports as appropriate

23. No trial data can be disclosed without the approval of the Trial Steering Committee (TSC)

24. All trial related documents must be retained for 25 years after the completion of the trial.

2.3 SITE APPROVAL

Each selected clinical trial site must complete the ICON8 and/or ICON8B Accreditation Form (depending on which pathways the site are to participate in), which includes the Investigator Statement, Signature and Delegation of Responsibilities Log, and staff contact details. The Investigator Statement verifies that the site is willing, and able to comply with the requirements of the trial. This will be signed by the Principal Investigator at the site. In addition and in compliance with the principles of ICH GCP, all site staff participating in the trial must complete the Signature and Delegation of Responsibilities Log and forward this to the MRC CTU. The MRC CTU must be notified of any changes to trial personnel and/or their responsibilities. An up-to-date copy of this log must be stored in the Site File at the site and also at the MRC CTU.

Before a site can be approved to start randomising patients the following documents should be forwarded to the national GCIG participating group coordinating the trial:

1. Site accreditation form/Investigator statement and checklist (based on the criteria in section 2.1: GCIG Group Approval)

2. CVs and evidence of GCP training for Principal and Co-Investigators

3. Local ethics approval (if required) of Protocol, Patient Information Sheet and Informed Consent Form

4. Delegation of responsibility log

5. Evidence of completion of initiation training

6. Normal ranges and procedures on methods used for analysing creatinine

7. Local Investigator Site File Assessment form (UK)

8. Pharmacy site-file Assessment form (UK)

9. Sample labels for carboplatin, paclitaxel, bevacizumab infusions

10. Sample prescriptions for each arm of the trial

When these documents have been received by the national GCIG participating group, the GCIG group will complete a provisional site approval form and fax this to the MRC CTU. The GCIG group will activate the site and inform them that initiation has been completed.

Each person working on the ICON8 trial must complete a section of the delegation of responsibility log, indicating their responsibilities and giving contact details (phone, fax and email address). Sites must notify their GCIG group of any subsequent changes to trial personnel and/or their responsibilities. The GCIG participating group will update the MRC CTU. An up-to-date copy of the log must be stored in the Investigator Site File, at the GCIG participating group and at the MRC CTU. In the UK the Clinical Trial Authorisation (CTA) for the trial requires that the Medicines and Healthcare Products Regulatory Agency (MHRA) be supplied with the names and addresses of all participating site

principal investigators. Trial staff at the MRC CTU will perform this task; hence it is vital to receive full contact details for all investigators prior to their entering patients.

Site initiation training will be provided by the national GCIG participating group via investigator meetings, teleconferences and/or review of training slides.

A list of activated sites may be obtained from the Trial Manager.

SECTION A: ICON8

This section includes all information on the selection, treatment and follow-up of patients entering the original ICON8 randomisation.

Please note that randomisation into the original ICON8 arms is closed as of November 2014.

This section should not be used to guide the selection, treatment or follow-up of any patients entering the ICON8B randomisation. For those patients, please refer to Section B.

A1 SELECTION OF PATIENTS

There will be no exceptions to eligibility requirements at the time of randomisation. Questions about eligibility criteria should be addressed prior to attempting to randomise the participant.

The eligibility criteria for this trial have been carefully considered. The eligibility criteria are the standards used to ensure that only medically appropriate patients are considered for this study. Patients not meeting the criteria should not join the study. For the safety of the patients, as well as to ensure that the results of this study can be useful for making treatment decisions regarding other patients with similar diseases, it is important that no exceptions be made to these criteria for admission to the study.

ICON8 will include patients with newly diagnosed, histologically confirmed, FIGO (1988) stage IC/IIA (high-risk) - IV epithelial ovarian carcinoma, primary peritoneal carcinoma, fallopian tube carcinoma or ovarian carcinosarcoma.

Patients will be considered eligible for enrolment into this trial if they fulfil all of the inclusion criteria and none of the exclusion criteria, as defined below:

A1.1 PATIENT INCLUSION CRITERIA

1. Females aged ≥18 years
2. Signed informed consent and ability to comply with the protocol
3. Histologically confirmed, with core biopsy from a disease site as minimum requirement (cytology alone is insufficient for diagnosis):
   - Epithelial ovarian carcinoma
   - Primary peritoneal carcinoma of Müllerian histological type
   - Fallopian tube carcinoma
   - Ovarian carcinosarcoma (malignant mixed Müllerian tumour (MMMT) of the ovary).
4. FIGO (1988) stage IC or above (see **Appendix 2**)[5], which may be based on clinical and radiological assessment in patients who have not undergone immediate primary surgery
5. Confirmed high-risk histological subtype for patients with FIGO (1988) stage IC/IIA disease, namely:
   - High grade serous carcinoma
   - Clear cell carcinoma
   - Other histological subtype considered poorly differentiated/grade 3
6. ECOG Performance Status (PS) 0-2
7. Life expectancy >12 weeks

[5] See Appendix 2 for FIGO staging classification. Please note that the FIGO 1988 staging classification as given in Appendix 2 must continue to be used to stage patients for entry in ICON8.

8. Adequate bone marrow function:
   - Absolute Neutrophil Count (ANC) ≥1.5 x 10^9/l
   - Platelets (Plt) ≥100 x 10^9/l
   - Haemoglobin (Hb) ≥9g/dl (can be post transfusion)

9. Adequate liver function:
   - Serum bilirubin (BR) ≤1.5 x ULN
   - Serum transaminases ≤3 x ULN in the absence of parenchymal liver metastases or ≤5 x ULN in the presence of parenchymal liver metastases

10. Adequate renal function as defined by:
   - Directly measured GFR (Glomerular Filtration Rate) ≥ 30 ml/min, or
   - Calculated creatinine clearance ≥ 60 ml/min

   NB. If the calculated creatinine clearance is <60 ml/min the GFR should be directly measured using either a 24 hour urine collection or an isotopic evaluation

11. Able to start chemotherapy within 8 weeks after immediate primary surgery (where applicable).

A1.2 PATIENT EXCLUSION CRITERIA

1. Non-epithelial ovarian cancer

2. Peritoneal cancer that is not of Müllerian origin, including mucinous histology

3. Borderline tumours (tumours of low malignant potential)

4. Prior systemic anti-cancer therapy for ovarian cancer (for example chemotherapy, monoclonal antibody therapy, tyrosine kinase inhibitor therapy or hormonal therapy)

5. Previous malignancies within 5 years prior to randomisation apart from:

   - adequately treated carcinoma in-situ of the cervix, breast ductal carcinoma in-situ, non-melanomatous skin cancer; or
   - previous/synchronous early-stage endometrial cancer defined as stage IA (FIGO 2009) grade 1 or 2 endometrioid cancers with no lymphovascular space invasion

6. Pre-existing sensory or motor neuropathy grade ≥2

7. Evidence of any other disease/metabolic dysfunction that in the opinion of the investigator would put the subject at high-risk of treatment-related complications or prevent compliance with the trial protocol

8. Planned intraperitoneal cytotoxic chemotherapy

9. Planned maintenance treatment with systemic anti-cancer therapy following completion of protocol treatment and prior to protocol defined progression

10. Any previous radiotherapy to the abdomen or pelvis

11. Sexually active women of childbearing potential not willing to use adequate contraception (eg. oral contraceptives, intrauterine device or barrier method of contraception in conjunction with spermicidal jelly or surgically sterile) for the study duration and at least six months afterwards

12. Pregnant or lactating women who are currently breastfeeding

13. Treatment with any other investigational agent prior to protocol defined progression

14. Known hypersensitivity to carboplatin, paclitaxel or their excipients (including cremophor)

15. History or clinical suspicion of brain metastases or spinal cord compression. CT/MRI of the brain is mandatory in the case of suspected brain metastases. Spinal MRI is mandatory in the case of suspected spinal cord compression. Patients with brain or meningeal metastases are not eligible.

A1.3 CONCOMITANT MEDICATIONS

Caution should be exercised for patients using any medication that may markedly affect renal function. Such medications may be used with care if deemed essential by the responsible investigator or may be continued if already in use prior to entry in the trial with no effect on renal function.

It is recommended that patients receiving therapeutic anti-coagulation are converted to, or maintained on, a low molecular weight heparin preparation for the duration of their protocol treatment.

A1.4 NUMBER OF PATIENTS

1485 patients (495 in each treatment arm) will be recruited over a period of approximately 36 months.

A1.5 SCREENING PROCEDURES AND PRE-RANDOMISATION INVESTIGATIONS

Written informed consent to enter into the trial and be randomised must be obtained from participants after explanation of the aims, methods, benefits and potential hazards of the trial and BEFORE any trial-specific procedures are performed or any blood is taken for the trial see **Appendix 9.**

It must be made completely and unambiguously clear that the participant is free to refuse to participate in all or any aspect of the trial, at any time and for any reason, without incurring any penalty or affecting their treatment.

Patients can be given as much time as they need to decide whether or not to participate in the trial but a **minimum of 24 hours** must be given from the time the trial information is given to the consent form being signed.

The following baseline information should be obtained **within 28 days prior to randomisation unless otherwise stated**, please see **Figure 4**. Investigations which have been performed as part of routine clinical practice can be used in the screening process. However, non-routine investigations or tests required for screening may only be performed following signature of the informed consent form.

Investigations performed prior to IPS are not acceptable for screening.

1. Demographic data; medical history, including previous and current diseases, and concomitant medications

2. Physical examination including the assessment of height, weight, pulse rate and blood pressure

3. ECOG performance status

4. Blood tests[6]
    - Full Blood Count (FBC)
    - Urea and Electrolytes (U&E): serum creatinine (SCr), urea (BUN), K^+, Na^+, Ca^{2+}
    - Liver Function Tests (LFT): Bilirubin, ALP, AST or ALT, albumin
    - Coagulation: PT or INR or aPTT
    - CA125

5. Assessment of renal function
    - Measurement of GFR using a radio-isotopic method or 24 hour urine collection or
    - Calculation of GFR by the modified Wright, Cockcroft-Gault or Jeliffe formulae.

Assessment of GFR should be performed within 14 days before randomisation. However, a radio-isotopic GFR performed within 28 days is also acceptable provided the patient's serum creatinine has not changed by more than 10% between the test and randomisation. If a calculated GFR is used this should be re-calculated prior to treatment using blood tests obtained within the 7 day period before cycle 1 day 1.

6. Other tests
    - Pregnancy test (if of child bearing potential)[8]
    - Correlative bloods for translational research (if applicable)[7]
    - ECG[8,8]

7. Imaging – see **section A1.6: Initial Tumour Assessment** for details
    - Tumour assessment[8,9]
    - Chest X-ray (if CT chest not performed as part of baseline tumour assessment)[8]

8. Baseline quality of life form[9]

---

[6] Results of tests done as part of routine clinical practice and performed within 28 days prior to randomisation may be used for the screening assessment. Any tests listed which have not been performed as part of routine clinical practice must only be performed after the consent form has been signed.
[7] Trial specific test – to be performed only after informed consent form has been signed.
[8] A pre-surgical ECG is acceptable if it was normal, and there is no history of cardiovascular disease.
[9] In patients who have undergone IPS, the baseline tumour assessment may not be available for screening and can be performed after randomisation (see section A1.6.1). This should be recorded on the screening CRF.

9. Check all inclusion and exclusion criteria.

Signed consent forms must be kept by the investigator and documented in the patient's medical records. A copy of the consent form should be given to the participant or family and a copy should be filed in the patient's medical records. With consent, a letter should be sent to the general practitioner informing him/her of the trial and the participant's involvement in it.

A1.5.1 ADDITIONAL PRE-CHEMOTHERAPY TESTS

Where haematology and biochemistry blood tests used for screening are taken more than 7 days prior to starting treatment, they must be repeated within 7 days before cycle 1 day 1. If a calculated GFR is being used for carboplatin dosing, this should be re-calculated using these blood tests (for instructions on carboplatin dosing on trial see **Section A3.4.2.1**: **Carboplatin Dose**). CA125 should also be repeated if it has not been performed within 7 days prior to starting chemotherapy.

The above is summarised, along with the other assessments required throughout the trial in **Section A5 Assessments and Procedures.**

A1.6 INITIAL TUMOUR ASSESSMENT

A1.6.1 IMMEDIATE PRIMARY SURGERY PATIENTS

All patients should have cross-sectional imaging (preferably by CT scan although MRI is allowed) of the pelvis and abdomen. The post-operative baseline tumour assessment should be performed no less than 4 weeks after surgery and no more than 2 weeks after protocol treatment starts. The only exception to this is if it is clinically necessary to start chemotherapy within 2 weeks after surgery. In this case, the baseline tumour assessment should occur 4 weeks (+/- 7 days) after surgery[11].

All subsequent follow-up scans should be the same modality (CT or MRI) and performed using the same technique. Patients will be classified as having measurable or non-measurable disease (as per RECISTv1.1) and target and non-target lesions identified if relevant (see **Appendix 8**).

Imaging of the chest must be performed to check for thoracic metastases in all patients prior to randomisation. This can be by CT or Chest X-ray, but if there is any clinical or radiological suspicion of parenchymal lung metastases or mediastinal metastases on the X-ray then a CT or MRI must be performed.

The information above is summarised, with details of subsequent tumour assessments required throughout the trial, in **Section A6 TRIAL ASSESSMENTS SCHEDULES ICON8, Tables 9, 10 and 11**.

A1.6.2 DELAYED PRIMARY SURGERY PATIENTS

All patients should have cross-sectional imaging (preferably by CT scan although MRI is allowed) of the pelvis and abdomen. The initial baseline scan must be done prior to randomisation. It should be performed within the 4 week period prior to randomisation. A longer window may be permitted at the discretion of the Chief Investigator/MRC CTU providing the interval between the scan date and cycle 1 day 1 will be less than 6 weeks. These cases should be discussed with the MRC CTU in the first instance who will liaise with the Chief Investigator where necessary.

All subsequent follow-up scans should be the same modality (CT or MRI) and performed using the same technique. Patients will be classified as having measurable or non-measurable disease (as per RECISTv1.1) and target and non-target lesions identified if relevant (see **Appendix 7**).

Imaging of the chest must be performed to check for thoracic metastases in all patients prior to randomisation. This can be by CT or Chest X-ray, but if there is any clinical or radiological suspicion of parenchymal lung metastases or mediastinal metastases on the X-ray then a CT or MRI must be performed.

The information above is summarised, with details of subsequent tumour assessments required throughout the trial, in **Section A6 TRIAL ASSESSMENTS SCHEDULES ICON8, Tables 12, 13 and 14**.

Figure 5: Timelines for Screening and Pre-treatment Investigations

Key

Abbreviation	Expansion
R	Randomisation

A. Patients entering the trial following IPS

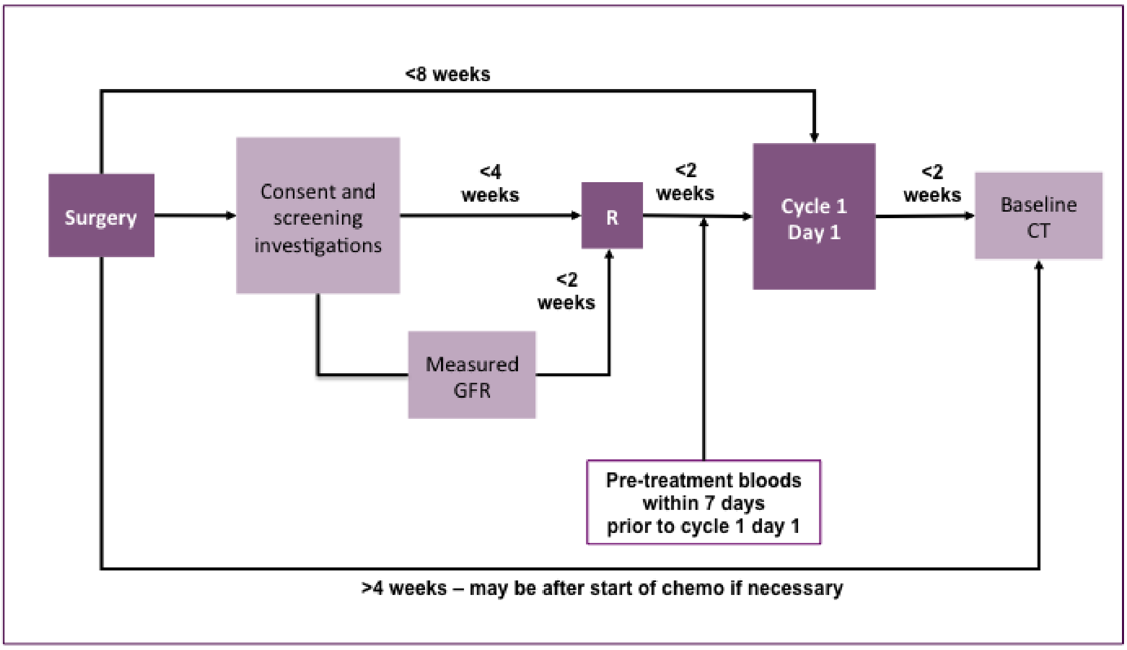

B. Patients entering the trial with a plan for DPS or primary chemotherapy alone

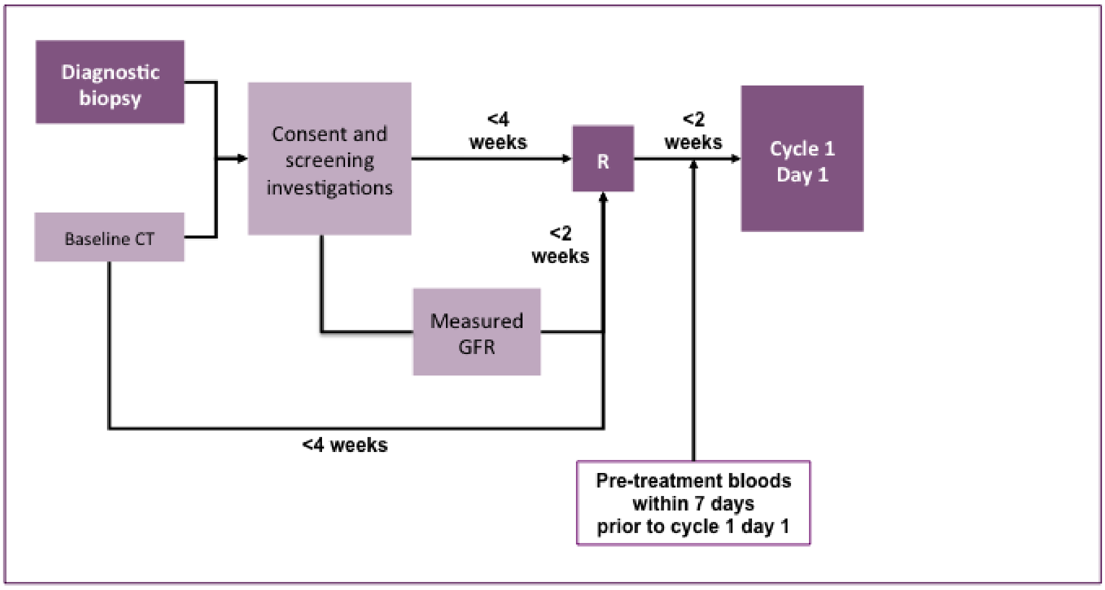

NB. Investigations performed as part of routine clinical practice prior to consent may be used for screening purposes but any trial-specific screening procedures should only be performed following consent.

A2 REGISTRATION AND RANDOMISATION

Patients will be randomly assigned in a 1:1:1 ratio to receive chemotherapy on a three-weekly cycle comprising:

- **Arm 1 (control arm):**
  - Carboplatin AUC5 and paclitaxel 175mg/m^2 both given on day 1
- **Arm 2 (dose-fractionated paclitaxel):**
  - Carboplatin AUC5 given on day 1 and paclitaxel 80mg/m^2 given on day 1, 8 and 15
- **Arm 3 (dose-fractionated carboplatin and paclitaxel):**
  - Carboplatin AUC2 and paclitaxel 80mg/m^2 given on day 1, 8, and 15.

A2.1 INFORMATION REQUIRED FOR RANDOMISATION

The following data will be required in order to enrol a patient:

1. Confirmation (yes or no) that the patient satisfies all the eligibility criteria
2. Confirmation (yes or no) that none of the exclusion criteria apply
3. Results for all the screening procedures specified in **Section A1.5: Screening Procedures and Pre-Randomisation Investigations**
4. Patient’s date of birth
5. Patient’s initials
6. FIGO (1988) stage
7. Histological subtype and grade
8. For patients who have undergone IPS: surgery date, outcome and if there is any plan to perform further debulking surgery (IDS)
9. For patients who have not undergone IPS: whether DPS is planned
10. In patients who have given specific consent, additional personal details will be collected so that information about health status may be obtained, if needed during follow-up, from the NHS Information Centre, NHS Central Register or other NHS information system. Any identifiable patient information collected will be stored separately from other data and not linked in any way.
11. Confirmation (yes or no) that the patient has consented to translational research
12. For patients who consent to translational research: confirmation of the level of consent.

A2.2 RANDOMISATION PROCEDURE

Patients will be randomised using the MRC CTU telephone randomisation service.

For international GCIG groups who are unable to call during operating hours, randomisation may be conducted via fax or directly by remote access to the randomisation service. The procedure for randomisations will be determined jointly by the MRC CTU and each individual GCIG group as they join the study, and will be detailed in the group-specific operating instructions.

Please note that randomisation into the original ICON8 arms is expected to close by the end of 2014. All sites will be notified but please contact the MRC CTU before approaching patients about randomisation into the ICON8 pathway.

RANDOMISATIONS
To randomise, call MRC CTU, Monday to Friday 09:00-17:00 (UK)
Tel: +44 (0) 20 7670 4777

A manual randomisation process will be set up to cover any instances when the main electronic system is not working. This will be detailed in the trial working Instructions.

A2.2.1 Timing of Treatment Following Randomisation

Trial treatment should commence within 2 weeks after randomisation for both IPS and DPS patients. This date should not be affected by the treatment arm the patient is allocated to.

A2.3 CO-ENROLMENT GUIDELINES

Co-enrolment in any therapeutic clinical trial is not allowed prior to protocol defined disease progression including any chemotherapy trial or any other trial of an investigational product for the treatment of ovarian cancer. Co-enrolment in other clinical trials, eg. of interventions in supportive care, may be permitted but should be discussed with the MRC CTU/Chief Investigator prior to entry. These cases should be discussed with the MRC CTU in the first instance who will liaise with the Chief Investigator where necessary.

Figure 6: ICON8 Treatment Schedule

Immediate Primary Surgery

surgery → randomise

Arm 1

Day	1	8	15	1	8	15	1	8	15	1	8	15	1	8	15	1	8	15
Cycle	1			2			3			4			5			6		
	C			C			C			C			C			C		
	T			T			T			T			T			T		

Arm 2

Day	1	8	15	1	8	15	1	8	15	1	8	15	1	8	15	1	8	15
Cycle	1			2			3			4			5			6		
	C			C			C			C			C			C		
	t	t	t	t	t	t	t	t	t	t	t	t	t	t	t	t	t	t

Arm 3

Day	1	8	15	1	8	15	1	8	15	1	8	15	1	8	15	1	8	15
Cycle	1			2			3			4			5			6		
	c	c	c	c	c	c	c	c	c	c	c	c	c	c	c	c	c	c
	t	t	t	t	t	t	t	t	t	t	t	t	t	t	t	t	t	t

NB. Chemotherapy to commence within 8 weeks after surgery and within 2 weeks after randomisation.

Delayed Primary Surgery

randomise → cycles 1–3 → surgery → cycles 4–6

Arm 1

Day	1	8	15	1	8	15	1	8	15	surgery	1	8	15	1	8	15	1	8	15
Cycle	1			2			3				4			5			6		
	C			C			C				C			C			C		
	T			T			T				T			T			T		

Arm 2

Day	1	8	15	1	8	15	1	8	15	surgery	1	8	15	1	8	15	1	8	15
Cycle	1			2			3				4			5			6		
	C			C			C				C			C			C		
	t	t	t	t	t	t	t	t			t	t	t	t	t	t	t	t	t

Arm 3

Day	1	8	15	1	8	15	1	8	15	surgery	1	8	15	1	8	15	1	8	15
Cycle	1			2			3				4			5			6		
	c	c	c	c	c	c	c	c			c	c	c	c	c	c	c	c	c
	t	t	t	t	t	t	t	t			t	t	t	t	t	t	t	t	t

NB. Chemotherapy to commence within 2 weeks after randomisation.
Surgery to occur as close as possible to cycle 3 day 22 and within 10 days after it.
Chemotherapy to recommence as soon as possible after surgery but at least 1 week after it.

C = Carboplatin AUC5	T = Paclitaxel 175mg/m^2
c = Carboplatin AUC2	t = Paclitaxel 80mg/m^2

A3 TREATMENT OF PATIENTS

A3.1 GENERAL PRINCIPLES

The ICON8 cohort of the ICON8 Trials Programme is exploring the concept of weekly dose-dense fractionation of carboplatin-paclitaxel chemotherapy in the first-line management of patients with ovarian cancer.

Patients will be randomised in a 1:1:1 ratio between arm 1: arm 2: arm 3. Treatment should start as soon as possible (and within 2 weeks) after randomisation.

Patients may receive full supportive care according to local practice (see **Section A3.6 – Concomitant Therapy**)

A3.2 TREATMENT ARMS

Further details regarding the dosing and reconstitution of individual drugs are given in in **section A3.4: Specific Drug Information.** Details regarding dose modifications, delays and omissions are given in **section A4: Drug Safety.**

Study treatment arms are:

- **Arm 1**
  6 cycles of therapy administered on day 1 every 3 weeks consisting of:
  Paclitaxel 175mg/m^2 administered over 3 hours
  Carboplatin AUC5[j] administered over 30 minutes to 1 hour

- **Arm 2 (dose-fractionated paclitaxel)**
  6 cycles of therapy each administered over 3 weeks consisting of:
  Paclitaxel 80mg/m^2 administered over 1 hour on days 1, 8 and 15
  Carboplatin AUC5[l] administered over 30 minutes to 1 hour on day 1

- **Arm 3 (dose-fractionated carboplatin and paclitaxel)**
  6 cycles of therapy each administered over 3 weeks consisting of:
  Paclitaxel 80mg/m^2 administered over 1 hour on days 1, 8 and 15
  Carboplatin AUC2[k] administered over 30 minutes on days 1, 8 and 15.

The treatment schedule, incorporating timing of surgery, is shown in **Figure 5.**

A3.3 SURGERY

A3.3.1 IMMEDIATE PRIMARY SURGERY (IPS)

Patients who have undergone IPS must commence chemotherapy within 8 weeks of their operation. It is expected that surgery will have been performed in accordance with internationally accepted standards (see **Appendix 6**).

[j] For 3-weekly administration, carboplatin dose is AUC5 if a measured GFR or a GFR calculated by the Wright method is used. If a GFR calculated by the Cockcroft-Gault or Jelliffe formulae is used, the carboplatin dose is AUC6. (see 8.4.3 and Appendix 5)

[k] For weekly administration, carboplatin dose is AUC2 irrespective of the method used to obtain the GFR.

A3.3.1.1 INTERVAL DEBULKING SURGERY (IDS)

Patients in whom IPS was attempted but optimal cytoreduction was not achieved may undergo a second attempt at debulking surgery after 3 cycles of chemotherapy (interval debulking surgery, IDS) at the local investigator's discretion. Intent to perform second debulking surgery should be declared at the time of patient enrolment into ICON8 and the treatment schedule for DPS patients should be followed.

A3.3.2 DELAYED PRIMARY SURGERY (DPS)

In patients entered into ICON8 who have not undergone IPS there should usually be intent to perform DPS after 3 cycles of chemotherapy. However, patients with stage III and IV disease that is considered inoperable and unlikely to become so or patients who choose not to have surgery are also eligible. Surgery should be performed in accordance with internationally accepted standards with the aim of achieving optimal debulking (see **Appendix 6**).

It is acknowledged that, depending on the results of tumour assessments following 3 cycles of chemotherapy, surgery may not be appropriate in a small proportion of patients. In the event of early disease progression, the patient should be withdrawn from protocol treatment (but trial follow-up should continue – see **Section A3.9: Follow-up**). However, if the patient is considered to be benefitting from protocol treatment and does not meet the criteria for protocol defined progression (see **Appendix 8**) then they can continue to be treated within the ICON8 protocol without undergoing surgery.

It is accepted that in exceptional circumstances, patients who are not deemed suitable for DPS after 3 cycles may be considered for debulking surgery at completion of chemotherapy. These cases should be discussed with the MRC CTU in the first instance who will liaise with the Chief Investigator where necessary. In these patients, follow-up should conform to the ICON8 trial protocol but an additional radiological assessment of disease will be required 4 weeks (+/- 7days) after their delayed surgery to act as a new baseline assessment for progression-free survival.

A3.3.2.1 TIMING OF DELAYED PRIMARY SURGERY

DPS should be undertaken as close to Cycle 3 day 22 as possible, and within a maximum of 10 days after this provided that haematological recovery has occurred.

Post-operatively, chemotherapy should recommence between 1- 6 weeks after DPS. To maintain treatment intensity, it is recommended that chemotherapy should be restarted as soon as the patient is fit enough, therefore it should ideally recommence within 1-2 weeks following surgery. However, there should be at least 1 week between DPS and post-operative chemotherapy to allow for post-surgical recovery.

Timings around DPS are shown in **Figure 7**.

Any exceptions to these timings must be discussed with the MRC CTU in the first instance who will liaise with the Chief Investigator where necessary.

A3.3.2.2 MODIFICATIONS TO CHEMOTHERAPY FOR PATIENTS UNDERGOING DPS

Myelosuppression from dose-fractionated chemotherapy may compromise the timing of surgery. Therefore, in women randomised to Arms 2 and 3 undergoing DPS cycle 3 day 15 chemotherapy should be omitted prior to surgery to reduce the likelihood of this occurring.

No modifications to cycle 3 are planned for patients in Arm 1.

Figure 7: Timelines Around DPS

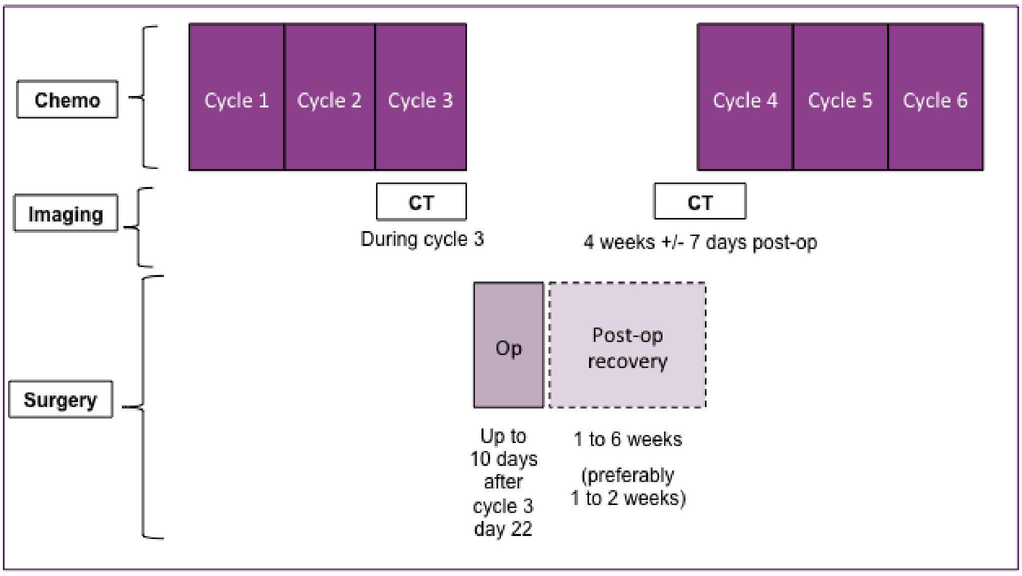

A3.4 SPECIFIC DRUG INFORMATION

Carboplatin and paclitaxel are both considered to be Investigational Medicinal Products (IMP) in ICON8. Each site will use their normally available generic hospital stock of carboplatin and paclitaxel, and their handling and management will be subject to standard local pharmacy procedures. Each trial site pharmacy will be responsible for drug accountability and destruction at their site as detailed in the ICON8 Pharmacy Guidelines document.

Dose banding was not permitted in stage I of the trial, which was evaluating the safety and feasibility of the dose-fractionated chemotherapy regimens, along with the dose intensity of the delivered regimens. Rounding of doses to the nearest 10mg was (and remains) acceptable. The stage I analyses were completed in February 2014 with acceptable safety and feasibility demonstrated. Therefore, dose banding (to within 5% of actual dose) will now be permissible but each site must gain approval from the Chief Investigator/MRC CTU at UCL before instituting dose banding for ICON8 participants. These cases should be discussed with the MRC CTU in the first instance who will liaise with the Chief Investigator where necessary.

A3.4.1 PACLITAXEL[l]

A3.4.1.1 THREE-WEEKLY ADMINISTRATION (ARM 1 ONLY)

PRE-MEDICATION

- Prior to commencing chemotherapy give paclitaxel hypersensitivity prophylaxis, including H1/H2 antagonists and corticosteroids, as per local standards, for example:

    - 30 minutes prior to paclitaxel
        - dexamethasone 20mg IV
        - chlorphenamine 10mg IV (push diluted with 5-10ml Normal Saline)
        - ranitidine 50mg IV (in 20ml Normal Saline over 2 minutes).

- Immediately pre-chemotherapy, give anti-emetics as per local standards; this may include oral or intravenous 5HT3 antagonists

- Anaphylaxis precautions should be available during infusion for the emergency treatment of hypersensitivity reactions

- Reconstitute and administer via a non-PVC giving set and connectors incorporating a filter ≤ 0.22µm

- Reconstitute paclitaxel 175mg/m^2 (cap at BSA 2.0m^2)[m] in 500ml of N Saline or 5% dextrose according to local standard practice

- Give paclitaxel intravenously over three hours via a rate-controlling device

- Monitor closely for allergic reactions and cardiac arrhythmias as per local guidelines

- On extravasation, paclitaxel is a vesicant. Local guidelines for the management of extravasation should be followed.

[l] Paclitaxel should be given prior to carboplatin. The occurrence of a hypersensitivity reaction to paclitaxel is not considered a dose limiting toxicity. Patients may be retreated at full dose after administration of medication to prevent hypersensitivity reactions. If a hypersensitivity reaction does occur then it should be managed according to local protocols, or as suggested in section 9.2.2.4 and the ICON8 trial specific clinical guidelines document.

[m] Body surface area (BSA) to be calculated using a computer algorithm or according to standard nomograms. BSA should only be recalculated if real body weight changes by more than 10%.

A3.4.1.2 Weekly administration (Arm 2 and 3)

Pre-medication

- Prior to commencing chemotherapy give paclitaxel hypersensitivity prophylaxis, including H1/H2 antagonists and corticosteroids, as per local standards, for example:

  - 30 minutes prior to paclitaxel
    - dexamethasone 8mg IV[n]
    - chlorphenamine 10mg IV (push diluted with 5-10ml Normal Saline)
    - ranitidine 50mg IV (in 20ml Normal Saline over 2 minutes)

- Immediately pre-chemotherapy give anti-emetics as per local standards; this may include oral or intravenous 5HT3 antagonists[o]

- Anaphylaxis precautions should be available during infusion for the emergency treatment of hypersensitivity reactions

- Reconstitute and administer via a non-PVC giving set and connectors incorporating a filter ≤ 0.22µm

- Reconstitute paclitaxel 80mg/m^2 (cap at BSA 2.0m^2)[n] in 250ml, or 100ml if dose is less than 75mg, of N Saline or 5% dextrose according to local standard practice

- Give paclitaxel intravenously over one hour via a rate-controlling device

- Monitor closely for allergic reactions and cardiac arrhythmias as per local guidelines

- Use of a cold cap is permitted with both weekly regimens

- On extravasation, paclitaxel is a vesicant. Local guidelines for the management of extravasation should be followed.

---

[n] If patients are unable to tolerate weekly dexamethasone at this dose and have not experienced paclitaxel hypersensitivity, the dose can be gradually reduced at the investigator's discretion. If a hypersensitivity reaction then develops, dexamethasone should be reintroduced at at least 8mg IV prior to ALL subsequent paclitaxel infusions (for further details, please refer to the ICON8 trial specific clinical guidelines document).

[o] For centres who do not have local protocols in place for anti-emetic use with weekly carboplatin-paclitaxel regimens, recommendations will be provided in the ICON8 trial specific clinical guidelines document.

A3.4.2 Carboplatin

- Reconstitute carboplatin to appropriate dose in 5% dextrose or N Saline according to standard local practice

- Give carboplatin intravenously over 30-60 minutes (depending on standard local practice)

- Allergic reactions to carboplatin are not a dose limiting toxicity and should be managed according to standard local practice. Patients may be re-challenged with increased prophylactic medications and/or slowing of infusion rates at the discretion of the treating physician. Recommendations on the management of carboplatin hypersensitivity can be found in **section A4.3.2.4: Hypersensitivity** and the ICON8 trial specific clinical guidelines document

- On extravasation, carboplatin is an irritant. Local guidelines for the management of extravasation should be followed.

A3.4.2.1 Carboplatin Dose

The carboplatin dose should be calculated according to the Calvert formula [40],

Carboplatin dose = Target AUC x (GFR + 25)

The exact dose of carboplatin therefore depends on the GFR and the method of calculating the GFR will also affect the carboplatin dose.

For the purpose of this protocol the GFR is considered equivalent to the creatinine clearance.

It is highly recommended that the GFR is assessed using an isotopic method except in the presence of significant third space fluid collections (ascites/pleural effusion/gross peripheral oedema) which would render this method inaccurate.

If a calculated creatinine clearance is used to calculate the carboplatin dose, then this should be done as per standard local practice using the Cockcroft-Gault, Jelliffe or Wright formulae (see **Appendix 5** for further details). When calculating creatinine clearance **actual body weight** should be used.

When concerns exist about carboplatin dosing in obese or elderly patients an isotopic GFR should be used instead.

If the calculated serum creatinine clearance is <60ml/minute, then a formal measurement of the GFR is mandatory, using either a 24 hour urine collection or an isotopic clearance. If the isotopic clearance is measured then the value uncorrected for body surface area (BSA) should be used in dose calculations.

The target AUC for three-weekly carboplatin (Arms 1 and 2) depends on the method of GFR assessment. Where the carboplatin dose is based on a GFR measured by isotopic clearance, or a 24 hour creatinine clearance (urine collection), the target AUC is 5. If the Wright formula is used to calculate the creatinine clearance the target AUC is also 5, as results obtained using the Wright method have been shown to correspond to directly measured GFR in cancer patients[41, 42]. If the Cockcroft-

Gault or Jelliffe formulae have been used then the target AUC is 6, as results obtained using these formulae are typically lower than a directly measured GFR.

The target AUC for weekly administration (Arm 3) will be 2 irrespective of the method of GFR calculation.

Dose capping of carboplatin may be carried out according to standard local practice, but it is recommended that the maximum three-weekly carboplatin dose (Arms 1 and 2) should not exceed 900mg. If the GFR (either calculated or measured) is >100ml/minute, caution should be exercised with carboplatin dosing and the dose should be confirmed with the trial site's PI, or another clinician with equivalent experience in the use of carboplatin for the treatment of ovarian cancer. Carboplatin dosing is summarised in Table 5.

Table 5: Target AUC Depending on GFR Calculation Method

	**Calculated GFR**	**Calculated GFR**	**Measured GFR (RECOMMENDED)**
	**Cockcroft-Gault and Jelliffe**	**Wright**	**Isotopic/24hr Urine**
**Arms 1 and 2**	AUC6	AUC5	AUC5
**Arm 3**	AUC2	AUC2	AUC2

A3.4.2.2 GFR Limitations

1. Isotopic GFR is inaccurate in patients with significant effusions, ascites or oedema as the isotope distributes into third space fluid collections

2. Patients who have had complicated or prolonged post operative recovery and who have been maintained on prolonged IV fluids with poor nutrition will have a falsely low serum creatinine

3. Formulae, such as the Wright, Cockcroft-Gault and Jelliffe, are inaccurate at the extremes of age and weight. The calculated GFR may be falsely high in obese young women and falsely low in thin elderly women. A measured GFR is particularly recommended in these patients

4. All formulae used to calculate creatinine clearance have limitations. These are further compounded due to different assays used to measure creatinine concentrations in different laboratories. This study is pragmatic with respect to this issue and the GFR/creatinine clearance should be measured/calculated as per standard local practice. If a serum creatinine obtained using an Isotope Dilution Mass Spectrometry (IDMS)-standardised assay has been used to calculate the GFR, a correction factor should not be used when calculating the carboplatin dose. However, investigators should be aware that serum creatinine values obtained using IDMS-standardised assays may be lower than those measured by non-IDMS methods, thus resulting in higher calculated GFR and carboplatin dose. The potential for carboplatin related toxicity may therefore be higher. The method used to measure serum creatinine will be recorded in the Investigator Site File.

It is assumed that clinicians entering patients into this protocol will be aware of these issues and the clinical judgement of an experienced clinician should be applied to the calculation of the carboplatin dose.

A3.4.2.3 REQUIREMENTS FOR RE-ASSESSMENT OF GFR DURING CHEMOTHERAPY

The GFR should be recalculated, or re-measured, for:

- Renal toxicity (serum creatinine >1.5 x ULN, CTCAE v4.0 Grade 2)
- Serum creatinine changes of ≥10% compared to baseline, or last creatinine value used to calculate carboplatin dose (whichever is most recent)*
- Cycle 2, if there has been significant doubt about the true GFR at cycle 1 (according to clinical judgement).

*In Arm 3, patients' serum creatinine is being monitored weekly. If a single change in serum creatinine occurs, investigators may exercise clinical judgment about the need to re-assess the GFR. However, if the increase in creatinine is sustained over more than one week, the GFR should be recalculated or remeasured.

Routine recalculation of the carboplatin dose at the start of each cycle (or, for patients on Arm 3, during a cycle) is not expected unless the above conditions are met.

A3.5 TRIAL TREATMENT RECORDING

Every administration of trial treatment must be recorded in the appropriate part of the CRF. In addition, reasons for any dose delays, dose reductions, dose omissions or permanent discontinuation of study treatment should also be documented.

A3.6 CONCOMITANT THERAPY

All non-cancer treatments that the responsible physician feels are appropriate are allowed.

Any diagnostic, therapeutic or surgical procedure performed during the trial period should be recorded including the date of occurrence, description of the procedure(s) and any clinical findings.

Patients should receive full supportive care during and after the administration of chemotherapy in accordance with local practice. This includes transfusion of blood and blood products and/or the use of erythropoietin and/or G-CSF; antibiotics for infective complications; and anti-emetics. The treatment details should be recorded in the trial CRFs[p]. Anaphylaxis precautions should be observed during administration of carboplatin and paclitaxel as per local practice.

Treatment with concomitant, systemic anti-cancer agents (apart from those specified in the protocol) or other concurrent anti-cancer investigational agents of any type are not allowed in this trial before protocol defined evidence of disease progression. The patient may only be entered into another therapeutic clinical trial of anti-cancer treatment for their ovarian cancer after documented protocol defined disease progression or withdrawal from this trial. (For guidelines on co-enrolment see **section A2.3: Co-enrolment Guidelines**)

---

[p] Anti-emetics routinely administered as part of the chemotherapy regimen do not need to be recorded on the Concomitant Medication CRF.

No patient planned to receive intraperitoneal therapy or maintenance chemotherapy will be eligible for entry into ICON8.

A3.7 TREATMENT FOR PROGRESSION

All anti-cancer treatments including chemotherapy, radiotherapy and surgery for progression will be recorded on trial CRFs during follow-up. Any other additional treatment that the responsible physician feels is appropriate, and is not prohibited by the trial protocol, is permitted.

A3.8 PROTOCOL TREATMENT DISCONTINUATION

In consenting to the trial, patients are consenting to trial treatment, trial follow-up and data collection. However, an individual patient may stop treatment early or be stopped early for any of the following reasons:

- Progression whilst on therapy
- Unacceptable toxicity
- Inter-current illness which prevents further treatment
- Withdrawal of consent for treatment by the patient
- Any alterations in the patient's condition which justifies the discontinuation of treatment in the investigator's opinion.

As the patient's participation in the trial is entirely voluntary, they may choose to discontinue the trial treatment at any time. Although the patient is not required to give a reason for discontinuing their trial treatment, a reasonable effort should be made to establish this reason while fully respecting the patient's rights.

Patients should however remain in the trial, following the same visit schedule, for the purposes of follow-up and data analysis (unless the patient withdraws their consent from all stages of the trial). If a patient is withdrawn from follow-up, refer to **Section A5.7 Early Stopping of Follow-up**.

A3.9 FOLLOW-UP

Once randomised, patients remain evaluable for the intention-to-treat analysis regardless of their subsequent course of treatment. Specific follow-up arrangements are described in **Section A5.1 Visit Schedule**

A4 DRUG SAFETY INFORMATION

A4.1 GENERAL INFORMATION ON PACLITAXEL AND CARBOPLATIN

ICON8 is a trial exploring the weekly dose-dense administration of carboplatin and paclitaxel in the first-line management of patients with ovarian cancer. Drug information on paclitaxel and carboplatin is given in this section. Their toxicities alone and in combination are well established, however, the frequency of these toxicities may be altered by the weekly administration employed in the research arms. In particular, haematological toxicities may be exacerbated. In order to maintain consistency in dose and administration between participating GCIG groups and individual sites, a standard approach to dose modifications, delays and omissions is being applied and the guidance below should be followed for patients who develop adverse events.

A4.1.1 EXPECTED ADVERSE EVENTS WITH PACLITAXEL AND CARBOPLATIN

A list of expected toxicities (based on the current UK SPCs) associated with carboplatin and paclitaxel which will assist the treating physician in the classification of expectedness of serious adverse reactions is given in the investigator site file. The SPCs, or equivalent national pharmaceutical source list, should be referred to for specific guidance.

A4.2 DOSE MODIFICATIONS, DELAYS AND OMISSIONS OF CARBOPLATIN AND PACLITAXEL: GENERAL PRINCIPLES

In order to maintain the dose-intensity and cumulative dose-delivery of carboplatin and paclitaxel chemotherapy, reasonable efforts should be made to minimise dose reduction and treatment delays. Haematological treatment parameters for each arm are shown in **Figure 8**. Early use of G-CSF to maintain dose-intensity, reduce likelihood of dose delays and improve treatment completion is strongly recommended in patients experiencing haematological toxicity (see ICON8 Clinical Management Guidelines document for further details).

Figure 8: Haematological Parameters Required for Administration of Trial Treatment

Arm 1	Arm 2	Arm 3
3-weekly carboplatin-paclitaxel	3-weekly carboplatin + weekly paclitaxel	Weekly carboplatin-paclitaxel
**Day 1:** ANC ≥1.0x10^9/l PLTs ≥75x10^9/l	**Day 1:** ANC ≥1.0x10^9/l PLTs ≥75x10^9/l	**Day 1, 8 & 15:** ANC ≥1.0x10^9/l PLTs ≥75x10^9/l
	**Day 8 & 15:** ANC ≥0.5x10^9/l PLTs ≥50x10^9/l	

- Parameters for entry in the trial, and therefore for administration of Cycle 1 d1, are given in Section 6.1 – Patient Inclusion Criteria.
- Following Cycle 1 d1 there are no trial-specific parameters for haemoglobin & patients should receive appropriate supportive measures for the management of anaemia.
- For patients receiving Arm 1 d1, Arm 2 d1 or Arm 3 d1,8,15 with an ANC between 1.0-1.5x10^9/l use of prophylactic G-CSF to reduce the risk of neutropenic complications is strongly recommended

Patients whose treatment is delayed because of adverse events should be evaluated at weekly intervals (or less) until adequate recovery has occurred. As a general principle, if a patient is unable to receive a trial treatment due to an adverse event:

- If the scheduled treatment was Arm 1 d1, Arm 2 d1 and Arm 3 d1, 8 or 15 (i.e. includes carboplatin), the dose should be delayed and administered when adequate recovery has occurred (with appropriate modification if indicated)
- If the scheduled treatment was Arm 2 d8 or 15 (i.e. weekly paclitaxel alone), the dose should be omitted.

Specific requirements for dose modifications and delays or omissions in the event of particular adverse events are described in **Section A4.3 – Arm 1, Section A4.4 – Arm 2, Section A4.5 – Arm 3.**

Dose modifications for each arm are shown in **Table 6**. If a dose modification is indicated, the dose of carboplatin and/or paclitaxel should be reduced by one dose level. Dose levels may be adjusted independently for each drug.

There are no dose escalations planned (including dose re-escalation after a dose reduction).

Patients who do not tolerate two separate carboplatin and/or paclitaxel dose reductions should discontinue treatment with trial chemotherapy. Any uncertainties about continuation should be discussed with the MRC CTU in the first instance who will liaise with the Chief Investigator where necessary.

If a patient experiences several toxicities and there are conflicting recommendations, please follow the most conservative dose adjustment recommended to minimise doses given.

Table 6: Dose Levels for Paclitaxel and Carboplatin

	**Protocol Starting Dose**	**Protocol Dose Level -1**	**Protocol Dose Level -2**
**3-weekly paclitaxel**<br>*(Arm 1)*	175mg/m^2	135mg/m^2	110mg/m^2
**3-weekly carboplatin**<br>If measured GFR used or GFR calculated by Wright formula<br>*(Arms 1 & 2)*	AUC5	AUC4	AUC3.5
**3-weekly carboplatin**<br>If GFR calculated by Cockcroft-Gault or Jelliffe formulae<br>*(Arms 1 & 2)*	AUC6	AUC5	AUC4.5

**Weekly paclitaxel** *(Arms 2 & 3)*	80mg/m^2	60mg/m^2	45mg/m^2
**Weekly carboplatin** *(Arm 3)*	AUC2	AUC1.67	AUC1.5

A4.3 ARM 1: 3-WEEKLY CARBOPLATIN-PACLITAXEL

A4.3.1 Haematological Toxicity

Day 1 chemotherapy treatment should be delayed if either of the following occurs in the pre-chemotherapy full blood count:

- ANC is less than 1.0 x 10^9/l
- PLT count is less than 75 x 10^9/l .

FBC should then be repeated at least weekly until haematological recovery has occurred (ANC ≥1.0 x 10^9/l and PLT ≥75 x 10^9/l).

If haematological recovery occurs within 7 days, no dose modification is mandated and dosing is left to the discretion of the individual investigator.

If haematological recovery occurs beyond 7 days, it is suggested that doses of carboplatin and paclitaxel are modified according to the day 22 blood count (or subsequent FBC if lower) according to the criteria in **Table 7**. It is recommended that G-CSF prophylaxis is used in preference to dose reduction if this is feasible in order to maintain planned dose intensity.

For patients who receive chemotherapy with an ANC between 1.0-1.5 x 10^9/l, the use of prophylactic G-CSF support to reduce the risk of neutropenic complications and future dose delays is ***strongly*** recommended. If G-CSF is used it can be given in accordance with standard local practice but recommendations for its use are given in the ICON8 trial specific clinical guidance document.

Dose limiting toxicities are defined, and dose modifications mandated, in Table 5. Please note that no dose delay or reduction is expected for anaemia but it should be managed using supportive measures that will maintain dose intensity.

Patients who fail to recover adequate counts after a delay of 2 weeks or more, or who have consecutive dose limiting toxicities, are unlikely to be able to tolerate standard doses of carboplatin and paclitaxel. If it is considered in the patient's best interest to remain within the trial and to continue to receive treatment according to this protocol, then significant modifications of chemotherapy dose or schedule may be required. Such extreme modifications are likely to be rare and should therefore be discussed on a case-by-case basis with the MRC CTU/Chief Investigator.

Any deviation from the proposed dose modification schedule should be discussed with the MRC CTU in the first instance who will liaise with the Chief Investigator where necessary.

Table 7: Guidelines for Paclitaxel and Carboplatin Dose Modification for Delayed Haematological Recovery – Arm 1 & 2 only

	**Delayed ANC recovery (>7 days)**[q]	
**Delayed PLT Recovery (>7 days)**[q]	ANC ≥1.0 x 10^9/l[r]	ANC <1.0 x 10^9/l[r]
PLT ≥75 x 10^9/l[s]	**Paclitaxel**:<br>No modification<br><br>**Carboplatin**:<br>No modification	**Paclitaxel**:<br>Either:<br>Use G-CSF and continue current dose[s]<br>Or<br>Reduce by 1 dose level (**see Table 6**)<br><br>**Carboplatin**:<br>Use G-CSF and continue current dose[t]<br>Or<br>Consider dose reduction by one dose level (**see Table 6**)
PLT <75 x 10^9/l[s]	**Paclitaxel**:<br>No modification<br><br>**Carboplatin**:<br>Reduce by 1 dose level (**see Table 6**)	**Paclitaxel**:<br>Either:<br>Use G-CSF and continue current dose[t]<br>Or<br>Reduce by 1 dose level (**see Table 6**)<br><br>**Carboplatin**:<br>Reduce by 1 dose level (**see Table 6**)

[q] If blood counts recover within 7 days (ie. before day 29) then no dose modification is mandated
[r] Use day 22 ANC/PLT count, or a subsequent FBC if lower, if counts are not recovered by day 29
[s] It is recommended that G-CSF prophylaxis is used in preference to dose reduction if feasible in order to maintain planned dose intensity.

Table 8: Guidelines for Paclitaxel and Carboplatin Dose Modification Following the Occurrence of a Dose Limiting Toxicity (DLT) – all Arms

	**Dose Limiting Toxicity-ANC**[t]	
**Dose Limiting Toxicity-PLT**[u]	**No**	Yes
No	**Paclitaxel:**<br>No modification<br><br>**Carboplatin**:<br>No modification	**Paclitaxel**:<br>Either:<br>Use G-CSF and continue current dose[v]<br>Or<br>Reduce by 1 dose level (**see Table 6**)<br><br>**Carboplatin**:<br>Use G-CSF and continue current dose[v]<br>Or<br>Consider dose reduction by one dose level (**see table 6**)
Yes	**Paclitaxel**:<br>No modification	**Paclitaxel**:<br>Either:<br>Use G-CSF and continue current dose[x]

[t] This is defined by the occurrence of
- febrile neutropenia (defined as ANC <1 x 10^9/l and a single temperature of >38.3°C or a sustained temperature of ≥38°C for more than one hour (CTCAEv4.0)) or
- prolonged grade 4 neutropenia (defined as ANC <0.5x10^9/l persisting ≥7 days)

There are no planned dose modifications for uncomplicated grade 4 neutropenia lasting less than 7 days.

[u] This is defined by the occurrence of
- grade 4 thrombocytopenia (defined as PLT <25 x 10^9/l) or
- bleeding associated with grade 3 thrombocytopenia (PLT 25 – 50 x 10^9/l)

There are no modifications planned for uncomplicated grade 3 thrombocytopenia.

[v] It is recommended that G-CSF prophylaxis is used in preference to dose reduction if feasible in order to maintain planned dose intensity.

	**Carboplatin**:<br><br>Reduce by 1 dose level (**see Table 6**)	Or<br><br>Reduce by 1 dose level (**see Table 6**)<br><br>**Carboplatin**:<br><br>Reduce by 1 dose level (**see Table 6**)

A4.3.2 NON-HAEMATOLOGICAL TOXICITY

A4.3.2.1 RENAL TOXICITY

The combination of carboplatin and paclitaxel, using the schedules described, is not directly expected to cause renal toxicity. There are, therefore, no specific dose modifications for renal toxicity. The GFR used to calculate the carboplatin dose should, however, be recalculated or re-measured in the event of renal toxicity or changes in the serum creatinine – see **Section A3.4.2.3 Requirements for re-assessment of GFR during chemotherapy.**

A4.3.2.2 NEUROPATHY

Grade 2 sensory or motor neuropathy (CTCAEv4.0) requires paclitaxel treatment to be **interrupted** until neuropathy has resolved to grade ≤1. On recovery, paclitaxel should be reintroduced but with the dose reduced by 1 dose level (see **Table 6**). If this requires a delay of more than three weeks then the paclitaxel should be omitted from subsequent cycles and treatment continued with single agent carboplatin at the same AUC used in combination with paclitaxel.

Further recommendations about the management of peripheral neuropathy on trial are given in the ICON8 Clinical Management Guidelines document.

Grade ≥3 sensory or motor neuropathy requires paclitaxel to be **omitted** from subsequent cycles, and treatment continued with single agent carboplatin at the same dose as previously used.

A4.3.2.3 MUCOSITIS

For mucositis grade ≥3 (CTCAEv4.0) chemotherapy should be delayed until the mucositis has resolved to grade ≤1. Paclitaxel can be reduced by one dose level (see **Table 6**) in subsequent cycles at the discretion of the treating physician. If the mucositis recurs, or persists for more than three weeks, at grade ≥3 chemotherapy dose modifications should be discussed with the MRC CTU in the first instance who will liaise with the Chief Investigator where necessary.

Mucositis should be treated symptomatically as per standard local practice.

A4.3.2.4 HYPERSENSITIVITY

A hypersensitivity reaction to either drug is not expected to be a dose limiting toxicity but the occurrence & management of hypersensitivity reactions is being monitored as part of the toxicity analysis. A CRF should be completed on the occurrence of a hypersensitivity reaction. Suggested protocols for the management of carboplatin and paclitaxel hypersensitivity can be found in the ICON8 trial specific clinical guidance document.

Paclitaxel

If a hypersensitivity reaction occurs then patients may be retreated with paclitaxel. This will depend on the severity of the reaction and the specific reaction. Retreatment should be managed according to standard local practice or as suggested in the ICON8 trial specific clinical guidance document. In the case of recurrent hypersensitivity reactions despite adequate premedication, the substitution of docetaxel for paclitaxel is not permitted as there is limited data on the efficacy of this treatment given on a weekly schedule. Treatment should continue with carboplatin alone.

Carboplatin

A hypersensitivity reaction to carboplatin should be managed according to standard local practice. Patients may be retreated according to standard local practice, including escalations of hypersensitivity prophylaxis, in-patient monitoring, increases in the duration of the infusion and use of formal desensitisation protocols (see ICON8 Clinical Management Guidelines for a suggested protocol).

If further hypersensitivity prevents the continued administration of carboplatin, substitution of cisplatin for carboplatin can be considered but due to the lack of evidence for the use of cisplatin with paclitaxel in dose-fractionated regimens, this would be considered off protocol treatment for trial purposes.

A4.3.2.5 LIVER TOXICITY

Hepatotoxicity is not expected with either chemotherapy drug and other causes of liver enzyme elevation should be actively pursued.

If transaminases become elevated and are <3 x ULN (CTCAE v4.0 grade 1) then treatment can be continued as per protocol without any dose modifications or delays. If transaminases become elevated to 3-5 x ULN (CTCAE v4.0 grade 2) then treatment can continue but dose reductions of paclitaxel may be performed according to local practice at the discretion of the treating physician.

If transaminases become elevated to >5 x ULN (CTCAE v4.0 grade 3) then treatment with paclitaxel should cease until resolution to <3 x ULN (CTCAE v4.0 grade 1).

A4.3.2.6 OTHER

There are no dose modifications planned for alopecia, nausea, diarrhoea or constipation. These side effects should be treated with supportive medical measures. Non-steroidal anti-inflammatory agents can be used prophylactically, or symptomatically, as per local practice for the treatment of paclitaxel-induced arthromyalgia.

For any other adverse event of CTCAE v4.0 grade 4 severity considered at least possibly related to study treatment, the patient should be discontinued from protocol therapy after discussion with the MRC CTU in the first instance who will liaise with the Chief Investigator where necessary.

For any other adverse event of CTCAE v4.0 grade 3 severity considered at least possibly related to study treatment, treatment should be withheld until recovery to grade 1 or less and subsequent treatment should be reduced by one dose level (**see Table 6**).

Any patients who develop malignant effusions while on therapy may have them drained according to local practice, assuming coagulation parameters and platelet counts are adequate.

A4.3.3 Stopping Paclitaxel or Carboplatin for toxicity: Alternative regimens

A4.3.3.1 Paclitaxel-specific toxicity

If it becomes necessary to discontinue paclitaxel because of toxic effects (eg. neuropathy or hypersensitivity) then it is recommended that patients continue trial treatment with single agent carboplatin. This can be administered at the same AUC used in combination with paclitaxel or 1 AUC unit higher at the investigator's discretion. This information will continue to be collected on the trial CRFs.

Docetaxel, or any other chemotherapeutic agent, may NOT be substituted for paclitaxel in any situation as there is limited data for the efficacy of other platinum-combination regimens used on a weekly schedule.

Alternative combination chemotherapy regimens used in the standard care of patients with ovarian cancer may be used if the treating investigator feels that it is in the patient's best interests to receive combination therapy rather than single agent carboplatin but this will be considered off-protocol treatment for trial purposes. In this circumstance the patient should remain in the trial following the same visit schedule for the purposes of follow-up and data analysis (see **Section A3.8 Protocol Treatment Discontinuation**). For data completeness, and to avoid potential bias in the reporting of adverse events between arms, data on alternative first-line treatment will be collected on the trial CRFs.

A4.3.3.2 Carboplatin-specific toxicity

If it becomes necessary to discontinue carboplatin because of toxic effects (eg. hypersensitivity) substitution of cisplatin for carboplatin can be considered but due to the lack of evidence for the use of cisplatin with paclitaxel in dose-fractionated regimens, this would be considered off protocol treatment for trial purposes. If cisplatin is used, a recommended schedule would be 75mg/m^2 3-weekly with paclitaxel dosing left at the investigator's discretion. In this circumstance the patient should remain in the trial following the same visit schedule for the purposes of follow-up and data analysis (see **Section A3.8 Protocol Treatment Discontinuation**). For data completeness, and to avoid potential bias in the reporting of adverse events between arms, data on alternative first-line treatment will be collected on the trial CRFs.

A4.4 ARM 2: 3-WEEKLY CARBOPLATIN WITH WEEKLY PACLITAXEL

A4.4.1 HAEMATOLOGICAL TOXICITY

Day 1 chemotherapy treatment should be delayed if either of the following occurs in the pre-chemotherapy full blood count:

- ANC is less than 1.0 x 10^9/l
- PLT count is less than 75 x 10^9/l.

FBC should then be repeated at least weekly until haematological recovery has occurred (ANC ≥1.0 x 10^9/l and PLT ≥75 x 10^9/l).

If haematological recovery occurs within 7 days, no dose modification is mandated and dosing is left to the discretion of the individual investigator.

If haematological recovery occurs beyond 7 days, it is suggested that doses of carboplatin and paclitaxel are modified according to the day 22 blood count (or subsequent FBC if lower) according to the criteria in Table 4. It is recommended that G-CSF prophylaxis is used in preference to dose reduction if this is feasible in order to maintain planned dose intensity.

Day 8 and 15 administration of weekly paclitaxel alone can proceed if:

- ANC is ≥0.5 x 10^9/l
- PLT count is ≥50 x 10^9/l
- There is no complicating fever, infection or bleeding.

If these parameters are not met, the dose should be omitted and the patient reviewed at the time of their next scheduled dose. No dose delays are allowed. Omitted doses will not be replaced.

Patients who require omission of a weekly paclitaxel dose for a low neutrophil count are likely to go on to have omissions in future cycles too. **Following first omission of a weekly paclitaxel dose**, it is therefore recommended that G-CSF is administered with future cycles to reduce the risk of dose delays, maintain dose-intensity and improve treatment completion.

If more than one paclitaxel dose is omitted in the same cycle or at least one dose is omitted from two consecutive cycles, the dose of both carboplatin and paclitaxel should be reduced by one dose level (**see Table 6**).

Dose limiting toxicities are defined, and dose modifications mandated, in **Table 8**. Please note that no dose delay or reduction is expected for anaemia but it should be managed using supportive measures that will maintain dose intensity.

For patients who receive day 1 with an ANC between 1.0-1.5 x 10^9/l, the use of prophylactic G-CSF support to reduce the risk of neutropenic complications and future dose delays is *strongly* recommended.

If G-CSF is used it can be given in accordance with standard local practice but recommendations for its use are given in the ICON8 trial specific clinical guidance document. If it is used, it can be continued at the local investigator's discretion irrespective of the day 8 or 15 ANC (ie. even if it is >1.0 x 10^9/l) but should not be administered in the 48hrs immediately prior to or in the 24 hours following

chemotherapy, ie. no G-CSF should be administered on days 6-9 and days 13-16 of each treatment cycle (for further details refer to the ICON8 trial specific clinical guidance document).

Patients who fail to recover adequate counts after a delay of 2 weeks or more to day 1, or who have consecutive dose limiting toxicities, are unlikely to be able to tolerate standard doses of carboplatin and paclitaxel. If it is considered in the patient's best interest to remain within the trial and to continue to receive treatment according to this protocol, then significant modifications of chemotherapy dose or schedule may be required. Such extreme modifications are likely to be rare and should therefore be discussed on a case-by-case basis with the MRC CTU/Chief Investigator. Any deviation from the proposed dose modification schedule should be discussed with the MRC CTU in the first instance who will liaise with the Chief Investigator where necessary.

A4.4.2 Non-haematological toxicity

A4.4.2.1 Renal toxicity

The combination of carboplatin and paclitaxel, using the schedules described, is not directly expected to cause renal toxicity. There are, therefore, no specific dose modifications for renal toxicity. The GFR used to calculate the carboplatin dose should, however, be recalculated or re-measured in the event of renal toxicity or changes in the serum creatinine – see **Section A3.4.2.3 Requirements for re-assessment of GFR during chemotherapy**.

A4.4.2.2 Neuropathy

Grade 2 sensory or motor neuropathy (CTCAEv4.0) requires paclitaxel treatment to be **interrupted** until neuropathy has resolved to grade ≤1. On recovery, paclitaxel should be reintroduced but with the dose reduced by 1 dose level (**see Table 6**). If this requires a delay of more than three weeks then the paclitaxel should be omitted from subsequent cycles and treatment continued with single agent carboplatin at the same AUC used in combination with paclitaxel.

Further recommendations about the management of peripheral neuropathy on trial are given in the ICON8 Clinical Management Guidelines document. **Grade ≥3 sensory or motor neuropathy** requires paclitaxel to be omitted from subsequent cycles, and treatment continued with single agent carboplatin at the same dose as previously used.

A4.4.2.3 Mucositis

For mucositis grade ≥3 (CTCAEv4.0) chemotherapy should be delayed until the mucositis has resolved to grade ≤1. Paclitaxel can be reduced by one dose level (see Table 5) in subsequent cycles at the discretion of the treating physician. If the mucositis recurs, or persists for more than three weeks, at grade ≥3 chemotherapy dose modifications should be discussed with the MRC CTU in the first instance who will liaise with the Chief Investigator where necessary.

Mucositis should be treated symptomatically as per standard local practice.

A4.4.2.4 Hypersensitivity

A hypersensitivity reaction to either drug is not expected to be a dose limiting toxicity but the occurrence & management of hypersensitivity reactions is being monitored as part of the toxicity analysis. A CRF should be completed on the occurrence of a hypersensitivity reaction. Suggested protocols for the management of carboplatin and paclitaxel hypersensitivity can be found in the ICON8 trial specific clinical guidance document.

Paclitaxel
If a hypersensitivity reaction occurs then patients may be retreated with paclitaxel. This will depend on the severity of the reaction and the specific reaction. Retreatment should be managed according to standard local practice or as suggested in the ICON8 trial specific clinical guidance document.

In the case of recurrent hypersensitivity reactions despite adequate premedication, the substitution of docetaxel for paclitaxel is not permitted as there is limited data on the efficacy of this treatment given on a weekly schedule.

Carboplatin

A hypersensitivity reaction to carboplatin should be managed according to standard local practice. Patients may be retreated according to standard local practice, including escalations of hypersensitivity prophylaxis, in-patient monitoring, increases in the duration of the infusion and use of formal desensitisation protocols (see ICON8 Clinical Management Guidelines for a suggested protocol).

If further hypersensitivity prevents the continued administration of carboplatin, substitution of cisplatin for carboplatin can be considered but due to the lack of evidence for the use of cisplatin with paclitaxel in dose-fractionated regimens, this would be considered off protocol treatment for trial purposes.

A4.4.2.5 LIVER TOXICITY
Hepatotoxicity is not expected with either chemotherapy drug and other causes of liver enzyme elevation should be actively pursued.

If transaminases become elevated and are <3 x ULN (CTCAE v4.0 grade 1) then treatment can be continued as per protocol without any dose modifications or delays. If transaminases become elevated to 3-5 x ULN (CTCAE v4.0 grade 2) then treatment can continue but dose reductions of paclitaxel may be performed according to local practice at the discretion of the treating physician.

If transaminases become elevated to >5 x ULN (CTCAE v4.0 grade 3) then treatment with paclitaxel should cease until resolution to <3 x ULN (CTCAE v4.0 grade 1).

A4.4.2.6 Rash Associated with Weekly Dose-Dense Paclitaxel
During ICON8, it has become apparent that some patients receiving weekly dose-dense paclitaxel have developed a skin rash on treatment. This most typically affects the dorsal surfaces of the hand/ forearm and is predominantly erythematous in nature. Associated skin peeling and occasionally blistering alongside pain, soreness and itching have been reported. Supportive management such as emollients, analgesia and antihistamines are recommended at investigators discretion.

It is recommended that if the rash is ≥ CTCAE grade 2 then weekly paclitaxel is omitted until recovery to ≤ CTCAE grade 1 and that subsequently the dose of weekly paclitaxel is reduced by one dose level (**see Table 6**).

A4.4.2.7 OTHER

There are no dose modifications planned for alopecia, nausea, diarrhoea, constipation or venous thromboembolism (including DVT and PE). These side effects should be treated with supportive medical measures. Non-steroidal anti-inflammatory agents can be used prophylactically, or symptomatically, as per local practice for the treatment of paclitaxel-induced arthromyalgia.

For any other adverse event of CTCAE v4.0 grade 4 severity considered at least possibly related to study treatment, the patient should be discontinued from protocol therapy after discussion with the MRC CTU in the first instance who will liaise with the Chief Investigator where necessary.

For any other adverse event of CTCAE v4.0 grade 3 severity considered at least possibly related to study treatment, treatment should be withheld until recovery to grade 1 or less and subsequent treatment should be reduced by one dose level (see **Table 6**).

Any patients who develop malignant effusions while on therapy may have them drained according to local practice, assuming coagulation parameters and platelet counts are adequate.

A4.4.3 STOPPING PACLITAXEL OR CARBOPLATIN FOR TOXICITY: ALTERNATIVE REGIMENS

A4.4.3.1 PACLITAXEL-SPECIFIC TOXICITY

If it becomes necessary to discontinue paclitaxel because of toxic effects (eg. neuropathy or hypersensitivity) then it is recommended that patients continue trial treatment with single agent carboplatin. This can be administered at the same AUC used in combination with paclitaxel or 1 AUC unit higher at the investigator's discretion. This information will continue to be collected on the trial CRFs.

Docetaxel, or any other chemotherapeutic agent, may NOT be substituted for paclitaxel in any situation as there is limited data for the efficacy of other platinum-combination regimens used on a weekly schedule.

Alternative combination chemotherapy regimens used in the standard care of patients with ovarian cancer may be used if the treating investigator feels that it is in the patient's best interests to receive combination therapy rather than single agent carboplatin but this will be considered off-protocol treatment for trial purposes. In this circumstance the patient should remain in the trial following the same visit schedule for the purposes of follow-up and data analysis (see **Section A3.8 Protocol Treatment Discontinuation**). For data completeness, and to avoid potential bias in the reporting of adverse events between arms, data on alternative first-line treatment will be collected on the trial CRFs.

A4.4.3.2 CARBOPLATIN-SPECIFIC TOXICITY

If it becomes necessary to discontinue carboplatin because of toxic effects (eg. hypersensitivity) substitution of cisplatin for carboplatin can be considered but due to the lack of evidence for the use of cisplatin with paclitaxel in dose-fractionated regimens, this would be considered off protocol treatment for trial purposes. If cisplatin is used, a recommended schedule would be $75mg/m^2$ 3-weekly with paclitaxel dosing left at the investigator's discretion. In this circumstance the patient should remain in the trial following the same visit schedule for the purposes of follow-up and data analysis (see **Section A3.8 Protocol Treatment Discontinuation**). For data completeness, and to avoid potential bias in the reporting of adverse events between arms, data on alternative first-line treatment will be collected on the trial CRFs.

A4.5 ARM 3: WEEKLY CARBOPLATIN-PACLITAXEL

A4.5.1 Haematological Toxicity

The administration of weekly carboplatin-paclitaxel can proceed if:

- ANC is ≥1.0 x 10^9/l
- PLT count is ≥75 x 10^9/l
- There is no complicating fever, infection or bleeding.

If these parameters are not met, the dose should be delayed by one week.

If haematological recovery occurs within 7 days, no dose modification is mandated and dosing is left to the discretion of the individual investigator. However, patients who require a dose delay for a low neutrophil count are likely to go on to have further delays. It is therefore recommended that G-CSF is administered with future cycles to reduce the risk of dose delays, maintain dose-intensity and improve treatment completion.

If haematological recovery occurs beyond 7 days or if two consecutive doses require a one-week deferral due to delayed haematological recovery, either G-CSF should be used to maintain dose intensity or the dose of both carboplatin and paclitaxel should be reduced by one dose level (see **Table 6**).

Dose limiting toxicities are defined, and dose modifications mandated, in **Table 8**. Please note that no dose delay or reduction is expected for anaemia but it should be managed using supportive measures that will maintain dose intensity.

For patients who receive chemotherapy with an ANC between 1.0-1.5 x 10^9/l, the use of prophylactic G-CSF support to reduce the risk of neutropenic complications and future dose delays is *strongly* recommended.

If G-CSF is used to maintain dose intensity for patients in arm 3, it must not be administered in the 48hrs immediately prior to or in the 24 hours following each administration of chemotherapy, ie. no G-CSF should be administered on days 6-9 and days 13-16 of each treatment cycle (for further details refer to the ICON8 trial specific clinical guidance document).

A4.5.2 Non-haematological toxicity

A4.5.2.1 Renal toxicity

The combination of carboplatin and paclitaxel, using the schedules described, is not directly expected to cause renal toxicity. There are, therefore, no specific dose modifications for renal toxicity. The GFR used to calculate the carboplatin dose should, however, be re-calculated or re-measured in the event of renal toxicity or changes in the serum creatinine – see **Section A3.4.2.3 Requirements for re-assessment of GFR during chemotherapy.**

A4.5.2.2 Neuropathy

Grade 2 sensory or motor neuropathy (CTCAEv4.0) requires paclitaxel treatment to be **interrupted** until neuropathy has resolved to grade ≤1. On recovery, paclitaxel should be reintroduced but with the dose reduced by 1 dose level (**see Table 6**). If this requires a delay of more than three weeks then the paclitaxel should be omitted from subsequent cycles and treatment continued with single agent carboplatin. Although it is recommended that weekly carboplatin is deferred alongside paclitaxel, it may be continued if the investigator feels this is clinically appropriate for a maximum of three weekly

doses. However, in this circumstance, any weekly doses of paclitaxel missed will not be replaced at the end of treatment.

Further details about the management of peripheral neuropathy on trial are given in the ICON8 Clinical Management Guidelines document.

Grade ≥3 sensory or motor neuropathy requires paclitaxel to be omitted from subsequent cycles, and treatment continued with single agent carboplatin.

IMPORTANT: For patients on Arm 3 continuing with carboplatin alone, the dose schedule should be converted to conventional 3-weekly chemotherapy (see **section 3.4.2: Carboplatin** for information on carboplatin dose). This change is mandated due to the lack of evidence supporting the use of dose-fractionated carboplatin as a single agent.

A4.5.2.3 Mucositis

For mucositis grade ≥3 (CTCAEv4.0) chemotherapy should be delayed until the mucositis has resolved to grade ≤1. Paclitaxel can be reduced by one dose level (**see Table 6**) in subsequent cycles at the discretion of the treating physician. If the mucositis recurs, or persists for more than three weeks, at grade ≥3 chemotherapy dose modifications should be discussed with the MRC CTU in the first instance who will liaise with the Chief Investigator where necessary.

Mucositis should be treated symptomatically as per standard local practice.

A4.5.2.4 Hypersensitivity

A hypersensitivity reaction to either drug is not expected to be a dose limiting toxicity but the occurrence & management of hypersensitivity reactions is being monitored as part of the toxicity analysis. A CRF should be completed on the occurrence of a hypersensitivity reaction. Suggested protocols for the management of carboplatin and paclitaxel hypersensitivity can be found in the ICON8 trial specific clinical guidance document.

Paclitaxel

If a hypersensitivity reaction occurs then patients may be retreated with paclitaxel. This will depend on the severity of the reaction and the specific reaction. Retreatment should be managed according to standard local practice or as suggested in the ICON8 trial specific clinical guidance document.
In the case of recurrent hypersensitivity reactions despite adequate premedication, the substitution of docetaxel for paclitaxel is not permitted as there is limited data on the efficacy of this treatment given on a weekly schedule. Treatment should continue with carboplatin alone.

IMPORTANT: For patients on Arm 3 continuing with carboplatin alone, the dose schedule should be converted to conventional 3-weekly chemotherapy (see **section 3.4.2: Carboplatin** for information on carboplatin dose). This change is mandated due to the lack of evidence supporting the use of dose-fractionated carboplatin as a single agent.

Carboplatin

A hypersensitivity reaction to carboplatin should be managed according to standard local practice. Patients may be retreated according to standard local practice, including escalations of hypersensitivity prophylaxis, in-patient monitoring, increases in the duration of the infusion and use

of formal desensitisation protocols (see ICON8 Clinical Management Guidelines for a suggested protocol).

If further hypersensitivity prevents the continued administration of carboplatin, substitution of cisplatin for carboplatin can be considered but due to the lack of evidence for the use of cisplatin with paclitaxel in dose-fractionated regimens, this would be considered off protocol treatment for trial purposes. If cisplatin is used, a recommended schedule would be 75mg/m^2 3-weekly with paclitaxel dosing left at the investigator’s discretion.

A4.5.2.5 LIVER TOXICITY

Hepatotoxicity is not expected with either chemotherapy drug and other causes of liver enzyme elevation should be actively pursued.

If transaminases become elevated and are <3 x ULN (CTCAE v4.0 grade 1) then treatment can be continued as per protocol without any dose modifications or delays. If transaminases become elevated to 3-5 x ULN (CTCAE v4.0 grade 2) then treatment can continue but dose reductions of paclitaxel may be performed according to local practice at the discretion of the treating physician.

If transaminases become elevated to >5 x ULN (CTCAE v4.0 grade 3) then treatment with paclitaxel should cease until resolution to <3 x ULN (CTCAE v4.0 grade 1).

A4.5.2.6 OTHER

There are no dose modifications planned for alopecia, nausea, diarrhoea constipation or venous thromboembolism (including DVT and PE). These side effects should be treated with supportive medical measures. Non-steroidal anti-inflammatory agents can be used prophylactically, or symptomatically, as per local practice for the treatment of paclitaxel-induced arthromyalgia.

For any other adverse event of CTCAE v4.0 grade 4 severity considered at least possibly related to study treatment, the patient should be discontinued from protocol therapy after discussion with the MRC CTU in the first instance who will liaise with the Chief Investigator where necessary.

For any other adverse event of CTCAE v4.0 grade 3 severity considered at least possibly related to study treatment, treatment should be withheld until recovery to grade 1 or less and subsequent treatment should be reduced by one dose level (**see Table 6**).

Any patients who develop malignant effusions while on therapy may have them drained according to local practice, assuming coagulation parameters and platelet counts are adequate.

A4.5.3 STOPPING PACLITAXEL OR CARBOPLATIN FOR TOXICITY: ALTERNATIVE REGIMENS

A4.5.3.1 PACLITAXEL-SPECIFIC TOXICITY

If it becomes necessary to discontinue paclitaxel because of toxic effects (eg. neuropathy or hypersensitivity) then it is recommended that patients continue trial treatment with single agent carboplatin. However, the dose schedule should be converted to conventional 3-weekly carboplatin with an AUC equivalent to 3 times that of the weekly dose or 1 AUC unit higher at the investigators discretion at the investigator’s discretion. This information will continue to be collected on the trial CRFs.

Docetaxel, or any other chemotherapeutic agent, may NOT be substituted for paclitaxel in any situation as there is limited data for the efficacy of other platinum-combination regimens used on a weekly schedule.

Alternative combination chemotherapy regimens used in the standard care of patients with ovarian cancer may be used if the treating investigator feels that it is in the patient's best interests to receive combination therapy rather than single agent carboplatin but this will be considered off-protocol treatment for trial purposes. In this circumstance the patient should remain in the trial following the same visit schedule for the purposes of follow-up and data analysis (see **see Section A3.8 Protocol Treatment Discontinuation**). For data completeness, and to avoid potential bias in the reporting of adverse events between arms, data on alternative first-line treatment will be collected on the trial CRFs.

A4.5.3.2 CARBOPLATIN-SPECIFIC TOXICITY

If it becomes necessary to discontinue carboplatin because of toxic effects (eg. hypersensitivity) substitution of cisplatin for carboplatin can be considered but due to the lack of evidence for the use of cisplatin with paclitaxel in dose-fractionated regimens, this would be considered off protocol treatment for trial purposes. If cisplatin is used, a recommended schedule would be $75mg/m^2$ 3-weekly with paclitaxel dosing left at the investigator's discretion. In this circumstance the patient should remain in the trial following the same visit schedule for the purposes of follow-up and data analysis (see **Section A3.8 Protocol Treatment Discontinuation**). For data completeness, and to avoid potential bias in the reporting of adverse events between arms, data on alternative first-line treatment will be collected on the trial CRFs.

A5 ASSESSMENTS AND PROCEDURES

All assessments and trial procedures must be performed in compliance with the most up to date version of the protocol, the principles of Good Clinical Practice, any relevant research governance and other regulatory requirements as appropriate.

A5.1 VISIT SCHEDULE

Summary information on the timing of interventions, assessments for safety and efficacy, and trial visits are given in **Section A6: Trial Assessment Schedules ICON8**. Separate tables for each trial arm have been prepared depending on whether patients have had IPS or will be undergoing DPS.

Trial visits are scheduled:

- During chemotherapy, patients should be seen 3-weekly prior to administration of day 1 of each chemotherapy cycle
- End of Treatment Visit 6 weeks after day 1 of the last cycle of first-line chemotherapy[w]
- 6-weekly from the End of Treatment Visit until 9 months post-randomisation
- Then 3-monthly until 2 years post-randomisation
- Then 6-monthly for 4 years and annually thereafter.

After progression, follow-up data will be collected 6-monthly. Information on subsequent anti-cancer therapy will continue to be collected after progression.

Once a patient has been randomised, follow-up information and appropriate CRFs (see ICON8 CRF Completion Guidelines) should be completed even if the patient does not remain on the trial treatments. Full documentation of cancer treatments and follow-up will be required.

A5.2 PROCEDURES FOR ASSESSING EFFICACY

Progression definitions based on RECISTv1.1 and clinical criteria are given in **Appendix 8**.

A5.2.1 TUMOUR IMAGING

Imaging assessments will be performed as specified in **Section A6: Trial Assessment Schedules ICON8.**

Tumour assessments for progression require a CT scan or MRI of the pelvis and abdomen, and should also include any other known tumour sites. A CT scan is the preferred method of evaluation but MRI can be used. The same assessment technique must be used throughout the trial.

For patients who underwent IPS, imaging after three cycles of chemotherapy is not required unless clinically indicated.

[w] If a patient had to stop trial chemotherapy early (e.g. for toxicity) but continued with alternative first-line chemotherapy off-trial, the end of treatment visit should occur 6 weeks after day 1 of the last cycle of non-trial chemotherapy.

For patients in whom DPS or IDS is planned, mid-point imaging should be performed pre-operatively for surgical planning, generally during cycle 3. A further CT scan should be performed 4 weeks +/- 7 days post-operatively which will be the baseline assessment for documentation of future progression.

All patients should have an end of treatment CT scan which should take place 6 weeks +/- 2 weeks after day 1 of the final cycle of first-line chemotherapy.

At subsequent follow-up visits tumour assessments should only be performed if clinical symptoms are suggestive of recurrence or there is CA125 progression as defined by GCIG criteria (see **Appendix 8**). Radiological tumour assessment should then occur within 3 weeks of the date of suspected clinical or CA125 progression. If there is CA125 progression but no radiological documentation of disease progression then imaging should be repeated 3-monthly until protocol defined progression occurs.

A5.2.1.1 STAGE 2 ANALYSIS

For patients who will be included in the stage 2 activity analysis - approximately the first 186 patients randomised - a single additional CT scan is required at 9 months post-randomisation.

A5.2.2 CA125

Serum CA125 will not be used to define progression but does form part of the assessment procedures. It should be assessed by the same laboratory throughout the trial period and centres are required to submit the normal ranges for CA125 for the laboratory used. CA125 should be assessed at baseline (within a 7 day period prior to the first cycle of chemotherapy) and prior to day 1 of each 3-weekly chemotherapy cycle. It should then be measured at each follow-up visit.

During follow-up, if there is CA125 progression by GCIG criteria (see **Appendix 7**) a CT scan should be performed, and repeated 3-monthly until protocol defined progression occurs.

A5.3 PROCEDURES FOR ASSESSING SAFETY

Safety assessments will be performed as specified in in **Section A6: Trial Assessment Schedules ICON8**

Details on reporting of Serious Adverse Events are given in **Section 3: Safety Reporting**

During treatment, patients should be seen within 72 hours prior to day 1 of each chemotherapy cycle. Physical examinations will be performed including an assessment of weight and vital signs (pulse rate and blood pressure). Blood tests including standard haematology (Hb, WBC, ANC, PLT) and standard biochemistry (Na^+, K^+, Ca^{2+}, Urea (BUN), creatinine, albumin, ALP, ALT (SGPT) or AST (SGOT) and bilirubin) will be performed to evaluate changes in these laboratory parameters. Patients having weekly chemotherapy should also have standard haematology and biochemistry tests prior to days 8 and 15 of each chemotherapy cycle.

A5.4 TRANSLATIONAL RESEARCH SAMPLES

Samples for translational research (TR) will only be collected from patients in participating centres who have consented separately to the TR programme. Further details can be found in **Appendix 11**; and for detailed information on the processing, labelling, handling, storage and shipment of these specimens please refer to the TRICON8 manual.

In all patients participating in the TR component of ICON8, formalin-fixed paraffin-embedded (FFPE) tumour samples are required. This is a baseline surgical specimen from IPS patients. From DPS patients FFPE samples are required from the baseline diagnostic biopsy and at surgery. At centres who are participating at level 2 of the TR programme, an additional blood sample for DNA analysis is required at baseline. At centres who are participating at level 3 of the TR programme additional blood samples will be taken prior to commencement of cycle 1, cycle 2 and cycle 6, and at documentation of disease progression (or at 5 years from randomisation in patients who have not progressed). For TR purposes, LDH levels in the blood will be recorded in the CRF if this parameter has been measured routinely according to local practice.

A5.5 PROCEDURES FOR ASSESSING QUALITY OF LIFE ASSESSMENTS

The impact of dose-fractionated chemotherapy on quality of life (QoL) is a secondary end-point of the ICON8 final stage analysis. Cancer specific questionnaires (the EORTC QLQ OV-28, which incorporates the EORTC QLQ C-30)[43] and a generic preference based measure (EQ-5D)[44] will be administered to record QoL. Use of these instruments is standard for first-line chemotherapy trials in ovarian cancer. The main focus of the QoL analysis will be on symptoms related to ovarian cancer and trial treatments, and questions on overall health and overall QoL. A detailed plan for the analysis of QoL data will be developed. Incorporation of the EQ-5D instrument facilitates the expression of health related quality of life (HRQL) in terms of 'utilities' which are used to estimate patients' quality-adjusted survival duration.

An explanation sheet for clinicians about the use of the QoL forms is given in **Appendix 10**.

Completion of the QoL form (QLQC30, OV-28 and EQ-5D questionnaires) should occur before medical assessments are performed, or chemotherapy is administered.

The first QoL form needs to be completed by the patient at her screening visit, after consent to participate has been given and prior to randomisation. A QoL form should be completed at the start of each chemotherapy cycle from cycle 2 onwards, before surgery and at the end of treatment visit. During follow-up, they should be completed 3-monthly for the first 2 years (months 6, 9, 12, 15, 18, 21 and 24 post-randomisation) then 6-monthly until 5 years (months 30, 36, 42, 48, 54 and 60 post-randomisation). An additional QoL form should be completed at the time of progression, prior to starting treatment for 1st relapse. Patients who progress should continue to complete QoL forms at the times specified, where at all possible.

QoL forms should be completed without conferring with friends, relatives or health professionals and all questions should be answered even if the patient feels them to be irrelevant. Patients should complete the QoL form on their own, whilst waiting to be seen in the clinic prior to medical assessment or intervention, and in a quiet area if possible. The form should not be taken away to be completed at home. The clinician or nurse in charge of the patient should collect the form before the patient leaves and should be available to answer questions on the form if the patient wishes, but should not assist with its completion. The form should be checked to ensure that the dates of completion and patient identifiers are correct. The patient should be offered an envelope in which the questionnaire can be sealed if they wish to keep it confidential.

A5.6 HEALTH ECONOMICS ASSESSMENTS

A cost-effectiveness analysis of dose-fractionated chemotherapy is a secondary end-point of the ICON8 final stage analysis.

The EQ-5D, which is included with each QoL assessment, will contribute to the health economics analysis. Additional information will also be collected on medical resource use during chemotherapy, including a short questionnaire administered with each QoL assessment during chemotherapy, at the end of treatment visit and during follow-up.

A5.7 EARLY STOPPING OF FOLLOW-UP

In consenting to the trial, patients are consenting to trial treatment, trial follow-up and data collection. If a patient wishes to withdraw from trial treatment, they should always be followed up providing they are willing. Patients stopping follow-up early have a negative impact on a trial's data. Centres should explain the importance of remaining on trial follow-up or, failing this, of allowing routine follow-up data to be used for trial purposes. However, if patients do not wish to remain on trial follow-up their decision must be respected.

If the patient explicitly states their wish not to contribute further data to the study, the MRC CTU (or relevant participating GCIG group) should be informed in writing. However, data up to the time of consent withdrawal will be included in the data reported for the study. Consent for future use of stored translational research samples already collected can be refused when leaving the trial early (but this should be discouraged and follow a discussion).

Patients may change their minds about stopping trial follow-up at any time and re-consent to participation in the trial.

Patients who stop trial follow-up early will not be replaced.

Patients will be followed up in the long-term through usual mechanisms, which may include flagging with the NHS Information Centre or similar approaches, unless the patient has explicitly withdrawn consent for further data collection.

A5.8 PATIENT TRANSFERS

For patients moving from the area, every effort should be made for the patient to be followed-up at another participating trial centre and for this trial centre to take over responsibility for the patient, or for follow-up to continue via other health care professionals, eg. General Practitioner. Details of other participating GCIG groups and clinical centres can be obtained from the MRC CTU. The consent of patients should be obtained for their names to be flagged for survival information through national registries. If the investigator moves then appropriate arrangements should be made to arrange for trial follow-up to continue at the centre.

A copy of the patient's CRFs will need to be provided to the new site and all due CRFs up until that time point and data queries must be resolved. The patient will have to sign a new consent form at the new site, and until this occurs, the patient remains the responsibility of the original centre.

A5.9 LOSS TO FOLLOW-UP

Every effort should be made to follow-up patients who have been randomised. Patients should, if possible, remain under the care of an oncologist or gynaecologist for the duration of the trial. If the care of a patient is returned to the General Practitioner, it is still the responsibility of the investigator to ensure that the follow-up data required by the protocol is collected and reported.

A6 TRIAL ASSESSMENTS SCHEDULES ICON8

A6.1 Table 9: Trial Assessment Schedule for IPS Patients: ICON8 Arm 1

	Screening	Treatment period visits								
**Assessment**	Within 28 days of randomisation	Cycle 1	Cycle 2	Cycle 3	Cycle 4	Cycle 5	Cycle 6	End of treatment visit	Follow-up 6 weekly until 9 months	Follow-up 3 monthly until 2 years then 6 monthly for 4 years
Day		1	1	1	1	1	1			
Informed consent	X									
Demographics	X									
Medical history	X									
Physical examination	X	X[a]	X	X	X	X	X	X	X	X
Height	X									
Weight	X	X[a]	X	X	X	X	X			
ECG[b]	X									
Vital signs	X	X	X	X	X	X	X	X		
Performance Status	X	X[a]	X	X	X	X	X	X	X	X
Pregnancy test[c]	X	X[a]								
Coagulation[b]	X									
Haematology[d]	X	X[a]	X	X	X	X	X	X		
Biochemistry[d]	X	X[a]	X	X	X	X	X	X		
GFR	X[g]	(X[g])	(X[g])	(X[g])	(X[g])	(X[g])	(X[g])			
Carboplatin[e]		X	X	X	X	X	X			
Paclitaxel[f]		X	X	X	X	X	X			
Tumour assessments	X[h]							X[o]	X[p] (X[o])	(X[o])
CA 125[d]	X[i]	X[a]	X	X	X	X	X	X	X	X
Chest X-ray/CT scan	X[j]									
Concomitant medication		X	X	X	X	X	X	X		
Adverse Events		X	X	X	X	X	X	X	X	X
QoL Forms	X		X[k]	X[k]	X[k]	X[k]	X[k]	X	X	X
Tumour Tissue Block	X[l]									
Blood for DNA	X[m]									
Additional blood for Translational Research	X[n]		X[n]				X[n]		(X[n])	(X[n])
Medical Resource Use		X	X	X	X	X	X	X	X	X
Disease confirmation (histology)	X									

a. Repeat assessments not required if already performed during previous 7 days for screening purposes.
b. Repeat, if indicated, during study treatment.
c. A pregnancy test is only required for women of childbearing potential.
d. From cycle 2 onwards, may be done up to 72 hours before day 1 of each cycle. LDH is not required to be measured but will be recorded in the CRF if available.
e. Carboplatin (AUC5 or 6 depending on method used to calculate GFR) given on day 1.
f. Paclitaxel (175mg/m^2) given on day 1.
g. Either a measured (radioisotopic clearance or 24 hour urine collection) or calculated GFR (Wright, Cockcroft-Gault or Jelliffe formulae) can be used to calculate the carboplatin dose. A pre-randomisation measured GFR is mandatory if the calculated GFR is <60ml/min and must occur within 14 days before randomisation (28 days allowed in specific circumstances – see Section 6.5). If a calculated GFR is used it must be recalculated within 7 days before cycle 1 day 1. Repeat GFR measurement or calculation during treatment if: an increase in serum creatinine ≥grade 2 occurs or the serum creatinine changes by ≥10%, and at cycle 2 if there was significant doubt about the true GFR at cycle 1. Routine recalculation of the carboplatin dose at the start of each cycle is not expected unless these conditions are met (see Section 8.4.2).
h. The post-operative baseline tumour assessment should be performed no less than 4 weeks after surgery, and no more than 2 weeks after study treatment starts. The only exception to this is if clinically it is necessary to start treatment within 2 weeks of surgery. In this instance the baseline post-operative CT scan should be done at 4 weeks post-operatively (± 7 days).
i. If available, pre-surgery CA 125 measurement should also be provided.
j. Imaging of the chest must be performed to check for thoracic metastases in all patients prior to randomisation. This can be by CT or Chest X-ray, but if there is any clinical or radiological suspicion of parenchymal lung metastases or mediastinal metastases on the X-ray then a CT or MRI must be performed and disease measured according to RECIST criteria.
k. QoL form to be completed on day one of the cycle only.
l. Tumour tissue block from primary surgery. Required for all levels of Translational Research.
m. Blood for DNA. Ideally take prior to cycle 1 but can be taken at any time throughout treatment if necessary. Required for level 2 and above of Translational Research.
n. Blood samples for Translational Research level 3. To be taken prior to cycle 1, 2 and 6; and at documentation of disease progression or 5 years after randomisation if patient has not progressed.
o. End of treatment assessments should be done 6 weeks ±2 weeks after day 1 of the last cycle of first line treatment. Tumour assessments are only mandated at the end of treatment visit. At subsequent follow-up visits tumour assessments should only be performed if clinical examination/ symptoms are suggestive of recurrence or there is CA125 progression by GCIG criteria. They should then occur within 3 weeks of suspected clinical or CA125 progression. If there is CA125 progression but no radiological documentation of disease progression then imaging should be repeated 3-monthly until protocol defined progression is documented.
p. In patients that are part of the stage 2 analysis, an additional tumour assessment is required 9 months after randomisation.

A6.2 Table 10: Trial Assessment Schedule for IPS Patients: ICON8 Arm 2

	Screening	Treatment period visits																				
**Assessment**	Within 28 days of randomisation	Cycle 1			Cycle 2			Cycle 3			Cycle 4			Cycle 5			Cycle 6			End of treatment visit	Follow-up 6 weekly until 9 months	Follow-up 3 monthly until 2 years then 6 monthly for 4 years
Day		1	8	15	1	8	5	1	8	15	1	8	15	1	8	15	1	8	15			
Informed consent	X																					
Demographics	X																					
Medical history	X																					
Physical examination	X	X[a]			X			X			X			X			X			X	X	X
Height	X																					
Weight	X	X[a]			X			X			X			X			X					
ECG[b]	X																					
Vital signs	X	X			X			X			X			X			X			X		
Performance Status	X	X[a]			X			X			X			X			X			X	X	X
Pregnancy test[c]	X	X[a]																				
Coagulation[b]	X																					
Haematology[d]	X	X[a]	X	X	X	X	X	X	X	X	X	X	X	X	X	X	X	X	X	X		
Biochemistry[d]	X	X[a]	X	X	X	X	X	X	X	X	X	X	X	X	X	X	X	X	X	X		
GFR	X[g]	(X[g])			(X[g])			(X[g])			(X[g])			(X[g])			(X[g])					
Carboplatin[e]		X			X			X			X			X			X					
Paclitaxel[f]		X	X	X	X	X	X	X	X	X	X	X	X	X	X	X	X	X	X			
Tumour assessments	X[h]																			X[o]	X[p] (X[o])	(X[o])
CA 125[d]	X[i]	X[a]			X			X			X			X			X			X	X	X
Chest X-ray/CT scan	X[j]																					
Concomitant medication		X			X			X			X			X			X			X		
Adverse Events		X			X			X			X			X			X			X	X	X
QoL Forms	X				X[k]			X[k]			X[k]			X[k]			X[k]			X	X	X
Tumour Tissue Block	X[l]																					
Blood for DNA	X[m]																					
Additional blood for Translational Research	X[n]				X[n]												X[n]				(X[n])	(X[n])
Medical Resource Use		X			X			X			X			X			X			X	X	X
Disease confirmation (histology)	X																					

a. Repeat assessments not required if already performed during previous 7 days for screening purposes.
b. Repeat, if indicated, during study treatment.
c. A pregnancy test is only required for women of childbearing potential.
d. In each cycle, haematology and biochemistry tests are required prior to d1, 8 and 15 chemotherapy. LDH is not required to be measured but will be recorded in the CRF if available.
e. Carboplatin (AUC5 or 6 depending on method used to calculate GFR) given on day 1.
f. Paclitaxel (80mg/m^2) given on day 1, 8 and 15.
g. Either a measured (radioisotopic clearance or 24 hour urine collection) or calculated GFR (Wright, Cockcroft-Gault or Jelliffe formulae) can be used to calculate the carboplatin dose. A pre-randomisation measured GFR is mandatory if the calculated GFR is <60ml/min and must occur within 14 days before randomisation (28 days allowed in specific circumstances – see Section 6.5). If a calculated GFR is used it must be recalculated within 7 days before cycle 1 day 1. Repeat GFR measurement or calculation during treatment if: an increase in serum creatinine ≥grade 2 occurs or the serum creatinine changes by ≥10%, and at cycle 2 if there was significant doubt about the true GFR at cycle 1. Routine recalculation of the carboplatin dose at the start of each cycle is not expected unless these conditions are met (see Section 8.4.2).
h. The post-operative baseline tumour assessment should be performed no less than 4 weeks after surgery, and no more than 2 weeks after study treatment starts. The only exception to this is if clinically it is necessary to start treatment within 2 weeks of surgery. In this instance the baseline post-operative CT scan should be done at 4 weeks post-operatively (± 7 days).
i. If available, pre-surgery CA 125 measurement should also be provided.
j. Imaging of the chest must be performed to check for thoracic metastases in all patients prior to randomisation. This can be by CT or Chest X-ray, but if there is any clinical or radiological suspicion of parenchymal lung metastases or mediastinal metastases on the X-ray then a CT or MRI must be performed and disease measured according to RECIST criteria.
k. QoL form to be completed on day one of the cycle only.
l. Tumour tissue block from primary surgery. Required for all levels of Translational Research.
m. Blood for DNA. Ideally take prior to cycle 1 but can be taken at any time throughout treatment if necessary. Required for level 2 and above of Translational Research.
n. Blood samples for Translational Research level 3. To be taken prior to cycle 1, 2 and 6; and at documentation of disease progression or 5 years after randomisation if patient has not progressed.
o. End of treatment assessments should be done 6 weeks ±2 weeks after day 1 of the last cycle of first line treatment. Tumour assessments are only mandated at the end of treatment visit. At subsequent follow-up visits tumour assessments should only be performed if clinical examination/ symptoms are suggestive of recurrence or there is CA125 progression by GCIG criteria. They should then occur within 3 weeks of suspected clinical or CA125 progression. If there is CA125 progression but no radiological documentation of disease progression then imaging should be repeated 3-monthly until protocol defined progression is documented.
p. In patients that are part of the stage 2 analysis, an additional tumour assessment is required 9 months after randomisation.

A6.3 TABLE 11: TRIAL ASSESSMENT SCHEDULE FOR IPS PATIENTS: ICON8 ARM 3

Assessment	Screening	Treatment period visits																			
	Within 28 days of randomisation	Cycle 1			Cycle 2			Cycle 3			Cycle 4			Cycle 5			Cycle 6			End of treatment visit	Follow-up 6 weekly until 9 months	Follow-up 3 monthly until 2 years then 6 monthly for 4 years
Day		1	8	15	1	8	5	1	8	15	1	8	15	1	8	15	1	8	15			
Informed consent	X																					
Demographics	X																					
Medical history	X																					
Physical examination	X	X[a]			X			X			X			X			X			X	X	X
Height	X																					
Weight	X	X[a]			X			X			X			X			X					
ECG[b]	X																					
Vital signs	X	X			X			X			X			X			X			X		
Performance Status	X	X[a]			X			X			X			X			X			X	X	X
Pregnancy test[c]	X	X[a]																				
Coagulation[b]	X																					
Haematology[d]	X	X[a]	X	X	X	X	X	X	X	X	X	X	X	X	X	X	X	X	X	X		
Biochemistry[d]	X	X[a]	X	X	X	X	X	X	X	X	X	X	X	X	X	X	X	X	X	X		
GFR	X[g]	(X[g])			(X[g])			(X[g])			(X[g])			(X[g])			(X[g])					
Carboplatin[e]		X	X	X	X	X	X	X	X	X	X	X	X	X	X	X	X	X	X			
Paclitaxel[f]		X	X	X	X	X	X	X	X	X	X	X	X	X	X	X	X	X	X			
Tumour assessments	X[h]																			X[o]	X[p] (X[o])	(X[o])
CA 125[d]	X[i]	X[a]			X			X			X			X			X			X	X	X
Chest X-ray/CT scan	X[j]																					
Concomitant medication		X			X			X			X			X			X			X		
Adverse Events		X			X			X			X			X			X			X	X	X
QoL Forms	X				X[k]			X[k]			X[k]			X[k]			X[k]			X	X	X
Tumour Tissue Block	X[l]																					
Blood for DNA	X[m]																					
Additional blood for Translational Research	X[n]				X[n]												X[n]				(X[n])	(X[n])
Medical Resource Use		X			X			X			X			X			X			X	X	X
Disease confirmation (histology)	X																					

a. Repeat assessments not required if already performed during previous 7 days for screening purposes.
b. Repeat, if indicated, during study treatment.
c. A pregnancy test is only required for women of childbearing potential.
d. In each cycle, haematology and biochemistry tests are required prior to d1, 8 and 15 chemotherapy. LDH is not required to be measured but will be recorded in the CRF if available.
e. Carboplatin (AUC2) given on day 1, 8 and 15.
f. Paclitaxel (80mg/m^2) given on day 1, 8 and 15.
g. Either a measured (radioisotopic clearance or 24 hour urine collection) or calculated GFR (Wright, Cockcroft-Gault or Jelliffe formulae) can be used to calculate the carboplatin dose. A pre-randomisation measured GFR is mandatory if the calculated GFR is <60ml/min and must occur within 14 days before randomisation (28 days allowed in specific circumstances – see Section 6.5). If a calculated GFR is used it must be recalculated within 7 days before cycle 1 day 1. Repeat GFR measurement or calculation during treatment if: an increase in serum creatinine ≥grade 2 occurs or the serum creatinine changes by ≥10%, and at cycle 2 if there was significant doubt about the true GFR at cycle 1. In Arm 3, serum creatinine is being monitored weekly. If a single rise in serum creatinine occurs, investigators may exercise clinical judgment about the need to re-assess the GFR. However, if the increase in creatinine is sustained over more than one week, the GFR should be recalculated or remeasured. Routine recalculation of the carboplatin dose at the start of or during each cycle is not expected unless these conditions are met (see Section 8.4.2).
h. The post-operative baseline tumour assessment should be performed no less than 4 weeks after surgery, and no more than 2 weeks after study treatment starts. The only exception to this is if clinically it is necessary to start treatment within 2 weeks of surgery. In this instance the baseline post-operative CT scan should be done at 4 weeks post-operatively (± 7 days).
i. If available, pre-surgery CA 125 measurement should also be provided.
j. Imaging of the chest must be performed to check for thoracic metastases in all patients prior to randomisation. This can be by CT or Chest X-ray, but if there is any clinical or radiological suspicion of parenchymal lung metastases or mediastinal metastases on the X-ray then a CT or MRI must be performed and disease measured according to RECIST criteria.
k. QoL form to be completed on day one of the cycle only.
l. Tumour tissue block from primary surgery. Required for all levels of Translational Research.
m. Blood for DNA. Ideally take prior to cycle 1 but can be taken at any time throughout treatment if necessary. Required for level 2 and above of Translational Research.
n. Blood samples for Translational Research level 3. To be taken prior to cycle 1, 2 and 6; and at documentation of disease progression or 5 years after randomisation if patient has not progressed.
o. End of treatment assessments should be done 6 weeks ± 2 weeks after day 1 of the last cycle of first line treatment. Tumour assessments are only mandated at the end of treatment visit. At subsequent follow-up visits tumour assessments should only be performed if clinical examination/ symptoms are suggestive of recurrence or there is CA125 progression by GCIG criteria. They should then occur within 3 weeks of suspected clinical or CA125 progression. If there is CA125 progression but no radiological documentation of disease progression then imaging should be repeated 3-monthly until protocol defined progression is documented.
p. In patients that are part of the stage 2 analysis, an additional tumour assessment is required 9 months after randomisation

A6.4 TABLE 12: TRIAL ASSESSMENT SCHEDULE FOR DPS PATIENTS: ICON8 ARM 1

Assessment	Screening	Treatment period visits									
	Within 28 days of randomisation	Cycle 1	Cycle 2	Cycle 3	Surgery	Cycle 4	Cycle 5	Cycle 6	End of treatment visit	Follow-up 6 weekly until 9 months	Follow-up 3 monthly until 2 years then 6 monthly for 4 years
Day		1	1	1		1	1	1			
Informed consent	X										
Demographics	X										
Medical history	X										
Physical examination	X	X[a]	X	X		X	X	X	X	X	X
Height	X										
Weight	X	X[a]	X	X		X	X	X			
ECG[b]	X										
Vital signs	X	X	X	X		X	X	X	X		
Performance Status	X	X[a]	X	X		X	X	X	X	X	X
Pregnancy test[c]	X	X[a]									
Coagulation[b]	X										
Haematology[d]	X	X[a]	X	X	X	X	X	X	X		
Biochemistry[d]	X	X[a]	X	X		X	X	X	X		
GFR	X[g]	(X[g])	(X[g])	(X[g])		(X[g])	(X[g])	(X[g])			
Carboplatin[e]		X	X	X		X[p]	X	X			
Paclitaxel[f]		X	X	X		X[p]	X	X			
Tumour assessments	X[h]				X[n]	X[q]			X[r]	X[s] (X[r])	(X[r])
CA 125[d]	X	X[a]	X	X		X	X	X	X	X	X
Chest X-ray/CT scan	X[i]										
Concomitant medication		X	X	X		X	X	X	X		
Adverse Events		X	X	X	X	X	X	X	X	X	X
QoL Forms	X		X[j]	X[j]	X[t]	X[j]	X[j]	X[j]	X	X	X
Tumour Tissue Block	X[k]				X[k]						
Blood for DNA	X[l]										
Additional blood for Translational Research	X[m]		X[m]					X[m]		(X[m])	(X[m])
Medical Resource Use		X	X	X		X	X	X	X	X	X
Disease confirmation (histology)	X										
Primary Surgery					X[o]						

a. Repeat assessments not required if already performed during previous 7 days for screening purposes.
b. Repeat, if indicated, during study treatment.
c. A pregnancy test is only required for women of childbearing potential.
d. From cycle 2 onwards, may be done up to 72 hours before day 1 of each cycle. LDH is not required to be measured but will be recorded in the CRF if available.
e. Carboplatin (AUC5 or 6 depending on method used to calculate GFR) given on day 1.
f. Paclitaxel (175mg/m^2) given on day 1.
g. Either a measured (radioisotopic clearance or 24 hour urine collection) or calculated GFR (Wright, Cockcroft-Gault or Jelliffe formulae) can be used to calculate the carboplatin dose. A pre-randomisation measured GFR is mandatory if the calculated GFR is <60ml/min and must occur within 14 days before randomisation (28 days allowed in specific circumstances – see Section 6.5). If a calculated GFR is used it must be recalculated within 7 days before cycle 1 day 1. Repeat GFR measurement or calculation during treatment if: an increase in serum creatinine ≥grade 2 occurs or the serum creatinine changes by ≥10%, and at cycle 2 if there was significant doubt about the true GFR at cycle 1. Routine recalculation of the carboplatin dose at the start of each cycle is not expected unless these conditions are met (see Section 8.4.2).
h. The baseline tumour assessment should be performed within 4 weeks prior to randomisation.
i. Imaging of the chest must be performed to check for thoracic metastases in all patients prior to randomisation. This can be by CT or Chest X-ray, but if there is any clinical or radiological suspicion of parenchymal lung metastases or mediastinal metastases on the X-ray then a CT or MRI must be performed and disease measured according to RECIST criteria.
j. QoL form to be completed on day one of the cycle only.
k. Tumour tissue block from diagnostic biopsy and primary surgery. Required for all levels of Translational Research.
l. Blood for DNA. Ideally take prior to cycle 1 but can be taken at any time throughout treatment if necessary. Required for level 2 and above of Translational Research.
m. Blood samples for Translational Research level 3. To be taken prior to cycle 1, 2 and 6; and at documentation of disease progression or 5 years after randomisation if patient has not progressed.
n. Midway tumour reassessment for pre-surgical planning, generally performed during cycle 3.
o. To be undertaken as close to Cycle 3 day 22 as possible, and within a maximum of 10 days after this provided that haematological recovery has occurred.
p. Chemotherapy to be started as soon as patient is fit enough between 1-6 weeks after DPS, ideally within 1-2 weeks.
q. Post-surgical CT scan to be performed 4 weeks ± 7 days after surgery.
r. End of treatment assessments should be done 6 weeks ± 2 weeks after day 1 of the last cycle of first line treatment. Tumour assessments are only mandated at the end of treatment visit. At subsequent follow-up visits tumour assessments should only be performed if clinical examination/ symptoms are suggestive of recurrence or there is CA125 progression by GCIG criteria. They should then occur within 3 weeks of suspected clinical or CA125 progression. If there is CA125 progression but no radiological documentation of disease progression then imaging should be repeated 3-monthly until protocol defined progression is documented.
s. In patients that are part of the stage 2 analysis, an additional tumour assessment is required 9 months after randomisation.
t. QoL form to be completed at the end of cycle 3 prior to surgery.

A6.5 TABLE 13: TRIAL ASSESSMENT SCHEDULE FOR DPS PATIENTS: ICON8 ARM 2

Assessment	Screening	Treatment period visits																					
	Within 28 days of randomisation	Cycle 1			Cycle 2			Cycle 3			Surgery	Cycle 4			Cycle 5			Cycle 6			End of treatment visit	Follow-up 6 weekly until 9 months	Follow-up 3 monthly until 2 years then 6 monthly for 4 years
Day		1	8	15	1	8	15	1	8	15		1	8	15	1	8	15	1	8	15			
Informed consent	X																						
Demographics	X																						
Medical history	X																						
Physical examination	X	X[a]			X			X				X			X			X			X	X	X
Height	X																						
Weight	X	X[a]			X			X				X			X			X					
ECG[b]	X																						
Vital signs	X	X			X			X				X			X			X			X		
Performance Status	X	X[a]			X			X				X			X			X			X	X	X
Pregnancy test[c]	X	X[a]																					
Coagulation[b]	X																						
Haematology[d]	X	X[a]	X	X	X	X	X	X	X		X	X	X	X	X	X	X	X	X	X	X		
Biochemistry[d]	X	X[a]	X	X	X	X	X	X	X		X	X	X	X	X	X	X	X	X	X	X		
GFR	X[g]	(X[g])			(X[g])			(X[g])				(X[g])			(X[g])			(X[g])					
Carboplatin[e]		X			X			X				X[p]			X			X					
Paclitaxel[f]		X	X	X	X	X	X	X	X			X[p]	X	X	X	X	X	X	X	X			
Tumour assessments	X[h]										X[n]	X[q]									X[r]	X[s] (X[r])	(X[r])
CA 125[d]	X	X[a]			X			X				X			X			X			X	X	X
Chest X-ray/CT scan	X[i]																						
Concomitant medication		X			X			X				X			X			X			X		
Adverse Events		X			X			X				X			X			X			X	X	X
QoL Forms	X				X[j]			X[j]			X[t]	X[j]			X[j]			X[j]			X	X	X
Tumour Tissue Block	X[k]										X[k]												
Blood for DNA	X[l]																						
Additional blood for Translational Research	X[m]				X[m]													X[m]				(X[m])	(X[m])
Medical Resource Use		X			X			X				X			X			X			X	X	X
Disease confirmation (histology)	X																						
Primary Surgery											X[o]												

a. Repeat assessments not required if already performed during previous 7 days for screening purposes.
b. Repeat, if indicated, during study treatment.
c. A pregnancy test is only required for women of childbearing potential.
d. In each cycle, haematology and biochemistry tests are required prior to d1, 8 and 15 chemotherapy. LDH is not required to be measured but will be recorded in the CRF if available.
e. Carboplatin (AUC5 or 6 depending on method used to calculate GFR) given on day 1.
f. Paclitaxel (80mg/m^2) given on day 1, 8 and 15. NB. Cycle 3 d15 to be omitted prior to surgery.
g. Either a measured (radioisotopic clearance or 24 hour urine collection) or calculated GFR (Wright, Cockcroft-Gault or Jelliffe formulae) can be used to calculate the carboplatin dose. A pre-randomisation measured GFR is mandatory if the calculated GFR is <60ml/min and must occur within 14 days before randomisation (28 days allowed in specific circumstances – see Section 6.5). If a calculated GFR is used it must be recalculated within 7 days before cycle 1 day 1. Repeat GFR measurement or calculation during treatment if: an increase in serum creatinine ≥grade 2 occurs or the serum creatinine changes by ≥10%, and at cycle 2 if there was significant doubt about the true GFR at cycle 1. Routine recalculation of the carboplatin dose at the start of each cycle is not expected unless these conditions are met (see Section 8.4.2).
h. The baseline tumour assessment should be performed within 4 weeks prior to randomisation.
i. Imaging of the chest must be performed to check for thoracic metastases in all patients prior to randomisation. This can be by CT or Chest X-ray, but if there is any clinical or radiological suspicion of parenchymal lung metastases or mediastinal metastases on the X-ray then a CT or MRI must be performed and disease measured according to RECIST criteria.
j. QoL form to be completed on day one of the cycle only.
k. Tumour tissue block from diagnostic biopsy and primary surgery. Required for all levels of Translational Research.
l. Blood for DNA. Ideally take prior to cycle 1 but can be taken at any time throughout treatment if necessary. Required for level 2 and above of Translational Research.
m. Blood samples for Translational Research level 3. To be taken prior to cycle 1, 2 and 6; and at documentation of disease progression or 5 years after randomisation if patient has not progressed.
n. Midway tumour reassessment for pre-surgical planning, generally performed during cycle 3.
o. To be undertaken as close to Cycle 3 day 22 as possible, and within a maximum of 10 days after this provided that haematological recovery has occurred.
p. Chemotherapy to be started as soon as patient is fit enough between 1-6 weeks after DPS, ideally within 1-2 weeks.
q. Post-surgical CT scan to be performed 4 weeks ± 7 days after surgery.
r. End of treatment assessments should be done 6 weeks ± 2 weeks after day 1 of the last cycle of first line treatment. Tumour assessments are only mandated at the end of treatment visit. At subsequent follow-up visits tumour assessments should only be performed if clinical examination/ symptoms are suggestive of recurrence or there is CA125 progression by GCIG criteria. They should then occur within 3 weeks of suspected clinical or CA125 progression. If there is CA125 progression but no radiological documentation of disease progression then imaging should be repeated 3-monthly until protocol defined progression is documented.
s. In patients that are part of the stage 2 analysis, an additional tumour assessment is required 9 months after randomisation.
t. QoL form to be completed at the end of cycle 3 prior to surgery.

A6.6 TABLE 14: TRIAL ASSESSMENT SCHEDULE FOR DPS PATIENTS: ICON8 ARM 3

Assessment	Screening	Treatment period visits																					
	Within 28 days of randomisation	Cycle 1			Cycle 2			Cycle 3			Surgery	Cycle 4			Cycle 5			Cycle 6			End of treatment visit	Follow-up 6 weekly until 9 months	Follow-up 3 monthly until 2 years then 6 monthly for 4 years
Day		1	8	15	1	8	15	1	8	15		1	8	15	1	8	15	1	8	15			
Informed consent	X																						
Demographics	X																						
Medical history	X																						
Physical examination	X	X[a]			X			X				X			X			X			X	X	X
Height	X																						
Weight	X	X[a]			X			X				X			X			X					
ECG[b]	X																						
Vital signs	X	X			X			X				X			X			X			X		
Performance Status	X	X[a]			X			X				X			X			X			X	X	X
Pregnancy test[c]	X	X[a]																					
Coagulation[b]	X																						
Haematology[d]	X	X[a]	X	X	X	X	X	X	X		X	X	X	X	X	X	X	X	X	X	X		
Biochemistry[d]	X	X[a]	X	X	X	X	X	X	X		X	X	X	X	X	X	X	X	X	X	X		
GFR	X[g]	(X[g])			(X[g])			(X[g])				(X[g])			(X[g])			(X[g])					
Carboplatin[e]		X	X	X	X	X	X	X	X			X[p]	X	X	X	X	X	X	X	X			
Paclitaxel[f]		X	X	X	X	X	X	X	X			X[p]	X	X	X	X	X	X	X	X			
Tumour assessments	X[h]										X[n]		X[q]								X[r]	X[s] (X[r])	(X[r])
CA 125[d]	X	X[a]			X			X				X			X			X			X	X	X
Chest X-ray/CT scan	X[i]																						
Concomitant medication		X			X			X				X			X			X			X		
Adverse Events		X			X			X				X			X			X			X	X	X
QoL Forms	X				X[j]			X[j]			X[t]	X[j]			X[j]			X[j]			X	X	X
Tumour Tissue Block	X[k]										X[k]												
Blood for DNA	X[l]																						
Additional blood for Translational Research	X[m]				X[m]													X[m]				(X[m])	(X[m])
Medical Resource Use		X			X			X				X			X			X			X	X	X
Disease confirmation (histology)	X																						
Primary Surgery											X[o]												

a. Repeat assessments not required if already performed during previous 7 days for screening purposes.
b. Repeat, if indicated, during study treatment.
c. A pregnancy test is only required for women of childbearing potential.
d. In each cycle, haematology and biochemistry tests are required prior to d1, 8 and 15 chemotherapy. LDH is not required to be measured but will be recorded in the CRF if available.
e. Carboplatin (AUC2) given on day 1, 8 and 15. Cycle 3 d15 to be omitted prior to surgery.
f. Paclitaxel (80mg/m^2) given on day 1, 8 and 15. NB. Cycle 3 d15 to be omitted prior to surgery.
g. Either a measured (radioisotopic clearance or 24 hour urine collection) or calculated GFR (Wright, Cockcroft-Gault or Jelliffe formulae) can be used to calculate the carboplatin dose. A pre-randomisation measured GFR is mandatory if the calculated GFR is <60ml/min and must occur within 14 days before randomisation (28 days allowed in specific circumstances – see Section 6.5). If a calculated GFR is used it must be recalculated within 7 days before cycle 1 day 1. Repeat GFR measurement or calculation during treatment if: an increase in serum creatinine ≥grade 2 occurs or the serum creatinine changes by ≥10%, and at cycle 2 if there was significant doubt about the true GFR at cycle 1. In Arm 3, serum creatinine is being monitored weekly. If a single rise in serum creatinine occurs, investigators may exercise clinical judgment about the need to re-assess the GFR. However, if the increase in creatinine is sustained over more than one week, the GFR should be recalculated or remeasured. Routine recalculation of the carboplatin dose at the start of or during each cycle is not expected unless these conditions are met (see Section 8.4.2).
h. The baseline tumour assessment should be performed within 4 weeks prior to randomisation.
i. Imaging of the chest must be performed to check for thoracic metastases in all patients prior to randomisation. This can be by CT or Chest X-ray, but if there is any clinical or radiological suspicion of parenchymal lung metastases or mediastinal metastases on the X-ray then a CT or MRI must be performed and disease measured according to RECIST criteria.
j. QoL form to be completed on day one of the cycle only.
k. Tumour tissue block from diagnostic biopsy and primary surgery. Required for all levels of Translational Research.
l. Blood for DNA. Ideally take prior to cycle 1 but can be taken at any time throughout treatment if necessary. Required for level 2 and above of Translational Research.
m. Blood samples for Translational Research level 3. To be taken prior to cycle 1, 2 and 6; and at documentation of disease progression or 5 years after randomisation if patient has not progressed.
n. Midway tumour reassessment for pre-surgical planning, generally performed during cycle 3.
o. To be undertaken as close to Cycle 3 day 22 as possible, and within a maximum of 10 days after this provided that haematological recovery has occurred.
p. Chemotherapy to be started as soon as patients is fit enough between 1-6 weeks after DPS, ideally within 1-2 weeks.
q. Post-surgical CT scan to be performed 4 weeks ± 7 days after surgery.
r. End of treatment assessments should be done 6 weeks ± 2 weeks after day 1 of the last cycle of first line treatment. Tumour assessments are only mandated at the end of treatment visit. At subsequent follow-up visits tumour assessments should only be performed if clinical examination/ symptoms are suggestive of recurrence or there is CA125 progression by GCIG criteria. They should then occur within 3 weeks of suspected clinical or CA125 progression. If there is CA125 progression but no radiological documentation of disease progression then imaging should be repeated 3-monthly until protocol defined progression is documented.
s. In patients that are part of the stage 2 analysis, an additional tumour assessment is required 9 months after randomisation.
t. QoL form to be completed at the end of cycle 3 prior to surgery.

SECTION B: ICON8B

This section includes information on the selection, treatment and follow-up of patients entering the ICON8B randomisation.

It should not be used to guide the treatment or follow-up of any patients entering the original ICON8 randomisation. For those patients, please refer to Section A.

The original ICON8B pathway was a phase III randomised trial investigating the combination of dose-fractionated chemotherapy and bevacizumab compared to either strategy alone for the first-line treatment of women with newly diagnosed high-risk stage III-IV epithelial ovarian, fallopian tube or primary peritoneal cancer.

As of 5th May 2017, following analysis of mature Progression-free survival data from the ICON8 trial, recruitment to arm B2 of ICON8B is suspended and participants will be randomised into a 2-arm comparison study (arm B1 vs arm B3) as described in section B.

B1 SELECTION OF PATIENTS

There will be **no exceptions** to eligibility requirements at the time of randomisation. Questions about eligibility criteria should be addressed prior to attempting to randomise the participant.

The eligibility criteria for this trial have been carefully considered. The eligibility criteria are the standards used to ensure that only medically appropriate patients are considered for this study. Patients not meeting the criteria should not join the study. For the safety of the patients, as well as to ensure that the results of this study can be useful for making treatment decisions regarding other patients with similar diseases, it is important that no exceptions be made to these criteria for admission to the study.

Participants will be considered eligible for enrolment in this trial if they fulfil all the inclusion criteria and none of the exclusion criteria as defined below.

B1.1 PATIENT INCLUSION CRITERIA

1. Females aged ≥18 years

2. Signed informed consent and ability to comply with the protocol

3. Histologically confirmed, with core biopsy from a disease site as minimum requirement (cytology alone is insufficient for diagnosis):
   - Epithelial ovarian carcinoma
   - Primary peritoneal carcinoma of Müllerian histological type
   - Fallopian tube carcinoma
   - Ovarian carcinosarcoma (malignant mixed Müllerian tumour (MMMT) of the ovary).

4. High-risk disease defined as:

- FIGO (2013) Stage IIIA1(ii), IIIA2 with positive retroperitoneal lymph nodes >10mm in diameter, IIIB or IIIC disease
  i. With >1cm residual disease following IPS[24] *or*
  ii. Planned to undergo primary chemotherapy with or without DPS
- FIGO Stage IV disease
  i. With any volume of residual disease following IPS[24] *or*
  ii. Planned to undergo primary chemotherapy with or without DPS.

  NB. The FIGO 2013 staging system should be used for women entering ICON8B (see **Appendix 3**). Stage may be based on clinical and radiological assessment in patients who have not undergone IPS.

5. ECOG Performance Status (PS) 0-2

6. No clinical symptoms or radiological evidence of bowel obstruction (including sub-acute obstruction), abdominal fistulae or extensive recto-sigmoid involvement on imaging related to ovarian cancer
7. No recent history of proven active peptic ulcer disease, diverticulitis or inflammatory bowel disease (Crohns' Disease and ulcerative colitis) or any prior episode of gastrointestinal perforation.

---

[24] IPS should take place a maximum of 56 days prior to study entry

8. Life expectancy >12 weeks

9. Adequate bone marrow function:
    - Absolute Neutrophil Count (ANC) ≥1.5 x 10^9/l
    - Platelets (Plt) ≥100 x 10^9/l
    - Haemoglobin (Hb) ≥9g/dl (can be post transfusion).

10. Adequate liver function:
    - Serum bilirubin (BR) ≤1.5 x ULN
    - Serum transaminases ≤3 x ULN in the absence of parenchymal liver metastases or ≤5 x ULN in the presence of parenchymal liver metastases.

11. Adequate renal function as defined by:
    - Directly measured GFR (Glomerular Filtration Rate) ≥ 30 ml/min, or
    - Calculated creatinine clearance ≥ 60 ml/min.

    NB. If the calculated creatinine clearance is <60 ml/min the GFR should be directly measured using either a 24 hour urine collection or an isotopic evaluation.

12. Adequate coagulation profile:
    - International normalised ratio (INR) ≤1.5
    - Activated prothrombin time (APTT) ≤1.5xULN.

13. Able to start chemotherapy within 8 weeks after IPS (where applicable).

B1.2 PATIENT EXCLUSION CRITERIA

1. Non-epithelial ovarian cancer

2. Peritoneal cancer that is not of Müllerian origin, including mucinous histology

3. Borderline tumours (i.e. tumours of low malignant potential)

4. Clinical symptoms or radiological evidence of bowel obstruction (including sub-acute obstruction) or extensive recto-sigmoid involvement on imaging related to ovarian cancer

5. Prior systemic anti-cancer therapy for ovarian cancer (for example chemotherapy, monoclonal antibody therapy, tyrosine kinase inhibitor therapy or hormonal therapy)

6. Previous malignancies within 5 years prior to randomisation apart from:
    a. adequately treated carcinoma in-situ of the cervix, breast ductal carcinoma in-situ, non-melanomatous skin cancer; or
    b. previous/synchronous early-stage endometrial cancer defined as stage IA (FIGO 2009) grade 1 or 2 endometrioid cancers with no lymphovascular space invasion.

7. Pre-existing sensory or motor neuropathy CTCAE grade ≥2

8. Proteinuria at baseline:

- >1gm protein/24h by a 24-hour urine collection.

NB. Proteinuria should be initially assessed by urine dipstick. If urine protein is ≥2+ on urine dipstick, a 24-hour urine protein collection must be performed.

9. Significant co-existing or previous medical conditions that are contra-indications to treatment with bevacizumab, including:

   a. Cerebrovascular disease, including transient ischaemic attacks (TIAs), cerebrovascular accident (CVA; i.e. stroke) and intracranial bleeds (i.e. intra-cerebral haemorrhage, sub-arachnoid haemorrhage or sub-dural haemorrhage) within 6 months before trial entry

   b. Cardiovascular disease as follows:
      i. Uncontrolled hypertension, defined as sustained BP>150/100mmHg while receiving anti-hypertensive medication

      NB. Patients with a BP>150/100 mmHg prior to randomisation should be commenced on a calcium-channel blocker or other anti-hypertensive agent; or in the case of patients already on anti-hypertensives, medical therapy should be optimised. The BP should then be re-checked a few days later, if BP is controlled to ≤150/100mmHg the patient may be entered into the trial

      ii. Myocardial infarction or unstable angina within 6 months prior to randomization
      iii. New York Heart Association (NYHA) grade ≥2 congestive heart failure
      iv. Poorly controlled cardiac arrhythmia despite medication

      NB. Patients with rate-controlled atrial fibrillation are eligible

      v. Peripheral vascular disease grade ≥3, i.e. symptomatic and interfering with activities of daily living requiring repair or revision

   c. History or evidence of bleeding diathesis or coagulopathy (in patients not on therapeutic anti-coagulant medication)

10. Chronic daily use of high-dose aspirin, >325mg/day, within 10 days prior to study entry

11. Surgery (including open biopsy) or significant traumatic injury within 28 days prior to anticipated date of first dose of bevacizumab [25]

    NB. If IPS was performed within 28 days of planned start of treatment, patients are eligible but bevacizumab must be omitted from cycle 1.

12. Serious non-healing wound, worse than CTCAE Wound Complication or Wound Dehiscence grade 1

13. Active ulcer or bone fracture

[25] Laparoscopic procedures are permitted provided at least 7 days have elapsed between procedure and anticipated first dose of bevaciuzmab

14. Anticipated to require extensive dental work during protocol treatment

15. Evidence of any other disease/metabolic dysfunction that in the opinion of the investigator would put the subject at high-risk of treatment-related complications or prevent compliance with the trial protocol

16. Evidence of intra-abdominal free air not explained by paracentesis or recent surgical procedure

17. Symptomatic abdominal fistulae

18. History or clinical suspicion of brain metastases or spinal cord compression. CT/MRI of the brain is mandatory in the case of suspected brain metastases. Spinal MRI is mandatory in the case of suspected spinal cord compression. Patients with brain or meningeal metastases are not eligible

19. Sexually active women of childbearing potential not willing to use adequate contraception (e.g. oral contraceptives, intrauterine device or barrier method of contraception in conjunction with spermicidal jelly or surgically sterile) for the study duration and at least six months afterwards

20. Pregnant or lactating women who are currently breastfeeding

21. Known hypersensitivity to carboplatin, paclitaxel, bevacizumab or their excipients (including cremophor)

22. Planned intraperitoneal cytotoxic chemotherapy

23. Planned treatment with any other systemic anti-cancer therapy following completion of protocol treatment and prior to protocol defined progression

24. Any previous radiotherapy to the abdomen or pelvis

25. Treatment with any other investigational agent prior to protocol defined progression.

B1.3 CONCOMITANT MEDICATIONS

B1.3.1 Excluded concomitant medications

- Aspirin at doses >325mg/day.

B1.3.2 Medications to be used with caution

- **Medication affecting renal function**
  Caution should be exercised for patients using any medication that may markedly affect renal function. Such medications may be used with care if deemed essential by the responsible investigator or may be continued if already in use prior to entry in the trial with no effect on renal function

- **Anti-coagulants**

It is advised that patients receiving therapeutic anti-coagulation are converted to, or maintained on, a low molecular weight heparin preparation for the duration of their protocol chemotherapy. Warfarin is permitted during the maintenance phase, i.e. when patients are receiving single agent bevacizumab

- **Bisphosphonates**
  Previous or concomitant treatment with bisphosphonates in patients receiving bevacizumab may increase the risk of osteonecrosis of the jaw (ONJ). Patients randomised to bevacizumab who have received bisphosphonates should have a dental examination prior to commencing treatment. All patients receiving bevacizumab should be encouraged to report any symptoms of pain in the mouth, teeth or jaw; numbness or heaviness in the jaw; or looseness of a tooth for further investigation.

B1.3.3 DATA ON CONCOMITANT MEDICATION

All baseline concomitant medication will be recorded on the **Baseline Concomitant Medication CRF**, and any changes to concomitant medication during protocol treatment will be recorded on the **Chemotherapy CRF, End of Chemotherapy CRF** and the **Week 66 CRF**.

B1.4 NUMBER OF PATIENTS

660 patients will be recruited (330 to each arm) over 4 years.

Co-enrolment in previous or future trials is considered in **Section B2.3: Co-enrolment Guidelines**.

B1.5 SCREENING PROCEDURES & PRE-RANDOMISATION INVESTIGATIONS

Written informed consent to enter into the trial and be randomised must be obtained from participants after explanation of the aims, methods, benefits and potential hazards of the trial and BEFORE any trial-specific procedures are performed or any blood is taken for the trial (**see Appendix 9**).

It must be made completely and unambiguously clear that the participant is free to refuse to participate in all or any aspect of the trial, at any time and for any reason, without incurring any penalty or affecting their treatment.

Patients can be given as much time as they need to decide whether or not to participate in the trial but a **minimum of 24 hours** must be given from the time the trial information is given to the consent form being signed.

Signed consent forms must be kept by the investigator and documented in the CRF and a copy given to the participant. With consent, a letter should be sent to the general practitioner informing him/her of the trial and the participant's involvement in it (**see Appendix 9**).

B1.5.1 CONSENT OF PARTICIPANTS FROM 5TH MAY 2017 UNTIL THE IMPLEMENTATION OF PROTOCOL V7.0

For patients consenting to the study between the timepoints of 5th May 2017 to the implementation of protocol V7.0 (and associated amendment documents), the Consent Form Guidance (V1.0, 3rd May 2017) must be given to patients to read alongside the PIS/ICF. Research staff should document in the medical notes that the changes to the study have been discussed with all new patients and

that they are aware that they will only be randomised to arm B1 or B3 (and that arm B2 has been suspended).

B1.5.2 Baseline tests

The following baseline information should be obtained **within 28 days prior to randomisation unless otherwise stated**. The timing of some screening procedures (e.g. baseline tumour assessment) vary depending on whether patients have had IPS or not; specific screening pathways for post-IPS and planned DPS/primary chemotherapy participants are shown in **Figure 9.**

Investigations which have been performed as part of routine clinical practice can be used in the screening process. However, non-routine investigations or tests required for screening may only be performed following signature of the informed consent form.

Investigations performed prior to IPS are not acceptable for screening, except for a pre-surgical ECG, which may be used for screening providing it was normal and the patient has no history of cardiovascular disease.

Baseline information to be collected:

1. Demographic data, including patients initials, DOB and NHS number

2. Medical history, including previous and current diseases, and concomitant medications

3. In patients who have undergone IPS, details of surgery performed and volume of residual disease

4. Ethnicity, smoking status and family history of ovarian cancer in patients who consent to participate at Translational Research Level 2 or 3 only (**see Appendix 12**)

5. Physical examination including the assessment of height, weight and blood pressure

6. ECOG performance status

7. Blood tests
   a. Full Blood Count (FBC)
   b. Urea and Electrolytes (U&E): serum creatinine (SCr), urea (BUN), K+, Na+, Ca2+
   c. Liver Function Tests (LFT): Bilirubin, ALP, AST or ALT, albumin
   d. Coagulation: PT or INR, aPTT
   e. CA125

8. Assessment of renal function
   a. Measurement of GFR using a radio-isotopic method or 24 hour urine collection, *or*
   b. Calculation of GFR by the modified Wright, Cockcroft-Gault or Jeliffe formulae

   NB. Assessment of GFR should be performed **within 14 days** before randomisation. However, a radio-isotopic GFR performed within 28 days is acceptable provided the patient's serum creatinine has not changed by more than 10% between the test and randomisation.

9. Urine protein
    a. Urine dipstick for protein, and if protein is ≥2+ on urine dipstick,
    b. 24-hour urine protein collection

NB. If protein is <2+ on the screening urine dipstick, a 24-hour urine protein is NOT required

10. Other tests
    a. Pregnancy test (if of child bearing potential)
    b. Correlative bloods for translational research (if applicable)
    c. ECG

11. Imaging – see **Section B1.6: Initial Tumour Assessment** for details
    a. Tumour assessment
    b. Chest X-ray (if CT chest not performed as part of baseline tumour assessment)

12. Baseline quality of life form

13. Check all inclusion and exclusion criteria.

B1.5.3 ADDITIONAL PRE-CHEMOTHERAPY TESTS

Where haematology and biochemistry blood tests used for screening are taken more than 14 days prior to starting treatment, they must be repeated within 14 days before cycle 1 day 1. If a calculated GFR is being used for carboplatin dosing, this should be re-calculated using these blood tests (for instructions on carboplatin dosing on trial see **Section B3.4.2: Carboplatin**). CA125 should also be repeated if it has not been performed within 14 days prior to starting chemotherapy.

Where urine protein dipstick and blood pressure used for screening are taken more than 14 days prior to starting treatment, they must be repeated within 14 days before cycle 1 day 1.

The above is summarised, along with the other assessments required throughout the trial in **Section B6: Trial Assessment Schedules for ICON8B Tables 19, 20, 21, 22, 23, and 24.**

B1.6 INITIAL TUMOUR ASSESSMENT

The following information is summarised, with details of subsequent tumour assessments required throughout the trial, in **Section B6: Trial Assessment Schedules for ICON8B Tables 19, 20, 21, 22, 23, and 24.**

B1.6.1 IMMEDIATE PRIMARY SURGERY PATIENTS

- All patients should have cross-sectional imaging (preferably by CT scan although MRI is allowed) of the pelvis and abdomen. All subsequent follow-up scans should be the same modality (CT or MRI) and performed using the same technique

- The post-operative baseline tumour assessment should be performed no less than 4 weeks after surgery and no more than 2 weeks after protocol treatment starts. This is to avoid post-surgical changes that can complicate the interpretation of residual disease on the post-operative scan

The only exception to this is if it is clinically necessary to start chemotherapy within 2 weeks after surgery. In this case, the baseline tumour assessment should occur 4 weeks (+/- 7 days) after surgery

- Imaging of the chest must be performed to check for thoracic metastases in all patients prior to randomisation. This can be by CT or Chest X-ray, but if there is any clinical or radiological suspicion of parenchymal lung metastases or mediastinal metastases on the X-ray then a CT or MRI must be performed

- Scans should be assessed according to RECIST v1.1. Patients will be classified as having measurable or non-measurable disease, and target and non-target lesions identified if relevant (see **Appendix 8**).

B1.6.2 Delayed Primary Surgery Patients

- All patients should have cross-sectional imaging (preferably by CT scan although MRI is allowed) of the pelvis and abdomen. All subsequent follow-up scans should be the same modality (CT or MRI) and performed using the same technique

- The initial baseline scan must be done prior to randomisation. It should be performed within the 4-week period prior to randomisation. A longer window may be permitted at the discretion of the Chief Investigator/MRC CTU providing the interval between the scan date and cycle 1 day 1 will be less than 6 weeks. These cases should be discussed with the MRC CTU in the first instance who will liaise with the Chief Investigator where necessary.

- Imaging of the chest must be performed to check for thoracic metastases in all patients prior to randomisation. This can be by CT or Chest X-ray, but if there is any clinical or radiological suspicion of parenchymal lung metastases or mediastinal metastases on the X-ray then a CT or MRI must be performed

- Scans should be assessed according to RECIST v1.1. Patients will be classified as having measurable or non-measurable disease, and target and non-target lesions identified if relevant (see **Appendix 8**).

Figure 9: Timelines for Screening and Pre-treatment Investigations

A. Patients entering the trial following IPS

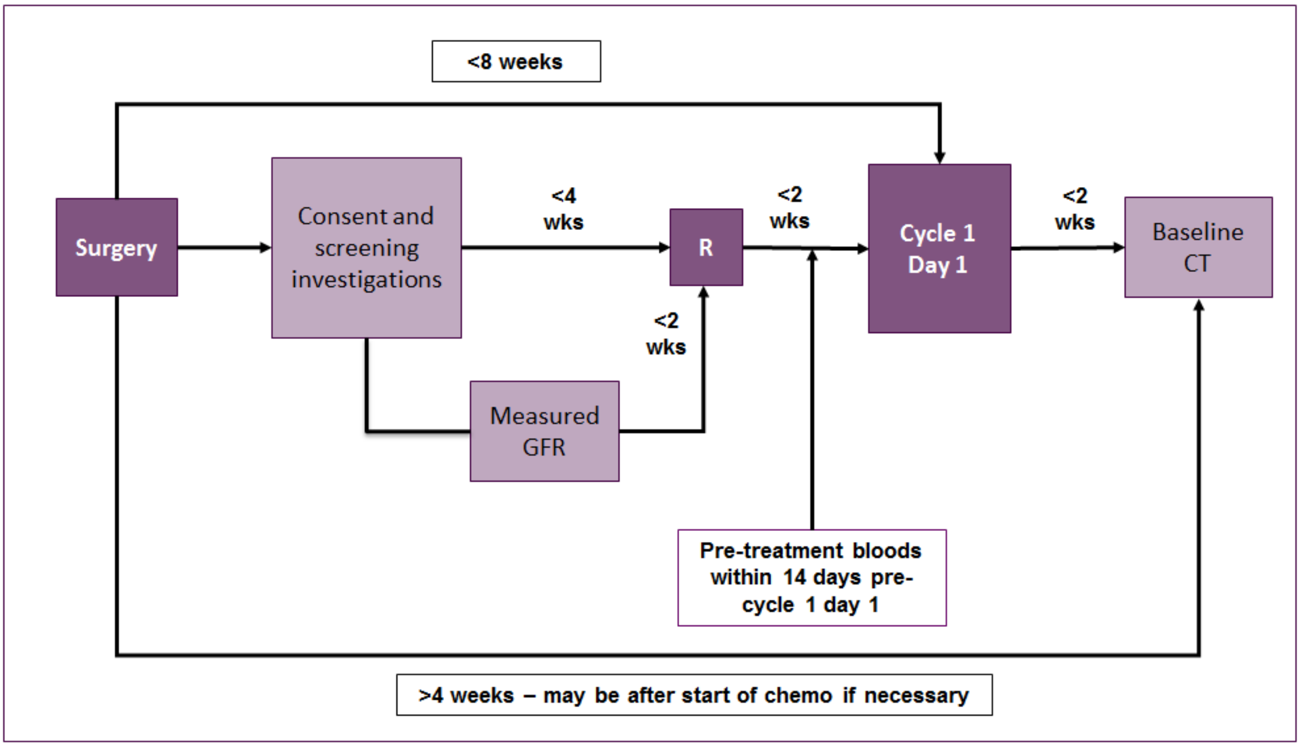

Key

Abbreviation	Expansion
R	Randomisation

NB. Investigations performed as part of routine clinical practice prior to consent may be used for screening purposes but any trial-specific screening procedures should only be performed following consent.

B. Patients entering the trial with a plan for DPS or primary chemotherapy alone

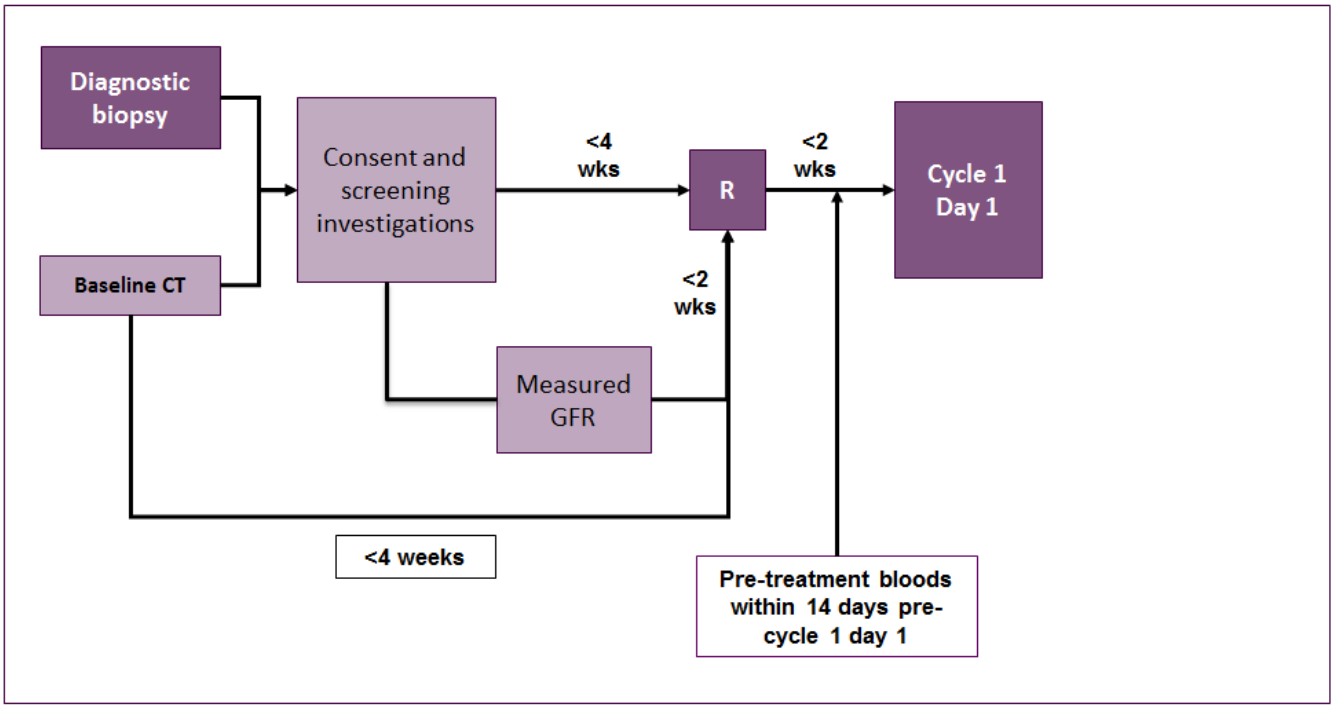

Key

Abbreviation	Expansion
R	Randomisation

NB. Investigations performed as part of routine clinical practice prior to consent may be used for screening purposes but any trial-specific screening procedures should only be performed following consent.

B2 REGISTRATION & RANDOMISATION

Patients will be randomly assigned in a 1:1 ratio to receive either:

- **Arm B1 (Bevacizumab Control):**
  - Carboplatin AUC5 + paclitaxel 175mg/m^2 q21d x 6 cycles *plus*
  - Bevacizumab 7.5mg/kg q21d with chemotherapy and as maintenance to complete 18 cycles in total

- **Arm B3 (Experimental, dose-fractionated + bevacizumab):**
  - Carboplatin AUC5 q21d + paclitaxel 80mg/m^2 q7d x 6 cycles *plus*
  - Bevacizumab 7.5mg/kg q21d with chemotherapy and as maintenance to complete 18 cycles in total.

Treatment should commence within 2 weeks after randomisation.

B2.1 INFORMATION REQUIRED FOR RANDOMISATION

The following data will be required in order to enrol a patient:

1. Confirmation (yes or no) that the patient satisfies all the eligibility criteria

2. Confirmation (yes or no) that none of the exclusion criteria apply

3. Results for all the screening procedures specified in **Section B1.5**

4. Patient's date of birth

5. Patient's initials

6. FIGO (2013) stage

7. Histological subtype and grade

8. For patients who have undergone IPS: surgery date, outcome and if there is any plan to perform further interval debulking surgery (IDS)

9. For patients who have not undergone IPS: whether DPS is planned

10. In patients who have given specific consent, NHS number will be collected so that information about health status may be obtained, if needed during follow-up, from the NHS Information Centre, NHS Central Register or other NHS information system. Any identifiable patient information collected will be securely stored separately from other data.

11. Confirmation (yes or no) that the patient has consented to translational research

12. For patients who consent to translational research: confirmation of the level of consent.

NB: Information required at randomisation may vary for non-UK sites, please contact your country coordinator for further information.

B2.2 RANDOMISATION PROCEDURE

Patients will be randomised using the MRC CTU telephone randomisation service.

For international GCIG/ non-UK groups who are unable to call during operating hours, randomisation may be conducted via fax or directly by remote access to the randomisation service. The procedure for randomisations will be determined jointly by the MRC CTU and each individual GCIG/ Non-UK group as they join the study, and will be detailed in the group-specific protocol appendix.

RANDOMISATIONS
To randomise, call MRC CTU, Monday to Friday 09:00-17:00 (UK time)
Tel: +44 (0) 20 7670 4777

A manual randomisation process will be set up to cover any instances when the main electronic system is not working.

Following randomisation patients must be given an ICON8B patient card and instructed by their research team to carry the card at all times to allow identification that they are participating in a clinical trial.

B2.3 CO-ENROLMENT GUIDELINES

Co-enrolment in any therapeutic clinical trial is not allowed prior to protocol defined disease progression (see **Appendix 8),** including any chemotherapy trial or any other trial of an investigational product for the treatment of ovarian cancer. However, for patients originally randomised to arm B2, participation in a therapeutic clinical trial may be allowed if the local PI feels this is in the patient's best interests. These cases should be discussed with the MRC CTU in the first instance who will liaise with the Chief Investigator where necessary.

Co-enrolment in other clinical trials, e.g. of interventions in supportive care, may be permitted but should be discussed with the MRC CTU/Chief Investigator prior to entry. These cases should be discussed with the MRC CTU in the first instance who will liaise with the Chief Investigator where necessary.

Figure 10: ICON8B Treatment Schedule A. Immediate Primary Surgery

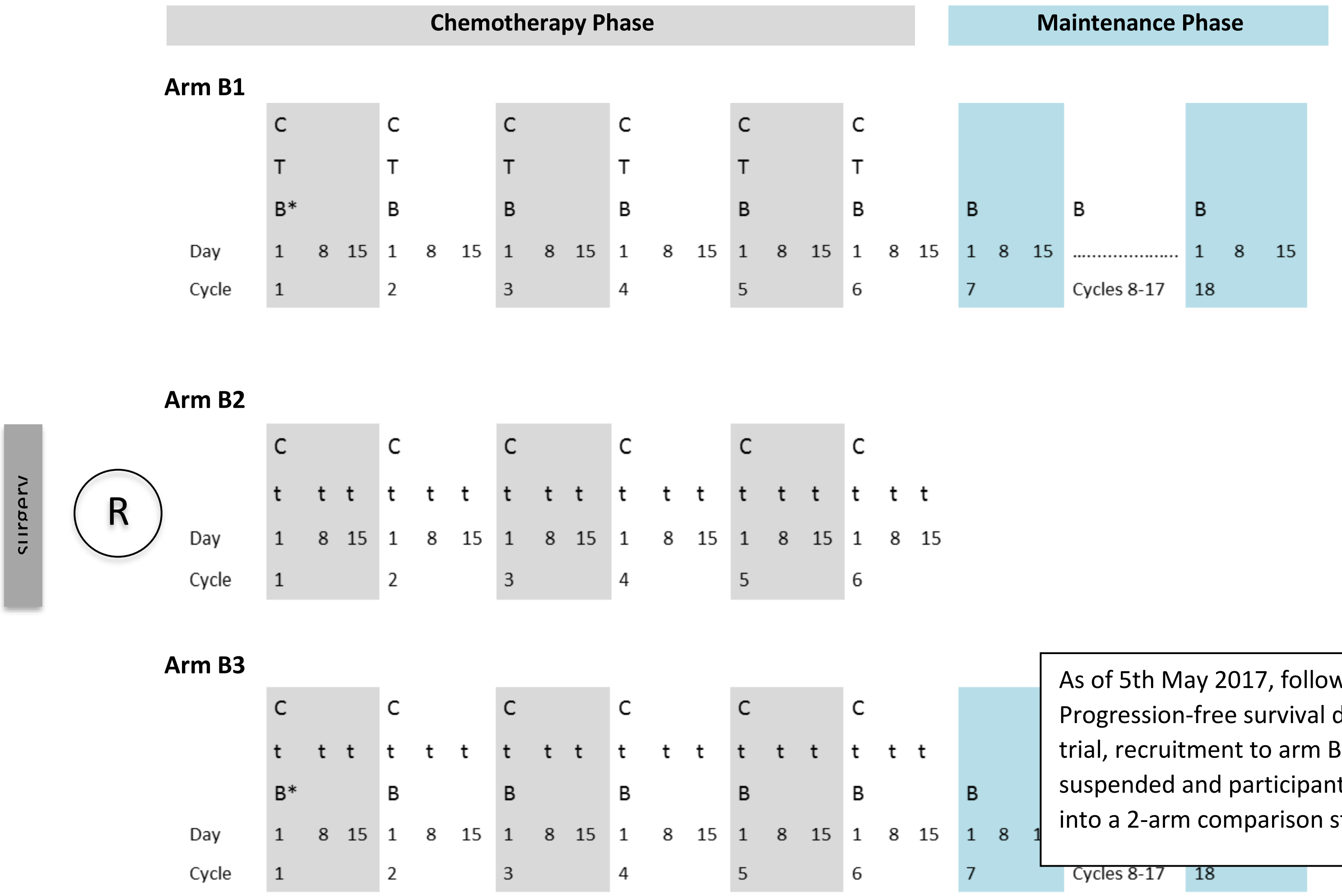

As of 5th May 2017, following analysis of mature Progression-free survival data from the ICON8 trial, recruitment to arm B2 of ICON8B is suspended and participants will be randomised into a 2-arm comparison study (arm B1 vs arm B3).

Key

Abbreviation	Expansion
**R**	Randomisation
**C**	Carboplatin
**T**	Paclitaxel 175mg/m²
**t**	Paclitaxel 80mg/m²
**B**	Bevacizumab

Timings

- Chemotherapy to commence within 8 weeks after surgery and within 2 weeks after randomisation.

Treatment modifications after surgery

- *Arms B1 and B3, omit bevacizumab if cycle 1 day 1 is less than 28 days after surgery OR patient has CTCAE Wound Complication or Wound Dehiscence worse than grade 1.

B. Delayed Primary Surgery

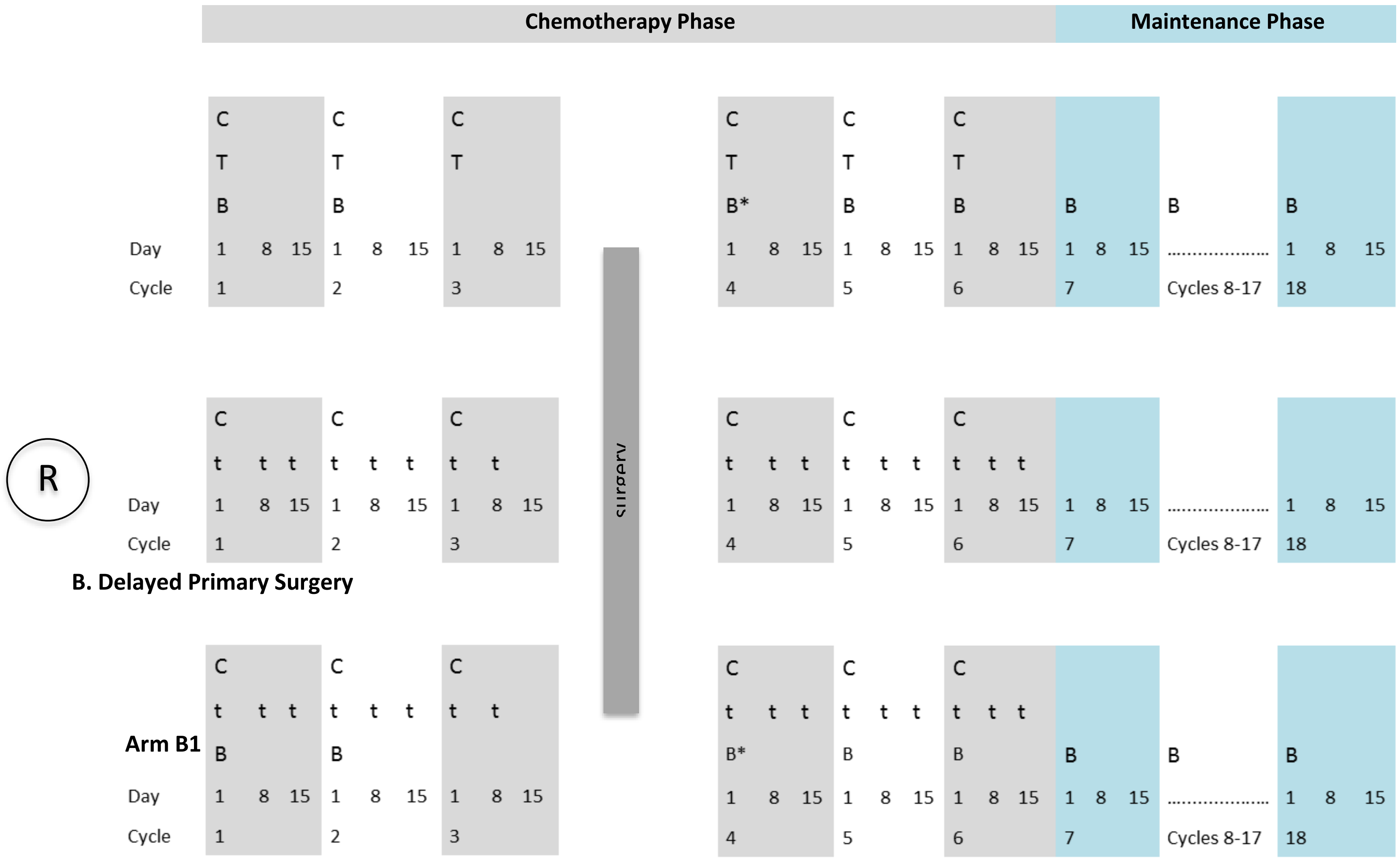

Key

Abbreviation	Expansion
**R**	Randomisation
**C**	Carboplatin
**T**	Paclitaxel 175mg/m^2
**t**	Paclitaxel 80mg/m^2
**B**	Bevacizumab

Timings

- Chemotherapy to commence within 2 weeks after randomisation
- Surgery to occur within 10 days after cycle 3 day 22
- Chemotherapy to recommence as soon as possible after surgery but at least 1 week after it

Treatment modifications around surgery

- Arms B1 and B3, omit bevacizumab from cycle 3
- Arms B2 and B3, omit cycle 3 day 15 paclitaxel
- *Arms B1 and B3, omit bevacizumab if cycle 4 day 1 is less than 28 days after surgery

Arm B2

As of 5th May 2017, following analysis of mature Progression-free survival data from the ICON8 trial, recruitment to arm B2 of ICON8B is suspended and participants will be randomised into a 2-arm comparison study (arm B1 vs arm B3).

Arm B3

B3 TREATMENT OF PATIENTS

B3.1 INTRODUCTION

The ICON8B trial is exploring alternative systemic anti-cancer therapy (SACT) strategies for the first-line treatment of high-risk ovarian cancer:

- **Arm B1 (Bevacizumab Control):**
  - Carboplatin AUC5 + paclitaxel 175mg/m^2 q21d x 6 cycles *plus*
  - Bevacizumab 7.5mg/kg q21d with chemotherapy and as maintenance to complete 18 cycles in total

- **Arm B2 (Dose-fractionated Control):** **As of 5th May 2017 participants will no longer be randomised into arm B2. Randomisations continue into arms B1 and B3.**
  - Carboplatin AUC5 q21d + paclitaxel 80mg/m^2 q7d x 6 cycles

- **Arm B3 (Experimental, dose-fractionated + bevacizumab):**
  - Carboplatin AUC5 q21d + paclitaxel 80mg/m^2 q7d x 6 cycles *plus*
  - Bevacizumab 7.5mg/kg q21d with chemotherapy and as maintenance to complete 18 cycles in total

The treatment regimens are described in detail in **Section B3.2: Treatment Arms**.

The trial is also assessing these regimens in the context of different surgical strategies: (i) in participants who have undergone immediate primary surgery (IPS) prior to entry in the trial, and (ii) in participants who receive primary (neo-adjuvant) chemotherapy and undergo delayed primary surgery (DPS) during treatment within ICON8B. Surgical treatment on trial is described in **Section 3.3: Surgery**.

In participants randomised to Arm B2 or B3 who undergo DPS, d15 paclitaxel must be omitted in the cycle immediately prior to surgery to reduce the likelihood of surgery being delayed due to myelosuppression.

Administration of bevacizumab around surgery is described in **Section B3.1.3: Use of Bevacizumab Around Surgery**.

Participants may receive full supportive care according to local practice, see **Section B3.6: Concomitant Therapy During Treatment on Trial**.

The treatment schedule incorporating timing of surgery and modification of the regimens around surgery is shown in **Figure 11**.

B3.1.1 COMMENCEMENT OF THERAPY

- All participants should commence chemotherapy within 2 weeks after randomisation
- In participants who have undergone IPS, treatment must commence within 8 weeks after their operation
- Any participants unable to comply with these requirements should be discussed with the MRC CTU.

B3.1.2 ANTICIPATED LENGTH OF TREATMENT COURSE

- In Arms B1 and B3, the length of the total treatment course (chemotherapy phase + maintenance phase) will be:
    - IPS patients, 54 weeks without interruptions
    - DPS patients, approximately 66 weeks assuming that DPS is performed on cycle 3 d22 with a 3 week post-op recovery period (median in ICON8 Stage IB analysis)

- In Arm B2, the length of the treatment course will be:
    - IPS patients, 18 weeks without interruptions
    - DPS patients, approximately 21 weeks assuming that DPS is performed on cycle 3 d22 with a 3 week post-op recovery period (median in ICON8 Stage IB analysis).

B3.1.3 USE OF BEVACIZUMAB AROUND SURGERY

Bevacizumab can cause problems with haemorrhage, thrombosis and wound healing and is contra-indicated within 28 days of surgery (as per the SPC). Therefore, bevacizumab must be omitted from:

1. IPS participants:

- Cycle 1 if chemotherapy commences within 28 days after IPS

2. DPS participants:

- Cycle 3 prior to surgery
- Cycle 4 if chemotherapy commences within 28 days after DPS

3. All patients in the presence of:

- CTCAE Wound Complication or Wound Dehiscence worse than grade 1.

B3.2 TREATMENT ARMS

Further details regarding the dosing and reconstitution of individual drugs are given in **Section B3.4: Specific Drug Information**, and on **modification of SACT around surgery** in **Section B3.3.3**.

Details regarding dose modifications, delays and omissions for safety are given in **Section B4: Drug Safety Information.**

B3.2.1 ARM B1: 3-WEEKLY CARBOPLATIN + PACLITAXEL + BEVACIZUMAB

B3.2.1.1 Chemotherapy phase

- 6 cycles of therapy administered on day 1 every 3 weeks consisting of:
    - Bevacizumab 7.5mg/kg administered over 30-90 minutes
    - Paclitaxel 175mg/m^2 administered over 180 minutes
    - Carboplatin AUC5 administered over 30-60 minutes[26]

- For IPS patients, bevacizumab must be omitted from cycle 1 if it is administered less than 28 days after surgery

- For patients undergoing DPS,
    - Cycle 3 bevacizumab must be omitted

[26] In ICON8 and ICON8B the recommended dose of three-weekly carboplatin is AUC5 with a measured GFR or a GFR calculated by the Wright method. If the Cockcroft-Gault or Jelliffe formulae are used to calculate the GFR, the carboplatin dose is AUC6.

  - Bevacizumab must be omitted from cycle 4 if it is administered less than 28 days after surgery.

B3.2.1.2 Maintenance phase

- Followed by maintenance bevacizumab on day 1 every 3 weeks given as:
  - Bevacizumab 7.5mg/kg over 30 minutes (or fastest rate tolerated previously)

- Up to 18 doses of bevacizumab should be administered in total
  - For IPS patients this will usually be 6 doses with chemotherapy and 12 maintenance cycles
  - For DPS patients this will usually be 4-5 doses with chemotherapy and 13-14 maintenance cycles
  - If fewer than 6 doses are administered with chemotherapy, additional maintenance cycles should be given to make the total course up to 18

- Participants may proceed to the maintenance phase if:
  - They have received an adequate course of chemotherapy, which is defined as at least 4 cycles of a platinum-based regimen
  - There is no protocol defined disease progression (see **Appendix 8**) on the end of chemotherapy tumour assessment (see **Section B5.2: Procedures for Assessing Efficacy**)
  - The 1st cycle of maintenance bevacizumab should be given on schedule 3 weeks after the start of the final cycle of chemotherapy. If results of the end of chemotherapy tumour assessment are not available by that date, maintenance bevacizumab should commence on schedule but a definitive decision on treatment continuation should be made based on the findings of the tumour assessment before the 2nd maintenance cycle is administered.

B3.2.2 Arm B2: 3-weekly carboplatin + weekly paclitaxel

As of 5th May 2017 participants will no longer be randomised into arm B2. Randomisations continue into arms B1 and B3.

B3.2.2.1 Chemotherapy phase

- 6 cycles of chemotherapy each administered over 3 weeks consisting of:
  - Paclitaxel 80mg/m^2 administered over 60 minutes on days 1, 8 and 15
  - Carboplatin AUC5 administered over 30-60 minutes on day 1

- For patients undergoing DPS, cycle 3 day 15 paclitaxel should be omitted.

B3.2.3 Arm B3: 3-weekly carboplatin + weekly paclitaxel + bevacizumab

B3.2.3.1 Chemotherapy phase

- 6 cycles of therapy each administered over 3 weeks consisting of:
  - Bevacizumab 7.5mg/kg administered over 30-90 minutes
  - Paclitaxel 80mg/m^2 administered over 60 minutes on days 1, 8 and 15
  - Carboplatin AUC5 administered over 30-60 minutes on days 1

- For IPS patients, bevacizumab must be omitted from cycle 1 if it is administered less than 28 days after surgery

- For patients undergoing DPS,
  - Cycle 3 bevacizumab must be omitted

  - Cycle 3 day 15 paclitaxel should be omitted
  - Bevacizumab must be omitted from cycle 4 if it is administered less than 28 days after surgery.

B3.2.3.2 Maintenance phase

- Followed by maintenance bevacizumab on day 1 every 3 weeks given as:
  - Bevacizumab 7.5mg/kg over 30 minutes (or fastest rate tolerated previously).

- Up to 18 doses of bevacizumab should be administered in total
  - For IPS patients this will usually be 6 doses with chemotherapy and 12 maintenance cycles
  - For DPS patients this will usually be 4-5 doses with chemotherapy and 13-14 maintenance cycles
  - If fewer than 6 doses are administered with chemotherapy, additional maintenance cycles should be given to make the total course up to 18.

- Participants may proceed to the maintenance phase if:
  - They have received an adequate course of chemotherapy, which is defined as at least 4 cycles of a platinum-based regimen
  - There is no protocol defined disease progression (see **Appendix 8**) on the end of chemotherapy tumour assessment (see **Section B5.2: Procedures for Assessing Efficacy**)
  - The 1st cycle of maintenance bevacizumab should be given on schedule 3 weeks after the start of the final cycle of chemotherapy. If results of the end of chemotherapy tumour assessment are not available by that date, maintenance bevacizumab should commence on schedule but a definitive decision on treatment continuation should be made based on the findings of the tumour assessment before the 2nd maintenance cycle is administered.

B3.3 SURGERY

Cytoreductive (debulking) surgery either performed as the first treatment (IPS) or following neo-adjuvant chemotherapy (DPS) is standard care for women with advanced ovarian cancer. It is expected that most participants will undergo surgery for their ovarian cancer but the decision to operate and timing of surgery (IPS vs. DPS), is at the discretion of the local investigator and Multi-Disciplinary Team.

Surgery should be performed in accordance with internationally accepted standards with the aim of achieving optimal debulking (see **Appendix 6).**

B3.3.1 Immediate Primary Surgery (IPS)

- Participants who have undergone IPS prior to entry in ICON8B must commence chemotherapy within 8 weeks after their operation.

B3.3.1.2 Interval Debulking Surgery (IDS)

- Participants in whom IPS was attempted but optimal cytoreduction was not achieved may undergo a second attempt at debulking surgery after 3 cycles of chemotherapy (interval debulking surgery, IDS) at the local investigator's discretion

- Intent to perform IDS should be declared at the time of enrolment into ICON8B and the treatment schedule for DPS patients should be followed.

B3.3.2 Delayed Primary Surgery (DPS)

- DPS should be performed following 3 cycles of neo-adjuvant chemotherapy

- To minimise breaks in chemotherapy, DPS should be undertaken as close to Cycle 3 day 22 as possible and within a maximum of 10 days after this providing that haematological recovery has occurred

- It is accepted that on occasion, administrative or other reasons may mean that surgery has to be scheduled after cycle 4 but efforts should be made by sites to minimise these occurences and ensure that surgery is performed in accordance within the specified timelines. This is particularly important in ICON8B due to the increased complexity associated with scheduling bevacizumab around DPS

- Post-operatively, chemotherapy should recommence between 1-6 weeks after DPS. To maintain treatment intensity, it is recommended that chemotherapy should be restarted as soon as the patient is fit enough, therefore it should ideally recommence within 1-2 weeks following surgery. However, there should be at least 1 week between DPS and post-operative chemotherapy to allow for post-surgical recovery. If post-operative chemotherapy is recommenced within 4 weeks after surgery, bevacizumab must be omitted from the 1st post-operative cycle (see **Section B3.3.3: Modifications of SACT Required Around Surgery**)

- It is acknowledged that, depending on the results of tumour assessments following 3 cycles of chemotherapy, surgery may not be appropriate in a small proportion of participants who were intended to have DPS at trial entry. In the event of early disease progression, the participant should stop protocol treatment (but trial follow-up should continue – see **Section B3.8: Protocol Treatment Discontinuation**). However, if the participant is considered to be benefitting from protocol treatment and does not meet the criteria for protocol defined progression (see **Appendix 8**) then they can continue to be treated within the ICON8B protocol without undergoing DPS

- It is accepted that in exceptional circumstances, participants who are not deemed suitable for DPS after 3 cycles may be considered for debulking surgery at completion of chemotherapy. These cases should be discussed with the MRC CTU in the first instance who will liaise with the Chief Investigator where necessary. The scheduling of bevacizumab will require modification to ensure patient safety. In these participants, follow-up should conform to the ICON8B trial protocol but an additional radiological assessment of disease will be required 4 weeks (+/- 7days) after their delayed surgery to act as a new baseline assessment for progression-free survival.

Timings around DPS are shown in **Figure 11**.

Any exceptions to these timings must be discussed with the MRC CTU in the first instance who will liaise with the Chief Investigator where necessary.

Figure 11: Timelines Around DPS

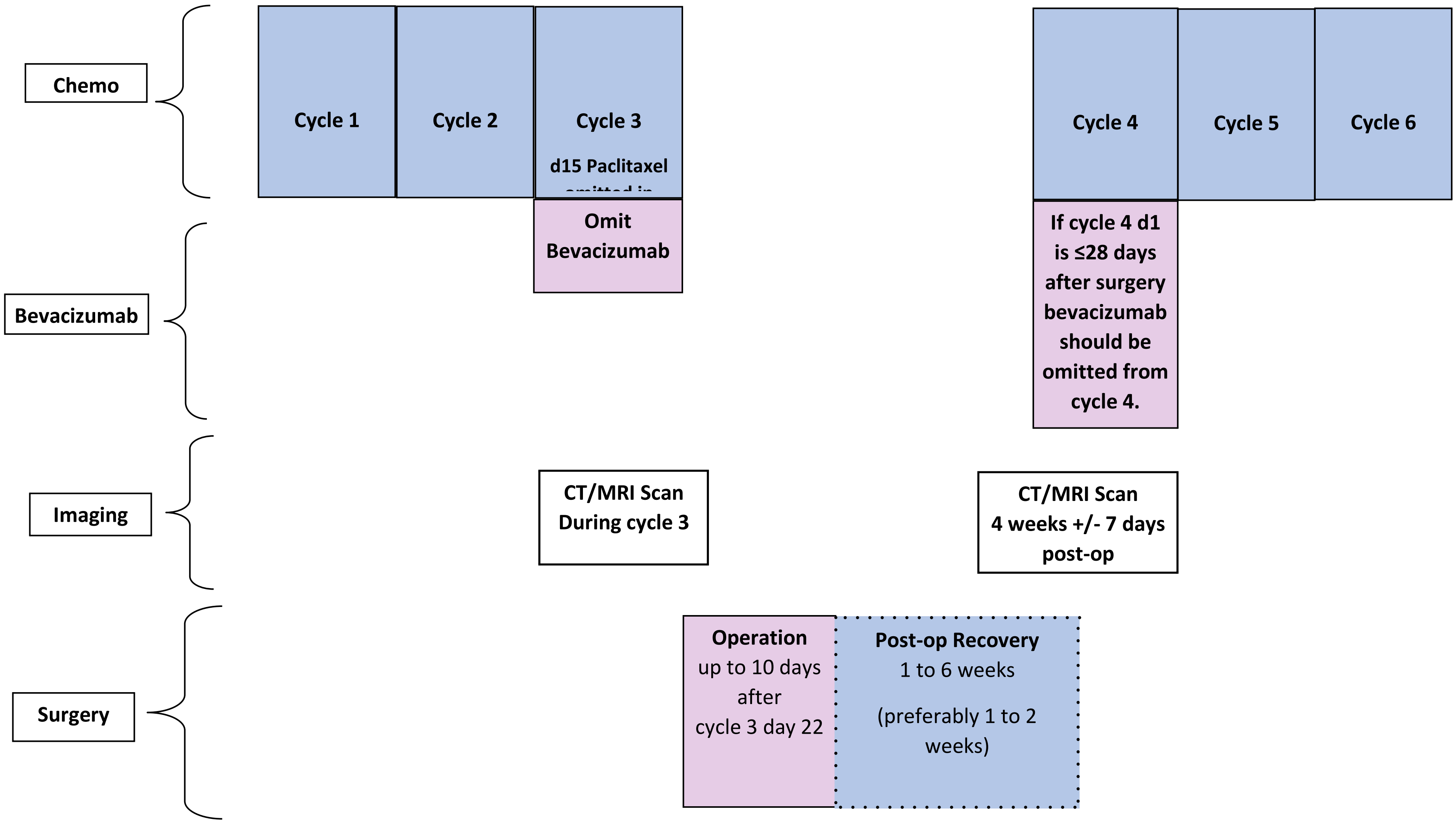

B3.3.3 Modifications of SACT Required Around Surgery

B3.3.3.1 Use of bevacizumab around surgery (Arms B1 and B3)

Bevacizumab can cause problems with haemorrhage, thrombosis and wound healing and is contra-indicated within 28 days of surgery (as per the SPC).

Therefore, bevacizumab must be omitted from:

1. IPS participants:
    - Cycle 1 if chemotherapy commences within 28 days after IPS

2. DPS participants:
    - Cycle 3 prior to surgery
    - Cycle 4 if chemotherapy commences within 28 days after DPS
3. All patients:
    - CTCAE Wound Complication or Wound Dehiscence worse than grade 1.

B3.3.3.2 Modification of weekly chemotherapy before DPS (Arms B2 and B3)

Myelosuppression from dose-fractionated chemotherapy may compromise the timing of surgery. Therefore, in women randomised to Arms B2 and B3 undergoing DPS, cycle 3 day 15 chemotherapy should be omitted prior to surgery to reduce the likelihood of this occurring.

No modifications to cycle 3 carboplatin or paclitaxel are planned for patients in Arm B1.

B3.4 SPECIFIC DRUG INFORMATION

- Carboplatin, paclitaxel and bevacizumab are all considered to be Investigational Medicinal Products (IMP) in ICON8B

- Each site will use their normally available generic hospital stock of carboplatin and paclitaxel

- In England bevacizumab for trial participants is funded by the Cancer Drugs Fund (CDF). Each site should make an individual CDF application as per their local practice, for each trial participant randomised to receive bevacizumab. Generic hospital stock of bevacizumab should then be used. For more details see **section B3.4.3: Bevacizumab**

- For sites outside of England guidance will be provided separately by the MRC CTU regarding the supply of bevacizumab within ICON8B

- The handling and management of all three drugs will be subject to standard local pharmacy procedures

- Each trial site pharmacy will be responsible for drug accountability and destruction at their site as detailed in the ICON8 Pharmacy Guidelines document. Drug accountability logs may form part of the central monitoring procedures for the trial (**see Section 4.2 Central Monitoring at the MRC CTU**)

- Dose banding (to within 5% of actual dose) of carboplatin and pacitaxel according to local practice is permissible.

- Use of the NHS England dose banding guidelines for carboplatin (V4, 1st Dec 2016) and paclitaxel (V1, 15th Mar 2016) are permitted within ICON8B.

- Bevacizumab doses can be rounded to the nearest 50 mg. Any alternative dose banding schedules must be approved by the MRC CTU and the Chief Investigator/TMG Pharmacist before application to ICON8B participants. For more details see **section B3.4.3: Bevacizumab**

- Carboplatin, paclitaxel and bevacizumab are all in routine use for the treatment of ovarian cancer at trial sites. Therefore, hypersensitivity pre-medication, anti-emetics and other supportive medication can be administered according to local practice. Recommendations, which are expected to be in accordance with most standard practice, are given below and more detailed suggestions for hypersensitivity prophylaxis and anti-emetic regimens are given in the ICON8B Clinical Management guidance document.

B3.4.1 PACLITAXEL

B3.4.1.1. Paclitaxel dosing

- The paclitaxel dose is based on body surface area (BSA), which should be calculated using a computer algorithm or according to standard nomograms. The BSA should only be recalculated if real body weight changes by more than 10%
- In all three arms, the dose should be capped at BSA 2.0
- Dose banding to within 5% of actual dose is permitted. The only exception to this is when the NHS England dose banding guidelines for paclitaxel are being used (V1, 15th Mar 2016).

B3.4.1.2 Three-Weekly Administration (Arm B1 Only)

- Hypersensitivity pre-medication, including H1 and H2 antagonists plus corticosteroids, must be administered prior to paclitaxel infusion, as per local standards

  For example:

  - 30 minutes prior to paclitaxel give
    - dexamethasone 20mg IV
    - chlorphenamine 10mg IV (push diluted with 5-10ml N Saline)
    - ranitidine 50mg IV (in 20ml N Saline over 2 minutes)

- Immediately pre-chemotherapy, give anti-emetics as per local standards; this may include oral or intravenous 5-HT3 antagonists

- Reconstitute paclitaxel 175mg/m^2 (cap at BSA 2.0m^2) in 500ml of N Saline or 5% dextrose according to local practice

- Administer via a non-PVC giving set and connectors incorporating a filter ≤ 0.22µm over 180 minutes via a rate-controlling device

- Anaphylaxis precautions should be available during infusion for the emergency treatment of hypersensitivity reactions

- Monitor closely for allergic reactions and cardiac arrhythmias as per local guidelines

- Use of a cold cap is permitted

- On extravasation, paclitaxel is a vesicant. Local guidelines for the management of extravasation should be followed.

B3.4.1.3 Weekly Administration (Arms B2 & B3)

- Hypersensitivity pre-medication, including H1/H2 antagonists plus corticosteroids, must be administered prior to paclitaxel infusion, as per local standards.

  For example:

  - 30 minutes prior to paclitaxel give
    - Dexamethasone between 7.6-8mg IV
    - chlorphenamine 10mg IV (push diluted with 5-10ml N. Saline)
    - ranitidine 50mg IV (in 20ml Normal Saline over 2 minutes)

  - If patients are unable to tolerate weekly dexamethasone at this dose and have not experienced paclitaxel hypersensitivity, the dose of dexamethasone can be gradually reduced at the investigator’s discretion. If a hypersensitivity reaction then develops, dexamethasone should be reintroduced at least between 7.6-8mg IV prior to **all** subsequent paclitaxel infusions (for further details, please refer to the ICON8B Clinical Management Guidance document)

- Immediately pre-chemotherapy give anti-emetics as per local standards; this may include oral or intravenous 5-HT3 antagonists

- Reconstitute paclitaxel 80mg/m^2 (cap at BSA 2.0m^2) in 250ml (or 100ml if dose is <75mg) N Saline or 5% dextrose according to local standard practice.

- Administer via a non-PVC giving set and connectors incorporating a filter ≤0.22µm over 60 minutes via a rate-controlling device

- Anaphylaxis precautions should be available during infusion for the emergency treatment of hypersensitivity reactions

- Monitor closely for allergic reactions and cardiac arrhythmias as per local guidelines

- Use of a cold cap is permitted

- On extravasation, paclitaxel is a vesicant. Local guidelines for the management of extravasation should be followed.

B3.4.2 CARBOPLATIN

B3.4.2.1 Carboplatin Dosing

The carboplatin dose is calculated according to the Calvert formula [40],

Carboplatin dose = Target AUC x (GFR + 25)

The exact dose of carboplatin therefore depends on the GFR and the method of calculating the GFR will also affect the carboplatin dose.

For the purpose of this protocol the GFR is considered equivalent to the creatinine clearance.

It is highly recommended that the GFR is assessed using an isotopic method except in the presence of significant third space fluid collections (ascites/pleural effusion/gross peripheral oedema) which would render this method inaccurate.

If a calculated creatinine clearance is used to calculate the carboplatin dose, then this should be done as per standard local practice using the Cockcroft-Gault, Jelliffe or Wright formulae (see **Appendix 5** for further details). When calculating creatinine clearance **actual body weight** should be used.

When concerns exist about carboplatin dosing in obese or elderly patients an isotopic GFR should be used instead.

If the calculated serum creatinine clearance is <60ml/minute, then a formal measurement of the GFR is mandatory, using either a 24-hour urine collection or an isotopic clearance. If the isotopic clearance is measured then the value uncorrected for body surface area (BSA) should be used in dose calculations.

The target AUC depends on the method of GFR assessment (see **Table 15**):

- Where the carboplatin dose is based on a GFR measured by isotopic clearance, or a 24-hour creatinine clearance (urine collection), the target AUC is 5
- If the Wright formula is used to calculate the creatinine clearance the target AUC is also 5, as results obtained using the Wright method have been shown to correspond to directly measured GFR in cancer patients [41, 42]
- If the Cockcroft-Gault or Jelliffe formulae have been used then the target AUC is 6, as results obtained using these formulae are typically lower than a directly measured GFR.

Dose capping of carboplatin may be carried out according to standard local practice, but it is recommended that the maximum three-weekly carboplatin dose should not exceed 900mg. If the GFR (either calculated or measured) is >100ml/minute, caution should be exercised with carboplatin dosing and the dose should be confirmed with the trial site's PI, or another clinician with equivalent experience in the use of carboplatin for the treatment of ovarian cancer.

Dose banding to within 5% of actual dose is permitted. The only exception to this is when the NHS England dose banding guidelines for carboplatin are being used ((V4, 1st Dec 2016)). See **Section B3.4: Specific Drug Information**).

Table 15: Target AUC Depending on GFR Calculation Method

	CALCULATED GFR	CALCULATED GFR	MEASURED GFR (RECOMMENDED)
	**Cockcroft-Gault and Jelliffe**	**Wright**	**Isotopic/24hr Urine**
**All arms**	AUC6	AUC5	AUC5

B3.4.2.2 GFR LIMITATIONS

1. Isotopic GFR is inaccurate in patients with significant effusions, ascites or oedema as the isotope distributes into third space fluid collections

2. Patients who have had complicated or prolonged post-operative recovery and who have been maintained on prolonged IV fluids with poor nutrition will have a falsely low serum creatinine

3. Formulae, such as the Wright, Cockcroft-Gault and Jelliffe, are inaccurate at the extremes of age and weight. The calculated GFR may be falsely high in obese young women and falsely low in thin elderly women. A measured GFR is particularly recommended in these patients

4. All formulae used to calculate creatinine clearance have limitations. These are further compounded due to different assays used to measure creatinine concentrations in different laboratories. This study is pragmatic with respect to this issue and the GFR/creatinine clearance should be measured/calculated as per standard local practice. If a serum creatinine obtained using an Isotope Dilution Mass Spectrometry (IDMS)-standardised assay has been used to calculate the GFR, a correction factor should not be used when calculating the carboplatin dose. However, investigators should be aware that serum creatinine values obtained using IDMS-standardised assays may be lower than those measured by non-IDMS methods, thus resulting in higher calculated GFR and carboplatin dose. The potential for carboplatin related toxicity may therefore be higher. The method used to measure serum creatinine will be recorded in the Investigator Site File.

It is assumed that clinicians entering patients into this protocol will be aware of these issues and the clinical judgement of an experienced clinician should be applied to the calculation of the carboplatin dose.

B3.4.2.3 REQUIREMENTS FOR REASSESSMENT OF GFR DURING CHEMOTHERAPY

The GFR should be recalculated, or re-measured, for:

- Renal toxicity (serum creatinine >1.5 x ULN, CTCAE v4.0 Grade 2)

- Serum creatinine changes of ≥10% compared to baseline, or last creatinine value used to calculate carboplatin dose (whichever is most recent)*

- Cycle 2, if there has been significant doubt about the true GFR at cycle 1 (according to clinical judgement).

Routine recalculation of the carboplatin dose at the start of each cycle is not expected unless the above conditions are met.

B3.4.2.4 Administration of carboplatin

- Reconstitute carboplatin to appropriate dose in 5% dextrose or N Saline according to standard local practice

- Give carboplatin intravenously over 30-60 minutes (depending on standard local practice)

- Allergic reactions to carboplatin are not a dose limiting toxicity and should be managed according to standard local practice. Patients may be re-challenged with increased prophylactic medications and/or slowing of infusion rates at the discretion of the treating investigator. Recommendations on the management of carboplatin hypersensitivity can be found in **section B4.2.2.4: Hypersensitivity** and the ICON8B Clinical Management Guidance document

- On extravasation, carboplatin is an irritant. Local guidelines for the management of extravasation should be followed.

B3.4.3 Bevacizumab

B3.4.3.1 Bevacizumab drug supply

In England, bevacizumab for ICON8B trial participants is funded by the Cancer Drugs Fund (CDF). Each site should make an individual CDF application as per their local practice, for each trial participant randomised to receive bevacizumab. Generic hospital stock of bevacizumab should then be used. Sites outside of England should refer to their Group Specific Operating Instructions for guidance on the provision of bevacizumab.

B3.4.3.2 Bevacizumab dosing

- The dose of bevacizumab is 7.5mg/kg
- No dose modifications are permitted
- Dose rounding to the nearest 50mg is allowed.
- The NHS England dose banding guidelines for bevacizumab (V1, 1st Dec 2016) are approved for use in ICON8B.

B3.4.3.3 Administration of bevacizumab

- Reconstitute bevacizumab to appropriate dose diluted up to a minimum volume of 100mL with N Saline. The constituted solution is stable if the final concentration is between 1.4 and 16.5 mg/ml

- If bevacizumab is being given in combination with chemotherapy then local policy should be followed with regard to the order that chemotherapy and bevacizumab is given

- No routine premedications, including anti-emetics, are required

- Bevacizumab should be administered as a continuous intravenous infusion via a rate regulating device. The total infusion time should be as per local practice providing the following timings are used as a minimum:
    - First cycle: given over 60 minutes
    - Second cycle: given over 30 minutes if there was no infusion-related reaction (e.g. fever/chills) with the first administration
    - Third, and all subsequent cycles: given over 15 minutes if the 30 minute infusion was well tolerated

- Bevacizumab infusion-related reactions are rare and are not usually expected to be a dose-limiting toxicity. Reactions should be managed according to usual local practice. Recommendations on the management of bevacizumab infusion-related reactions are given in **Section B4.4.4.12: Infusion and Hypersensitivity Reactions** and further detailed in the ICON8 Clinical Management Guidance document for sites without a local protocol. If the patient does experience an infusion-related reaction while receiving bevacizumab then that, and subsequent, doses should be infused over a longer time. For example if there are problems when bevacizumab is given over 15 minutes then it should subsequently be given over 30 minutes, and if there were problems when bevacizumab is given over 30 minutes then it should subsequently be given over 60 minutes.

B3.5 TRIAL TREATMENT RECORDING

Every administration of trial treatment must be recorded in the **Chemotherapy CRF**. In addition, reasons for any dose delays, dose reductions, dose omissions or permanent discontinuation of study treatment should also be documented in the **Chemotherapy CRF** and the **End of Chemotherapy CRF** as appropriate.

B3.6 CONCOMITANT THERAPY DURING PROTOCOL TREATMENT

All non-cancer treatments that the investigator feels are appropriate are allowed. Regular high dose aspirin (>325mg/day) should not be used in participants receiving bevacizumab (see **B1.3.1: Excluded Concomitant Medications**). Details of medication to be used with caution (medication affecting renal function, warfarin and bisphosphonates) are given in **B1.3.2: Medications to be used with caution**.

Any diagnostic, therapeutic or surgical procedure performed during the trial period should be recorded in the participant's medical records and the trial CRFs where appropriate.

Participants should receive full supportive care during and after the administration of chemotherapy in accordance with local practice. This includes transfusion of blood and blood products and/or the use of erythropoietin and/or G-CSF; antibiotics for infective complications; and anti-emetics. The treatment details should be recorded in the trial CRFs[27]. Anaphylaxis precautions should be observed during administration of carboplatin and paclitaxel as per local practice.

Treatment with concomitant, systemic anti-cancer agents (apart from those specified in the protocol) or other concurrent anti-cancer investigational agents of any type are not allowed in this trial before

[27] Anti-emetics routinely administered as part of the chemotherapy regimen do not need to be recorded on the Chemotherapy CRF.

protocol defined evidence of disease progression. The participant may only be entered into another therapeutic clinical trial of anti-cancer treatment for their ovarian cancer after documented protocol defined disease progression or withdrawal from this trial. (For guidelines on co-enrolment also see **section B2.3: Co-enrolment Guidelines**)

B3.7 TREATMENT AFTER PROGRESSION

Treatment after protocol-defined progression is at the discretion of the treating clinician.

All subsequent anti-cancer treatments including systemic anti-cancer therapy, radiotherapy and surgery should be recorded on the **Further Lines of Anti-cancer Therapy CRF**.

Following protocol defined progression participants should be reviewed 6-monthly for trial follow-up using the **Long Term Follow Up CRF**.

B3.8 PROTOCOL TREATMENT DISCONTINUATION

In consenting to the trial, participants are consenting to trial treatment, trial follow-up and data collection. However, an individual participant may stop treatment early for any of the following reasons:

- Progression whilst on therapy
- Unacceptable toxicity
- Inter-current illness which prevents further treatment
- Withdrawal of consent for treatment by the participant
- Any alterations in the participant's condition which justifies the discontinuation of treatment in the investigator's opinion.

As the participant's participation in the trial is entirely voluntary, they may choose to discontinue the trial treatment at any time. Although the participant is not required to give a reason for discontinuing their trial treatment, a reasonable effort should be made to establish this reason while fully respecting their rights.

Participants should however remain in the trial, following the same visit schedule, for the purposes of follow-up and data collection (unless they withdraw their consent from all stages of the trial).

If a patient switches to any of the chemotherapy regimens listed below, the details of treatment should be completed on the **Chemotherapy Phase Form** (Form 6) and **NOT** on the **Non-Trial Chemotherapy Phase Form** (Form 19):

- Patients switching to single agent Carboplatin on a 3 weekly basis

- Patients switching to standard 3 weekly Carboplatin and Paclitaxel
- Patients switching to 3 weekly carboplatin and weekly paclitaxel
- Patients switching to single agent Carboplatin and bevacizumab

Patients switching to these regimes should follow the protocol visit and assessments schedule for the purposes of follow-up and data analysis (unless they withdraw their consent from all stages of the trial).

If a participant is withdrawn from follow-up, refer to **Section B5.7: Early Stopping of Follow-up**.

B3.9 FOLLOW-UP

Once randomised, patients remain evaluable for the intention-to-treat analysis regardless of their subsequent course of treatment. Specific follow-up arrangements are described in **Section B5.1.3 Follow-up Phase.**

B3.10 GUIDANCE FOR PATIENTS RANDOMISED TO ARM B2

For patients randomised to arm B2 prior to the suspension of arm B2 (on 5th May 2017) the ICON8 TMG advises that in light of the ICON8 PFS results, clinicians should consider and discuss with each arm B2 patient whether they would prefer to continue on arm B2 for the remainder of their chemotherapy cycles or would wish to switch their regimen by adding bevacizumab (the schedule of arm B3) or by adding bevacizumab and switching to a 3 week schedule (arm B1). In most cases bevacizumab access would require (in England) application under the revised Cancer Drugs Fund. Administration of non-trial treatment should be discussed in the first instance with the MRC CTU.

Arm B2 patients who go on to receive non-trial treatment will remain on trial, following the same visit schedule where possible, for the purposes of follow-up and data collection (unless they withdraw their consent from all stages of the trial).

For patients that had been randomsied to arm B2 and who have completed protocol treatment, the TMG acknowledges that both investigators and patients may have some concerns regarding their treatment received within ICON8B. It is important to note that most women entering ICON8B have received neoadjuvant treatment. This is a clinical scenario in which there are limited published data reporting the use of bevacizumab, as this group of women were specifically excluded from ICON7 and GOG218, the two key phase III trials that evaluated incorporation of bevacizumab into first-line ovarian cancer treatment algorithms. We can reassure investigators that the survival of the cohort of patients receiving neoadjuvant weekly chemotherapy in ICON8 was longer than that reported in the primary chemotherapy arms of both the CHORUS and EORTC55971 trials, and similar to that seen for patients with high-risk disease receiving bevacizumab in the ICON7 trial.

B4 DRUG SAFETY INFOMATION

B4.1 GENERAL INFORMATION ON PACLITAXEL, CARBOPLATIN AND BEVACIZUMAB

Drug safety information on paclitaxel, carboplatin and bevacizumab is given in this section. Their toxicities alone and in combination are well established, however, the frequency of these toxicities may be altered by the weekly administration of paclitaxel employed in Arms B2 and B3. In particular, haematological toxicities may be exacerbated. In order to maintain consistency in dose and administration between participating GCIG groups and individual sites, a standard approach to dose modifications, delays and omissions is being applied and the guidance below should be followed for patients who develop adverse events.

General principles for the use of bevacizumab, both in combination with carboplatin and paclitaxel and as a single agent in the maintenance phase is available in **section B4.4.1: Bevacizumab General Principles**. Guidance on the management of adverse events in relation to bevacizumab, including where bevacizumab should be delayed, omitted or permanently discontinued is available in **section B4.4 Bevacizumab Modifications**. During the ICON8B trial we will be collecting information on a number of **notable events**, some of which require expedited SAE reporting, details of these can be found in **section 3.2: ICON8B Specific Notable Events**.

B4.1.1 Expected Adverse Events

A list of expected toxicities (based on the current UK SPCs) associated with carboplatin, paclitaxel and bevacizumab which will assist the treating physician in the classification of expectedness of serious adverse reactions is given in the investigator site file. Please see the current SPC for a list of expected toxicities associated with the drugs being used in this trial at https://www.medicines.org.uk/emc/ .

B4.1.2 Dose Modifications, Delays and Omissions of Carboplatin , Paclitaxel and Bevacizumab: General Principles

B4.1.2.1 Chemotherapy phase

In order to maintain the dose-intensity and cumulative dose-delivery of carboplatin and paclitaxel, reasonable efforts should be made to minimise dose reduction and treatment delays. Haematological treatment parameters for each arm are shown in **Table 17**. Early use of G-CSF to maintain dose-intensity, reduce likelihood of dose delays and improve treatment completion is strongly recommended in patients experiencing haematological toxicity (see ICON8B Clinical Management Guidance document for further details).

Patients whose treatment is delayed because of adverse events should be evaluated at weekly intervals (or less) until adequate recovery has occurred. As a general principle, if a patient is unable to receive a trial treatment due to an adverse event:

- If the scheduled treatment was Arm B1 d1, Arm B2 d1 and Arm B3 d1 (i.e. includes carboplatin), the dose should be delayed and administered when adequate recovery has occurred (with appropriate modification if indicated)
- If the scheduled treatment was Arm B2 d8 or 15 or Arm B3 d8 or d15 (i.e. weekly paclitaxel alone), the dose should be omitted
- If the scheduled treatment was Arm B1 d1 or Arm B3 d1 and chemotherapy has been delayed, bevacizumab should also be delayed. This is a pragmatic decision to simplify trial treatment scheduling.

Dose modifications for carboplatin and paclitaxel in each arm are shown in **Table 16**. If a dose modification is indicated, the dose of carboplatin and/or paclitaxel should be reduced by one dose level. Dose levels may be adjusted independently for each drug.

There are no dose escalations planned (including dose re-escalation after a dose reduction).

Patients who do not tolerate two separate carboplatin and/or paclitaxel dose reductions should discontinue treatment with trial chemotherapy. Any uncertainties about continuation should be discussed with the MRC CTU in the first instance who will liaise with the Chief Investigator where necessary.

If a patient experiences several toxicities and there are conflicting recommendations, please follow the most conservative dose adjustment recommended to minimise doses given.

Specific requirements for bevacizumab treatment during the maintenance phase are given in **Section B3.2.3.2: Maintenance Phase**

Table 16: Dose Levels for Paclitaxel and Carboplatin

	**Protocol Starting Dose**	**Protocol Dose Level -1**	**Protocol Dose Level -2**
**3-weekly paclitaxel** *(Arm B1)*	175mg/m^2	135mg/m^2	110mg/m^2
**3-weekly carboplatin** If measured GFR used or GFR calculated by Wright formula *(All arms)*	AUC5	AUC4	AUC3.5
**3-weekly carboplatin** If GFR calculated by Cockcroft-Gault or Jelliffe formulae *(All arms)*	AUC6	AUC5	AUC4.5

**Weekly paclitaxel** *(Arms B2 & B3)*	80mg/m^2	60mg/m^2	45mg/m^2

Specific requirements for dose modifications and delays or omissions in the event of particular adverse events are described in **Section B4.2** and **Section B4.3**.

For details of modifications of bevacizumab during the maintenance phase please refer to **Section B4.4: Bevacizumab Modifications (Arms B1 and B3).**

B4.2 ARM B1: 3-WEEKLY CARBOPLATIN-PACLITAXEL PLUS 3-WEEKLY BEVACIZUMAB

B4.2.1 Haematological Toxicity

Day 1 chemotherapy treatment should be delayed if either of the following occurs in the pre-chemotherapy full blood count:

- ANC is less than 1.0 x 10^9/l
- PLT count is less than 75 x 10^9/l.

FBC should then be repeated at least weekly until haematological recovery has occurred (ANC ≥1.0 x 10^9/l and PLT ≥75 x 10^9/l).

If haematological recovery occurs within 7 days, no dose modification is mandated and dosing is left to the discretion of the individual investigator.

If haematological recovery occurs beyond 7 days, it is suggested that doses of carboplatin and paclitaxel are modified according to the day 22 blood count (or subsequent FBC if lower) according to the criteria in **Table 17**. It is recommended that G-CSF prophylaxis is used in preference to dose reduction if this is feasible in order to maintain planned dose intensity.

For patients who receive chemotherapy with an ANC between 1.0-1.5 x 10^9/l, the use of prophylactic G-CSF support to reduce the risk of neutropenic complications and future dose delays is *strongly* recommended. If G-CSF is used it can be given in accordance with standard local practice but recommendations for its use are given in the ICON8B trial specific clinical guidance document.

Dose limiting toxicities are defined, and dose modifications mandated, in **Table 18**. Please note that no dose delay or reduction is expected for anaemia but it should be managed using supportive measures that will maintain dose intensity.

Patients who fail to recover adequate counts after a delay of 2 weeks or more, or who have consecutive dose limiting toxicities, are unlikely to be able to tolerate standard doses of carboplatin and paclitaxel. If it is considered in the patient's best interest to remain within the trial and to continue to receive treatment according to this protocol, then significant modifications of chemotherapy dose or schedule may be required. Such extreme modifications are likely to be rare and should therefore be discussed on a case-by-case basis with the MRC CTU in the first instance who will liaise with the Chief Investigator where necessary.

Any deviation from the proposed dose modification schedule should be discussed with the MRC CTU/Chief Investigator.

Table 17: Guidelines for Paclitaxel and Carboplatin Dose Modification for Delayed Haematological Recovery – All arms

	**Delayed ANC recovery (>7 days)**[28]	
**Delayed PLT Recovery (>7 days)**[q]	ANC ≥1.0 x 10^9/l[29]	ANC <1.0 x 10^9/l[r]
PLT ≥75 x 10^9/l	**Paclitaxel**:<br>No modification<br><br>**Carboplatin**:<br>No modification	**Paclitaxel**:<br>Either:<br>Use G-CSF and continue current dose[30]<br>**Or**<br>Reduce by 1 dose level (see **Table 16**)<br><br>**Carboplatin**:<br>Use G-CSF and continue current dose<br>**Or**<br>Consider dose reduction by one dose level (see **Table 16**)
PLT <75 x 10^9/l	**Paclitaxel**:<br>No modification<br><br>**Carboplatin**:<br>Reduce by 1 dose level (see **Table 16**)	**Paclitaxel**:<br>Either:<br>Use G-CSF and continue current dose[30]<br>**Or**<br>Reduce by 1 dose level (see **Table 16**)<br><br>**Carboplatin**:<br>Reduce by 1 dose level (see **Table 16**)

[28] If blood counts recover within 7 days (ie. before day 29) then no dose modification is mandated
[29] Use day 22 ANC/PLT count, or a subsequent FBC if lower, if counts are not recovered by day 29
[30] It is recommended that G-CSF prophylaxis is used in preference to dose reduction if feasible in order to maintain planned dose intensity.

Table 18: Guidelines for Paclitaxel and Carboplatin Dose Modification Following the Occurrence of a Haematological Dose Limiting Toxicity (DLT) – all Arms

	**Dose Limiting Toxicity-ANC[31]**	
**Dose Limiting Toxicity-PLT[32]**	**No**	Yes
No	**Paclitaxel:**<br>No modification<br><br>**Carboplatin**:<br>No modification	**Paclitaxel**:<br>Either:<br>Use G-CSF and continue current dose[33]<br>**Or**<br>Reduce by 1 dose level (see **Table 16**)<br><br>**Carboplatin**:<br>Use G-CSF and continue current dose<br>**Or**<br>Consider dose reduction by one dose level (see **table 16**)

[31] This is defined by the occurrence of

- febrile neutropenia (defined as ANC $<1 \times 10^9$/l and a single temperature of $>38.3^{\circ}$C or a sustained temperature of $\geq 38^{\circ}$C for more than one hour (CTCAEv4.0)) or
- prolonged grade 4 neutropenia (defined as ANC $<0.5 \times 10^9$/l persisting ≥7 days)

There are no planned dose modifications for uncomplicated grade 4 neutropenia lasting less than 7 days.

[32] This is defined by the occurrence of

- grade 4 thrombocytopenia (defined as PLT $<25 \times 10^9$/l) or
- bleeding associated with grade 3 thrombocytopenia (PLT 25 – 50 x 10^9/l)

There are no modifications planned for uncomplicated grade 3 thrombocytopenia.

[33] It is recommended that G-CSF prophylaxis is used in preference to dose reduction if feasible in order to maintain planned dose intensity.

Yes	**Paclitaxel**:<br><br>No modification<br><br>**Carboplatin**:<br><br>Reduce by 1 dose level (see **Table 16**)	**Paclitaxel**:<br><br>Either:<br><br>Use G-CSF and continue current dose[33]<br><br>**Or**<br><br>Reduce by 1 dose level (see **Table 16**)<br><br>**Carboplatin**:<br><br>Reduce by 1 dose level (see **Table 16**)

B4.2.2 Non-Haematological Toxicity

B4.2.2.1 Renal Toxicity
The combination of carboplatin and paclitaxel, using the schedules described, is not directly expected to cause renal toxicity. There are, therefore, no specific dose modifications for renal toxicity. The GFR used to calculate the carboplatin dose should, however, be recalculated or re-measured in the event of renal toxicity or changes in the serum creatinine – see **Section B3.4.2: Carboplatin.**

B4.2.2.2 Neuropathy
Grade 2 sensory or motor neuropathy (CTCAE v4.0) requires paclitaxel treatment to be **omitted** until neuropathy has resolved to grade ≤1. On recovery, paclitaxel should be reintroduced but with the dose reduced by 1 dose level (**see Table 16**). If this requires a delay of more than three weeks then the paclitaxel should be omitted from subsequent cycles and treatment continued with carboplatin at the same AUC used in combination with paclitaxel, plus bevacizumab.

Grade ≥3 sensory or motor neuropathy requires paclitaxel to be **omitted** from subsequent cycles, and treatment continued with carboplatin at the same dose as previously used, plus bevacizumab.

B4.2.2.3 Mucositis
For mucositis grade ≥3 (CTCAEv4.0) chemotherapy should be delayed until the mucositis has resolved to grade ≤1. Paclitaxel can be reduced by one dose level (**see Table 16**) in subsequent cycles at the discretion of the treating physician. If the mucositis recurs, or persists for more than three weeks, at grade ≥3 chemotherapy dose modifications should be discussed with the MRC CTU in the first instance who will liaise with the Chief Investigator where necessary.

Mucositis should be treated symptomatically as per standard local practice.

B4.2.2.4 Hypersensitivity
A hypersensitivity reaction to either drug is not expected to be a dose limiting toxicity but the occurrence & management of hypersensitivity reactions is being monitored as part of the toxicity

analysis. Suggested protocols for the management of carboplatin and paclitaxel hypersensitivity can be found in the ICON8B Clinical Management Guidance document.

Paclitaxel

If a hypersensitivity reaction occurs then patients may be retreated with paclitaxel. This will depend on the severity of the reaction and the specific reaction. Retreatment should be managed according to standard local practice or as suggested in the ICON8B Clinical Management Guidance document.

In the case of recurrent hypersensitivity reactions despite adequate premedication, the substitution of docetaxel for paclitaxel is not permitted as there is limited data on the efficacy of this treatment given on a weekly schedule. Treatment should continue with carboplatin alone. In this circumstance the patient should remain in the trial following the same visit schedule for the purposes of follow-up and data analysis (**see Section B3.8: Protocol Treatment Discontinuation**). For data completeness, and to avoid potential bias in the reporting of adverse events between arms, data on alternative first-line treatment will be collected on the trial CRFs.

Carboplatin

A hypersensitivity reaction to carboplatin should be managed according to standard local practice. Patients may be retreated according to standard local practice, including escalations of hypersensitivity prophylaxis, in-patient monitoring, increases in the duration of the infusion and use of formal desensitisation protocols (see the ICON8B Clinical Management Guidance document for a suggested protocol).

If further hypersensitivity prevents the continued administration of carboplatin, substitution of cisplatin for carboplatin can be considered but due to the lack of evidence for the use of cisplatin with paclitaxel in dose-fractionated regimens, this would be considered off protocol treatment for trial purposes. In this circumstance the patient should remain in the trial following the same visit schedule for the purposes of follow-up and data analysis (**see Section B3.8: Protocol Treatment Discontinuation**). For data completeness, and to avoid potential bias in the reporting of adverse events between arms, data on alternative first-line treatment will be collected on the trial CRFs.

B4.2.2.5 Liver Toxicity

Hepatotoxicity is not expected with either chemotherapy drug and other causes of liver enzyme elevation should be actively pursued.

If transaminases become elevated and are <3 x ULN (CTCAE v4.0 grade 1) then treatment can be continued as per protocol without any dose modifications or delays. If transaminases become elevated to 3-5 x ULN (CTCAE v4.0 grade 2) then treatment can continue but dose reductions of paclitaxel may be performed according to local practice at the discretion of the treating physician.

If transaminases become elevated to >5 x ULN (CTCAE v4.0 grade 3) then treatment with paclitaxel should cease until resolution to <3 x ULN (CTCAE v4.0 grade 1).

B4.2.2.6 Other

There are no dose modifications planned for alopecia, nausea, diarrhoea, constipation or venous thromboembolism (including DVT and PE). These side effects should be treated with supportive medical measures. Non-steroidal anti-inflammatory agents can be used prophylactically, or symptomatically, as per local practice for the treatment of paclitaxel-induced arthromyalgia.

For any other adverse event of CTCAE v4.0 grade 4 severity considered at least possibly related to study treatment, the patient should be discontinued from protocol therapy after discussion with the MRC CTU in the first instance who will liaise with the Chief Investigator where necessary.

For any other adverse event of CTCAE v4.0 grade 3 severity considered at least possibly related to study treatment, treatment should be withheld until recovery to grade 1 or less and subsequent treatment should be reduced by one dose level (see **Table 16**).

Any patients who develop malignant effusions while on therapy may have them drained according to local practice, assuming coagulation parameters and platelet counts are adequate.

B4.2.3 Stopping Paclitaxel or Carboplatin for Toxicity: Alternative Regimens

B4.2.3.1 Paclitaxel- Specific Toxicity

If it becomes necessary to discontinue paclitaxel because of toxic effects (eg. neuropathy or hypersensitivity) then it is recommended that patients continue trial treatment with single agent carboplatin. This can be administered at the same AUC used in combination with paclitaxel or 1 AUC unit higher at the investigator's discretion. This information will continue to be collected on the trial CRFs.

Docetaxel, or any other chemotherapeutic agent, may NOT be substituted for paclitaxel in any situation as there are limited data for the efficacy of other platinum-combination regimens used on a weekly schedule.

Alternative combination chemotherapy regimens used in the standard care of patients with ovarian cancer may be used if the treating investigator feels that it is in the patient's best interests to receive combination therapy rather than single agent carboplatin but this will be considered off-protocol treatment for trial purposes. In this circumstance the patient should remain in the trial following the same visit schedule for the purposes of follow-up and data analysis (**see Section B3.8: Protocol Treatment Discontinuation**). For data completeness, and to avoid potential bias in the reporting of adverse events between arms, data on alternative first-line treatment will be collected on the trial CRFs.

B4.2.3.2 Carboplatin-Specific Toxicity

If it becomes necessary to discontinue carboplatin because of toxic effects (eg. hypersensitivity) substitution of cisplatin for carboplatin can be considered but due to the lack of evidence for the use of cisplatin with paclitaxel in dose-fractionated regimens, this would be considered off protocol treatment for trial purposes. If cisplatin is used, a recommended schedule would be 75mg/m^2 3-weekly with paclitaxel dosing left at the investigator's discretion. In this circumstance the patient should remain in the trial following the same visit schedule for the purposes of follow-up and data analysis (**see Section B3.8: Protocol Treatment Discontinuation**). For data completeness, and to avoid

potential bias in the reporting of adverse events between arms, data on alternative first-line treatment will be collected on the trial CRFs.

B4.3 ARM B2: 3-WEEKLY CARBOPLATIN WITH WEEKLY PACLITAXEL / ARM B3: 3-WEEKLY CARBOPLATIN AND BEVACIZUMAB WITH WEEKLY PACLITAXEL

B4.3.1 Haematological Toxicity

Day 1 chemotherapy treatment should be delayed if either of the following occurs in the pre-chemotherapy full blood count:

- ANC is less than 1.0 x 10^9/l
- PLT count is less than 75 x 10^9/l.

FBC should then be repeated at least weekly until haematological recovery has occurred (ANC ≥1.0 x 10^9/l and PLT ≥75 x 10^9/l).

If haematological recovery occurs within 7 days, no dose modification is mandated and dosing is left to the discretion of the individual investigator.

If haematological recovery occurs beyond 7 days, it is suggested that doses of carboplatin and paclitaxel are modified according to the day 22 blood count (or subsequent FBC if lower) according to the criteria in **Table 17**. It is recommended that G-CSF prophylaxis is used in preference to dose reduction if this is feasible in order to maintain planned dose intensity.

Day 8 and 15 administration of weekly paclitaxel alone can proceed if:

- ANC is ≥0.5 x 10^9/l
- PLT count is ≥50 x 10^9/l
- There is no complicating fever, infection or bleeding.

If these parameters are not met, the dose should be omitted and the patient reviewed at the time of their next scheduled dose. No dose delays are allowed. Omitted doses will not be replaced.

Patients who require omission of a weekly paclitaxel dose for a low neutrophil count are likely to go on to have omissions in future cycles too. **Following first omission of a weekly paclitaxel dose for neutropenia**, it is therefore recommended that G-CSF is administered with future cycles to reduce the risk of dose delays, maintain dose-intensity and improve treatment completion.

If more than one paclitaxel dose is omitted in the same cycle or at least one dose is omitted from two consecutive cycles, the dose of both carboplatin and paclitaxel should be reduced by one dose level (**see Table 16**).

Dose limiting toxicities are defined, and dose modifications mandated, in **Table 18**. Please note that no dose delay or reduction is expected for anaemia but it should be managed using supportive measures that will maintain dose intensity.

For patients who receive day 1 with an ANC between 1.0-1.5 x 10^9/l, the use of prophylactic G-CSF support to reduce the risk of neutropenic complications and future dose delays is *strongly* recommended.

If G-CSF is used it can be given in accordance with standard local practice but recommendations for its use are given in the ICON8B trial specific clinical guidance document. If it is used, it can be continued at the local investigator's discretion irrespective of the day 8 or 15 ANC (ie. even if it is >1.0 x 10^9/l) but should not be administered in the 48hrs immediately prior to or in the 24 hours following chemotherapy, ie. no G-CSF should be administered on days 6-9 and days 13-16 of each treatment cycle (for further details refer to the ICON8B trial specific clinical guidance document).

Patients who fail to recover adequate counts after a delay of 2 weeks or more to day 1, or who have consecutive dose limiting toxicities, are unlikely to be able to tolerate standard doses of carboplatin and paclitaxel. If it is considered in the patient's best interest to remain within the trial and to continue to receive treatment according to this protocol, then significant modifications of chemotherapy dose or schedule may be required. Such extreme modifications are likely to be rare and should therefore be discussed on a case-by-case basis with the MRC CTU in the first instance who will liaise with the Chief Investigator where necessary. Any deviation from the proposed dose modification schedule should be discussed with the MRC CTU/Chief Investigator.

B4.3.2 Non-haematological Toxicity

B4.3.2.1 Renal Toxicity

The combination of carboplatin and paclitaxel, using the schedules described, is not directly expected to cause renal toxicity. There are, therefore, no specific dose modifications for renal toxicity. The GFR used to calculate the carboplatin dose should, however, be re-calculated or re-measured in the event of renal toxicity or changes in the serum creatinine – see **Section B3.4.2: Carboplatin.**

B4.3.2.1 Neuropathy

Grade 2 sensory or motor neuropathy (CTCAEv4.0) requires paclitaxel treatment to be **omitted** until neuropathy has resolved to grade ≤1. On recovery, paclitaxel should be reintroduced but with the dose reduced by 1 dose level (see **Table 16**). If this requires a delay of more than three weeks then the paclitaxel should be omitted from subsequent cycles and treatment continued with single agent carboplatin (or in the case of arm B3 patients, carboplatin and bevacizumab alone) at the same AUC used in combination with paclitaxel.

Grade ≥3 sensory or motor neuropathy requires paclitaxel to be omitted from subsequent cycles, and treatment continued with single agent carboplatin at the same dose as previously used (or in the case of arm B3 patients, carboplatin and bevacizumab alone).

B4.3.2.3 Mucositis

For mucositis grade ≥3 (CTCAEv4.0) chemotherapy should be delayed until the mucositis has resolved to grade ≤1. Paclitaxel can be reduced by one dose level (see **Table 16**) in subsequent cycles at the discretion of the treating physician. If the mucositis recurs, or persists for more than three weeks, at grade ≥3 chemotherapy dose modifications should be discussed with the MRC CTU in the first instance who will liaise with the Chief Investigator where necessary.

Mucositis should be treated symptomatically as per standard local practice.

B4.3.2.4 Hypersensitivity

A hypersensitivity reaction to either drug is not expected to be a dose limiting toxicity but the occurrence & management of hypersensitivity reactions is being monitored as part of the toxicity analysis. Hypersensitivity events should be recorded on either the Chemotherapy CRF or Bevacizumab Maintenance CRF, depending on which treatment phase the patient is in. Suggested protocols for the

management of carboplatin and paclitaxel hypersensitivity can be found in the ICON8B trial specific clinical guidance document.

Paclitaxel

If a hypersensitivity reaction occurs then patients may be retreated with paclitaxel. This will depend on the severity of the reaction and the specific reaction. Retreatment should be managed according to standard local practice or as suggested in the ICON8B trial specific clinical guidance document.

In the case of recurrent hypersensitivity reactions despite adequate premedication, the substitution of docetaxel for paclitaxel is not permitted as there are limited data on the efficacy of this treatment given on a weekly schedule. In this circumstance the patient should remain in the trial following the same visit schedule for the purposes of follow-up and data analysis (**see Section B3.8: Protocol Treatment Discontinuation**). For data completeness, and to avoid potential bias in the reporting of adverse events between arms, data on alternative first-line treatment will be collected on the trial CRFs.

Carboplatin

A hypersensitivity reaction to carboplatin should be managed according to standard local practice. Patients may be retreated according to standard local practice, including escalations of hypersensitivity prophylaxis, in-patient monitoring, increases in the duration of the infusion and use of formal desensitisation protocols (see ICON8B trial specific clinical guidance document for a suggested protocol).

If further hypersensitivity prevents the continued administration of carboplatin, substitution of cisplatin for carboplatin can be considered but due to the lack of evidence for the use of cisplatin with paclitaxel in dose-fractionated regimens, this would be considered off protocol treatment for trial purposes. In this circumstance the patient should remain in the trial following the same visit schedule for the purposes of follow-up and data analysis (**see Section B3.8: Protocol Treatment Discontinuation**). For data completeness, and to avoid potential bias in the reporting of adverse events between arms, data on alternative first-line treatment will be collected on the trial CRFs.

B4.3.2.5 Liver Toxicity

Hepatotoxicity is not expected with either chemotherapy drug and other causes of liver enzyme elevation should be actively pursued.

If transaminases become elevated and are <3 x ULN (CTCAE v4.0 grade 1) then treatment can be continued as per protocol without any dose modifications or delays. If transaminases become elevated to 3-5 x ULN (CTCAE v4.0 grade 2) then treatment can continue but dose reductions of paclitaxel may be performed according to local practice at the discretion of the treating physician.

If transaminases become elevated to >5 x ULN (CTCAE v4.0 grade 3) then treatment with paclitaxel should cease until resolution to <3 x ULN (CTCAE v4.0 grade 1).

B4.3.2.6 Rash Associated with Weekly Dose-Dense Paclitaxel

During ICON8, it has become apparent that some patients receiving weekly dose-dense paclitaxel have developed a skin rash on treatment. This most typically affects the dorsal surfaces of the hand/ forearm and is predominantly erythematous in nature. Associated skin peeling and occasionally blistering alongside pain, soreness and itching have been reported. Supportive management such as emollients, analgesia and antihistamines are recommended at investigators discretion.

It is recommended that if the rash is ≥ CTCAE grade 2 then weekly paclitaxel is omitted until recovery to ≤ CTCAE grade 1 and that subsequently the dose of weekly paclitaxel is reduced by one dose level (see **Table 16**).

B4.3.2.7 Other

There are no dose modifications planned for alopecia, nausea, diarrhoea, constipation or venous thromboembolism (including DVT and PE). These side effects should be treated with supportive medical measures. Non-steroidal anti-inflammatory agents can be used prophylactically, or symptomatically, as per local practice for the treatment of paclitaxel-induced arthromyalgia.

For any other adverse event of CTCAE v4.0 grade 4 severity considered at least possibly related to study treatment, the patient should be discontinued from protocol therapy after discussion with the MRC CTU in the first instance who will liaise with the Chief Investigator where necessary.

For any other adverse event of CTCAE v4.0 grade 3 severity considered at least possibly related to study treatment, treatment should be withheld until recovery to grade 1 or less and subsequent treatment should be reduced by one dose level (see **Table 16**).

Any patients who develop malignant effusions while on therapy may have them drained according to local practice, assuming coagulation parameters and platelet counts are adequate.

B4.3.3 STOPPING PACLITAXEL OR CARBOPLATIN FOR TOXICITY: ALTERNATIVE REGIMENS

B4.3.3.1 Paclitaxel-Specific Toxicity

If it becomes necessary to discontinue paclitaxel because of toxic effects (eg. neuropathy or hypersensitivity) then it is recommended that patients continue trial treatment with single agent carboplatin. This can be administered at the same AUC used in combination with paclitaxel or 1 AUC unit higher at the investigator's discretion. This information will continue to be collected on the trial CRFs.

Docetaxel, or any other chemotherapeutic agent, may NOT be substituted for paclitaxel in any situation as there are limited data for the efficacy of other platinum-combination regimens used on a weekly schedule.

Alternative combination chemotherapy regimens used in the standard care of patients with ovarian cancer may be used if the treating investigator feels that it is in the patient's best interests to receive combination therapy rather than single agent carboplatin but this will be considered off-protocol treatment for trial purposes. In this circumstance the patient should remain in the trial following the

same visit schedule for the purposes of follow-up and data analysis (**see Section B3.8: Protocol Treatment Discontinuation**). For data completeness, and to avoid potential bias in the reporting of adverse events between arms, data on alternative first-line treatment will be collected on the trial CRFs.

B4.3.3.2 Carboplatin-Specific Toxicity

If it becomes necessary to discontinue carboplatin because of toxic effects (eg. hypersensitivity) substitution of cisplatin for carboplatin can be considered but due to the lack of evidence for the use of cisplatin with paclitaxel in dose-fractionated regimens, this would be considered off protocol treatment for trial purposes. If cisplatin is used, a recommended schedule would be 75mg/m^2 3-weekly with paclitaxel dosing left at the investigator's discretion. In this circumstance the patient should remain in the trial following the same visit schedule for the purposes of follow-up and data analysis (**see Section B3.8: Protocol Treatment Discontinuation**). For data completeness, and to avoid potential bias in the reporting of adverse events between arms, data on alternative first-line treatment will be collected on the trial CRFs.

B4.4 BEVACIZUMAB MODIFICATIONS: DELAYS AND OMISSIONS DUE TO ADVERSE EVENTS (ARMS B1 AND B3)

B4.4.1 Bevacizumab: General Principles

Situations where administration of bevacizumab should be delayed, omitted or permanently discontinued are available in **section B4.4.2**. The following general principles should be followed:

- During the chemotherapy phase: Where it is not safe to proceed with bevacizumab, the administration of bevacizumab should be omitted from that cycle and treatment can proceed with carboplatin and paclitaxel

- In the situation where bevacizumab is omitted for **2 consecutive cycles (6 weeks) bevacizumab should not be restarted** unless discussed with the ICON8B trial physician or chief investigator: mrcctu.icon8b@ucl.ac.uk. The only exception to this is when bevacizumab

is omitted from cycles 3 and 4 to facilitate delayed primary surgery as detailed in section B3.3.3: Modifications of SACT around Surgery.

During the maintenance phase:

- Where it is not safe to proceed with bevacizumab the administration of bevacizumab should be delayed and the patient should be evaluated at weekly intervals until adequate recovery has occurred
- Where a **delay exceeds 30 days**, bevacizumab should be permanently discontinued
- Where fewer than 6 doses of bevacizumab are administered with chemotherapy, additional maintenance cycles should be given to complete a total course of 18 cycles.

Please note there are **no dose modifications for bevacizumab** and doses should only be re-calculated for patients who experience a >10% change in body weight as compared to baseline.

B4.4.2 Bevacizumab Notable Events (DPS Patients Only)

This section will detail the management of the most common and severe side effects observed in patients receiving bevacizumab, with or without chemotherapy.

The events listed below have been designated as **notable adverse events** in this trial for **DPS patients ONLY** and should be reported on an **SAE form** regardless of whether they fulfil the standard definitions of serious (given in Section 3: Safety Reporting).

Please see section 3.2: ICON8B Specific Notable Events for detailed instructions on reporting ICON8B notable events.

ICON8B Notable Adverse Events for DPS Patients ONLY

- Grade 3 and grade 4 hypertension
- Grade 3 proteinuria/nephrotic syndrome
- Grade 3 and grade 4 venous thromboembolic events including pulmonary embolism
- Any arterial thromboembolic events, including pulmonary embolism

Report all new cases that occur up to the week 66 follow-up visit.
OR

If a patient is still receiving trial treatment after week 66 then these events should continue to be reported until 30 days after the last protocol treatment

- Grade 3 or grade 4 haemorrhagic (bleeding) events
- All grades of post operative wound healing complications or delayed wound healing
- All grades of gastro-intestinal perforation
- All grades of fistulae.

B4.4.3 Specific Toxicities Requiring Permanent Discontinuation of Bevacizumab

Any patient who develops any one of the following toxicities attributable to bevacizumab **should not receive further bevacizumab.** For situations where bevacizumab should be withheld pending further action see section B4.4.4: SPECIFIC ADVERSE EVENTS OF BEVACIZUMAB AND RECOMMENDATIONS FOR TREATMENT

- Grade 4 hypertension
- Nephrotic Syndrome
- Grade 4 intestinal obstruction
- Wound dehiscence requiring medical therapy
- Any grade of necrotising fasciitis
- Any grade of fistula **(unless the criteria listed in section B4.4.4.5: Fistulae are met)**
- Any grade of gastro-intestinal perforation
- Grade 3/4 bleeding **(except if occuring in relation to DPS, please see section B4.4.4.7: Haemorrhage)**
- Grade 3/4 congestive heart failure or left ventricular dysfunction (symptomatic heart failure, LVEF <40%)
- Any grade of arterial thromboembolic events (including transient ischaemic attack (TIA), cerebrovascular accident (CVA) and myocardial infarction (MI) or new diagnosis of ischaemic heart disease during study treatment)
- Any grade of osteonecrosis of the jaw
- Any grade of Posterior Reversible Encophalopathy Syndrome (PRES).

B4.4.4 Specific Adverse Events of Bevacizumab and Recommendations for Treatment

B4.4.4.1 Hypertension

Where a patient is found to have a persistently elevated systolic blood pressure of >150mmHg and/or a diastolic blood pressure of >100mmHg, bevacizumab should be withheld until blood pressure becomes controlled (<150/100 mmHg on 2 occasions at least 1 week apart). The ICON8B hypertension GP letter should be sent to the patient's GP to advise on monitoring and control of hypertension. The patient should be given the ICON8B blood pressure monitoring form to complete during periods when BP is elevated.

Where a patient develops CTCAE grade 4 hypertension (life threatening consequence e.g. hypertensive crisis or hypertensive encephalopathy) bevacizumab should be permanently discontinued and should be treated as a medical emergency.

Blood pressure measurements should be taken after the patient has been in a resting position for ≥5 minutes. Where the initial reading is above 140 mmHg systolic and/or 90 mmHg diastolic blood pressure this should be confirmed with a repeat measurement at least a 1 hour interval.

Please see the ICON8B Clinical Management Guidance document in the Investigator Site File for further guidance on the management of hypertension in arms B1 and B3.

B4.4.4.2 Proteinuria

Patients treated with bevacizumab are at increased risk of proteinuria, particularly with a history of hypertension.

- Where urine dipstick shows <2+proteinuria, bevacizumab can be administered as planned
- Where urine dipstick shows ≥2+ proteinuria, follow algorithm in **Figure 12**
- Where nephrotic syndrome occurs then bevacizumab should be permanently discontinued.

Figure 12: Algorithm for Proteinuria (dipstick) ≥ 2+

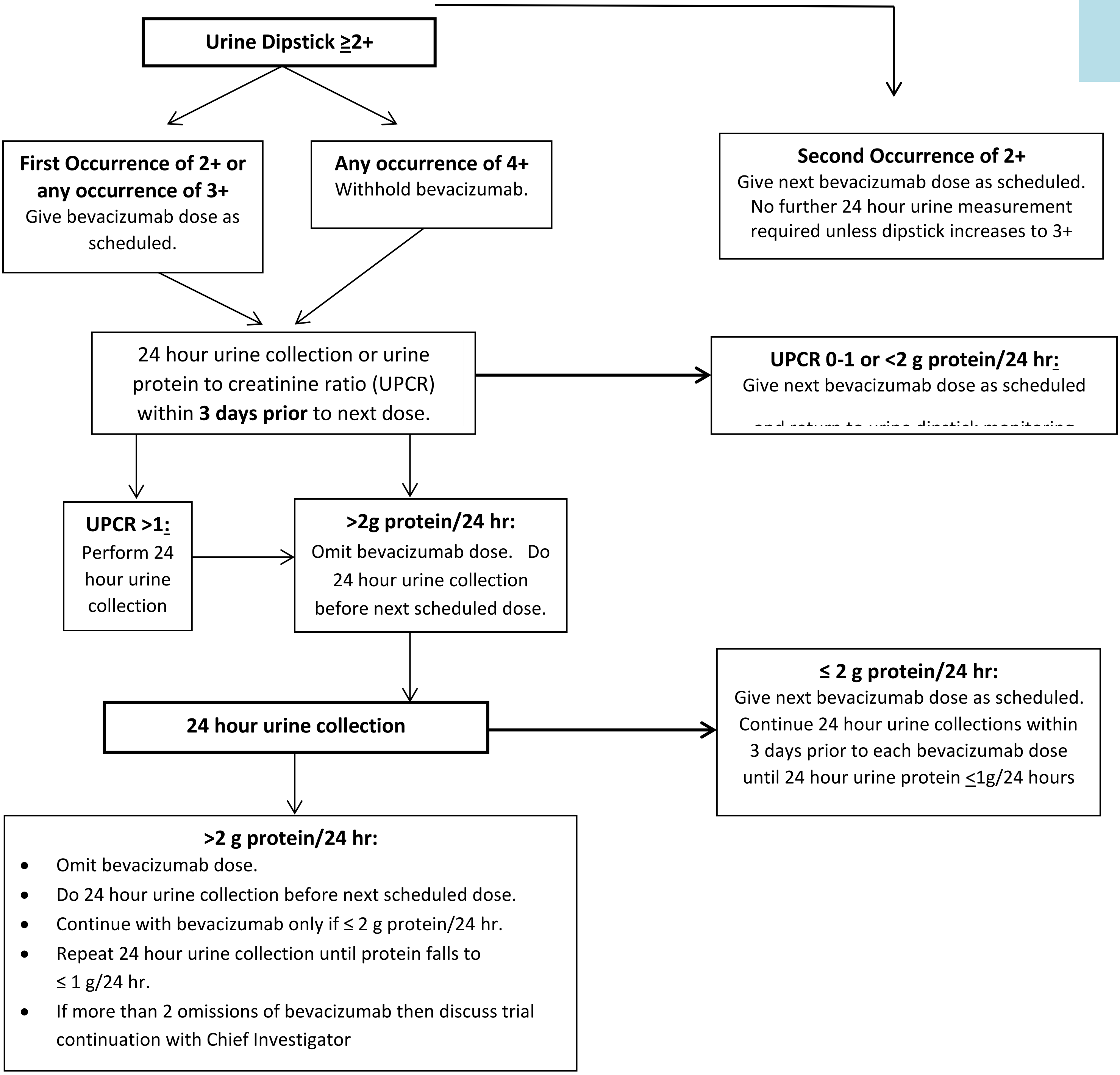

B4.4.4.3 Intestinal Obstruction (including subacute obstruction)

Where CTCAE grade 1 intestinal obstruction develops bevacizumab should be used with caution and only after careful clinical assessment. Where intestinal obstruction develops ≥CTCAE grade 2, bevacizumab should be withheld until intestinal obstruction improves to ≤CTCAE grade 1.

Where CTCAE grade 4 intestinal obstruction develops bevacizumab should be permanently discontinued. Investigators should take appropriate steps to assess for disease progression should intestinal obstruction occur.

B4.4.4.4 Impaired Wound Healing:

Bevacizumab may adversely affect wound healing and and should be omitted until four weeks has passed from any surgical intervention, including surgical interventions for non-cancer indications.

As a precautionary measure, it is recommended that an interval of 7 days is left between any central venous access devices (CVAD) and the onset of bevacizumab being given. Where this is not feasible then bevacizumab can be started earlier, according to standard local practice, but not less than two days after the placement of CVAD.The status of the wound must be checked before treatment commences to ensure healing is occuring.

Any patients experiencing significant wound healing complications (≥CTCAE grade 2) whilst receiving bevacizumab should have bevacizumab withheld until the wound is fully healed. Where any wound dehiscence requiring medical therapy occurs bevacizumab should be permanently discontinued.
Non-emergency surgery should be delayed if possible until at least 28 days after the last dose of bevacizumab. For patients requiring unplanned surgery, bevacizumab should be discontinued once a decision to operate has been taken. Surgeons should be aware that there is an increased risk of impaired wound healing and careful consideration should be given to the benefits and risks of any potential operation. Bevacizumab should be discontinued in patients who develop necrotising fasciitis, and appropriate treatment should be promptly initiated.

B4.4.4.5 Fistulae

Where any CTCAE grade of fistula develops bevacizumab should be permanently discontinued unless the following criteria are met:

- The fistula is deemed to be unrelated to bevacizumab and to be related to surgery or the disease process itself
- The fistula has resolved completely
- Continuation of, restarting or commencing bevacizumab has been agreed by the Chief Investigator.

B4.4.4.6 Gastrointestinal Perforation:

Bevacizumab should be permanently discontinued in patients who develop gastrointestinal perforation. Patients particularly at risk are those with intra-abdominal inflammation e.g. gastric ulcer, inflammatory bowel disease or diverticulitis and those with recent active symptoms from these conditions are excluded from entering ICON8B.

Patients need to be carefully monitored for this potential complication during treatment and made aware of this risk so that they know to seek medical attention if any suggestive symptoms occur. Any cases of gastrointestinal perforation should be managed aggressively, with the involvement of the gastrointestinal or gynaecological oncology surgical teams in the usual manner. The surgical team must be informed that bevacizumab may potentially compromise wound healing.

B4.4.4.7 Haemorrhage:

Patients treated with bevacizumab have an increased risk of haemorrhage, especially tumour-associated haemorrhage and also minor mucocutaneous haemorrhage (e.g. epistaxis).

If CTCAE grade 3/4 bleeding occurs appropriate treatment should be instituted and bevacizumab treatment will be discontinued permanently. An exception to this is blood transfusions that occur after DPS without exceptional blood loss which will continue to be classified as CTCAE grade 3/4 haemorrhage, however bevacizumab need not be discontinued in these circumstances.

B4.4.4.8 Risk of Cardiovascular Toxicity:

An increased risk of cardiovascular toxicity has been reported in some clinical trials of bevacizumab.

Bevacizumab should be permanently discontinued in patients who develop myocardial infarction, cerebrovascular disease, or CTCAE grade 3/4 congestive heart failure, or left ventricular dysfunction (symptomatic heart failure, LVEF <40%). Patients diagnosed with new ischaemic heart disease during the study should also permanently discontinue bevacizumab.

B4.4.4.9 Thrombosis/Embolism:

The incidence of arterial and venous thromboembolic events is increased by bevacizumab. Patients who develop the following CTCAE grades of thrombosis /embolism must discontinue bevacizumab and the following action is recommended:

- CTCAE grade 3 venous thromboembolic events; withhold bevacizumab upon diagnosis.
- Bevacizumab may be resumed after initiation of therapeutic-dose anticoagulant therapy if all of the following criteria are met:

  (i) The patient should preferably be treated with low molecular weight heparin

  (ii) The patient must not have had a CTCAE grade 3 or 4 haemorrhagic event since entering the study

  (iii) Patient has been anti-coagulated and has become asymptomatic from a previous symptomatic PE.
- Bevacizumab should be permanently discontinued in patients who develop any CTCAE grade of arterial thromboembolic event (e.g. cerebrovascular accidents, transient ischaemic attacks, myocardial infarctions)

- Bevacizumab should be permanently discontinued in patients with any life-threatening (CTCAE grade 4) thromboembolic events.

B4.4.4.10 Osteonecrosis of the Jaw (ONJ)

Bevacizumab should be permanently discontinued if osteonecrosis of the jaw develops. Caution should be exercised when bevacizumab and intravenous bisphosphonates are administered simultaneously or sequentially.

B4.4.4.11 Posterior Reversible Encophalopathy Syndrome (PRES)

There have been rare reports of bevacizumab-treated patients developing signs and symptoms that are consistent with PRES, a rare neurologic disorder, which can present with the following signs and symptoms among others: seizures, headache, altered mental status, visual disturbance, or cortical blindness, with or without associated hypertension. A diagnosis of PRES requires confirmation by brain imaging, preferably magnetic resonance imaging (MRI). In patients developing PRES, bevacizumab should be permanently discontinued.

B4.4.4.12 Infusion and hypersensitivity reactions

Patients may be at risk of developing infusion/hypersensitivity reactions to bevacizumab. If a reaction occurs, the infusion should be discontinued and appropriate medical therapies should be administered. A systematic premedication is not warranted. For further information please refer to the ICON8B Clinical Management Guidance document.

B5 ASSESSMENTS & PROCEDURES

All assessments and trial procedures must be performed in compliance with the most up to date version of the protocol, the principles of Good Clinical Practice, any relevant research governance and other regulatory requirements as appropriate.

B5.1 VISIT SCHEDULE

Summary information on the timing of interventions, assessments for safety and efficacy, and trial visits are given in **Section B6: Trial Assessments Schedule for ICON8B**, **Tables 19-24**. Separate tables have been prepared for each arm depending on whether participants have had IPS or will be having primary chemotherapy (with or without DPS).

B5.1.1 Chemotherapy phase visits

- During carboplatin + paclitaxel, patients should be seen 3-weekly prior to administration of day 1 of each chemotherapy cycle
- Chemotherapy phase visits should usually occur within 72 hours prior to day 1
- The end of chemotherapy visit should occur 3 weeks (+/- 1 week) after the start of the final cycle of carboplatin + paclitaxel
- If a patient stops trial chemotherapy early (e.g. for toxicity) but continues with non-trial 1st line chemotherapy, the end of chemotherapy visit should occur 3 weeks (+/-1 week) after day 1 of the last cycle of **non-trial chemotherapy.**

B5.1.2 Maintenance phase visits (Arms B1 and B3)

- The 1st maintenance visit (which is expected to coincide with the end of chemotherapy visit), is the 1st administration of maintenance bevacizumab
- During maintenance bevacizumab, patients should have safety assessments performed prior to **every** bevacizumab administration (3-weekly) as defined in section **B5.4.2.**
- A clinical review for trial purposes including additional assessments (as detailed in section **B5.4.2**) is required **prior to the first bevacizumab maintenance treatment administration**, and then prior to **every other cycle of bevacizumab thereafter.**
- The End of bevacizumab maintenance visit should be conducted 3 weeks (+/-1 week) after the final cycle of bevacizumab.
  NB. If the end of bevacizumab visit is due within 6 weeks of the week 66 visit then the end of bevacizumab visit may be omitted.
- If patients stop bevacizumab early, 6-weekly follow-up should continue until week 66 post-randomisation.

B5.1.3 Follow-up phase

- All patients should have follow-up visits:
  - 6-weekly (+/-1 week) from the end of chemotherapy visit to week 66 post-randomisation. In participants on maintenance bevacizumab, these are expected to coincide with the regular maintenance phase visits
  - 3-monthly from week 66 until the end of 2 years post-randomisation
  - 6-monthly in years 3, 4, 5 and 6
  - Annually thereafter until trial closure.

After protocol defined progression, follow-up data will be collected 6-monthly. Information on subsequent anti-cancer therapy will continue to be collected after progression.

Once a participant has been randomised, follow-up information and appropriate CRFs (see ICON8B CRF Completion Guidelines) should be completed even if they do not remain on the trial treatments (unless the participant requests to withdraw from follow-up, see **section B5.7: Early Stopping of Follow-up**). Full documentation of cancer treatments and follow-up will be required to ensure data completeness for the intention-to-treat analyses.

B5.2 PROCEDURES FOR ASSESSING EFFICACY

Progression definitions based on RECIST v1.1 and clinical criteria are given in **Appendix 8**.

B5.2.1 Tumour Imaging Assessments

Imaging assessments will be performed as specified in **Section B6: Trial Assessments Schedule for ICON8B**, **Tables 19-24**.

Tumour assessments require a CT scan of the pelvis and abdomen, and should also include any other known tumour sites. A CT scan is the preferred method of evaluation but MRI can be used. The same assessment technique should be used throughout the trial to allow comparison between scans. Use of PET-CT is NOT permitted for protocol mandated tumour assessments.

B5.2.1.1 Chemotherapy phase imaging

- For patients who underwent IPS, imaging after three cycles of chemotherapy is not required unless clinically indicated
- For patients in whom DPS or IDS is planned,
    - Mid-point imaging should be performed pre-operatively for surgical planning according to local practice, generally during cycle 3
    - A further CT scan should be performed 4 weeks +/-1 week after surgery which will be the baseline assessment for documentation of future progression
- All patients must have an end of chemotherapy CT scan which should take place 3 weeks (+/- 1 week) after day 1 of the final cycle of first-line chemotherapy.

B5.2.1.2 Maintenance phase imaging (Arms B1 and B3)

- Imaging during maintenance bevacizumab is not required unless clinically indicated or the participant has CA125 progression by GCIG criteria (see **Appendix 8**).

B5.2.1.3 Follow-up phase imaging

- All patients should have a CT scan at week 66 (+/- 2 weeks) post-randomisation
- At subsequent follow-up visits, tumour assessments should only be performed if clinical symptoms are suggestive of recurrence or there is CA125 progression as defined by GCIG criteria (see **Appendix 8**). Radiological tumour assessment should then occur within 3 weeks of the date of suspected clinical or CA125 progression. If there is CA125 progression but no radiological documentation of disease progression then imaging should be repeated 3-monthly until protocol defined progression occurs.

B5.2.2 CA125

Serum CA125 will not be used to define progression but does form part of the assessment procedures. It should be assessed by the same laboratory throughout the trial period and centres are required to submit the normal ranges for CA125 for the laboratory used.

CA125 should be assessed:

- At baseline, within a 14 day period prior to the first cycle of chemotherapy
- At each chemotherapy phase visit
- At every other bevacizumab maintenance phase visit
- At each follow-up visit until disease progression. Following first progression CA125 is not mandatory but should be recorded on the Follow-Up CRF if it has been performed as part of standard practice.

During follow-up, if there is CA125 progression by GCIG criteria (see **Appendix 8**) a CT scan should be performed and repeated 3-monthly until protocol defined progression occurs.

B5.3 PROCEDURES FOR ASSESSING SAFETY

Safety assessments will be performed as specified in **Section B6: Trial Assessments Schedule for ICON8B**, **Tables 19-24**.

Details on reporting of Serious Adverse Events are given in **Section 3: Safety Reporting**.

B5.3.1 CHEMOTHERAPY PHASE

- Clinical assessment and pre-treatment investigations should be performed within 72 hours prior to day 1 of each chemotherapy cycle
- All patients:
    - Assessment of any adverse events that occurred in the preceding chemotherapy cycle
    - General physical exam directed by the physician including an assessment of weight. This can be performed by a nurse if they hold a nurse clinician qualification or equivalent training to be able to safely perform a physical exam. They must be appropriately delegated to perform this task by the PI on the delegation log.
    - Blood tests including:
        - Standard haematology (Hb, WBC, ANC, PLT) and
        - Standard biochemistry (to include at least creatinine, albumin, ALT (SGPT) or AST (SGOT) and bilirubin)
        - CA125
- Patients on Arms B1 and B3 only (i.e. receiving bevacizumab):
    - Blood pressure
    - Urinalysis for proteinuria
    - 24-hour urine protein if indicated (see **Section B4.4.4.2 Proteinuria**)
- Patients on Arms B2 and B3 only (i.e. having weekly chemotherapy)
    - Standard haematology blood tests (Hb. WBC, ANC, PLT) prior to days 8 and 15 of each chemotherapy cycle

- In patients undergoing DPS:
    - Pre-operative safety assessments and investigations should be performed according to standard practice at trial sites
    - Assessment of post-operative adverse events should be performed at the next chemotherapy visit and recorded on the **Surgery CRF.**

B5.4.2 MAINTENANCE PHASE (ARMS B1 AND B3)

- The following tests should be performed prior to every administration of bevacizumab, in accordance with standard practice
    - Blood pressure
    - Urinalysis for proteinuria
    - 24-hour urine protein if indicated (see **Section B4.4.4.2 Proteinuria**).

- In addition, the following clinical assessments should be performed 6-weekly during the maintenance phase, starting prior to the first bevacizumab maintenance administration and then prior to day 1 of **every other** bevacizumab cycle. The following clinical assessments and pre-treatment investigations should be performed within 5 days prior to day 1 of every other bevacizumab maintenance administration:
    - Assessment of any adverse events that occurred in the preceding 6 week period[34]
    - Physical examination including an assessment of weight
    - Blood tests: standard haematology (Hb, WBC, ANC, PLT)
    - ECOG
    - Blood pressure
    - Urinalysis for proteinuria
    - 24-hour urine protein if indicated (see **Section B4.4.4.2 Proteinuria**)
    - CA125 blood test

B5.4 PROCEDURES FOR ASSESSING QUALITY OF LIFE

The impact of the three trial treatment strategies on quality of life (QoL) is a secondary end-point of ICON8B.

Patient reported QoL will be assessed using cancer-specific questionnaires which are included in the **Quality of Life CRFs**. The questionnaires used are the EORTC QLQ OV-28, which incorporates the EORTC QLQ C-30,[43] and the EQ-5D[44].

B5.4.1 TIMING OF QUALITY OF LIFE ASSESSMENTS

- **Baseline:** A baseline QoL form needs to be completed by the patient at her screening visit, after consent to participate has been given and prior to randomisation.
- **Chemotherapy phase**: A QoL form should be completed at the start of each chemotherapy cycle from cycle 2 to cycle 6 (up to 72 hours before).
- **End of Chemotherapy**: A QoL form should be completed at the End of Chemotherapy visit (3 weeks +/- 1 week after the start of the final cycle of carboplatin + paclitaxel (+bevacizumab).

[34] To allow comparison of bevacizumab toxicity across all trial arms, data on a limited set of adverse events will also be collected at 6-weekly follow-up visits in Arm B2.

- **End of Bevacizumab**: A QoL form should be completed at the End of Bevacizumab visit (3 weeks +/- 1 week after the start of the final cycle of bevacizumab).
- **Week 66**: A QoL should be completed at the week 66 visit (week 66 post randomisation).
- **Follow-up phase:**
    - A QoL form should be completed at week 102, month 36, month 48, month 60 and month 72 follow-up visits (up to the end of 6 years after randomisation)
    - Patients who progress should continue to complete QoL forms at the trial specified follow-up visits (6-monthly), where at all possible.

B5.4.2 Good practice guidance for completion of the Quality of Life questionnaires

- The quality of life form should be given to the patient before medical assessments are performed or chemotherapy is administered
- The questionnaires should be completed without conferring with friends, relatives or health professionals and all questions should be answered even if the patient feels them to be irrelevant
- Patients should complete the QoL form on their own, whilst waiting to be seen in the clinic prior to medical assessment or intervention, and in a quiet area if possible. The form should not be taken away to be completed at home.
- The clinician or nurse in charge of the patient should collect the form before the patient leaves and should be available to answer questions on the form if the patient wishes, but should not assist with its completion. The form should be checked to ensure that the dates of completion and patient identifiers are correct
- The patient should be offered an envelope in which the questionnaire can be sealed if they wish to keep it confidential.

B5.5 HEALTH ECONOMICS ASSESSMENT

A cost-effectiveness analysis of the three trial treatment strategies is a secondary outcome measure of ICON8B.

Cost-effectiveness will be assessed using the EQ-5D questionnaire and an additional UK specific Medical Resource Utilisation questionnaire. These questionnaires are included in the Quality of Life CRF and are completed at the same time points as the QoL assessments.

There are also additional questions on the Chemotherapy, Maintenance and Follow-up CRFs, which should be completed by site staff.

B5.6 TRANSLATIONAL RESEARCH SAMPLES

Samples for translational research (TRICON8B) will only be collected from patients in participating centres who have consented separately to participate in biological studies. Further details can be found in **Appendix 12**; and for detailed information on the processing, labelling, handling, storage and shipment of these specimens please refer to the TRICON8B Manual.

In all patients participating in the translational component of ICON8B, formalin-fixed paraffin-embedded (FFPE) tumour samples are required. This is a baseline surgical specimen from IPS patients. From DPS patients, FFPE samples are required from the baseline diagnostic biopsy and at surgery. At centres who are participating at level 2 of the TRICON8B programme, an additional blood sample for

DNA analysis is required at baseline. At centres who are participating at level 3 of the TRICON8B programme additional blood samples will be taken at up to 26 time points as detailed in **Sections B6.7, B6.8** and the TRICON8B manual.

B5.7 EARLY STOPPING OF FOLLOW-UP

If a patient chooses to discontinue their trial treatment, they should always be followed up providing they are willing, that is, they should be encouraged to not leave the whole trial. If they do not wish to remain on the trial follow-up schedule, data to allow the primary outcome measures (progression-free and overall survival) should still be recorded where possible. Only if the patient states their wish for no further data to be collected should they be withdrawn from the trial completely. The MRC CTU should be informed of this in writing using the appropriate documentation.

If the medical data collected during the patient's participation in the trial are kept for research and analysis purposes, they can be anonymised if necessary. Consent for future use of stored samples already collected can be refused when leaving the trial early (but this should be discouraged and should follow a discussion).

Patients may change their minds about stopping trial follow-up at any time and re-consent to participation in the trial.

Patients who stop trial follow-up early will not be replaced.

Patients will be followed up in the long-term through usual mechanisms, which may include flagging with the NHS Information Centre, or similar approaches.

B5.8 PATIENT TRANSFERS

For patients moving from the area, every effort should be made for the patient to be followed-up at another participating trial centre and for this trial centre to take over responsibility for the patient, or for follow-up to continue via other health care professionals, eg. General Practitioner. Details of other participating GCIG groups and clinical centres can be obtained from the MRC CTU. The consent of patients should be obtained for their names to be flagged for survival information through national registries. If the investigator moves then appropriate arrangements should be made to arrange for trial follow-up to continue at the centre.

A copy of the patient's CRFs will need to be provided to the new site and all due CRFs up until that time point and data queries must be resolved. The patient will have to sign a new consent form at the new site, and until this occurs, the patient remains the responsibility of the original centre.

B5.9 LOSS TO FOLLOW-UP

Every effort should be made to follow-up patients who have been randomised. Patients should, if possible, remain under the care of an oncologist or gynaecologist for the duration of the trial. If the care of a patient is returned to the General Practitioner, it is still the responsibility of the investigator to ensure that the follow-up data required by the protocol are collected and reported.

B6 TRIAL ASSESSMENTS SCHEDULE FOR ICON8B

B6.1 TABLE 19: TRIAL ASSESSMENTS SCHEDULE FOR IPS PATIENTS ICON8B ARM B1: 3-WEEKLY CARBOPLATIN + PACLITAXEL + BEVACIZUMAB

	Screening	Chemotherapy phase						Maintenance phase			Follow-Up phase			
**Assessments**	**Within 28 days of randomisation (unless specified in footnotes)**	**Cycle 1**	**Cycle 2**	**Cycle 3**	**Cycle 4**	**Cycle 5**	**Cycle 6**	**1st maintenance visit/end of chemotherapy visit**	**Subsequent 6-weekly visit**	**End of bevacizumab visit**	**6-weekly follow-up visits (after end of bevacizumab)**	**Week 66 visit**	**Subsequent follow-up visits**	**At progression**
**Cycle Day**		**1**	**1**	**1**	**1**	**1**	**1**							
**Informed consent**	X													
**Disease confirmation (histology)**	X													
**Demographics**	X													
**Medical history**	X													
**Physical examination**	X	X[a]	X	X	X	X	X	X[o]	X[r]	X[p]				
**Dental Examination**	X[s]													
**Blood pressure [d]**	X[d]	X	X	X	X	X	X	X[o]	X[r]	X[p]				
**Urine protein dipstick [d]**	X[d]	X	X	X	X	X	X	X[o]	X[r]	X[p]				
**Height**	X													
**Weight[d]**	X	X[a]	X	X	X	X	X	X[o]	X[r]					
**ECG[b]**	X													
**Performance Status**	X	X[a]	X	X	X	X	X	X[o]	X[r]	X[p]	X	X	X	X

Assessments	Screening	Chemotherapy phase						Maintenance phase			Follow-Up phase			
	Within 28 days of randomisation (unless specified in footnotes)	Cycle 1	Cycle 2	Cycle 3	Cycle 4	Cycle 5	Cycle 6	1st maintenance visit/end of chemotherapy visit	Subsequent 6-weekly visit	End of bevacizumab visit	6-weekly follow-up visits (after end of bevacizumab)	Week 66 visit	Subsequent follow-up visits	At progression
**Cycle Day**		**1**	**1**	**1**	**1**	**1**	**1**							
**Pregnancy test[c]**	X													
**Coagulation profile[b]**	X													
**Full blood count[d]**	X[d]	X	X	X	X	X	X	X[o]	X[r]					
**Biochemistry[d]**	X[d]	X	X	X	X	X	X	X[o]						
**GFR**	X[g]													
**Carboplatin[e f]**		X	X	X	X	X	X							
**Paclitaxel[f]**		X	X	X	X	X	X							
**Bevacizumab[f]**		X[f]	X	X	X	X	X	X[r]	X[r]					
**Tumour assessments**	X[h]							X[o]				X		X
**CA 125[d]**	X[i]	X[a]	X	X	X	X	X	X[o]	X[r]	X[p]	X	X	X	X
**Chest X-ray**	X[j]													
**Concomitant medication**	X	X	X	X	X	X	X	X[o]	X[r]	X[p]				
**Adverse events**			X	X	X	X	X	X[o]	X[r]	X[p]	X	X		
**Quality of Life questionnaires**	X		X[k]	X[k]	X[k]	X[k]	X[k]	X[o]		X[p]		X	X[k]	
**Medical Resource Use questionnaire**			X	X	X	X	X	X[o]		X[p]		X	X	

	Screening	Chemotherapy phase						Maintenance phase			Follow-Up phase			
**Assessments**	**Within 28 days of randomisation (unless specified in footnotes)**	**Cycle 1**	**Cycle 2**	**Cycle 3**	**Cycle 4**	**Cycle 5**	**Cycle 6**	**1st maintenance visit/end of chemotherapy visit**	**Subsequent 6-weekly visit**	**End of bevacizumab visit**	**6-weekly follow-up visits (after end of bevacizumab)**	**Week 66 visit**	**Subsequent follow-up visits**	**At progression**
**Cycle Day**		**1**	**1**	**1**	**1**	**1**	**1**							
**Tumour Tissue Block (TR Level 1)**	X[l]													
**Blood for DNA (TR Level 2)**	X[m]													
**Blood for plasma (TR Level 3)**	X[n]		X[n]	X[n]	X[n]	X[n]	X[n]	X[n]	X[n]	X[n]	X[n]	X[n]	X[n]	X[n]
**Ethnicity** [q]	X[q]													
**Smoking status** [q]	X[q]													
**Family history of ovarian cancer** [q]	X[q]													

a. Repeat assessments not required if already performed during previous 7 days for screening purposes.
b. Repeat, if indicated, during study treatment.
c. A pregnancy test is only required for women of childbearing potential.
d. Screening bloods, urine protein dipstick & BP should be performed 14 days prior to cycle 1 day 1. In each cycle, haematology & biochemistry, urine protein dipstick & BP are required 72 hours prior to d1 chemotherapy. LDH is not required but will be recorded in the CRF if available.
e. Carboplatin (AUC5 or 6 depending on method used to calculate GFR) given on day 1.
f. Chemotherapy to commence within 8 weeks after surgery and within 2 weeks after randomisation. Bevacizumab should be omitted if day 1 of chemotherapy is ≤28 days after surgery. During chemotherapy phase visits, patients should be seen 3-weekly, 72 hours prior to administration of day 1 of each chemotherapy cycle.
g. Either a measured (radioisotopic clearance or 24 hour urine collection) or calculated GFR (Wright, Cockcroft-Gault or Jelliffe formulae) can be used to calculate the carboplatin dose. A pre-randomisation measured GFR is mandatory if the calculated GFR is <60ml/min and must occur within 14

days before randomisation (28 days allowed in specific circumstances – see **Section B5.1: Screening Procedures**). If a calculated GFR is used it must be recalculated within 7 days before cycle 1 day 1. Repeat GFR measurement or calculation during treatment if: an increase in serum creatinine ≥grade 2 occurs or the serum creatinine changes by ≥10%, and at cycle 2 if there was significant doubt about the true GFR at cycle 1. Routine recalculation of the carboplatin dose at the start of each cycle is not expected unless these conditions are met (see **Section B3.4.2: Carboplatin**).

h. The post-operative baseline tumour assessment (CT abdo/pelvis) should be performed no less than 4 weeks after surgery, and no more than 2 weeks after study treatment starts. The only exception to this is if clinically it is necessary to start treatment within 2 weeks of surgery. In this instance the baseline post-operative CT scan should be done at 4 weeks post-operatively (± 7 days).

i. If available, pre-surgery CA 125 measurement should also be provided.

j. Imaging of the chest must be performed to check for thoracic metastases in all patients prior to randomisation. This can be by CT or Chest X-ray, but if there is any clinical or radiological suspicion of parenchymal lung metastases or mediastinal metastases on the X-ray then a CT or MRI must be performed and disease measured according to RECIST criteria.

k. QoL form to be completed up to 72 hours before day one of the cycle. During long term follow up a QoL form should be completed at week 102, month 36, month 48, month 60 and month 72.

l. Tumour tissue block from primary surgery required for all levels of Translational Research.

m. Blood for DNA. Ideally take prior to cycle 1 but can be taken at any time throughout treatment if necessary. Required for level 2 and above of Translational Research.

n. Blood samples for Translational Research level 3. To be taken at baseline prior to cycle 1, at cycle 2-6, at bevacizumab maintenance cycles 7, 9, 11, 13, 15 and 17, at the end of bevacizumab treatment, all 6-weekly follow-up visits, week 66, all long term follow-up visits and at documentation of disease progression or 5 years after randomisation if patient has not progressed. Serial plasma samples during the chemotherapy phase and the bevacizumab maintenance phase can be collected up to 72 hours prior to day 1 of the cycle

o. End of chemotherapy treatment assessments should be done 3 weeks (±1 week) after day 1 of the last cycle of 1st line chemotherapy. End of chemotherapy tumour assessments should take place 3 weeks (± 1 week) after day 1 of the last cycle of 1st line chemotherapy. At subsequent follow-up visits until week 66 tumour assessments should only be performed if clinical examination/ symptoms are suggestive of recurrence or there is CA125 progression by GCIG criteria. They should then occur within 3 weeks of suspected clinical or CA125 progression. If there is CA125 progression but no radiological documentation of disease progression then imaging should be repeated 3-monthly until protocol defined progression is documented.

p. End of bevacizumab visit should be 3 weeks (+/-1 week) after the final cycle of bevacizumab. If patients stop bevacizumab early, 6-weekly follow-up should continue until week 66 post-randomisation. If this visit is due within 6 weeks of the week 66 visit then it may be omitted.

q. Only required for patients who consent to level 2 and above of Translational Research.

R. Bevacizumab administered on day 1 every 3 weeks during maintenance phase. Patient should have BP and urine dipstick performed prior to every bevacizumab administration (3-weekly). Patients should be seen 6-weekly for a full clinical review within 5 days of the first bevacizumab maintenance administration and within 5 days of every other cycle thereafter (as detailed in **B5.4.2 MAINTENANCE PHASE (ARMS B1 AND B3)**

s. Dental examination required prior to commencing treatment only for patients who have received intravenous bisphosphonates and are randomised to receive bevacizumab.

B6.2 TABLE 20: TRIAL ASSESSMENTS SCHEDULE FOR IPS PATIENTS ICON8B ARM B2: 3-WEEKLY CARBOPLATIN + WEEKLY PACLITAXEL

Assessments	Screening	Chemotherapy phase																			Follow-Up phase			
	Within 28 days of randomisation (unless specified in footnotes)	Cycle 1			Cycle 2			Cycle 3			Cycle 4			Cycle 5			Cycle 6			End of chemotherapy visit	6-weekly follow-up visits	Week 66 visit	Subsequent follow-up visits	At progression
Cycle Day		1	8	15	1	8	15	1	8	15	1	8	15	1	8	15	1	8	15					
Informed consent	X																							
Disease confirmation (histology)	X																							
Demographics	X																							
Medical history	X																							
Physical examination	X[a]	X			X			X			X			X			X							
Blood pressure	X																							
Urine protein dipstick	X																							
Height	X																							
Weight	X[a]	X			X			X			X			X			X							
ECG[b]	X[b]																							
Performance Status	X[a]	X			X			X			X			X			X			X[o]	X	X	X	X

Assessments	Screening	Chemotherapy phase																			Follow-Up phase			
	Within 28 days of randomisation (unless specified in footnotes)	Cycle 1			Cycle 2			Cycle 3			Cycle 4			Cycle 5			Cycle 6			End of chemotherapy visit	6-weekly follow-up visits	Week 66 visit	Subsequent follow-up visits	At progression
**Cycle Day**		1	8	15	1	8	15	1	8	15	1	8	15	1	8	15	1	8	15					
**Pregnancy test[c]**	X[c]																							
**Coagulation profile[b]**	X[b]																							
**Full blood count[d]**	X[d]	X	X	X	X	X	X	X	X	X	X	X	X	X	X	X	X	X	X	X[o]				
**Biochemistry[d]**	X[d]	X			X			X			X			X			X			X[o]				
**Carboplatin[e]**		X[e]			X[e]			X[e]			X[e]			X[e]			X[e]							
**Paclitaxel [f]**		X[f]	Xf	X[f]	X[f]	X[f]	X[f]	X[f]	X[f]	X[f]	X[f]	X[f]	X[f]	X[f]	X[f]	X[f]	X[f]	X[f]	X[f]					
**GFR**	X[g]																							
**Tumour assessments**	X[h]																			X[o]		X		X
**CA 125**	X[i]	X			X			X			X			X			X			X[o]	X	X	X	X
**Chest X-ray**	X[j]																							
**Concomitant medication**	X	X			X			X			X			X			X			X[o]				
**Adverse events**					X			X			X			X			X			X[o]	X	X		
**Quality of Life questionnaires**	X				X[k]			X[k]			X[k]			X[k]			X[k]			X[o]		X	X[k]	

Assessments	Screening	Chemotherapy phase																			Follow-Up phase			
	Within 28 days of randomisation (unless specified in footnotes)	Cycle 1			Cycle 2			Cycle 3			Cycle 4			Cycle 5			Cycle 6			End of chemotherapy visit	6-weekly follow-up visits	Week 66 visit	Subsequent follow-up visits	At progression
**Cycle Day**		1	8	15	1	8	15	1	8	15	1	8	15	1	8	15	1	8	15					
**Medical Resource Use questionnaire**					X[k]			X			X[k]			X[k]			X[k]			X[o]		X	X[k]	
**Tumour Tissue Block (TR Level 1)**	X[l]																							
**Blood for DNA (TR Level 2)**	X[m]																							
**Blood for plasma (TR Level 3)**	X[n]				X[n]			X[n]			X[n]			X[n]			X[n]			X[n]	X[n]	X[n]	X[n]	X[n]
**Ethnicity [p]**	X[p]																							
**Smoking status [p]**	X[p]																							
**Family history of ovarian cancer [p]**	X[p]																							

a. Repeat assessments not required if already performed during previous 7 days for screening purposes.
b. Repeat, if indicated, during study treatment.
c. A pregnancy test is only required for women of childbearing potential.

d. Screening bloods should be performed 14 days prior to cycle 1 day 1. In each cycle, haematology and biochemistry tests are required 72 hours prior to d1 chemotherapy. LDH is not required to be measured but will be recorded in the CRF if available.

e. Carboplatin (AUC5 or 6 depending on method used to calculate GFR) given on day 1. Chemotherapy to commence within 8 weeks after surgery and within 2 weeks after randomisation. During chemotherapy phase visits, patients should be seen 3-weekly, 72 hours prior to administration of day 1 of each chemotherapy cycle.

f. Paclitaxel (80mg/m^2) given on day 1, 8 and 15.

g. Either a measured (radioisotopic clearance or 24 hour urine collection) or calculated GFR (Wright, Cockcroft-Gault or Jelliffe formulae) can be used to calculate the carboplatin dose. A pre-randomisation measured GFR is mandatory if the calculated GFR is <60ml/min and must occur within 14 days before randomisation (28 days allowed in specific circumstances – see **Section B5.1: Screening Procedures**). If a calculated GFR is used it must be recalculated within 7 days before cycle 1 day 1. Repeat GFR measurement or calculation during treatment if: an increase in serum creatinine ≥grade 2 occurs or the serum creatinine changes by ≥10%, and at cycle 2 if there was significant doubt about the true GFR at cycle 1. Routine recalculation of the carboplatin dose at the start of each cycle is not expected unless these conditions are met (see **Section B3.4.2: Carboplatin**).

h. The post-operative baseline tumour assessment (CT abdo/pelvis) should be performed no less than 4 weeks after surgery, and no more than 2 weeks after study treatment starts. The only exception to this is if clinically it is necessary to start treatment within 2 weeks of surgery. In this instance the baseline post-operative CT scan should be done at 4 weeks post-operatively (± 7 days).

i. If available, pre-surgery CA 125 measurement should also be provided.

j. Imaging of the chest must be performed to check for thoracic metastases in all patients prior to randomisation. This can be by CT or Chest X-ray, but if there is any clinical or radiological suspicion of parenchymal lung metastases or mediastinal metastases on the X-ray then a CT or MRI must be performed and disease measured according to RECIST criteria.

k. QoL form to be completed up to 72 hours before day one of the cycle. During long term follow up a QoL form should be completed at week 102, month 36, month 48, month 60 and month 72.

l. Tumour tissue block from primary surgery required for all levels of Translational Research.

m. Blood for DNA. Ideally take prior to cycle 1 but can be taken at any time throughout treatment if necessary. Required for level 2 and above of Translational Research.

n. Blood samples for Translational Research level 3. To be taken at baseline prior to cycle 1, at cycles 2-6, at the end of chemotherapy visit, at all 6-weekly follow-up visits, week 66, all long term follow-up visits and at documentation of disease progression or 5 years after randomisation if patient has not progressed. Serial plasma samples during the chemotherapy phase can be collected up to 72 hours prior to day 1 of the cycle

o. End of chemotherapy treatment assessments should be done 3 weeks (±1 week) after day 1 of the last cycle of 1st line chemotherapy. End of chemotherapy tumour assessments should take place 3 weeks (±1 weeks) after day 1 of the last cycle of 1st line chemotherapy. At subsequent follow-up visits until week 66 tumour assessments should only be performed if clinical examination/ symptoms are suggestive of recurrence or there is CA125 progression by GCIG criteria. They should then occur within 3 weeks of suspected clinical or CA125 progression. If there is CA125 progression but no radiological documentation of disease progression then imaging should be repeated 3-monthly until protocol defined progression is documented.

p. Only required for patients who consent to level 2 and above of Translational Research.

B6.3 TABLE 21: TRIAL ASSESSMENTS SCHEDULE FOR IPS PATIENTS ICON8B ARM B3: 3-WEEKLY CARBOPLATIN + BEVACIZUMAB + WEEKLY PACLITAXEL

Assessments	Screening	Chemotherapy phase																		Maintenance phase			Follow-Up phase			
	Within 28 days of randomisation (unless specified in footnotes)	Cycle 1			Cycle 2			Cycle 3			Cycle 4			Cycle 5			Cycle 6			1st maintenance visit/end of chemotherapy visit	Subsequent 6-weekly visit	End of bevacizumab visit	6-weekly follow-up visits (after end of bevacizumab)	Week 66 visit	Subsequent follow-up visits	At progression
**Cycle Day**		1	8	15	1	8	15	1	8	15	1	8	15	1	8	15	1	8	15							
**Informed consent**	X																									
**Disease confirmation (histology)**	X																									
**Demographics**	X																									
**Medical history**	X																									
**Physical examination**	X	X			X			X			X			X			X			X[p]	X[s]	X[q]				
**Dental Examination**	X[t]																									
**Blood pressure [d]**	X[d]	X			X			X			X			X			X			X[p]	X[s]	X[q]				
**Urine protein dipstick [d]**	X[d]	X			X			X			X			X			X			X[p]	X[s]	X[q]				
**Height**	X																									
**Weight [d]**	X	X[a]			X			X			X			X			X			X[p]	X[s]					

Assessments	Screening	Chemotherapy phase																		Maintenance phase			Follow-Up phase			
	Within 28 days of randomisation (unless specified in footnotes)	Cycle 1			Cycle 2			Cycle 3			Cycle 4			Cycle 5			Cycle 6			1st maintenance visit/end of chemotherapy visit	Subsequent 6-weekly visit	End of bevacizumab visit	6-weekly follow-up visits (after end of bevacizumab)	Week 66 visit	Subsequent follow-up visits	At progression
**Cycle Day**		1	8	15	1	8	15	1	8	15	1	8	15	1	8	15	1	8	15							
**ECG [b]**	X																									
**Performance Status**	X	X[a]			X			X			X			X			X			X[p]	X[s]	X[q]	X	X	X	X
**Pregnancy test[c]**	X																									
**Coagulation profile [b]**	X																									
**Full blood count [d]**	X[d]	X	X	X	X	X	X	X	X	X	X	X	X	X	X	X	X	X	X	X[p]	X[s]					
**Biochemistry [d]**	X[d]	X			X			X			X			X			X			X[p]						
**Carboplatin [e g]**		X[g]			X			X			X			X			X									
**Paclitaxel [f g]**		X[g]	X	X	X	X	X	X	X	X	X	X	X	X	X	X	X	X	X							
**Bevacizumab [g]**		X[g]			X			X			X			X			X			X[s]	X[s]					
**GFR**	X[h]																									
**Tumour assessments**	X[i]																			X[p]				X		X
**CA 125[d]**	X[j]	X[a]			X			X			X			X			X			X	X[s]	X[q]	X	X[q]	X	X
**Chest X-ray**	X[k]																									

Assessments	Screening	Chemotherapy phase																		Maintenance phase			Follow-Up phase			
	Within 28 days of randomisation (unless specified in footnotes)	Cycle 1			Cycle 2			Cycle 3			Cycle 4			Cycle 5			Cycle 6			1st maintenance visit/end of chemotherapy visit	Subsequent 6-weekly visit	End of bevacizumab visit	6-weekly follow-up visits (after end of bevacizumab)	Week 66 visit	Subsequent follow-up visits	At progression
**Cycle Day**		1	8	15	1	8	15	1	8	15	1	8	15	1	8	15	1	8	15							
**Concomitant medication**	X	X			X			X			X			X			X			X	X[s]	X[q]				
**Adverse events**					X			X			X			X			X			X	X[s]	X[q]	X	X[q]		
**Quality of Life questionnaires**	X				X[l]			X[l]			X[l]			X[l]			X[l]			X		X[q]		X[q]	X[l]	
**Medical Resource Use questionnaire**					X			X			X			X			X			X		X[q]		X[q]	X	
**Tumour Tissue Block (TR Level 1)**	X[m]																									
**Blood for DNA (TR Level 2)**	X[n]																									
**Blood for plasma (TR Level 3)**	X[o]				X[o]			X[o]			X[o]			X[o]			X[o]			X[o]		X[q o]	X[o]	X[o]	X[o]	X[o]
**Ethnicity [r]**	X[r]																									
**Smoking status [r]**	X[r]																									
**Family history of ovarian**	X[r]																									

Assessments	Screening	Chemotherapy phase																		Maintenance phase			Follow-Up phase			
	Within 28 days of randomisation (unless specified in footnotes)	Cycle 1			Cycle 2			Cycle 3			Cycle 4			Cycle 5			Cycle 6			1st maintenance visit/end of chemotherapy visit	Subsequent 6-weekly visit	End of bevacizumab visit	6-weekly follow-up visits (after end of bevacizumab)	Week 66 visit	Subsequent follow-up visits	At progression
**Cycle Day**		1	8	15	1	8	15	1	8	15	1	8	15	1	8	15	1	8	15							
**cancer [r]**																										

a. Repeat assessments not required if already performed during previous 7 days for screening purposes.
b. Repeat, if indicated, during study treatment.
c. A pregnancy test is only required for women of childbearing potential.
d. Screening bloods, urine protein dipstick & BP should be performed 14 days prior to cycle 1 day 1. In each cycle, haematology & biochemistry, urine protein dipstick & BP are required 72 hours prior to d1 chemotherapy. LDH is not required but will be recorded in the CRF if available.
e. Carboplatin (AUC5 or 6 depending on method used to calculate GFR) given on day 1.
f. Paclitaxel (80mg/m^2) given on day 1, 8 and 15.
g. Chemotherapy to commence within 8 weeks after surgery and within 2 weeks after randomisation. Bevacizumab should be omitted if day 1 of chemotherapy is ≤28 days after surgery. During chemotherapy phase visits, patients should be seen 3-weekly, 72 hours prior to administration of day 1 of each chemotherapy cycle.
h. Either a measured (radioisotopic clearance or 24 hour urine collection) or calculated GFR (Wright, Cockcroft-Gault or Jelliffe formulae) can be used to calculate the carboplatin dose. A pre-randomisation measured GFR is mandatory if the calculated GFR is <60ml/min and must occur within 14 days before randomisation (28 days allowed in specific circumstances – see **Section B5.1: Screening Procedures**). If a calculated GFR is used it must be recalculated within 7 days before cycle 1 day 1. Repeat GFR measurement or calculation during treatment if: an increase in serum creatinine ≥grade 2 occurs or the serum creatinine changes by ≥10%, and at cycle 2 if there was significant doubt about the true GFR at cycle 1. Routine recalculation of the carboplatin dose at the start of each cycle is not expected unless these conditions are met (see **Section B3.4.2: Carboplatin**).
i. The post-operative baseline tumour assessment (CT abdo/pelvis) should be performed no less than 4 weeks after surgery, and no more than 2 weeks after study treatment starts. The only exception to this is if clinically it is necessary to start treatment within 2 weeks of surgery. In this instance the baseline post-operative CT scan should be done at 4 weeks post-operatively (± 7 days).
j. If available, pre-surgery CA 125 measurement should also be provided.
k. Imaging of the chest must be performed to check for thoracic metastases in all patients prior to randomisation. This can be by CT or Chest X-ray, but if there is any clinical or radiological suspicion of parenchymal lung metastases or mediastinal metastases on the X-ray then a CT or MRI must be performed and disease measured according to RECIST criteria.

l. QoL form to be completed up to 72 hours before day one of the cycle. During long term follow up a QoL form should be completed at week 102, month 36, month 48, month 60 and month 72.

m. Tumour tissue block from primary surgery required for all levels of Translational Research.

n. Blood for DNA. Ideally take prior to cycle 1 but can be taken at any time throughout treatment if necessary. Required for level 2 and above of Translational Research.

o. Blood samples for Translational Research level 3. To be taken at baseline prior to cycle 1, at cycle 2-6, at bevacizumab maintenance cycles 7, 9, 11, 13, 15 and 17, at the end of bevacizumab treatment, all 6-weekly follow-up visits , week 66, all long term follow-up visits and at documentation of disease progression or 5 years after randomisation if patient has not progressed. Serial plasma samples during the chemotherapy phase and the bevacizumab maintenance phase can be collected up to 72 hours prior to day 1 of the cycle

p. End of chemotherapy treatment assessments should be done 3 weeks (±1 week) after day 1 of the last cycle of 1st line chemotherapy. End of chemotherapy tumour assessments should take place 3 weeks (±1 week) after day 1 of the last cycle of 1st line chemotherapy. At subsequent follow-up visits until week 66 tumour assessments should only be performed if clinical examination/ symptoms are suggestive of recurrence or there is CA125 progression by GCIG criteria. They should then occur within 3 weeks of suspected clinical or CA125 progression. If there is CA125 progression but no radiological documentation of disease progression then imaging should be repeated 3-monthly until protocol defined progression is documented.

q. End of bevacizumab visit should be 3 weeks (+/-1 week) after the final cycle of bevacizumab. If patients stop bevacizumab early, 6-weekly follow-up should continue until week 66 post-randomisation. If this visit is due within 6 weeks of the week 66 visit then it may be omitted.

r. Only required for patients who consent to level 2 and above of Translational Research.

S. Bevacizumab administered on day 1 every 3 weeks during maintenance phase. Patient should have BP and urine dipstick performed prior to every bevacizumab administration (3-weekly). Patients should be seen 6-weekly for a full clinical review within 5 days of the first bevacizumab maintenance administration and within 5 days of every other cycle thereafter (as detailed in **B5.4.2 MAINTENANCE PHASE (ARMS B1 AND B3)**

t. Dental examination required prior to commencing treatment only for patients who have received intravenous bisphosphonates and are randomised to receive bevacizumab.

B6.4 TABLE 22: TRIAL ASSESSMENTS SCHEDULE FOR DPS PATIENTS ICON8B ARM B1: 3-WEEKLY CARBOPLATIN + PACLITAXEL + BEVACIZUMAB

	Screening	Chemotherapy phase							Maintenance phase			Follow-Up phase			
**Assessments**	**Within 28 days of randomisation (unless specified in footnotes)**	**Cycle 1**	**Cycle 2**	**Cycle 3**	**Surgery**	**Cycle 4**	**Cycle 5**	**Cycle 6**	**1st maintenance visit/end of chemotherapy visit**	**Subsequent 6-weekly visit**	**End of bevacizumab visit**	**6-weekly follow-up visits (after end of bevacizumab)**	**Week 66 visit**	**Subsequent follow-up visits**	**At progression**
**Cycle Day**		1	1	1		1	1	1							
**Informed consent**	X														
**Disease confirmation (histology)**	X														
**Demographics**	X														
**Medical history**	X														

Assessments	Screening	Chemotherapy phase							Maintenance phase			Follow-Up phase			
	Within 28 days of randomisation (unless specified in footnotes)	Cycle 1	Cycle 2	Cycle 3	Surgery	Cycle 4	Cycle 5	Cycle 6	1st maintenance visit/end of chemotherapy visit	Subsequent 6-weekly visit	End of bevacizumab visit	6-weekly follow-up visits (after end of bevacizumab)	Week 66 visit	Subsequent follow-up visits	At progression
**Cycle Day**		1	1	1		1	1	1							
**Physical examination**	X	X[a]	X	X		X	X	X	X[m]	X[p]	X[n]				
**Dental Examination**	X[W]														
**Blood pressure** [d]	X[d]	X	X	X		X	X	X	X[mp]	X[p]	X[n]				
**Urine protein dipstick** [d]	X[d]	X	X	X		X	X	X	X[mp]	X[p]	X[n]				
**Height**	X														
**Weight**	X	X[a]	X	X		X	X	X	X[mp]	X[p]					
**ECG**[b]	X[b]														
**Performance Status**	X	X[a]	X	X		X	X	X	X[mp]	X[p]	X[n]	X	X	X	X
**Pregnancy test**[c]	X[c]														
**Coagulation profile**[b]	X[b]														
**Full blood count**[d]	X[d]	X	X	X		X	X	X	X[mp]	X[p]					
**Biochemistry**[d]	X[d]	X	X	X		X	X	X	X[m]						
**GFR**	X[g]														
**Carboplatin**[e f]		X[e f]	X[e f]	X[e f]		X[e f s]	X[e f]	X[e f]							
**Paclitaxel** [f]		X[f]	X	X		X[s]	X	X							
**Bevacizumab** [f]		X[f]	X			X[s t]	X	X	X[p]	X[p]					
**Tumour assessments**	X			X[u]		X[v]			X[m]				X		X

	Screening	Chemotherapy phase							Maintenance phase			Follow-Up phase			
**Assessments**	**Within 28 days of randomisation (unless specified in footnotes)**	**Cycle 1**	**Cycle 2**	**Cycle 3**	**Surgery**	**Cycle 4**	**Cycle 5**	**Cycle 6**	**1st maintenance visit/end of chemotherapy visit**	**Subsequent 6-weekly visit**	**End of bevacizumab visit**	**6-weekly follow-up visits (after end of bevacizumab)**	**Week 66 visit**	**Subsequent follow-up visits**	**At progression**
**Cycle Day**		1	1	1		1	1	1							
**CA 125[d]**	X[d]	X[a]	X	X		X	X	X	X[m]	X[p]	X[n]	X	X	X	X
**Chest X-ray**	X[h]														
**Concomitant medication**	X	X	X	X		X	X	X	X[m]	X[p]	X[n]				
**Adverse events**			X	X		X	X	X	X[mp]	X[p]	X[n]	X	X		
**Primary Surgery**					X[q]										
**Surgical Adverse Events**						X									
**Quality of Life questionnaires**	X		X[i]	X[i]		X[i]	X[i]	X[i]	X[mp]		X[n]		X	X[i]	
**Medical Resource Use questionnaire**			X	X		X	X	X	X[mp]		X[n]		X	X	
**Tumour Tissue Block (TR Level 1)**	X[j]				X										
**Blood for DNA (TR Level 2)**	X[k]														
**Blood for plasma (TR Level 3)**	X[l]		X[l]	X[l]		X[l]	X[l]	X[l]	X[l]		X[l]	X[l]	X[l]	X[l]	X[l]
**Ethnicity[o]**	X[o]														
**Smoking status[o]**	X[o]														
**Family history of ovarian**	X[o]														

Assessments	Screening	Chemotherapy phase							Maintenance phase			Follow-Up phase			
	Within 28 days of randomisation (unless specified in footnotes)	Cycle 1	Cycle 2	Cycle 3	Surgery	Cycle 4	Cycle 5	Cycle 6	1st maintenance visit/end of chemotherapy visit	Subsequent 6-weekly visit	End of bevacizumab visit	6-weekly follow-up visits (after end of bevacizumab)	Week 66 visit	Subsequent follow-up visits	At progression
**Cycle Day**		1	1	1		1	1	1							
**cancer** [o]															

a. Repeat assessments not required if already performed during previous 7 days for screening purposes.
b. Repeat, if indicated, during study treatment.
c. A pregnancy test is only required for women of childbearing potential.
d. Screening bloods, urine protein dipstick & BP should be performed 14 days prior to cycle 1 day 1. In each cycle, haematology & biochemistry, urine protein dipstick & BP are required 72 hours prior to d1 chemotherapy. LDH is not required but will be recorded in the CRF if available.
e. Carboplatin (AUC5 or 6 depending on method used to calculate GFR) given on day 1.
f. Chemotherapy+Bevacizumab to commence within 2 weeks after randomisation. During chemotherapy phase visits, patients should be seen 3-weekly, 72 hours prior to administration of day 1 of each chemotherapy cycle.
g. Either a measured (radioisotopic clearance or 24 hour urine collection) or calculated GFR (Wright, Cockcroft-Gault or Jelliffe formulae) can be used to calculate the carboplatin dose. A pre-randomisation measured GFR is mandatory if the calculated GFR is <60ml/min and must occur within 14 days before randomisation (28 days allowed in specific circumstances – see Section **Section B5.1: Screening Procedures**). If a calculated GFR is used it must be recalculated within 7 days before cycle 1 day 1. Repeat GFR measurement or calculation during treatment if: an increase in serum creatinine ≥grade 2 occurs or the serum creatinine changes by ≥10%, and at cycle 2 if there was significant doubt about the true GFR at cycle 1.

Routine recalculation of the carboplatin dose at the start of each cycle is not expected unless these conditions are met (see **Section B3.4.2: Carboplatin**).

h. Imaging of the chest must be performed to check for thoracic metastases in all patients prior to randomisation. This can be by CT or Chest X-ray, but if there is any clinical or radiological suspicion of parenchymal lung metastases or mediastinal metastases on the X-ray then a CT or MRI must be performed and disease measured according to RECIST criteria.
i. QoL form to be completed up to 72 hours before day one of the cycle. During long term follow up a QoL form should be completed at week 102, month 36, month 48, month 60 and month 72.
j. Tumour tissue block from diagnostic biopsy and primary surgery required for all levels of Translational Research.
k. Blood for DNA. Ideally take prior to cycle 1 but can be taken at any time throughout treatment if necessary. Required for level 2 and above of Translational Research.
l. Blood samples for Translational Research level 3. To be taken at baseline prior to cycle 1, at cycle 2-6, at bevacizumab maintenance cycles 7, 9, 11, 13, 15 and 17, at the end of bevacizumab treatment, all 6-weekly follow-up visits, week 66, all long term follow-up visits and at documentation of disease progression or 5 years after randomisation if patient has not progressed. Serial plasma samples during the chemotherapy phase and the bevacizumab maintenance phase can be collected up to 72 hours prior to day 1 of the cycle
m. End of chemotherapy treatment assessments should be done 3 weeks (±1 week) after day 1 of the last cycle of 1st line chemotherapy. End of chemotherapy tumour assessments should take place 3 weeks (±1 weeks) after day 1 of the last cycle of 1st line chemotherapy. At subsequent follow-up visits until week 66 tumour assessments should only be performed if clinical examination/ symptoms are suggestive of recurrence or there is CA125 progression by GCIG criteria. They should then occur within 3 weeks of suspected clinical or CA125 progression. If there is CA125 progression but no radiological documentation of disease progression then imaging should be repeated 3-monthly until protocol defined progression is documented.
n. End of bevacizumab visit should be 3 weeks (+/-1 week) after the final cycle of bevacizumab. If patients stop bevacizumab early, 6-weekly follow-up should continue until week 66 post-randomisation. If this visit is due within 6 weeks of the week 66 visit then it may be omitted.
o. Only required for patients who consent to level 2 and above of Translational Research.
P. Bevacizumab administered on day 1 every 3 weeks during maintenance phase. Patient should have BP and urine dipstick performed prior to every bevacizumab administration (3-weekly). Patients should be seen 6-weekly for a full clinical review within 5 days of the first bevacizumab maintenance administration and within 5 days of every other cycle thereafter (as detailed in **B5.4.2 MAINTENANCE PHASE (ARMS B1 AND B3)**
q. Surgery to occur within 10 days after cycle 3 day 22.
r. Bevacizumab must be omitted during cycle 3.
s. Chemotherapy should recommence as soon as possible after surgery but must be ≥1 week post surgery.
t. If cycle 4 day 1 is ≤28 days after surgery bevacizumab should be omitted from cycle 4.
u. Mid-point imaging should be performed pre-operatively for surgical planning according to local practice, generally during cycle 3.
v. A CT scan should be performed 4 weeks +/-1 week after surgery which will be the baseline assessment for documentation of future progression.
w. Dental examination required prior to commencing treatment only for patients who have received intravenous bisphosphonates and are randomised to receive bevacizumab.

B6.5 TABLE 23: TRIAL ASSESSMENTS SCHEDULE FOR DPS PATIENTS ICON8B ARM B2: 3-WEEKLY CARBOPLATIN + WEEKLY PACLITAXEL

Assessments	Screening	Chemotherapy phase																				Follow-Up phase			
	Within 28 days of randomisation (unless specified in footnotes)	Cycle 1			Cycle 2			Cycle 3			Surgery	Cycle 4			Cycle 5			Cycle 6			End of chemotherapy visit	6-weekly follow-up visits	Week 66 visit	Subsequent follow-up visits	At progression
**Cycle Day**		**1**	**8**	**15**	**1**	**8**	**15**	**1**	**8**	**15**		**1**	**8**	**15**	**1**	**8**	**15**	**1**	**8**	**15**					
**Informed consent**	X																								

Assessments	Screening	Chemotherapy phase																				Follow-Up phase			
	Within 28 days of randomisation (unless specified in footnotes)	Cycle 1			Cycle 2			Cycle 3			Surgery	Cycle 4			Cycle 5			Cycle 6			End of chemotherapy visit	6-weekly follow-up visits	Week 66 visit	Subsequent follow-up visits	At progression
**Cycle Day**		1	8	15	1	8	15	1	8	15		1	8	15	1	8	15	1	8	15					
**Disease confirmation (histology)**	X																								
**Demographics**	X																								
**Medical history**	X																								
**Physical examination**	X	X[a]			X			X				X			X			X			X[m]				
**Blood pressure**	X																								
**Urine protein dipstick**	X																								
**Height**	X																								
**Weight**	X	X[a]			X			X				X			X			X							
**ECG[b]**	X[b]																								
**Performance Status**	X	X[a]			X			X				X			X			X			X[m]	X	X	X	X
**Pregnancy test [c]**	X[c]																								
**Coagulation profile [b]**	X[b]																								

Assessments	Screening	Chemotherapy phase																				Follow-Up phase			
	Within 28 days of randomisation (unless specified in footnotes)	Cycle 1			Cycle 2			Cycle 3			Surgery	Cycle 4			Cycle 5			Cycle 6			End of chemotherapy visit	6-weekly follow-up visits	Week 66 visit	Subsequent follow-up visits	At progression
Cycle Day		1	8	15	1	8	15	1	8	15		1	8	15	1	8	15	1	8	15					
Full blood count [d]	X [d]	X	X	X	X	X	X	X	X	X		X	X	X	X	X	X	X	X	X	X [m]				
Biochemistry[d]	X [d]	X			X			X				X			X			X			X [m]				
Carboplatin [e f]		X[e]			X[e]			X[e]				X[q]			X[e]			X[e]							
Paclitaxel [f p]		X[f]	X	X	X	X	X	X	X			X[q]	X	X	X	X	X	X	X	X					
GFR	X [g]																								
Tumour assessments	X								X [r]				X [s]								X [m]		X		X
Primary Surgery											X [o]														
CA 125[d]	X	X[a]			X			X				X			X			X			X [m]	X	X	X	X
Chest X-ray	X [h]																								
Concomitant medication	X	X			X			X				X			X			X			X [m]				
Adverse events					X			X				X			X			X			X [m]	X	X		
Surgical adverse events												X													

Assessments	Screening	Chemotherapy phase																				Follow-Up phase			
	Within 28 days of randomisation (unless specified in footnotes)	Cycle 1			Cycle 2			Cycle 3			Surgery	Cycle 4			Cycle 5			Cycle 6			End of chemotherapy visit	6-weekly follow-up visits	Week 66 visit	Subsequent follow-up visits	At progression
**Cycle Day**		1	8	15	1	8	15	1	8	15		1	8	15	1	8	15	1	8	15					
**Quality of Life questionnaires**	X				X[i]			X[i]				X[i]			X[i]			X[i]			X		X	X[i]	
**Medical Resource Use questionnaire**					X			X				X			X			X			X		X	X	
**Tumour Tissue Block (TR Level 1)**	X[j]										X[j]														
**Blood for DNA (TR Level 2)**	X[k]																								
**Blood for plasma (TR Level 3)**	X[l]				X[l]			X[l]				X[l]			X[l]			X[l]			X[l]	X[l]	X[l]	X[l]	X[l]
**Ethnicity** [n]	X[n]																								
**Smoking status** [n]	X[n]																								
**Family history of ovarian cancer** [n]	X[n]																								

a. Repeat assessments not required if already performed during previous 7 days for screening purposes.
b. Repeat, if indicated, during study treatment.

c. A pregnancy test is only required for women of childbearing potential.

d. Screening bloods should be performed 14 days prior to cycle 1 day 1. In each cycle, haematology and biochemistry tests are required 72 hours prior to d1 chemotherapy. LDH is not required to be measured but will be recorded in the CRF if available.

e. Carboplatin (AUC5 or 6 depending on method used to calculate GFR) given on day 1.

f. Chemotherapy to commence within 2 weeks after randomisation. During chemotherapy phase visits, patients should be seen 3-weekly, 72 hours prior to administration of day 1 of each chemotherapy cycle.

g. Either a measured (radioisotopic clearance or 24 hour urine collection) or calculated GFR (Wright, Cockcroft-Gault or Jelliffe formulae) can be used to calculate the carboplatin dose. A pre-randomisation measured GFR is mandatory if the calculated GFR is <60ml/min and must occur within 14 days before randomisation (28 days allowed in specific circumstances – see Section **Section B5.1: Screening Procedures**). If a calculated GFR is used it must be recalculated within 7 days before cycle 1 day 1. Repeat GFR measurement or calculation during treatment if: an increase in serum creatinine ≥grade 2 occurs or the serum creatinine changes by ≥10%, and at cycle 2 if there was significant doubt about the true GFR at cycle 1. Routine recalculation of the carboplatin dose at the start of each cycle is not expected unless these conditions are met (see **Section B3.4.2: Carboplatin**).

h. Imaging of the chest must be performed to check for thoracic metastases in all patients prior to randomisation. This can be by CT or Chest X-ray, but if there is any clinical or radiological suspicion of parenchymal lung metastases or mediastinal metastases on the X-ray then a CT or MRI must be performed and disease measured according to RECIST criteria.

i. QoL form to be completed up to 72 hours before day one of the cycle. During long term follow up a QoL form should be completed at week 102, month 36, month 48, month 60 and month 72.

j. Tumour tissue block from diagnostic biopsy and primary surgery required for all levels of Translational Research.

k. Blood for DNA. Ideally take prior to cycle 1 but can be taken at any time throughout treatment if necessary. Required for level 2 and above of Translational Research.

l. Blood samples for Translational Research level 3. To be taken at baseline prior to cycle 1, at cycles 2-6, at the end of chemotherapy visit, at all 6-weekly follow-up visits, week 66, all long term follow-up visits and at documentation of disease progression or 5 years after randomisation if patient has not progressed. Serial plasma samples during the chemotherapy phase can be collected up to 72 hours prior to day 1 of the cycle

m. End of chemotherapy treatment assessments should be done 3 weeks (±1 week) after day 1 of the last cycle of 1st line chemotherapy. End of chemotherapy tumour assessments should take place 3 weeks (±1 week) after day 1 of the last cycle of 1st line chemotherapy. At subsequent follow-up visits until week 66 tumour assessments should only be performed if clinical examination/ symptoms are suggestive of recurrence or there is CA125 progression by GCIG criteria. They should then occur within 3 weeks of suspected clinical or CA125 progression. If there is CA125 progression but no radiological documentation of disease progression then imaging should be repeated 3-monthly until protocol defined progression is documented.

n. Only required for patients who consent to level 2 and above of Translational Research.

o. Surgery to occur within 10 days after cycle 3 day 22.

p. Paclitaxel must be omitted on cycle 3 day 15 prior to surgery. If surgery takes place at a later cycle please ensure day 15 paclitaxel is omitted from the cycle immediately prior to surgery.

q. Chemotherapy should recommence as soon as possible after surgery but must be ≥1 week post surgery.
r. Mid-point imaging should be performed pre-operatively for surgical planning according to local practice, generally during cycle 3.
s. A CT scan should be performed 4 weeks +/-1 week after surgery which will be the baseline assessment for documentation of future progression.

B6.6 TABLE 24: TRIAL ASSESSMENTS SCHEDULE FOR DPS PATIENTS ICON8B ARM B3: 3-WEEKLY CARBOPLATIN + BEVACIZUMAB + WEEKLY PACLITAXEL

Assessments	Screening	Chemotherapy phase																				Maintenance phase			Follow-Up phase			
	Within 28 days of randomisation (unless specified in footnotes)	Cycle 1			Cycle 2			Cycle 3			Surgery	Cycle 4			Cycle 5			Cycle 6				1st maintenance visit/end of chemotherapy visit	Subsequent 6-weekly visit	End of bevacizumab visit	6-weekly follow-up visits (after end of bevacizumab)	Week 66 visit	Subsequent follow-up visits	At progression
**Cycle Day**		1	8	15	1	8	15	1	8	15		1	8	15	1	8	15	1	8	15								
**Informed consent**	X																											
**Disease confirmation (histology)**	X																											
**Demographics**	X																											
**Medical history**	X																											
**Physical examination**	X	X[a]			X			X				X			X			X				X[m]	X[p]	X[n]				
**Dental Examination**	X[y]																											
**Blood pressure [d]**	X[d]	X			X			X				X			X			X				X[m]	X[p]	X[n]				
**Urine protein dipstick [d]**	X[d]	X			X			X				X			X			X				X[m]	X[p]	X[n]				
**Height**	X																											
**Weight[d]**	X	X[a]			X			X				X			X			X				X[m]	X[p]					
**ECG[b]**	X[b]																											

Assessments	Screening	Chemotherapy phase																			Maintenance phase			Follow-Up phase			
	Within 28 days of randomisation (unless specified in footnotes)	Cycle 1			Cycle 2			Cycle 3			Surgery	Cycle 4			Cycle 5			Cycle 6			1st maintenance visit/end of chemotherapy visit	Subsequent 6-weekly visit	End of bevacizumab visit	6-weekly follow-up visits (after end of bevacizumab)	Week 66 visit	Subsequent follow-up visits	At progression
**Cycle Day**		1	8	15	1	8	15	1	8	15		1	8	15	1	8	15	1	8	15							
**Performance Status**	X	X[a]			X			X				X			X			X			X[m]	X[p]	X[n]	X	X	X	X
**Pregnancy test[c]**	X[c]																										
**Coagulation profile[b]**	X[b]																										
**Full blood count[d]**	X[d]	X	X	X	X	X	X	X	X			X	X	X	X	X	X	X	X	X	X[m]	X[p]					
**Biochemistry[d]**	X[d]	X			X			X				X			X			X			X[m]						
**GFR**	X[g]																										
**Carboplatin[e f]**		X[f]			X[e]			X[e]				X[t]			X[e]			X[e]									
**Paclitaxel[f s]**		X[f]	X	X	X	X	X	X	X[s]			X[t]	X	X	X	X	X	X	X	X							
**Bevacizumab[f r]**		X[f]			X							X[r]			X			X			X[p]	X[p]					
**Primary Surgery**											X[q]																
**Tumour assessments**	X[h]								X[v]				X[w]								X[m]				X		X
**CA 125[d]**	X	X[a]			X			X				X			X			X			X[m]	X[p]	X[n]	X	X	X	X
**Chest X-ray**	X[h]																										

Assessments	Screening	Chemotherapy phase																			Maintenance phase			Follow-Up phase			
	Within 28 days of randomisation (unless specified in footnotes)	Cycle 1			Cycle 2			Cycle 3			Surgery	Cycle 4			Cycle 5			Cycle 6			1st maintenance visit/end of chemotherapy visit	Subsequent 6-weekly visit	End of bevacizumab visit	6-weekly follow-up visits (after end of bevacizumab)	Week 66 visit	Subsequent follow-up visits	At progression
**Cycle Day**		1	8	15	1	8	15	1	8	15		1	8	15	1	8	15	1	8	15							
**Concomitant medication**	X	X			X			X				X			X			X			X[m]	X[p]	X[n]				
**Adverse events**					X			X				X			X			X			X[m]	X[p]	X[n]	X	X		
**Surgical adverse event**												X															
**Quality of Life questionnaires**	X				X[x]			X[x]				X[x]			X[x]			X[x]			X		X		X	X[x]	
**Medical Resource Use questionnaire**					X			X				X			X			X			X		X		X	X	
**Tumour Tissue Block (TR Level 1)**	X[j]										X[j]																
**Blood for DNA (TR Level 2)**	X[k]																										
**Blood for plasma (TR Level 3)**	X[z]				X[z]			X[z]				X[z]			X[z]			X[z]			X[z]	X[Z]	X[z]	X[z]	X[z]	X[z]	X[z]
**Ethnicity** [o]	X[o]																										
**Smoking status** [o]	X[o]																										

	Screening	Chemotherapy phase																			Maintenance phase			Follow-Up phase			
**Assessments**	Within 28 days of randomisation (unless specified in footnotes)	Cycle 1			Cycle 2			Cycle 3			Surgery	Cycle 4			Cycle 5			Cycle 6			1st maintenance visit/end of chemotherapy visit	Subsequent 6-weekly visit	End of bevacizumab visit	6-weekly follow-up visits (after end of bevacizumab)	Week 66 visit	Subsequent follow-up visits	At progression
**Cycle Day**		1	8	15	1	8	15	1	8	15		1	8	15	1	8	15	1	8	15							
**Family history of ovarian cancer** [o]	X[o]																										

a. Repeat assessments not required if already performed during previous 7 days for screening purposes.
b. Repeat, if indicated, during study treatment.
c. A pregnancy test is only required for women of childbearing potential.
d. Screening bloods, urine protein dipstick & BP should be performed 14 days prior to cycle 1 day 1. In each cycle, haematology & biochemistry, urine protein dipstick & BP are required 72 hours prior to d1 chemotherapy. LDH is not required but will be recorded in the CRF if available.
e. Carboplatin (AUC5 or 6 depending on method used to calculate GFR) given on day 1.
f. Chemotherapy+Bevacizumab to commence within 2 weeks after randomisation. During chemotherapy phase visits, patients should be seen 3-weekly, 72 hours prior to administration of day 1 of each chemotherapy cycle.
g. Either a measured (radioisotopic clearance or 24 hour urine collection) or calculated GFR (Wright, Cockcroft-Gault or Jelliffe formulae) can be used to calculate the carboplatin dose. A pre-randomisation measured GFR is mandatory if the calculated GFR is <60ml/min and must occur within 14 days before randomisation (28 days allowed in specific circumstances – see **Section B5.1: Screening Procedures**). If a calculated GFR is used it must be recalculated within 7 days before cycle 1 day 1. Repeat GFR measurement or calculation during treatment if: an increase in serum creatinine ≥grade 2 occurs or the serum creatinine changes by ≥10%, and at cycle 2 if there was significant doubt about the true GFR at cycle 1. Routine recalculation of the carboplatin dose at the start of each cycle is not expected unless these conditions are met (see **Section B3.4.2: Carboplatin**).
h. Imaging of the chest must be performed to check for thoracic metastases in all patients prior to randomisation. This can be by CT or Chest X-ray, but if there is any clinical or radiological suspicion of parenchymal lung metastases or mediastinal metastases on the X-ray then a CT or MRI must be performed and disease measured according to RECIST criteria.

i. QoL form to be completed up to 72 hours before day one of the cycle. During long term follow up a QoL form should be completed at week 102, month 36, month 48, month 60 and month 72.
j. Tumour tissue block from diagnostic biopsy and primary surgery required for all levels of Translational Research.
k. Blood for DNA. Ideally take prior to cycle 1 but can be taken at any time throughout treatment if necessary. Required for level 2 and above of Translational Research.
l. Blood samples for Translational Research level 3. To be taken prior to cycle 1, 2, and 6, at end of bevacizumab treatment; and at documentation of disease progression or 5 years after randomisation if patient has not progressed.
m. End of chemotherapy treatment assessments should be done 3 weeks (±1 week) after day 1 of the last cycle of 1st line chemotherapy. End of chemotherapy tumour assessments should take place 3 weeks (±1 week) after day 1 of the last cycle of 1st line chemotherapy. At subsequent follow-up visits until week 66 tumour assessments should only be performed if clinical examination/ symptoms are suggestive of recurrence or there is CA125 progression by GCIG criteria. They should then occur within 3 weeks of suspected clinical or CA125 progression. If there is CA125 progression but no radiological documentation of disease progression then imaging should be repeated 3-monthly until protocol defined progression is documented.
n. End of bevacizumab visit should be 3 weeks (+/-1 week) after the final cycle of bevacizumab. If patients stop bevacizumab early, 6-weekly follow-up should continue until week 66 post-randomisation. If this visit is due within 6 weeks of the week 66 visit then it may be omitted.
o. Only required for patients who consent to level 2 and above of Translational Research.
P. Bevacizumab administered on day 1 every 3 weeks during maintenance phase. Patient should have BP and urine dipstick performed prior to every bevacizumab administration (3-weekly). Patients should be seen 6-weekly for a full clinical review within 5 days of the first bevacizumab maintenance administration and within 5 days of every other cycle thereafter (as detailed in **B5.4.2 MAINTENANCE PHASE (ARMS B1 AND B3)**
q. Surgery to occur within 10 days after cycle 3 day 22.
r. Bevacizumab must be omitted during cycle 3.
s. Paclitaxel must be omitted on cycle 3 day 15 prior to surgery. If surgery takes place at a later cycle please ensure day 15 paclitaxel is omitted from the cycle immediately prior to surgery.
t. Chemotherapy should recommence as soon as possible after surgery but must be ≥1 week post surgery.
u. If cycle 4 day 1 is ≤28 days after surgery bevacizumab should be omitted from cycle 4.
v. Mid-point imaging should be performed pre-operatively for surgical planning according to local practice, generally during cycle 3.
w. A CT scan should be performed 4 weeks +/-1 week after surgery which will be the baseline assessment for documentation of future progression.
x. QOL form to be completed at the end of cycle 3 prior to surgery. An extra clinic visit is not required for administration of this questionnaire. It can be given to the patient at the pre-operative cycle and returned by post or conducted by site staff via phone.
y. Dental examination required prior to commencing treatment only for patients who have received intravenous bisphosphonates and are randomised to receive bevacizumab.
z. Blood samples for Translational Research level 3. To be taken at baseline prior to cycle 1, at cycle 2-6, at bevacizumab maintenance cycles 7, 9, 11, 13, 15 and 17, at the end of bevacizumab treatment, all 6-weekly follow-up visits, week 66, all long term follow-up visits and at documentation of disease

progression or 5 years after randomisation if patient has not progressed. Serial plasma samples during the chemotherapy phase and the bevacizumab maintenance phase can be collected up to 72 hours prior to day 1 of the cycle.

B6.7 ARM B1 AND B3 LEVEL 3 TRANSLATIONAL SAMPLE TIMEPOINTS (PLASMA SAMPLING)

Please note that only selected centres will be participating at level 3 of sample collection. (X = plasma sampling timepoint)

	Trial Timepoint (chemotherapy phase)											
	Week 0	Week 3	Week 6	Week 9	Week 12	Week 15	Week 18	Week 21	Week 24	Week 27	Week 30	Week 33
	Baseline	Cycle 1	Cycle 2	Cycle 3	Cycle 4	Cycle 5	Cycle 6	End of Chemo Visit / Cycle 7		Cycle 9		Cycle 11
Arm B1 & B3 Level 3 Sample Timepoints	X		X	X	X	X	X	X		X		X

Trial Timepoint (6-weekly follow-up phase)

	Week 36	Week 39	Week 42	Week 45	Week 48	Week 51	Week 54	Week 57	Week 60	Week 63	Week 66
		Cycle 13		Cycle 15		Cycle 17		End of bevacizumab		6-weekly f/u visit	f/u visit
Arm B1 & B3 Level 3 Sample Timepoints		X		X		X		X		X	X

	Timepoint (long term follow-up phase)										
	Week 78	Week 90	Week 102	Month 30	Month 36	Month 42	Month 48	Month 54	Month 60	Month 66	Month 72/ Progression
	Long term f/u	Long term f/u	Long term f/u	Long term f/u	Long term f/u	Long term f/u	Long term f/u	Long term f/u	Long term f/u	Long term f/u	Long term f/u
Arm B1 & B3 Level 3 Sample Timepoints	X	X	X	X	X	X	X	X	X	X	X

B6.8 ARM B2 LEVEL 3 TRANSLATIONAL SAMPLE TIMEPOINTS (PLASMA SAMPLING)

Please note that only selected centres will be participating at level 3 of sample collection. (X = plasma sampling timepoint)

	Trial Timepoint (chemotherapy phase)											
	Week 0	Week 3	Week 6	Week 9	Week 12	Week 15	Week 18	Week 21	Week 24	Week 27	Week 30	Week 33

	Baseline	Cycle 1	Cycle 2	Cycle 3	Cycle 4	Cycle 5	Cycle 6	End of Chemo Visit		6-weekly f/u visit		6-weekly f/u visit
Arm B2 Level 3 Sample Timepoints	X		X	X	X	X	X	X		X		X

	**Trial Timepoint (6-weekly follow-up phase)**										
	Week 36	Week 39	Week 42	Week 45	Week 48	Week 51	Week 54	Week 57	Week 60	Week 63	Week 66
		6-weekly f/u visit		6-weekly f/u visit		6-weekly f/u visit		6-weekly f/u visit		6-weekly f/u visit	f/u visit
Arm B2 Level 3 Sample Timepoints		X		X		X		X		X	X

	**Timepoint (long term follow-up phase)**										
	Week 78	Week 90	Week 102	Month 30	Month 36	Month 42	Month 48	Month 54	Month 60	Month 66	Month 72/ Progression
	Long term f/u	Long term f/u	Long term f/u	Long term f/u	Long term f/u	Long term f/u	Long term f/u	Long term f/u	Long term f/u	Long term f/u	Long term f/u
Arm B2 Level 3 Sample Timepoints	X	X	X	X	X	X	X	X	X	X	X

3 SAFETY REPORTING

The principles of GCP require that both investigators and sponsors follow specific procedures when reporting adverse events or reactions in clinical trials. These procedures are described in this section of the protocol.

3.1 DEFINITIONS OF ADVERSE EVENTS AND ADVERSE REACTIONS

The definitions of the EU Directive 2001/20/EC Article 2 based on the Principles of ICH GCP apply to this trial protocol. These definitions are given in **Table 25**.

Table 25: Definitions of Adverse Events and Reactions

Term	Definition
**Adverse Event (AE)**	Any untoward medical occurrence in a patient or clinical trial subject to whom a medicinal product has been administered including occurrences which are not necessarily caused by or related to that product.
**Adverse Reaction (AR)**	Any untoward and unintended response to an investigational medicinal product related to any dose administered.
**Unexpected Adverse Reaction (UAR)**	An adverse reaction, the nature and severity of which is not consistent with the information about the medicinal product in question set out in the Summary of Product Characteristics (SPC) or Investigator Brochure (IB) for that product
**Serious AE (SAE) or Serious AR (SAR) or Suspected Unexpected Serious Adverse Reaction (SUSAR)**	Respectively any adverse event, adverse reaction or unexpected adverse reaction that:<br>• results in death<br>• is life-threatening*<br>• requires hospitalisation or prolongation of existing hospitalisation**<br>• results in persistent or significant disability or incapacity<br>• consists of a congenital anomaly or birth defect<br>• other important medical condition***

*The term life-threatening in the definition of a serious event refers to an event in which the patient is at risk of death at the time of the event; it does not refer to an event that hypothetically might cause death if it were more severe, for example, a silent myocardial infarction.

**Hospitalisation is defined as an inpatient admission, regardless of length of stay, even if the hospitalisation is a precautionary measure for continued observation. Hospitalisations for a pre-existing condition, that has not worsened or for an elective procedure do not constitute an SAE.

*** Medical judgement should be exercised in deciding whether an AE or AR is serious in other situations. The following should also be considered serious: important AEs or ARs that are not immediately life-threatening or do not result in death or hospitalisation but may jeopardise the subject or may require intervention to prevent one of the other outcomes listed in the definition above; for example, a secondary malignancy, an allergic bronchospasm requiring intensive emergency treatment, seizures or blood dyscrasias that do not result in hospitalisation or development of drug dependency.

3.1.1 Medicinal Products

An investigational medicinal product is defined as the tested investigational medicinal product and the comparators used in the study. (EU guidance ENTR/CT 3, April 2006 revision).

Adverse reactions include any untoward or unintended response to drugs. Reactions to an IMP or comparator should be reported appropriately.

Adverse Events

Adverse events will be graded using the NCI Common Toxicity Criteria (CTCAE) version 4.0, an abridged version is listed in **Appendix 7.** The complete version can be found at http://evs.nci.nih.gov/ftp1/CTCAE/CTCAE_4.03_2010-06-14_QuickReference_5x7.pdf

Adverse Events include:

- An exacerbation of a pre-existing illness
- An increase in frequency or intensity of a pre-existing episodic event or condition
- A condition (even though it may have been present prior to the start of the trial) detected after trial drug administration
- Continuous persistent disease or a symptom present at baseline that worsens following administration of the study treatment.

All patients experiencing a SAE or adverse event should be followed up as outlined in the protocol. For patients who decide to withdraw from **ICON8** trial treatment after an adverse event, the procedure outlined in **Section A3.8 Protocol Treatment Discontinuation**, should be followed.

For patients who decide to withdraw from **ICON8B** trial treatment after an adverse event, the procedure outlined in **Section B3.8 Protocol Treatment Discontinuation**, should be followed.

Exempted Serious Adverse Events

The following events, in the context of this trial, should not be considered as SAEs. No SAE form is required and they are exempt from expedited reporting. They must instead be reported on the appropriate CRF section:

1. Disease progression, symptoms of disease progression or death as a result of disease progression

2. Elective hospitalisation and surgery for treatment of ovarian cancer, primary peritoneal cancer, fallopian tube cancer or its complications

3. Elective hospitalisation to simplify treatment or procedures

4. Elective hospitalisation for pre-existing conditions that have not been exacerbated by trial treatment.

3.2 ICON8B SPECIFIC NOTABLE EVENTS

ICON8B Specific Notable Adverse Events (All Arms)

The following events are regarded as **Notable Adverse Events** for **ICON8B patients ONLY** and must be reported as an adverse event in the patient's CRF during the reporting period specified:

- Hypertension
- Proteinuria
- Arterial thromboembolic events
- Hemorrhagic (bleeding) events.

Up to the week 66 follow-up visit OR if a patient is still receiving trial treatment after week 66 then these events should continue to be reported until 30 days after the last protocol treatment administration.

3.2.2 ICON8B Specific Additional Notable Events Requiring Expedited Reporting (DPS PATIENTS ONLY)

The following **Notable Adverse Events** of the severity specified below, must be reported for **ICON8B** **DPS patients ONLY** within **24 hours** of becoming aware of the event using the ICON8B SAE form, regardless of whether they fulfill the standard definition of serious (detailed in **section 3.1: Definitions**). These notable events should be reported as per the SAE notification procedure outlined in **section 3.4.4: Notification Procedure.**

- Grade 3 and grade 4 hypertension
- Grade 3 proteinuria
- Nephrotic syndrome
- Grade 3 and grade 4 venous thromboembolic events including pulmonary embolism
- Any arterial thromboembolic events, including pulmonary embolism
- Grade 3 or grade 4 haemorrhagic (bleeding) events
- All grades of post operative wound healing complications or delayed wound healing
- All grades of gastro-intestinal perforation
- All grades of fistulae.

Report all new cases that occur up to the week 66 follow-up visit.

OR

If a patient is still receiving trial treatment after week 66 then these events should continue to be reported until 30 days after the last protocol treatment

3.3 OTHER NOTABLE EVENTS (ICON8 AND ICON8B, ALL ARMS)

3.3.1 Pregnancy

Pregnancy is very unlikely as all patients in this trial will most likely undergo hysterectomy and therefore will not be of child bearing potential. In the unlikely event a pregnancy does occur it will be recorded initially on the SAE form and will be followed up on the Outcome of Pregnancy CRF for both ICON8 and ICON8B patients.

3.4 CLINICAL TRIAL SITE/INVESTIGATOR RESPONSIBILITIES

All non-serious AEs and ARs, whether expected or not, should be recorded in the patient's medical notes and reported in the toxicity (symptoms) section of the appropriate CRF and sent to MRC CTU within **the agreed timescale**. SAEs and SARs should be notified to the MRC CTU **within 24 hours** of the investigator becoming aware of the event as described in **section 3.4.5 Notification Procedure**

A flowchart (**Figure 13**) is given at the end of this section to help explain the notification procedures. Any questions concerning this process should be directed to the MRC CTU and/or GCIG Group in the first instance.

3.4.1 INVESTIGATOR ASSESSMENT

3.4.1.1 Seriousness

When an AE or AR occurs the investigator responsible for the care of the patient must first assess whether the event is serious using the definitions given in **Table 25.** If the event is serious and not exempt from expedited reporting as detailed in **section 3.1.3: Exempted Adverse Events**, then an SAE form must be completed and faxed to MRC CTU and/or the relevant GCIG group within 24 hours following the notification procedure in **section 3.4.4: Notification Procedure**.

3.4.1.2 Severity or grading

The severity (ie. intensity) of all AEs and ARs (serious and non-serious) in this trial should be graded using Common Terminology Criteria for Adverse Events (CTCAE) v4.0, an abridged version is listed in **Appendix 7.** The complete version can be found at:
http://evs.nci.nih.gov/ftp1/CTCAE/CTCAE_4.03_2010-06-14_QuickReference_5x7.pdf

3.4.1.3 Causality

The Investigator must assess the causality of all adverse events in relation to the trial therapy using the definitions in **Table 26**. There are 5 causality categories: unrelated, unlikely, possible, probable and definitely related. If the causality assessment is unrelated or unlikely to be related then the event is classified as an SAE. If the causality is assessed as either possible, probable or definitely related then the event is classified as an SAR.

3.4.1.4 Expectedness

If there is at least a possible involvement of the trial treatment, the Investigator should make an initial assessment of the expectedness of the event using the list of expected toxicities for ICON8 and ICON8B provided by the MRC CTU. The Expected Adverse Events list for ICON8 and ICON8B are based on information in the current SPCs for carboplatin, paclitaxel and bevacizumab (for ICON8B only). Investigators must file the current version of the Expected Adverse Events list in the safety reporting section of their Investigator File.

An unexpected adverse reaction is one not previously reported in the current SPC or one that is more frequent or more severe that previously reported. The definition of an unexpected adverse reaction (UAE) is given in **Table 26**. Please see the current SPC for a list of expected toxicities associated with the drugs being used in this trial at https://www.medicines.org.uk/emc/. If a SAR is assessed as being unexpected, it becomes a SUSAR.

The sponsor has final responsibility for determination of expectedness (for reporting purposes), and this decision will be made on the basis of the above definition and the information provided by the Investigator.

Table 26: Assigning Type of SAE Through Causality

Relationship	Description	Event Type
**Unrelated**	There is no evidence of any causal relationship	Unrelated SAE
**Unlikely**	There is little evidence to suggest there is a causal relationship (eg. the event did not occur within a reasonable time after administration of the trial medication). There is another reasonable explanation for the event (eg. the patient's clinical condition, other concomitant treatment).	Unrelated SAE
**Possible**	There is some evidence to suggest a causal relationship (eg. because the event occurs within a reasonable time after administration of the trial medication). However, the influence of other factors may have contributed to the event (eg. the patient's clinical condition, other concomitant treatments).	SAR
**Probable**	There is evidence to suggest a causal relationship and the influence of other factors is unlikely.	SAR
**Definitely**	There is clear evidence to suggest a causal relationship and other possible contributing factors can be ruled out.	SAR

If an SAE in an **ICON8** patient is considered to be related to trial treatment and drug is stopped or the dose modified, **Section A3.8 Protocol Treatment Discontinuation**, should be followed.

If an SAE in an **ICON8B** patient is considered to be related to trial treatment and drug is stopped or the dose modified, **Section B3.8 Protocol Treatment Discontinuation**, should be followed.

3.4.2 Notification

The MRC CTU (and relevant GCIG group if required) should be notified of all SAEs, SARs and SUSARs within **24 hours** of the investigator becoming aware of the event.

3.4.3 SAE Reporting Period

Investigators should notify the MRC CTU of all **ICON8 and ICON8B** SAEs occurring from the time of consent until 30 days after the last protocol treatment administration. SARs and SUSARS must be notified to the MRC CTU until trial closure even after protocol treatment has stopped. Any subsequent events that may be attributed to treatment should be reported to national regulatory bodies using their reporting system.

3.4.4 Notification Procedure (ICON8 and ICON8B):

1. The SAE form must be completed by an investigator or co-investigator (clinician named on the ICON8 or ICON8B signature list and delegation of responsibilities log who is responsible for the patient's care and delegated with the study task of SAE reporting), with due care being paid to the grading, causality and expectedness of the event as outlined above. In the absence of the responsible investigator, the form should be completed and signed by a member of the site trial

team and faxed or emailed as appropriate. The responsible investigator should subsequently check the SAE form, make changes as appropriate, sign and then re-fax to MRC CTU or GCIG group as soon as possible. The initial report must be followed by detailed, written reports as appropriate

The **minimum criteria** required for reporting an SAE are the trial number and date of birth, name of investigator reporting, the event, and why it is considered serious.

2. The SAE Form must be sent by fax to the MRC CTU or GCIG group using the fax numbers detailed below within 24 hours of the investigator's knowledge of the event

**UK (MRC/NCRI)**	**Fax: 020 7670 4818**
**All other GCIG groups**	**Fax: +44 (0) 20 7670 4818**

3. Follow-up: Patients must be followed-up until clinical recovery is complete and laboratory results have returned to normal or baseline, or until the event has stabilised. Follow-up should continue after completion of protocol treatment if necessary. Follow-up information should be noted on a further SAE form by ticking the box marked 'follow-up' and faxing to the MRC CTU and/or GCIG group as information becomes available. Extra, annotated information and/or copies of test results may be provided separately. The patient must be identified by trial number, date of birth and initials only. The patient's name should not be used on any correspondence and should be deleted from any test results

4. Staff should follow their institution's procedure for local notification requirements.

SAE REPORTING
Within 24 hours of becoming aware of an SAE, please fax a completed SAE form to the MRC CTU on:
Fax: +44 (0) 20 7670 4818

3.5 MRC CTU RESPONSIBILITIES

Medically qualified staff at the MRC CTU and/or the Chief Investigator (or a medically qualified delegate) will review all SAE reports received. The causality assessment given by the local investigator at the hospital cannot be overruled and in the case of disagreement, both opinions will be provided in any subsequent reports.

The MRC CTU is undertaking the duties of trial sponsor and is responsible for reporting of SUSARs and other SARs to the regulatory authorities (MHRA and competent authorities of other European member states and any other countries in which the trial is taking place) and research ethics committees, as appropriate. Fatal and life-threatening SUSARs must be reported to the competent authorities within 7 days of the MRC CTU becoming aware of the event; other SUSARs must be reported within 15 days.

All groups will be provided with the reports that they need to make the necessary regulatory submissions for their country (unless agreed that the MRC CTU will make these submissions).

The MRC CTU will also keep the Chief Investigators in each GCIG group informed of any safety issues that arise during the course of the trial, and through the GCIG groups will ensure that all other investigators are informed.

n the required format to Competent Authorities (regulatory authority and ethics committee).

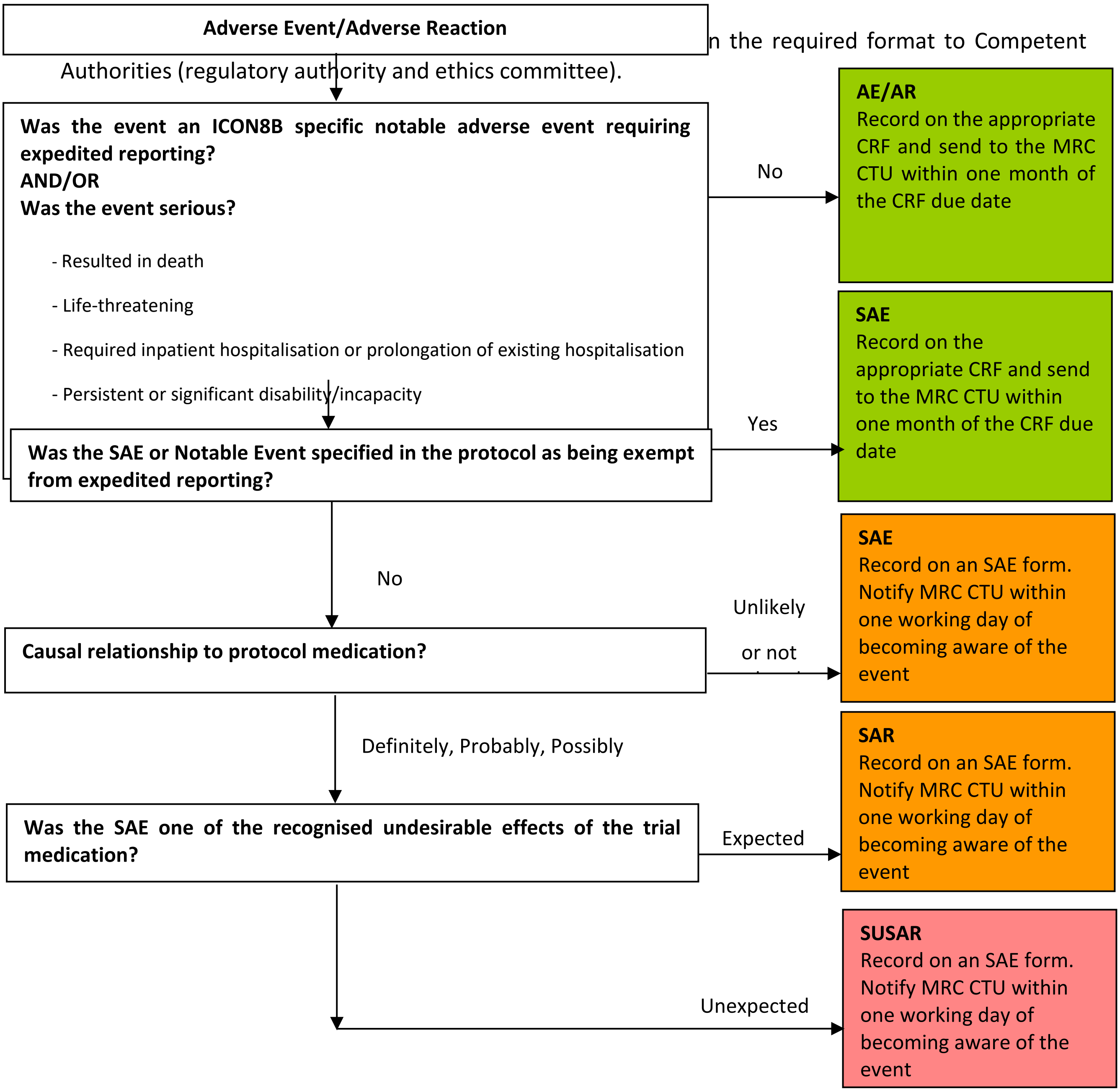

**CRF:** Case report form	**IB:** Investigator's brochure
**SAE:** Serious adverse event	**SAR:** Serious adverse reaction
**SPC:** Summary of product characteristics	**SUSAR:** Suspected unexpected serious adverse reaction

4 STATISTICAL CONSIDERATIONS

4.1 ICON8 STATISTICAL CONSIDERATIONS

4.1.1 METHOD OF RANDOMISATION

Patients will be randomly assigned to one of the three treatment groups in a 1:1:1 ratio using a minimisation method with a random element. The minimisation will be stratified by the stratification factors defined:

- GCIG group
- Disease stage
- Timing of surgery and outcome of surgery for IPS arm.

The selection of minimisation over block randomisation is to avoid any possible imbalance for the first and second stage analyses, which are to be conducted when only a small number of patients have been randomised.

4.1.2 OUTCOME MEASURES

ICON8 will have up to three planned staged analyses. The outcome measures for each stage are summarised below.

Table 15: Outcome measures of ICON8 stages

	Stage 1	Stage 2	Final Stage
**Primary**	Feasibility and safety of protocol treatment	9 month progression-free survival rate	Progression-free survival (PFS) and overall survival (OS)
**Secondary**	--	--	Toxicity<br>Quality of life<br>Health Economics

4.1.3 SAMPLE SIZE

ICON8 has a three-stage design and could be stopped at either of the first two stages. If a research arm is too toxic, not feasible to deliver or without minimal activity, recruitment to that arm can be stopped early or the regimen of the arm can be modified. If both research arms continue to the final stage, a total of 1485 patients will be randomised. The trial is designed to detect a hazard ratio of 0.75 in PFS and OS between the control arm (Arm 1) and each of the research arms (Arm 2 or Arm 3), with a 2.5% significance level and 90% power.

4.1.4 STAGE 1

Two analyses will be performed: the first when 50 women have been randomised to each arm and could have completed protocol defined treatment or been in the study at least 6 months after randomisation, whichever occurs first (**Stage 1A**); and the second when 50 women planned to undergo DPS have been randomised to each arm and could have completed protocol defined treatment or been in the study at least 6 months after randomisation, whichever occurs first (**Stage 1B**).

Feasibility will be assessed as the deliverability of 6 cycles of protocol treatment and safety will be assessed as the rate of any ≥grade 3 toxicity experienced per patient.

The detailed protocol treatment received and any toxicity experienced for all three arms will be presented to the Independent Data Monitoring Committee (IDMC) for review. The following is a guideline which may be used by the IDMC in their review:

1. It is expected that over 80% of women will complete 6 cycles of protocol treatment. In Arms 2 and 3, having completed a cycle is defined as having received at least 2 of the 3 weekly doses. With 50 patients randomised to each arm, the rate of deliverability of protocol treatment would be estimated with a standard error of <7%. If, at that stage, the number of women who have received fewer than 6 cycles of protocol treatment in Arm 2 or Arm 3 is less than 37, the lower limit of the 95% confidence interval would be less than 60% and the research regimens will be reconsidered. [No formal power calculation was carried out for this descriptive analysis, but it corresponds approximately to a Fleming/A'Hern design[45] with α=0.025, 1-β=0.86, p1=0.6, p2=0.8]

2. In addition, the IDMC will be asked to monitor the actual dose intensity of carboplatin and paclitaxel administered in each arm to exclude any significant effect on this from dose omissions/delays

3. For toxicity, an absolute difference in the proportion of patients experiencing any grade 3 or 4 toxicity between the control arm (Arm 1) and each of research arms (Arm 2 or Arm 3) of ≤15% would be considered acceptable. If the lower limit of the 95% confidence interval for the difference would exclude 15% estimated from these 50 patients, the research regimens may be reconsidered

4. In addition, the IDMC will be asked to monitor the proportion of patients in the research arms who develop febrile neutropenia (expected frequency 10% in control arm) and grade 2 sensory and motor neuropathy (expected frequency 20% in control arm) and advise if there is a clinically significant increase, for example, an increase of more than 10%, in these expected frequencies in either of the research arms

4.1.5 STAGE 2

The primary outcome measure for the second stage analysis is 9-month PFS rate (ie. the percentage of women who have not progressed or died at 9 months after randomisation) as it is the earliest meaningful activity outcome.

It is anticipated that the 9-month PFS rate will be approximately 68% in the control arm (based on the expected median survival of 16 months, and assuming exponential survival); a HR of 0.75 would translate to an expected 9-month PFS rate of ~75% in the research arms. Using A'Hern's method with a significance level of 0.05 (one-sided test) and 80% power, to test the 9-month PFS rate ≤60% vs. ≥75% in a research arm, the number of patients required in that arm is 62. As a guideline which may be used by the IDMC in their review, success would be demonstrated if ≥44 patients in a research arm are alive and progression free at 9 months; the lower 90% confidence limit would then exclude rates below 60%. The research arms would be reconsidered if the observed 9-month PFS rate is less than 60% i.e. if ≤37/62 patients are alive and free from progression at 9 months. The analysis will be performed 9 months after the first 62 patients have been randomised to each arm. Radiological assessment of disease status will be mandated at 9 months for the first 62 patients randomised each of three arms. The results of analyses for 9-month PFS rate and treatment completion together with profiles of general feasibility and toxicity for all three arms will be reviewed by the IDMC. Recruitment will continue during this period, and safety data will be reviewed at least annually by the IDMC.

9-month PFS rates will not be formally compared between the control arm and the research arms, or between the research arms themselves. However, the 9-month PFS rate will be recorded for the control arm to ensure that it is in keeping with results from previously published studies and in particular will be considered if the 9-month PFS rate in the research arm does not meet the defined criteria for success or reconsideration of the trial arms defined above.

The ratio of women randomised in the trial undergoing IPS to planned DPS will be monitored. The stage 1B analysis may be performed after the activity analysis if the number of women undergoing planned DPS is lower than anticipated.

4.1.6 FINAL STAGE

The final stage analysis is powered for progression-free survival (PFS) and overall survival (OS), in accordance with GCIG recommendations for first-line trials in ovarian cancer[46]. For both outcome measures, the planned main comparisons are between the control arm and each of the research arms (Arm 1 vs. Arm 2 and Arm 1 vs. Arm 3) with each comparison analysed using a 2-sided test at the 2.5% significance level, to account for the multiple comparisons. If both research arms are better (at a 0.05 significance level) than the control arm they will be compared with each other (Arm 2 vs. Arm 3) in an exploratory manner. This is a closed test, protecting the type 1 error but with more limited power than the main comparisons. PFS data will mature and be analysed first, approximately 2 years ahead of OS data. In view of the implicit ranking of PFS (the preferred outcome by GCIG definition) and OS, and the high degree of correlation between the two (all OS events are also PFS events) no further adjustment of the type 1 error is required.

The EORTC 55971 trial did not show any evidence of a difference in PFS or OS between women randomised to IPS or DPS, but PFS (and OS) was lower than that shown in many other first-line trials, suggesting that women with worse prognoses, in whom IPS was ruled out as the best option, had been selected for entry to the trial. We estimate that 70% of women will be enrolled having had IPS and in these women median OS is estimated as 36 months. We estimate that 30% of women will be enrolled with planned DPS, the median OS of these women is estimated as 30 months. Therefore sample size estimates assume a median overall survival of 34 months (36*0.7 + 30*0.3 = 34.2). Median PFS is estimated as 18 months for IPS and 12 months for DPS patients with an overall median PFS of 16 months (18*0.7 + 12*0.3 = 16.2). A hazard ratio of 0.75 will be targeted for both PFS and OS; with a two-sided significance level of 0.025 (because there will be 2 main comparisons against control) and 90% power. Assuming exponential survival and comparing the treatment arms using the standard unadjusted log-rank test a total of 602 events are required for each comparison. It is planned to recruit a total of 1485 women in 3 years, with 990 women in each comparison. The total number of PFS events could be observed with a further 1 year follow-up after the last patient randomised and the total number of OS events could be observed with a further 3 years follow-up from end of recruitment, a total of 6 years.

4.1.7 INTERIM MONITORING AND ANALYSES

An Independent Data Monitoring Committee (IDMC) will review data, unblinded by treatment group, on patients in the trial. The IDMC will meet at least annually during the accrual period and around the time that the planned first and second stage analyses are performed.

At each meeting, the IDMC will be asked to give advice on whether the accumulated data from the trial, together with results from other relevant trials, justifies continuing recruitment of further patients or further follow-up. A recommendation to discontinue recruitment, in all patients or in selected subgroups will be made only if the result is likely to convince a broad range of clinicians including participants in the trial and the general clinical community. Although no formal stopping rule is specified, this approach corresponds to a Haybittle-Peto stopping rule in which a p-value<0.001 for efficacy would typically be deemed as sufficient evidence to convince the general clinical community of a treatment benefit. Use of the Haybittle-Peto-type stopping rule in interim analyses will protect the overall type I error without further sample size adjustment. If a recommendation is made to continue, the IDMC will advise on the frequency of future reviews of the data on the basis of accrual and event rates. The IDMC will make recommendations to the Trial Steering Committee as to the continuation of the trial.

4.1.8 BRIEF ANALYSIS PLAN

Analyses will be performed on an intention-to-treat basis. Progression-free survival (PFS) is calculated from the date of randomisation to date of first progression or death from any cause, whichever occurs

first. If neither event is observed at the time of analysis, patients are censored at the date of last follow-up. Overall survival (OS) is calculated from the date of randomisation to date of death from any cause. Patients who are still alive at the time of analysis are censored at the date last known to be alive. The primary analysis will be a non-stratified log-rank test for the difference in the distribution of PFS and OS between each of research arms and the control arm. Cox model analyses adjusting for patient baseline characteristics will also be performed for PFS and OS. Interaction analysis by patient baseline characteristics on PFS and OS will be performed in an exploratory manner. Toxicity will be measured using the NCI Common Toxicity Criteria Adverse Events (CTCAE version 4.0) and the Mann-Whitney test will be used in the comparison. The first and second stage analyses will be mainly descriptive.

A detailed analysis plan will be developed and maintained.

4.2 ICON8B STATISTICAL CONSIDERATIONS

4.2.1 Method of Randomisation

Participants will be allocated to treatment groups centrally in a 1:1 ratio using a minimisation method with a random element. The allocation will be stratified by factors that include disease stage and timing of surgery.

4.2.2 Outcome Measures

Analyses will compare the three randomised arms on the following outcome measures:

4.2.3 Primary Outcome

- Progression-free survival (PFS)
- Overall survival (OS).

4.2.4 Secondary Outcomes

- Toxicity
- Quality of life
- Health economics.

4.2.5 Original Sample Size of ICON8B

The original design of the trial was with two efficacy comparisons, arm B3 vs B1 and arm B3 vs B2, and powered for both progression-free and overall survival. The targeted HR was 0.75 for both OS and PFS; this is the approximate HR found by addition of bevacizumab to standard chemotherapy in the ICON7 high risk subgroup, and also from dose fractionation compared to standard chemotherapy in JGOG 3016, and so represents a feasible effect in the absence of target saturation, as well as the minimum effect likely to change practice. For power of 90%, and with 2-sided alpha of 2.5% (in view of the 2 primary efficacy comparisons), the trial aimed to randomise 1170 patients (≈390 patients per arm) over 4 years. We anticipated 600 PFS events approximately 2 years after the last randomisation, giving 90%

power to detect the target PFS HR. After a further 2-3 years of follow-up, we expected to observe 460 deaths, giving 80% power for the targeted OS HR.

For each of the two superiority comparisons, arm B3 vs B1 and arm B3 vs B2, the PFS and OS rates for the control arms from the bevacizumab arm of the ICON7 high risk subgroup, and the bevacizumab maintenance arm of GOG218 were used, using the higher rates observed in either trial at years 1-3 for conservatism (PFS rates at years 1, 2, 3 and 5 of 75%, 28%, 20% and 15%; OS rates at years 1-8 of 90%, 77%, 58%, 45%, 37%, 30%, 26%, 25% respectively).

To show non-inferiority of arm B2 over arm B1, we assumed the same underlying PFS rate and wished to demonstrate that arm B2 retains at least 50% of the additional benefit that bevacizumab has shown over standard chemotherapy, representing this by a non-inferiority bound for the PFS HR of 0.87. With a one-sided significance level of 2.5%, and 90% power, 2500 patients would be required to observe 2168 events and we would expect an accrual period of 9 years in total with 2 years follow-up prior to analysis. If the two arms are truly equivalent, the 800 patients we plan to accrue to these arms would have 90% power to exclude a HR of ~0.77. Therefore, in the original design of ICON8B, if arm B3 failed to show superiority over arms B1 and B2, we planned to increase power for this comparison through a network meta-analysis incorporating indirect comparisons. Together with any further trials available at the time of the proposed analysis, GOG218 and ICON7 high risk subgroup would have provided one estimate; JGOG 3016 and ICON8 arm 1 vs arm 2 (equivalent high risk subgroup) would provide the second estimate, potentially supplemented by patients randomised into GOG262 who did not receive bevacizumab. The same set of trials would also allow further assessment of potential heterogeneity when bevacizumab is added to a background of standard vs dose dense chemotherapy.

4.2.6 Revised Sample Size of ICON8B

The sample size of ICON8B was reviewed following the suspension of recruitment to arm B2. The main change was to a 2-sided alpha of 5%, with ICON8B becoming a two-arm trial the adjustment for multiple testing was no longer necessary. Other sample size parameters remained as above (targeted HR of 0.75, recruitment period of 4 years. The same PFS and OS rates that were used in the original sample size calculation as described above were used (PFS rates at years 1, 2, 3 and 5 of 75%, 28%, 20% and 15%; OS rates at years 1-8 of 90%, 77%, 58%, 45%, 37%, 30%, 26%, 25% respectively).

The trial now aims to recruit 660 patients over four years (≈330 in each arm), and would expect to see 509 PFS events two years after the last randomisation, giving 90% power to detect the target PFS HR. After a further 3 years, we would expect to have observed 435 deaths, giving 85% power for the targeted OS HR.

4.2.7 Interim Monitoring & Analyses

The same independent Data Monitoring Committee (IDMC) will monitor both ICON8 and ICON8B, with meetings to review the latter at approximately at 1-year intervals after start of enrolment. In addition, an IDMC meeting may be requested by IDMC members, the Chief Investigator or the TMG at any time.

At each meeting, the IDMC will be asked to give advice on whether the accumulated data from the trial, together with results from other relevant trials, justifies continuing recruitment of further patients or further follow-up. A recommendation to discontinue recruitment, in all patients or in selected subgroups will be made only if the result is likely to convince a broad range of clinicians including participants in the trial and the general clinical community. Although no formal stopping rule is specified, this approach corresponds to a Haybittle-Peto stopping rule in which a p-value<0.001 for efficacy would typically be deemed as sufficient evidence to convince the general clinical community of a treatment benefit. Use of the Haybittle-Peto-type stopping rule in interim analyses will protect the overall type I error without further sample size adjustment. If a recommendation is made to continue, the IDMC will advise on the frequency of future reviews of the data on the basis of accrual and event rates. The IDMC will make recommendations to the Trial Steering Committee as to the continuation of the trial.

To monitor any complications arising from the combination of DPS and bevacizumab, a safety analysis will be performed and the IDMC will meet when 50 DPS patients have been randomised in each arm and completed chemotherapy, approximately six months after randomisation. This would give approximately 100 DPS patients treated with bevacizumab, and 50 without. Unless otherwise specified, the calculations below are based on assessment of event rates in the bevacizumab arms (B1 and B3) combined; rates in arm B2 will also be calculated to verify assumptions about control group rates, but at this stage there would be insufficient power to base conclusions on direct comparisons of arms.

Perioperative events that may potentially be increased by the use of bevacizumab and require specific assessment are haemorrhage (grade 3+), fistulae (grade 2+), GI perforation (grade 2+) and thrombotic events (grade 3+). We anticipate approximately 10% of patients on the non-bevacizumab arm would report at least one of these complications. If there were good evidence that the rate amongst patients receiving bevacizumab was more than 50% higher, the nature and timing of bevacizumab use in DPS patients may need to be reconsidered. The proportion of bevacizumab patients with at least one of these events reported will be calculated, together with a 90% confidence interval; if ≥22/100 patients have at least one of the specified events, the lower limit of the confidence interval limit would lie above 15%; this would indicate a possible need to reconsider the use and timing of bevacizumab in DPS patients. If data from the trial arm not containing bevacizumab (arm B2) suggest a lower event rate eg 6%, or higher eg 16%, the corresponding number of events to be observed in the bevacizumab arm that would indicate an unacceptable event rate would be 15/100 and 32/100 respectively.

Wound healing complications may be increased by the use of bevacizumab; a maximum rate of 10% of DPS patients with wound complications of grade 2 or higher would be accepted; if ≥16/100 patients with these events are observed, the lower 90% confidence limit would lie above 10% and this may also indicate a possible need to reconsider the use and timing of bevacizumab in DPS patients.

In addition, the median time to re-starting chemotherapy after DPS, and the proportion of patients not re-starting chemotherapy (excluding those in whom DPS takes place after cycle 6) will be compared in the bevacizumab and non-bevacizumab containing arms. This is expected to be ~4 weeks (SD 9 days) in arm 2 based on data from CHORUS and ICON8; any additional delay in the bevacizumab arms is likely to reflect the impact of events subject to the monitoring described above. However, in the absence of unacceptable event rates, time to re-starting chemotherapy may reflect the cumulative effect of lower grade toxicities, and therefore may provide a sensitive indicator of potential problems; an increase in the average time to restart chemotherapy of 14 days (from 30-44) would be of clinical concern, and could be detected with >95% power (2-sided α 0.05).

In addition to the planned stage 1 analysis, the number of notable events will also be monitored on a monthly basis. The rate of haemorrhage (G3+), fistulae, GI perforation and thrombotic events will be calculated in the bevacizumab arms combined, along with a 90% confidence interval. If the lower bound of this confidence interval excludes 15%, then the IDMC will be contacted to discuss the use of bevacizumab in DPS patients.

The rate of GI perforation events will also be assessed individually as well as in the group mentioned above. A 90% confidence interval will be calculated and if the lower bound of this excluded 5%, then the IDMC will be contacted.

Following the stage 1 analysis, the need to continue this monthly check will be discussed with the IDMC, with regards to the frequency the check is carried out.

4.2.8 Analysis Plan (Brief)

The analyses will be described fully in the Statistical Analysis Plan, briefly:

Efficacy analyses will be performed on an intention-to-treat basis. Arm B3 will be tested against arm B1 for superiority. Any patients that were recruited to arm B2 prior to the suspension of this arm will be still be followed up, and their data will be analysed in a descriptive manner.

Progression-free survival (PFS) will be calculated from the date of randomisation to date of first progression or death from any cause, whichever occurs first. Overall survival (OS) will be calculated from the date of randomisation to date of death from any cause. The primary analysis will be a non-stratified log-rank test for the difference in the distribution of PFS and OS between each research arm and the control arm. Cox models adjusting for patient baseline characteristics will also be performed for PFS and OS. If evidence is found of non-proportional hazards a restricted means analysis will provide the primary measure of effect. Interaction analysis by patient baseline characteristics on PFS and OS will be performed in an exploratory manner. Toxicity will be measured using the NCI Common Toxicity Criteria Adverse Events (CTCAE version 4.0).

4.3 QUALITY OF LIFE ANALYSIS

The impact of the two trial treatment strategies on quality of life (QoL) is a secondary end-point of ICON8B.

Patient reported QoL will be assessed using cancer-specific questionnaires which are included in the **Quality of Life CRFs**. The questionnaires used are the EORTC QLQ OV-28, which incorporates the EORTC QLQ C-30,[43] and the EQ-5D[44].

Use of these instruments is standard for first-line chemotherapy trials in ovarian cancer. The main focus of the QoL analysis will be on symptoms related to ovarian cancer and trial treatments, and questions on overall health and overall QoL. A detailed plan for the analysis of QoL data will be developed. Incorporation of the EQ-5D instrument facilitates the expression of health related quality of life (HRQL) in terms of ‘utilities’ which are used to estimate patients’ quality-adjusted survival duration.

5. QUALITY ASSURANCE & CONTROL

5.1 RISK ASSESSMENT

The Quality Assurance (QA) and Quality Control (QC) considerations have been based on a formal Risk Assessment, which acknowledges the risks associated with the conduct of the trial and how to address them with QA and QC processes. QA includes all the planned and systematic actions established to ensure the trial is performed and data generated, documented and/or recorded and reported in compliance with the principles of ICH GCP and applicable regulatory requirements. QC includes the operational techniques and activities done within the QA system to verify that the requirements for quality of the trial-related activities are fulfilled. This Risk Assessment, which has led to the development of other Quality Management Documentation (e.g. Monitoring plan, Safety Management Plan and Data Management Plan)has been reviewed by the Quality Management Advisory Group (QMAG) and the Research Governance Committee (RGC). The RGC has approved the Risk Assessment.

The MRC CTU has performed a risk assessment to assess the impact of trial participation on the rights and safety of patients, the reliability of trial results and the impact of trial results on the research institution leading the trial. This has guided the development of procedures in the trial with respect to informed consent, confidentiality, trial monitoring and audit.

5.2 MONITORING AT THE MRC CTU

The MRC CTU will conduct day-to-day central monitoring of the trial. MRC CTU staff will:

1. Perform visual checks that CRFs are completed with no missing items or obvious inconsistencies
2. Check that CRFs are completed by authorised persons
3. Perform data entry and identify missing or inconsistent data and follow-up with groups/sites for resolution by performing data compliance checks as detailed in the trial Data Management Plan.
4. Program database plausibility checks for validity and consistency of data
5. Identify and address random or systematic errors which arise and review recruitment rates
6. Review the first completed prescription for the first patient in each arm at each site (only required for new sites, sites already participating in ICON8 do not need to provide this during site approval for ICON8B).
7. Review of anonymised consent forms for each patient.

Other essential trial issues, events and outputs will be detailed in the Monitoring, Data Management and Safety Management Plan that are based on the trial-specific Risk Assessment.

5.3 ON-SITE MONITORING

Clinical site monitoring will be performed according to the ICON8/ICON8B Monitoring Plan.

Sites co-ordinated by the collaborating GCIG groups may follow their own national monitoring plans after discussion with the TMG (if they are different to those set down in the ICON 8/8B Monitoring Plan).

Monitors will verify adherence to the protocol and the completeness, consistency and accuracy of a selection of the data being entered on Case Report Forms (CRFs). Any data recorded directly on the CRFs (ie. no prior written or electronic record of data), may be considered to be source data.

Monitors will require access to all patient medical records including, but not limited to, laboratory test results and surgery, pathology and radiology reports and supporting documents. Monitors will also require access to pharmacy records relating to administration of chemotherapy. The principal investigator (or deputy) should work with the monitor to ensure that any problems detected are resolved.

The frequency, type and intensity for routine monitoring and the requirements for triggered monitoring will be detailed in the ICON8/8B Monitoring Plan. This plan will also detail the procedures for review and sign-off.

5.4 DATA QUALITY ASSURANCE

The data collected will be entered into the trial database from the original CRF received from the site. The site will retain a copy of the CRF.

All data recorded in each patient's CRF will be entered onto the ICON8 clinical trial database at the MRC CTU.

A comprehensive validation check program will identify illogical and/or inconsistent data, data chases will be performed regularly as detailed in the trial Data Management Plan. Trained data management personnel will review validation check reports, correcting any data entry errors. If investigator input is required to clarify or correct any missing, ambiguous or inconsistent data, the data manager will generate a Data Clarification Form (DCF). The data manager will send this form to the relevant GCIG group, who will forward it to the investigator for completion. When the completed DCF is returned to the data manager, the data on the clinical database will be corrected accordingly.

Data return rate tables will be run from the database following each data chase which will detail by site; number of patients randomised, number and percentage of missing forms. See Data Management Plan and Return Rate Policy for further information.

5.4.1 CONFIDENTIALITY OF TRIAL DOCUMENTS AND PATIENT RECORDS

We plan to follow the principles of the UK DPA regardless of the countries where the trial is being conducted.

The investigator must assure that patient's anonymity will be maintained and that their identities are protected from unauthorised parties. Patients will not be identified on CRFs by their names, but by an identification code. The investigator should keep a patient enrolment log showing codes, names and addresses.

5.4.2 DIRECT ACCESS TO PATIENT RECORDS

Participating investigators should agree to allow trial-related monitoring, including audits, ethics committee review and regulatory inspections by providing direct access to source data and documents as required. Patients' consent for this must be obtained.

5.4.3 PATIENT CONFIDENTIALITY

The MRC CTU is registered under the UK Data Protection Act to hold data as required for trial purposes. Trial databases will be held by MRC CTU. Patients will be allocated a unique trial number that will link all of the clinical information held about them on the trial databases. It will also be used in all correspondence with participating clinical trial sites. Anonymised data will be shared on reasonable request however sensitive patient identifiable data will not be shared under any circumstances. At no point in presentations or publications of trial data will individual patients be identified.

6 REGULATORY & ETHICAL ISSUES

6.1 COMPLIANCE

6.1.1 Regulatory Compliance

The trial will be conducted in compliance with the approved protocol, the Declaration of Helsinki 1996, the principles of Good Clinical Practice (GCP), Commission Directive 2005/28/EC with the implementation in national legislation in the UK by Statutory Instrument 2004/1031 and subsequent amendments, the UK Data Protection Act (DPA number: Z5886415), and the National Health Service (NHS) Research Governance Framework for Health and Social Care (RGF). International sites will comply with the principles of GCP as laid down by the ICH topic E6 (Note for Guidance on GCP), Commission Directive 2005/28/EC (the European Directive 2001/20/EC [where applicable]) and applicable national regulations.

6.1.2 Site Compliance

The site will comply with the above and international sites will comply with the principles of GCP as laid down by the ICH topic E6 (Note for Guidance on GCP), Commission Directive 2005/28/EC (the European Directive 2001/20/EC [where applicable]) and applicable national regulations. An agreement will be in place between the site and the MRC CTU, setting out respective roles and responsibilities.

The site will inform the Trials Unit as soon as they are aware of a possible serious breach of compliance, so that the Trials Unit can report this breach if necessary within 7 days as per the UK regulatory requirements. For the purposes of this regulation, a 'serious breach' is one that is likely to affect to a significant degree:

1. The safety or physical or mental integrity of the subjects in the trial, or
2. The scientific value of the trial.

6.1.3 Data Collection & Retention

CRFs, clinical notes and administrative documentation should be kept in a secure location (for example, locked filing cabinets in a room with restricted access) and held for 25 years after the end of the trial. During this period, all data should be accessible to the competent or equivalent authorities, and the Sponsor with suitable notice. The data may be subject to an audit by the competent authorities.

6.2 ETHICAL CONDUCT OF THE STUDY

6.2.1 Ethical Considerations

This is a randomised controlled trial. Therefore, neither the patient nor the physicians will be able to choose the patient's treatment. Treatment will be allocated randomly according to a computer-generated list. This is to ensure that the groups of patients receiving each of the different treatments are similar.

GCIG participating groups should consider whether local funding sources are available to meet patients' excess travel costs associated with participation in the trial.

6.2.2 ICON8 Ethical Considerations

In the ICON8 pathway, compared with the control arm 1, patients randomised to the research arms 2 or 3 will have to attend hospital weekly during therapy for a total of 12 (IPS) or 11 additional visits (DPS). Patients in the research arms will have additional blood tests performed to ensure that it is safe to continue on weekly treatment. All other investigations and visits are as per standard of care.

6.2.3 ICON8B Ethical Considerations

As per ICON8, patients in ICON8B randomised to the dose-fractionated control arm B2 or the research arm B3 will have to attend hospital weekly during the chemotherapy phase of treatment for a total of 12 (IPS patients) or 11 additional visits (DPS patients). Due to the treatment scheduling patients in arms B2 and B3 will have additional blood tests performed to ensure that it is safe to continue on weekly treatment (12 extra blood tests for IPS patients, 11 extra blood tests for DPS patients).

Although licenced for use in ovarian cancer, bevacizumab is not standard of care in all institutions for first line treatment. Patients in arms B1 and B3 will require additional hospital visits during the maintenance phase to receive bevacizumab infusions compared to patients in arm B2 not receiving bevacizumab who will attend 6 times in total during the maintainance phase. IPS patients in arms B1 and B2 will attend hospital 12 times during the maintenance phase, DPS patients will attend hospital 13 times (if they receive 5 bevacizumab doses during the chemotherapy phase).

Total treatment time will be 18 weeks for IPS patients in arm B2 compared to 54 weeks for IPS patients in arms B1 and B3 due to maintenance bevacizumab treatment (if treatment is given with no inturruptions). For DPS patients, in arm B2 total treatment time will be 21 weeks, compared to 66 weeks in arms B1 and B3 due to maintenance bevacizumab treatment, assuming that DPS is performed on cycle 3 day 22 with a 3-4 week post-op recovery period. All other investigations and visits are as per standard of care.

Patients in arms B1 and B3 may be at risk of bevacizumab specific toxicity including hypertension and proteinuria. Because the ICON8B patient population will have either had or be planned to undergo major surgery there is a risk of bevacizumab surgery specific related toxicity, including wound healing complications and haemorrhage. These risks have been balanced against the potential benefit of bevacizumab and one of the aims of the study is to assess the safety of neo-adjuvant bevacizumab in patients undergoing DPS. Within ICON8B, bevacizumab will be given at a 7.5mg/kg dose compared to the higher 15mg/kg dose on the licensed specification. The lower study dose is based on findings from the ICON7 study which showed the 7.5mg/kg dose still to be effective.

6.3 ETHICAL APPROVAL

The protocol has the appropriate national research ethics committee approval for the countries in which it will be conducted.

Before initiation of the trial at each clinical site, the protocol, all informed consent forms, and information materials to be given to the prospective participant will be submitted to each ethics committee for approval. Any further amendments will be submitted and approved by each ethics committee.

Prior to allowing randomisation of any patient, each clinical centre in the UK must obtain local R&D approval including approval of the local patient information sheet. Each patient's consent to participate in the trial should be obtained after a full explanation has been given of the treatment options, including the conventional and generally accepted methods of treatment. International sites should refer to their Group Specific Operating Instructions for requirements prior to first patient randomisation.

The right of the patient to refuse to participate in the trial without giving reasons must be respected. After the patient has entered the trial, the clinician must remain free to give alternative treatment to that specified in the protocol, at any stage, if he/she feels it to be in the best interest of the patient. However, the reason for doing so should be recorded and the patient will remain within the trial for the purpose of follow-up and data analysis according to the treatment option to which they have been allocated. Similarly, the patient must remain free to withdraw at any time from the protocol treatment and trial follow-up without giving reasons and without prejudicing her further treatment.

A statement of the MRC policy on ethical considerations in clinical trials of cancer therapy, including the question of informed consent, is available from the MRC Head Office web site http://www.mrc.ac.uk/

6.4 COMPETANT AUTHORITY APPROVALS

This protocol will be reviewed by/submitted to the national competent or equivalent authority/MHRA/FDA etc, as appropriate in each country where the trial will be run.

This is a Clinical Trial of an Investigational Medicinal Product (IMP) as defined by the EU Directive 2001/20/EC.

In the UK, ICON8 has been registered with the MHRA and has been granted a Clinical Trial Authorisation (CTA). The CTA reference is 2010-022209-16. The EudraCT number for the trial is 2010-022209-16.

The progress of the trial and safety issues will be reported to the competent authority, regulatory agency or equivalent in accordance with local requirements and practices in a timely manner.

Safety reports, including expedited reporting and SUSARS will be submitted to the competent authority in accordance with each authority's requirements in a timely manner.

6.5 OTHER APPROVALS

The protocol will be submitted by those delegated to do so to the relevant R&D department of each participating site in the UK or to other local departments for approval as required in each country. A copy of the local R&D approval (or other relevant approval as above) and of the PIS and Consent Form (CF) on local headed paper should be forwarded to the MRC CTU before patients are entered.

Each participating site receiving funding or support from the US government will obtain a Federalwide Assurance (FWA).

6.6 REGULATORY REQUIREMENTS FOR PATIENT ENROLMENT

Investigators may not enrol patients to this trial without:

- The necessary notification or approval of the protocol and any amendments by the competent authority of their country (in accordance with local regulations)

- The approval of the protocol and any amendments by their Ethics Committee/Institutional Review Board (in accordance with local regulations).

6.7 TRIAL CLOSURE

The trial will be considered closed for regulatory purposes after data on overall survival are sufficiently mature for the primary publication. Further observational follow-up of all patients enrolled in the trial may continue indefinitely. This will initially be via hospitals and clinics, but in the longer term may exploit national registers where consent for this has been given.

6.8 ARCHIVING

The documents which individually and collectively permit evaluation of the conduct of a clinical trial and the quality of the data produced are defined as essential documents.

These documents service to demonstrate the compliance of the investigator, sponsor and monitor the standards of GCP and with applicable regulatory requirements. They should be filed in an organised way that will facilitate management of the clinical trial, audit and inspection (Trial Master File).

ICON8 Trials Programme essential documents must be retained (archived) for 25 years to allow for audit and inspection by regulatory authorities and should be readily available upon request.

An archive index/log should be maintained to record all essential documents that have been entered into the archive, and to track and retrieve documents on loan from the archive.

The investigator should make the MRC CTU (Sponsor/trial organisers) aware of the storage arrangements for the documents to be stored at investigator sites. If the investigator becomes unable to store their essential documents, the MRC CTU should be notified in writing so that alternative storage arrangements can be agreed. If the investigator is no longer able to maintain custody of their essential documents, the MRC CTU should be notified in writing and the investigator/institution see to it that appropriate arrangements can be made.

6.9 DESTRUCTION OF ESSENTIAL DOCUMENTS

The reasons for destruction of essential documents should be documented and signed by a person with appropriate authority. This record should be retained for a further five years from the date that the essential documents were destroyed. The MRC CTU will notify investigators in writing when their trial records can be destroyed.

7 SPONSORSHIP AND INDEMNITY

The ICON8 Trials Programme is a GCIG trial. The trial will be performed in accordance with the national laws and regulations of the countries in which it is being performed and applicable European laws and regulations. The MRC/NCRI is acting as the lead GCIG group and MRC, on behalf of the lead group, is the overall Sponsor of the trial worldwide. Where required, a legal representative of the participating GCIG group will be appointed who will fulfil the functions of Sponsor in that country.

University College London (UCL) holds insurance against claims from participants for injury caused by their participation in this clinical trial. Participants may be able to claim compensation if they can prove that UCL has been negligent. However, as this clinical trial is being carried out in a hospital, the hospital continues to have a duty of care to the participant of the clinical trial. UCL does not accept liability for any breach in the hospital's duty of care, or any negligence on the part of hospital employees. This applies whether the hospital is an NHS Trust or otherwise. Participants may also be able to claim compensation for injury caused by participation in this clinical trial without the need to prove negligence on the part of UCL or another party. Participants who sustain injury and wish to make a claim for compensation should do so in writing in the first instance to the Chief Investigator, via the ICON8 Trial Manager, who will pass the claim to the UCL's Insurers, via the UCL's office.

Hospitals selected to participate in this clinical trial must provide clinical negligence insurance cover for harm caused by their employees and a copy of the relevant insurance policy or summary can be provided on request.

MRC, as overall sponsor is responsible for ensuring that for each GCIG group there are appropriate arrangements for indemnity and/or clinical trial insurance as required by national regulatory authorities. Insurance certificates should be held for all GCIG groups where insurance is required under national laws. MRC will ensure that this is in place before a group is allowed to begin randomising patients.

8 ANCILLARY STUDIES

8.1 TRANSLATIONAL RESEARCH

The design of the ICON8 Trials Programme provides the opportunity to obtain several valuable tissue collections including:

1. Paired FFPE tumour samples obtained before and after the administration of platinum and taxane-containing chemotherapy regimens in patients undergoing delayed primary surgery. These samples will enable assessment of molecular correlates of platinum and taxane sensitivity and resistance

2. Surgical FFPE tumour samples from primary and metastatic sites. These samples will allow assessment of molecular heterogeneity

3. Baseline genomic DNA sample that would be available for pharmacogenomic analysis

4. Longitudinal plasma collection. This will allow the assessment of circulating biomarkers of treatment sensitivity and resistance.

Sample collection will be performed at 3 levels:

1. FFPE tissue samples only (all centres expected to participate)
2. FFPE tissue and whole blood
3. FFPE tissue, whole blood and longitudinal plasma sampling.

Centres will choose to participate at Level 1, 2 or 3 depending on availability of appropriate expertise and facilities for the processing and storage of samples.

Further information on **TRICON8**, the translational research sub-study of **ICON8**, is given in **Appendix 11** and detailed instructions on sample processing, labelling, handling, storage and shipment of collected specimens is in the TRICON8 manual.

Further information on **TRICON8B**, the translational research sub-study of **ICON8B**, is given in **Appendix 12** and detailed instructions on sample processing, labelling, handling, storage and shipment of collected specimens is in the TRICON8B manual.

9 FINANCE

The ICON8 Trials Programme is a Gynaecologic Cancer Intergroup trial. The MRC/NCRI Group is the lead group. The trial will be coordinated at and by the MRC Clinical Trials Unit at UCL in London.

The trial has public funding for UK sites from Cancer Research UK through the Clinical Trials Awards and Advisory Committee (Award No's C1489/A12127 (ICON8) and CA1489/A17092 (ICON8B)), and will also be supported by Medical Research Council core funding.

Funding arrangements for international sites will be detailed in their GCIG group-specific appendix.

TRICON8 has public funding for UK sites from Cancer Research UK through the Clinical Trials Awards and Advisory Committee (Award No C608/A15871).

10 OVERSIGHT & TRIAL COMMITTEES

There are a number of committees involved with the oversight of the trial. These committees are detailed below, and the relationship between them expressed in **Figure 14**.

10.1 TRIAL MANAGEMENT TEAM (TMT)

The Trial Management Team will be made up of the Chief Investigator(s) (CIs) and the MRC CTU who are responsible for the day to day running of the trial as detailed in trial SOPs. They will work together as the Trial Management Team. ICON8/8B Team Representatives from MRC CTU will compromise the core trial management team responsible for non-medical day-to-day operational and quality management. The MRC CTU will prepare reports for the TMG, TSC and IDMC, including interim analyses, and will make safety and progress reports to the main REC and Medicines and Healthcare Products Regulatory Agency (MHRA) and to other groups for their regulatory and ethics requirements as needed.

10.2 GCIG ICON8 TRIAL MANAGEMENT GROUP (TMG)

The Trial Management Group (TMG) will meet at least every six months by teleconference while trial patients are receiving protocol therapy and will aim to meet in person at least once per year. The TMG will advise the CIs and MRC CTU in the promotion and running of the trial. TMG members will review serious adverse events which have occurred in the trial at regular intervals as specified in the ICON8 Safety Management Plan. If there are specific safety concerns these may be raised with the TSC and IDMC. TMG members will include active trial investigators who represent their GCIG group and members with specific interests (eg. pharmacist, nurse, user representatives). A charter has been developed for this committee. Current members are listed in **Appendix 1**.

10.3 INDEPENDENT TRIAL STEERING COMMITTEE (TSC)

The MRC Gynaecology Trial Steering Committee has members who are independent of investigators and the MRC CTU while also including CTU staff working on the trial. It will provide overall supervision of the trial. It will meet at least annually, and will receive reports from the MRC CTU, TMG and IDMC.

10.4 INDEPENDENT DATA MONITORING COMMITTEE (IDMC)

The Independent Data Monitoring Committee (IDMC) is independent of investigators and the MRC CTU. The group will meet at least annually while patients are receiving trial treatment. The IDMC will review reports from the MRC CTU and give advice on continuing recruitment. There are planned staged analyses in this trial which will be reviewed by the IDMC. No other formal stopping rules for efficacy are planned. The IDMC will make recommendations to the TSC as to the continuation of the trial.

A charter for the IDMC has been developed and a copy is available from the MRC CTU on request. Details of data that will be provided to the IDMC at their meetings and monitoring are provided in the IDMC charter.

Figure 14: Diagram of Relationships between Trial Committees

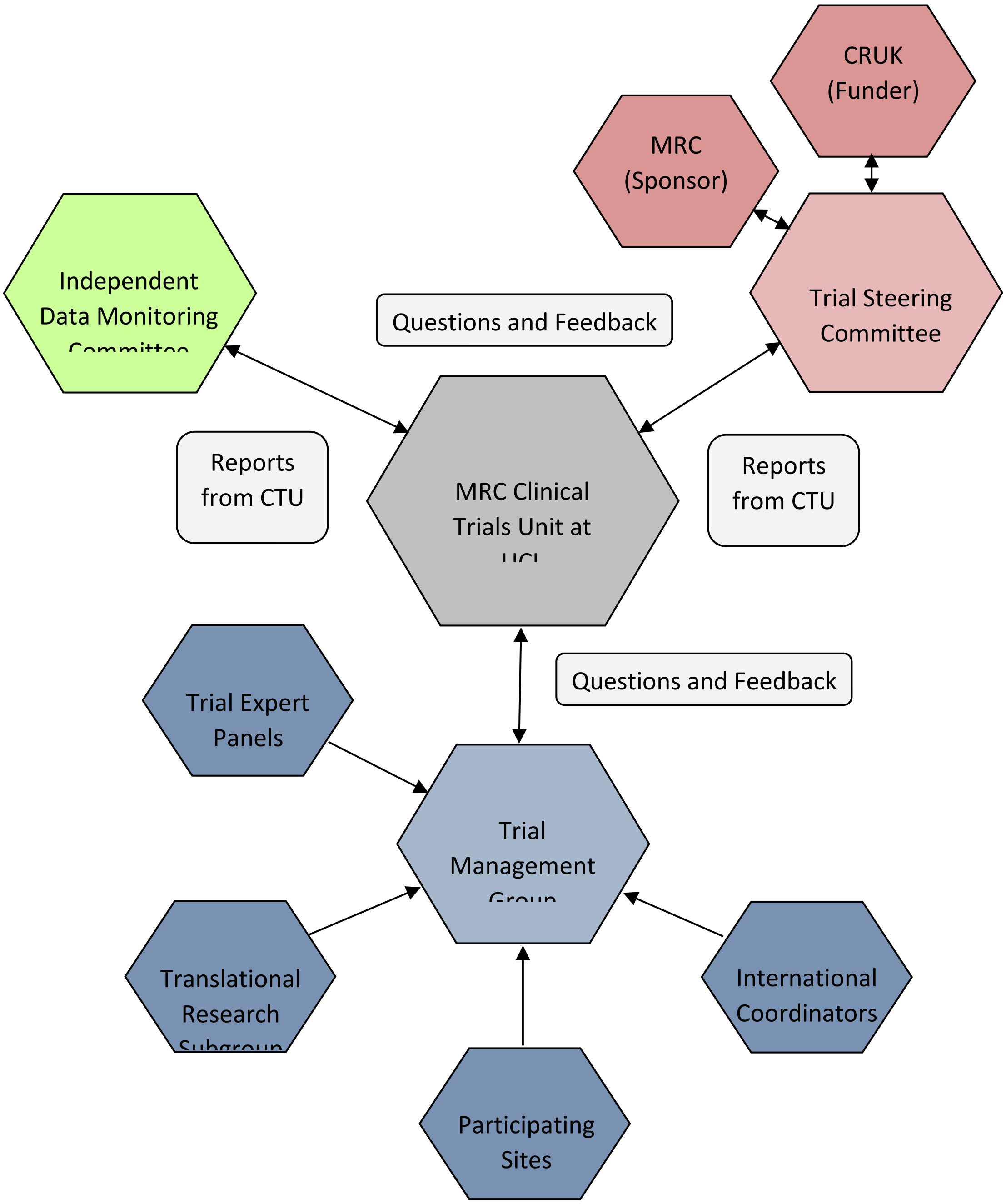

11 PUBLICATION

The data from all collaborating groups will be analysed together. Individual clinicians or groups must not publish data concerning their patients that are directly relevant to questions posed by the trial until the reports on the trial's main outcome measures has been published. Data will not normally be released externally prior to publication of the trial's main outcome measures. All requests for external data release will be approved by the Trial Steering Committee (TSC).

If the trial is stopped at any of the planned staged analyses or at any other time, trial results up to that point will be analysed and published as soon as possible.

For the ICON8 cohort, results of the stage 1 feasibility and safety analysis will be published. After the stage 2 analysis, advice will be sought from the TSC with regards to publication of those results. If recruitment continues to the planned final sample size, survival follow-up will continue until all randomised patients have completed protocol treatment and the results on overall survival have been published. The progression-free survival analysis is expected to occur 1 year after the last patient is randomised, and the overall survival analysis is expected to occur 3 years after the last patient is randomised. The results of the progression-free and overall survival analyses will be published separately, and as soon as possible after each analysis has occurred.

For the ICON8B cohort, results of the safety analysis in DPS patients will be published. If recruitment continues to the planned final sample size, survival follow-up will continue until all randomised patients have completed protocol treatment and the results on overall survival have been published. The progression-free survival analysis is expected to occur 1 year after the last patient is randomised, and the overall survival analysis is expected to occur 3 years after the last patient is randomised. The results of the progression-free and overall survival analyses will be published separately, and as soon as possible after each analysis has occurred.

The Writing Committee will be formed according to MRC CTU policy. The Trial Management Group (TMG) will form the basis of the Writing Committee and advise on the nature of publications and presentations.

For publications where there are no named authors, the paper will be published in the name of the ICON8 Trials Programme collaborators and members of the Writing Committee will be identified. If, on other publications, there are named authors these should include the trial's Chief Investigator(s), Lead Scientist(s), Statistician(s) and Clinical Operations staff involved in the trial.

Members of the TMG, TSC and IDMC will be listed with their affiliations in the acknowledgements section of the main publication. All publications shall include a list of participating clinicians. All publications should list CTU operations staff who contributed to the conduct of the trial.

12 PROTOCOL AMENDMENTS

Please check with the relevant GCIG group that you are using the most recent version of the ICON8 Protocol.

- Protocol Version 1.0 (22nd December 2010) was submitted to MHRA as part of the Clinical Trial Authorisation Application. Minor, non-substantial amendments were made to make version 2.0 (10th March 2011): submitted as part of the National Research Ethics Service application

- Protocol version 3.0 (31st May 2013) was submitted for ethical and regulatory approval as a substantial amendment due to the addition of ovarian carcinosarcoma to the eligibility criteria, addition of an extra quality of life assessment prior to delayed primary surgery, and changes to the translational research sub-study (TRICON8). There were also multiple minor changes for clarity

- Protocol version 4.0 (13th March 2014) was submitted for ethical and regulatory approval due to changes in the indemnity arrangements for the trial as a result of the MRC CTU becoming a university unit within UCL. There were also multiple minor changes for clarity

- Protocol version 5.0 (17th December 2015) was submitted for ethical and regulatory approval due to the addition of the ICON8B study cohort. The previous ICON8 protocol was placed onto the MRC protocol template V3.1. There were also multiple minor changes for clarity.

- Protocol version 6.0 (19th April 2016) was submitted for ethical and regulatory approval as a substantial amendment due to addition of multiple plasma sample timepoints to TRICON8B. There were also multiple minor changes for clarity and updates to the trial team and TMG member list.

- Protocol version 7.0 (26th June 2017) was submitted for ethical and regulatory approval as a substantial amendment to suspend recruitment to arm B2 of ICON8B following analysis of mature Progression-free survival data from the ICON8 trial. From 5th May 2017 ICON8B participants will be randomised into a 2-arm comparison study (arm B1 vs arm B3) as described in section B. The QoL timepoints in ICON8B have been reduced and the NHS England dose banding guidelines have been incorporated into the guidence regarding treatment administration in ICON8B. There were also multiple minor changes for clarity and updates to the trial team and TMG member list.

13 REFERENCES

1. Ferlay J, Shin HR, Bray F, Forman D, Mathers C, Parkin DM. GLOBOCAN 2008 Cancer Incidence and Mortality Worldwide: IARC CancerBase No. 10 [Internet]. International Agency for Research on Cancer; Lyon, France; 2008.

2. Parmar MKB, Adams M, Balestrino M, Bertelsen K, Bonazzi C, Calvert H, et al. Paclitaxel plus carboplatin versus standard chemotherapy with either single-agent carboplatin or cyclophosphamide, doxorubicin, and cisplatin in women with ovarian cancer: the ICON3 randomised trial. The Lancet. 2002; **360**(9332): 505 - 15.

3. Bookman MA, Brady MF, McGuire WP, Harper PG, Alberts DS, Friedlander M, et al. Evaluation of new platinum-based treatment regimens in advanced-stage ovarian cancer: a Phase III Trial of the Gynecologic Cancer Intergroup. J Clin Oncol. 2009; **27**(9): 1419-25.

4. Vergote I, Trope CG, Amant F, Kristensen GB, Ehlen T, Johnson N, et al. Neoadjuvant chemotherapy or primary surgery in stage IIIC or IV ovarian cancer. N Engl J Med. 2010; **363**(10): 943-53.

5. du Bois A, M. Quinn M, Thigpen T, Vermorken J, Avall-Lundqvist E, Bookman M, et al. 2004 consensus statements on the management of ovarian cancer: final document of the 3rd International Gynecologic Cancer Intergroup Ovarian Cancer Consensus Conference (GCIG OCCC 2004). Annals of Oncology. 2005; **16**(Supplement 8): viii7–viii12.

6. Pignata S, Scambia G, Savarese A, Sorio R, Breda E, Legge F, et al. Carboplatin (C) plus paclitaxel (P) versus carboplatin plus pegylated liposomal doxorubicin (PLD) in patients with advanced ovarian cancer (AOC): Final analysis of the MITO-2 randomized multicenter trial. J Clin Oncol (Meeting Abstracts). 2010; **28**(18_suppl): LBA5033-.

7. Teneriello MG, Gordon AN, Lim P, Janicek M. Phase III trial of induction gemcitabine (G) or paclitaxel (T) plus carboplatin (C) followed by elective T consolidation in advanced ovarian cancer (OC): Final safety and efficacy report. J Clin Oncol (Meeting Abstracts). 2010; **28**(18_suppl): LBA5008-.

8. Burger RA, Brady MF, Bookman MA, Walker JL, Homesley HD, Fowler J, et al. Phase III trial of bevacizumab (BEV) in the primary treatment of advanced epithelial ovarian cancer (EOC), primary peritoneal cancer (PPC), or fallopian tube cancer (FTC): A Gynecologic Oncology Group study. J Clin Oncol (Meeting Abstracts). 2010; **28**(18_suppl): LBA1.

9. Perren TJ, Swart AM, Pfisterer J, Ledermann JA, Pujade-Lauraine E, Kristensen G, et al. A phase 3 trial of bevacizumab in ovarian cancer. N Engl J Med. 2011; **365**(26): 2484-96.

10. Stark D, Nankivell M, Pujade-Lauraine E, Kristensen G, Elit L, Stockler M, et al. Standard chemotherapy with or without bevacizumab in advanced ovarian cancer: quality-of-life outcomes from the International Collaboration on Ovarian Neoplasms (ICON7) phase 3 randomised trial. The lancet oncology. 2013; **14**(3): 236-43.

11. Aghajanian C, Blank SV, Goff BA, Judson PL, Teneriello MG, Husain A, et al. OCEANS: a randomized, double-blind, placebo-controlled phase III trial of chemotherapy with or without bevacizumab in patients with platinum-sensitive recurrent epithelial ovarian, primary peritoneal, or fallopian tube cancer. J Clin Oncol. 2012; **30**(17): 2039-45.

12. Pujade-Lauraine E, Hilpert F, Weber B, Reuss A, Poveda A, Kristensen G, et al. AURELIA: A randomized phase III trial evaluating bevacizumab (BEV) plus chemotherapy (CT) for platinum (PT)-resistant recurrent ovarian cancer (OC). ASCO Meeting Abstracts. 2012; **30**(18_suppl): LBA5002.

13. Belotti D, Vergani V, Drudis T, Borsotti P, Pitelli MR, Viale G, et al. The microtubule-affecting drug paclitaxel has antiangiogenic activity. Clin Cancer Res. 1996; **2** 1843-9.

14. Kamat AA, Kim TJ, Landen CN, Lu C, Han LY, Lin YG, et al. Metronomic chemotherapy enhances the efficacy of antivascular therapy in ovarian cancer. Cancer Res. 2007; **67**(281–288).

15. Lau DH, Guo L, Gandara D, Young L, Xue L. Is inhibition of cancer angiogenesis and growth by paclitaxel schedule dependent? Anti-Cancer Drugs: 2004; **15** (9): 6.

16. Lau DH, Xue L, Young L, Burke PA, Cheung AT. Paclitaxel (Taxol): An Inhibitor of Angiogenesis in a Highly Vascularized Transgenic Breast Cancer. Cancer Biotherapy & Radiopharmaceuticals 1999; **14**(6): 5.

17. Milross CG, Mason KA, Hunter NR, Chung WK, Peters LJ, Milas L. Relationship of mitotic arrest and apoptosis to antitumor effect of paclitaxel. . J Natl Cancer Inst 1996; **88**: 1308–14.

18. Joerger M, Huitema AD, Richel DJ, Dittrich C, Pavlidis N, Briasoulis E, et al. Population pharmacokinetics and pharmacodynamics of paclitaxel and carboplatin in ovarian cancer patients: a study by the European organization for research and treatment of cancer-pharmacology and molecular mechanisms group and new drug development group. Clin Cancer Res. 2007; **13**(21): 6410-8.

19. Gilewski T, Norton L. Cytokinetics and breast cancer chemotherapy. In: Harris JR, Lippman ME, Morrow MM, editors. Diseases of the Breast. 1996 ed. Philadelphia, PA.: Lippincott-Raven; 1996. p. 751-68.

20. Abu-Rustum NR, Aghajanian C, Barakat RR, Fennelly D, Shapiro F, Spriggs D. Salvage weekly paclitaxel in recurrent ovarian cancer. Semin Oncol. 1997; **24**(5 Suppl 15): S15-62-S15-67.

21. Fennelly D, Aghajanian C, Shapiro F, O'Flaherty C, McKenzie M, O'Connor C, et al. Phase I and pharmacologic study of paclitaxel administered weekly in patients with relapsed ovarian cancer. J Clin Oncol. 1997; **15**(1): 187-92.

22. Kita T, Kikuchi Y, Takano M, Suzuki M, Oowada M, Konno R, et al. The effect of single weekly paclitaxel in heavily pretreated patients with recurrent or persistent advanced ovarian cancer. Gynecol Oncol. 2004; **92**(3): 813-8.

23. Markman M, Hall J, Spitz D, Weiner S, Carson L, Van Le L, et al. Phase II trial of weekly single-agent paclitaxel in platinum/paclitaxel-refractory ovarian cancer. J Clin Oncol. 2002; **20**(9): 2365-9.

24. Rosenberg P, Andersson H, Boman K, Ridderheim M, Sorbe B, Puistola U, et al. Randomized trial of single agent paclitaxel given weekly versus every three weeks and with peroral versus intravenous steroid premedication to patients with ovarian cancer previously treated with platinum. Acta Oncol. 2002; **41**(5): 418-24.

25. Rose PG, Smrekar M, Fusco N. A phase II trial of weekly paclitaxel and every 3 weeks of carboplatin in potentially platinum-sensitive ovarian and peritoneal carcinoma. Gynecol Oncol. 2005; **96**(2): 296-300.

26. Katsumata N, Yasuda M, Takahashi F, Isonishi S, Jobo T, Aoki D, et al. Dose-dense paclitaxel once a week in combination with carboplatin every 3 weeks for advanced ovarian cancer: a phase 3, open-label, randomised controlled trial. Lancet. 2009; **374**(9698): 1331-8.

27. Katsumata N1, Yasuda M, Isonishi S, Takahashi F, Michimae H, Kimura E. Long-term results of dose-dense paclitaxel and carboplatin versus conventional paclitaxel and carboplatin for treatment of

advanced epithelial ovarian, fallopian tube, or primary peritoneal cancer (JGOG 3016): a randomised, controlled, open-label trial. Lancet Oncol. 2013 Sep;14(10):1020-6

28. Cadron I, Leunen K, Amant F, Van Gorp T, Neven P, Vergote I. The "Leuven" dose-dense paclitaxel/carboplatin regimen in patients with recurrent ovarian cancer. Gynecol Oncol. 2007; **106**(2): 354-61.

29. Sharma R, Graham J, Mitchell H, Brooks A, Blagden S, Gabra H. Extended weekly dose-dense paclitaxel/carboplatin is feasible and active in heavily pre-treated platinum-resistant recurrent ovarian cancer. Br J Cancer. 2009; **100**(5): 707-12.

30. Bolanos M, Borrega P, Gonzalez-Beca R, Arranz JA, Velasco A, Perez MM, et al. Weekly paclitaxel/carboplatinum as first-line chemotherapy in late relapses of epithelial ovarian cancer. A preliminary report on side effects. Proc Am Soc Clin Oncol 20, (abstr 870); 2001; 2001. p. 870.

31. Kikuchi A, Sakamoto H, Yamamoto T. Weekly carboplatin and paclitaxel is safe, active, and well tolerated in recurrent ovarian cancer cases of Japanese women previously treated with cisplatin-containing multidrug chemotherapy. Int J Gynecol Cancer. 2005; **15**(1): 45-9.

32. Havrilesky LJ, Alvarez AA, Sayer RA, Lancaster JM, Soper JT, Berchuck A, et al. Weekly low-dose carboplatin and paclitaxel in the treatment of recurrent ovarian and peritoneal cancer. Gynecol Oncol. 2003; **88**(1): 51-7.

33. Dunton C. Phase II study of weekly paclitaxel and weekly carboplatinum in recurrent platinum sensitive ovarian cancer. Proc Am Soc Clin Oncol 22, (abstr 1876); 2003; 2003.

34. Safra T, Menczer J, Bernstein RM, Shpigel S, Matcejevsky D, Inbar MJ, et al. Combined weekly carboplatin and paclitaxel as primary treatment of advanced epithelial ovarian carcinoma. Gynecol Oncol. 2009; **114**(2): 215-8.

35. Pignata S, Breda E, Scambia G, Pisano C, Zagonel V, Lorusso D, et al. A phase II study of weekly carboplatin and paclitaxel as first-line treatment of elderly patients with advanced ovarian cancer. A Multicentre Italian Trial in Ovarian cancer (MITO-5) study. Crit Rev Oncol Hematol. 2008; **66**(3): 229-36.

36. Wu CH, Yang CH, Lee JN, Hsu SC, Tsai EM. Weekly and monthly regimens of paclitaxel and carboplatin in the management of advanced ovarian cancer. A preliminary report on side effects. Int J Gynecol Cancer. 2001; **11**(4): 295-9.

37. Sehouli J, Stengel D, Mustea A, Camara O, Keil E, Elling D, et al. Weekly paclitaxel and carboplatin (PC-W) for patients with primary advanced ovarian cancer: results of a multicenter phase-II study of the NOGGO. Cancer Chemother Pharmacol. 2008; **61**(2): 243-50.

38. Burg ME, Janssen JT, Ottevanger PB, Kerkhofs LG, Valster F, Stouthard JM, et al. Multicenter randomized phase III trial of 3-weekly paclitaxel/platinum (PC3w) versus weekly paclitaxel/platinum (PCw) induction therapy followed by PC3w maintenance therapy in advanced epithelial ovarian cancer (EOC). Proc Am Soc Clin Oncol 27, (abstr 5538); 2009; 2009.

39. Schuette W, Blankenburg T, Guschall W, Dittrich I, Schroeder M, Schweisfurth H, et al. Multicenter randomized trial for stage IIIB/IV non-small-cell lung cancer using every-3-week versus weekly paclitaxel/carboplatin. Clin Lung Cancer. 2006; **7**(5): 338-43.

40. Calvert AH, Newell DR, Gumbrell LA, O'Reilly S, Burnell M, Boxall FE, et al. Carboplatin dosage: prospective evaluation of a simple formula based on renal function. J Clin Oncol. 1989; **7**(11): 1748-56.

41. Barraclough LH, Field C, Wieringa G, Swindell R, Livsey JE, Davidson SE. Estimation of renal function -- what is appropriate in cancer patients? Clin Oncol (R Coll Radiol). 2008; **20**(10): 721-6.

42. Marx GM, Blake GM, Galani E, Steer CB, Harper SE, Adamson KL, et al. Evaluation of the Cockroft-Gault, Jelliffe and Wright formulae in estimating renal function in elderly cancer patients. Ann Oncol. 2004; **15**(2): 291-5.

43. Fayers PM. Interpreting quality of life data: population-based reference data for the EORTC QLQ-C30. Eur J Cancer. 2001; **37**(11): 1331-4.

44. Kind P. The EuroQoL instrument: an index of health related quality of life. 2 ed; 1996.

45. A'Hern RP. Sample size tables for exact single-stage phase II designs. Statistics in medicine. 2001; **20**(6): 859-66.

46. Stuart GC, Kitchener H, Bacon M, duBois A, Friedlander M, Ledermann J, et al. 2010 Gynecologic Cancer InterGroup (GCIG) consensus statement on clinical trials in ovarian cancer: report from the Fourth Ovarian Cancer Consensus Conference. Int J Gynecol Cancer. 2011; **21**(4): 750-5.

47. Benedet JL, Bender H, Jones H, 3rd, Ngan HY, Pecorelli S. FIGO staging classifications and clinical practice guidelines in the management of gynecologic cancers. FIGO Committee on Gynecologic Oncology. Int J Gynaecol Obstet. 2000; **70**(2): 209-62.

48. Oken MM, Creech RH, Tormey DC. Toxicity and response criteria of the eastern cooperative oncolocgy group. AM J Clin ONcol. 1982; **5**(6): 7.

49. Wright JG, Boddy AV, Highley M, Fenwick J, McGill A, Calvert AH. Estimation of glomerular filtration rate in cancer patients. Br J Cancer. 2001; **84**(4): 452-9.

50. Jelliffe RW. Letter: Creatinine clearance: bedside estimate. Ann Intern Med. 1973; **79**(4): 604-5.

51. Cockcroft D, Gault M. Prediction of creatinine clearance from serum creatinine. Nephron. 1976; **16**: 31-41.

52. Petru E, Luck HJ, Stuart G, Gaffney D, Millan D, Vergote I. Gynecologic Cancer Intergroup (GCIG) proposals for changes of the current FIGO staging system. Eur J Obstet Gynecol Reprod Biol. 2009; **143**(2): 69-74.

53. Eisenhauer EA, Therasse P, Bogaerts J, Schwartz LH, Sargent D, Ford R, et al. New response evaluation criteria in solid tumours: revised RECIST guideline (version 1.1). Eur J Cancer. 2009; **45**(2): 228-47.

54. Vergote I, Rustin GJ, Eisenhauer EA, Kristensen GB, Pujade-Lauraine E, Parmar MK, et al. Re: new guidelines to evaluate the response to treatment in solid tumors [ovarian cancer]. Gynecologic Cancer Intergroup. J Natl Cancer Inst. 2000; **92**(18): 1534-5.

55. Pujade-Lauraine E, Hilpert F, Weber B, Reuss A, Poveda A, Kristensen G et al. Bevacizumab Combined With Chemotherapy for Platinum-Resistant Recurrent Ovarian Cancer: The AURELIA Open-Label Randomized Phase III Trial. J Clin Oncol. 2014; 32: 1302-1308
56. Chan J, Brady MF, Penson R, Huang H, Birrer MJ, Walker JL, et al. Weekly vs. Every-3-Week Paclitaxel and Carboplatin for Ovarian Cancerr: N Engl J Med 2016;374:738-48.
57. Gonzalez-Martin A, Gladieff L, Tholander B, Stroyakovsky D, Gore M, Scambia G et al. Updated results from OCTAVIA (front-line bevacizumab, carboplatin and weekly paclitaxel therapy for ovarian cancer). European Journal of Cancer.2013; 50(4): 862 – 863
58. Bear H, Tang G, Rastogi P, Geyer Jr CE, Robidoux A, Atkins JN et al. Bevacizumab Added to Neoadjuvant Chemotherapy for Breast Cancer. N Engl J Med 2012; 366:310-320

59. Von Minckwitz G, Eidtmann H, Rezai M, Fasching P, Tesch H, Eggemann H. Neoadjuvant Chemotherapy and Bevacizumab for HER2-Negative Breast Cancer. N Engl J Med 2012; 366:299-309
60. Chéreau, Elisabeth, Lambaudie, Eric, Houvenaeghel, Gilles. Morbidity of Surgery After Neoadjuvant Chemotherapy Including Bevacizumab for Advanced Ovarian Cancer. Int J Gynecol Cancer. 2013; 23 (7): 1326-1330
61. Salani R, O'Malley DM, Copeland LJ, Cohn DE, Backes FJ, Fowler M et al. Feasibility of Interval Cytoreduction Following Neoadjuvant Chemotherapy With Carboplatin, Weekly Paclitaxel, and Bevacizumab for Advanced Ovarian Cancer—A Phase 1 Study. Int J Gynecol Cancer. 2014; 24 (4): 682-686
62. Rouzier R, Morice P, Floquet A, Selle F, Lambaudie E, Fourchotte V et al. A randomized, open-label, phase II study assessing the efficacy and the safety of bevacizumab in neoadjuvant therapy in patients with FIGO stage IIIc/IV ovarian, tubal, or peritoneal adenocarcinoma, initially unresectable. ASCO Meeting Abstracts. 2014. 32:5s. (suppl; abstr TPS5614)
63. Joly F. Personal Communication. 2014.
64. du Bois A1, Reuss A, Pujade-Lauraine E, Harter P, Ray-Coquard I, Pfisterer J. Role of surgical outcome as prognostic factor in advanced epithelial ovarian cancer: a combined exploratory analysis of 3 prospectively randomized phase 3 multicenter trials: by the Arbeitsgemeinschaft Gynaekologische Onkologie Studiengruppe Ovarialkarzinom (AGO-OVAR) and the Groupe d'Investigateurs Nationaux Pour les Etudes des Cancers de l'Ovaire (GINECO).Cancer. 2009; 115 (6): 1234-1244
65. Van Cutsem E, Rivera F, Berry S, Kretzschmar A, Michael M, DiBartolomeo M et al. Safety and efficacy of first-line bevacizumab with FOLFOX, XELOX, FOLFIRI and fluoropyrimidines in metastatic colorectal cancer: the BEAT study. Ann Oncol. 2009 Nov;20(11):1842-7
66. Kozloff M, Yood MU, Berlin J, Flynn PJ, Kabbinavar FF, Purdie DM et al. Clinical outcomes associated with bevacizumab-containing treatment of metastatic colorectal cancer: the BRiTE observational cohort study. Oncologist. 2009 Sep;14(9):862-70.
67. Constantinidou A, Cunningham D, Shurmahi F, Asghar U, Barbachano Y, Khan A et al. Perioperative chemotherapy with or without bevacizumab in patients with metastatic colorectal cancer undergoing liver resection. Clinical colorectal cancer. 2013; 12(1): 15-22
68. Okines AF, Langley RE, Thompson LC, Stenning SP, Stevenson L, Falk S et al. Bevacizumab with peri-operative epirubicin, cisplatin and capecitabine (ECX) in localised gastro-oesophageal adenocarcinoma: a safety report. Ann Oncol. 2013 Mar;24(3):702-9.
69. Prat J for the FIGO Committee on Gynecologic Oncology. Staging classification for cancer of the ovary, fallopian tube, and peritoneum. Int J Gynecol Obstet. 2014; 124 (1): 1-5

14 APPENDICES

		Page No.
Appendix 1	Trial Management Group	**203**
Appendix 2	1988 FIGO Staging (For ICON8)	**204**
Appendix 3	2013 FIGO Staging (For ICON8B)	**207**
Appendix 4	ECOG Performance Status	**208**
Appendix 5	Calculation and Measurement of GFR to Determine Carboplatin Dose	**209**
Appendix 6	GCIG Statement On Minimum Surgical Standards	**211**
Appendix 7	Common Terminology Criteria For Adverse Events v4 (abridged)	**212**
Appendix 8	Evaluation Of Residual Disease, Evaluation Of And Definition Of Progression	**219**
Appendix 9	Patient Information Sheet And Informed Consent Forms Guidance	**223**
Appendix 10	Quality Of Life - Information Sheet For Clinicians	**224**
Appendix 11	Translational Research: TRICON8 (For ICON8)	**225**
Appendix 12	Translational Research: TRICON8B (For ICON8B)	**227**
Appendix 13	Chemotherapy Response Score	**229**
Appendix 14	Multi-omics imaging analysis	**230**

APPENDIX 1 TRIAL MANAGEMENT GROUP

**UK (MRC/NCRI)**	Dr Andrew Clamp (Chief Investigator)<br><br>(Senior Lecturer and Honorary Consultant in Medical Oncology, The Christie Hospital, Manchester; Andrew.Clamp@christie.nhs.uk)<br><br>Prof. Jonathan Ledermann(Co-Chief Investigator)<br><br>(Consultant Medical Oncologist, University College London, Cancer Research UK and UCL Cancer Trials<br><br>Centre; j.ledermann@ctc.ucl.ac.uk)<br><br>Prof. Rick Kaplan (MRC CTU Programme Lead)<br><br>Dr Alia Alchawaf (Trial Physician)<br><br>Rahela Choudhury (MRC CTU Clinical Operations Manager)<br><br>Gosala Gopalakrishnan (MRC CTU Trial Manager)<br><br>Emma Kent (MRC CTU Trial Manager)
**Korea (KGOG)**	Prof. Jae Weon Kim (KGOG Chief Investigator)<br>Min-Suh Kim (KGOG Operations)
**Mexico (GICOM)**	Prof. Dolores Gallardo (GICOM Chief Investigator)<br>Adriana Chavez-Blanco (GICOM Operations)
**Australia & New Zealand (ANZGOG)**	Dr Andrew Dean/Prof. Michael Friedlander (ANZGOG Chief Investigators)<br>Kim Gilles (ANZGOG Operations)
**Ireland (ICORG)**	Dr Dearbhaile O'Donnell (ICORG Chief Investigator)<br>Beata Sapetto- Rebow (ICORG Operations)
**Collaborators**	Prof. Tim Perren<br>Prof. Iain McNeish<br>Mr Raj Naik<br>Dr Jane Hook<br>Mrs Sudha Sundar<br>Dr Chris Coyle
**Pharmacist**	Geoff Saunders
**Trial Statistician**	Adrian Cook

	Liz James
**Patient Representatives**	Dianna Fry<br>Eva Burnett<br>Sue Mannix
**Translational Research**	Dr James Brenton
**Health Economics**	Prof. Mark Sculpher
**QoL Lead**	Dr Sarah Blagden

APPENDIX 2 1988 FIGO STAGING

Appendix 2.1 FIGO Staging: Carcinoma of the Ovary[47]

Table 27

FIGO		TNM
	Primary Tumour cannot be assessed	TX
0	No evidence of primary tumour	T0
I	Tumour confined to ovaries	T1
IA	Tumour limited to one ovary, capsule intact<br>No tumour on ovarian surface<br>No malignant cells in the ascites or peritoneal washings	T1a
IB	Tumour limited to both ovaries, capsules intact<br>No tumour on ovarian surface<br>No malignant cells in the ascites or peritoneal washings	T1b
IC	Tumour limited to one or both ovaries, with any of the following:<br>Capsule ruptured<br>Tumour on ovarian surface<br>Positive malignant cells in the ascites or positive peritoneal washings	T1c
II	Tumour involves one or both ovaries with pelvic extension	T2
IIA	Extension and/ or implants in uterus and/or tubes<br>No malignant cells in the ascites or peritoneal washings	T2a
IIB	Extension to other pelvic organ<br>No malignant cells in the ascites or peritoneal washings	T2b
IIC	IIA/B with positive malignant cells in the ascites or positive peritoneal washings	T2c
III	Tumour involves one or both ovaries with microscopically confirmed peritoneal metastasis outside the pelvis and/or regional lymph nodes metastasis	T3 and/or N1
IIIA	Microscopic peritoneal metastasis beyond the pelvis	T3a

IIIB	Macroscopic peritoneal metastasis beyond the pelvis 2 cm or less in greatest dimension	T3b
IIIC	Peritoneal metastasis beyond pelvis more than 2cm in greatest dimension and/or regional lymph nodes metastasis	T3c and/or N1
IV	Distant metastasis beyond the peritoneal cavity	M1

Note: Liver capsule metastasis is T3/ Stage III, liver parenchymal metastasis M1/Stage IV. Pleural effusion must have positive cytology.

APPENDIX 2.2 FIGO STAGING: CARCINOMA OF THE FALLOPIAN TUBE[47]

Table 28

FIGO		TNM
	Primary Tumour cannot be assessed	TX
0	No evidence of primary tumour	T0
	Carcinoma in situ (pre-invasive carcinoma)	Tis
I	Tumour confined to fallopian tubes	T1
IA	Tumour limited to one tube, without penetrating the serosal surface; no ascites	T1a
IB	Tumour limited to both tubes, without penetrating the serosal surface; no ascites	T1b
IC	Tumour limited to one or both tubes, with extension onto/through the tubal serosa; or with positive malignant cells in the ascites or positive peritoneal washings	T1c
II	Tumour involves one or both fallopian tubes with pelvic extension	T2
IIA	Extension and/ or metastasis to uterus and/or ovaries	T2a
IIB	Extension to other pelvic organ	T2b
IIC	IIB/C with positive malignant cells in the ascites or positive peritoneal washings	T2c
III	Tumour involves one or both fallopian tubes with peritoneal<br><br>Implants outside the pelvis and /or positive regional lymph	T3 and/or N1

	nodes	
IIIA	Microscopic peritoneal metastasis outside the pelvis	T3a
IIIB	Macroscopic peritoneal metastasis outside the pelvis 2 cm or less in greatest dimension	T3b
IIIC	Peritoneal metastasis more than 2cm in greatest dimension and/or positive regional lymph nodes	T3c and/or N1
IV	Distant metastasis beyond the peritoneal cavity	M1

Note: Liver capsule metastasis is T3/ Stage III, liver parenchymal metastasis M1/Stage IV. Pleural effusion must have positive cytology.

Appendix 2.3 Primary Peritoneal Carcinoma Staging

There is no recognised formal staging system for primary peritoneal carcinoma and the FIGO staging for epithelial ovarian carcinoma has been adopted. Surface involvement of the ovaries in the absence of more widespread peritoneal disease would be classified as FIGO stage IC. This patient would only be eligible if the tumour was Grade 3. Patients with more extensive peritoneal disease would be classified as having stage IIC disease if this is confined to the pelvis, and stage III or IV disease for disease beyond the pelvis (defined according to the FIGO ovarian system- **Appendix 2.1**).

Appendix 2.4 Ovarian Carcinosarcoma Staging

Staging of ovarian carcinosarcoma follows the FIGO staging for epithelial ovarian carcinoma (**Appendix 2.1**).

APPENDIX 3 FIGO 2013 STAGING

To be used for assessment of stage in participants entering ICON8B.

Table 29

FIGO		TNM
	Primary Tumour cannot be assessed	TX
0	No evidence of primary tumour	T0
I	Tumour confined to ovaries or fallopian tube(s)	T1
IA	Tumour limited to one ovary (capsule intact) or fallopian tube;<br>No tumour on ovarian or fallopian tube surface;<br>No malignant cells in the ascites or peritoneal washings	T1a
IB	Tumour limited to both ovaries (capsules intact) or fallopian tubes with;<br>No tumour on ovarian or fallopian tube surface<br>No malignant cells in the ascites or peritoneal washings	T1b
IC	Tumour limited to one or both ovaries or fallopian tubes with any of the following:<br>Surgical spill (IC1)<br>Capsule ruptured before surgery or tumour on ovarian or fallopian tube surface (IC2)<br>Malignant cells in the ascites or peritoneal washings (IC3)	T1c
II	Tumour involves one or both ovaries or fallopian tubes with pelvic extension (below pelvic brim) or primary peritoneal cancer	T2
IIA	Extension and/ or implants on uterus and/or fallopian tubes and/or ovaries	T2a
IIB	Extension to other pelvic intraperitoneal tissue	T2b
III	Tumour involves one or both ovaries or fallopian tubes, or primary peritoneal cancer, with cytologically or histologically confirmed spread spread to the peritoneum outside the pelvis and/or metastasis to the retroperitoneal lymph nodes	T3 and/or N1
IIIA1	Positive retroperitoneal lymph nodes only (cytologically or histologically proven)	T3a
IIIA1(i)	Metastasis up to 10mm in greatest diameter	T3a
IIIA1(ii)	Metastasis more than 10mm in greatest diameter	T3a
IIIA2	Microscopic extrapelvic (above the pelvic brim) peitoneal involvement with or without positive retroperitoneal lymph nodes	T3a

IIIB	Macroscopic peritoneal metastasis beyond the pelvis up to 2 cm in greatest dimension, with or without metastasis to the retroperitoneal lymph nodes	T3b
IIIC	Macroscopic peritoneal metastasis beyond the pelvis more than 2 cm in greatest dimension, with or without metastasis to the retro- peritoneal lymph nodes (includes extension of tumour to capsule of liver and spleen without parenchymal involvement of either organ)	T3c and/or N1
IV	Distant metastasis excluding peritoneal metastases	M1
IVA	Pleural effusion with positive cytology	M1
IVB	Parenchymal metastases and metastases to extra-abdominal organs (including inguinal lymph nodes and lymph nodes outside of the abdominal cavity)	M1

APPENDIX 4 ECOG PERFORMANCE STATUS[48]

Table 30

Description	Scale
Normal activity	0
Symptomatic but ambulatory self-care	1
Ambulatory more than 50% of the time	2
Ambulatory 50% or less of time, nursing care needed	3
Bedridden, may need hospitalisation	4

APPENDIX 5 CALCULATION AND MEASUREMENT OF GFR TO DETERMINE CARBOPLATIN DOSE

It is highly recommended that the GFR is measured using an isotopic method except in the presence of significant third space fluid collections (ascites/pleural effusion/gross peripheral oedema) in accordance with internationally recognised guidelines for the use of renally excreted drugs with narrow therapeutic indices (NKDEP guidelines for prescribers 2010). If the calculated serum creatinine clearance is <60ml/minute, then a formal measurement of the GFR is mandatory, using either a 24 hour urine collection or an isotopic clearance. If the isotopic clearance is measured then the value uncorrected for body surface area (BSA) should be used in dose calculations.

For the purposes of this protocol, the GFR can be considered equivalent to the creatinine clearance (CrCl). If a calculated CrCl method is used to estimate the GFR, it is recommended that this is performed as per local practice by the use of the Wright, Cockcroft-Gault or Jelliffe formulae. Of note, the modified Wright formula, when utilized with the appropriate correction for creatinine assay method, has consistently been shown to be the most accurate formula for estimating renal function in cancer patients[41, 42].

APPENDIX 5.1 CALCULATION OF GFR USING THE WRIGHT FORMULA[49]

There are a number of different Wright formulae, depending on whether or not the creatinine kinase is available and used in the calculation, and also depending on how the serum creatinine is measured. The formula immediately below does not require a creatinine kinase measurement. This formula is also only valid if the laboratory measuring the serum creatinine uses the Jaffe method to do this. Centres will need to check with their local pathology laboratory how the serum creatinine is measured.

$$GFR = \frac{\{[6580 - (38.8 \times age)] \times BSA \times 0.832\}}{SCr}$$

If the creatinine is measured using enzymic methods then the following Wright formula should be used:

$$GFR = \frac{\{[6230-(32.8 \times age)] \times BSA \times 0.77\}}{SCr}$$

Where

GFR= Glomerular Filtration Rate (ml/min)
BSA= DuBois Body Surface Area (m2)
SCr = Serum Creatinine (µmol/l)
Wt = Weight (kg)
Age = Age in years (20 to 80)

To convert serum creatinine in mg/dl to µmol/l use the following formula:

Cr (µmol/l) = Cr (mg/dl) x 88.4

APPENDIX 5.2 CALCULATION OF GFR USING THE JELLIFFE FORMULA[50]

$$GFR = \frac{0.9 \times [98-\{0.8(age-20)\}] \times [BSA/1.73]}{SCr \times 0.0113}$$

APPENDIX 5.3 CALCULATION OF GFR USING THE COCKCROFT-GAULT FORMULA[51]

$$GFR = \frac{1.05 \times (140-age) \times Wt}{SCr}$$

NB. The local laboratory creatinine assay used and the method used to measure GFR or calculate creatinine clearance will be collected at a site level and should be recorded in the Investigator Site File.

APPENDIX 6 GCIG CONSENSUS STATEMENT ON MINIMUM SURGICAL STANDARDS[5]

1. Tissue should be obtained for pathological diagnosis to confirm the presence of epithelial ovarian, primary peritoneal or fallopian tube carcinoma

2. Staging should be performed according to FIGO (1988) guidelines. This includes at least lymph node sampling and peritoneal staging in early stage invasive disease (FIGO I – IIA)

3. Up-front maximal surgical effort at cytoreduction with the goal of no residual disease should be undertaken

4. Patients with ovarian cancer should have their surgery performed by an appropriately trained surgeon with experience in the management of ovarian cancer.

APPENDIX 7 COMMON TERMINOLOGY CRITERIA FOR ADVERSE EVENTS

Toxicity is defined in accordance with the NCI-CTCAE v4.0, which is available at http://evs.nci.nih.gov/ftp1/CTCAE/CTCAE_4.03_2010-06-14_QuickReference_5x7.pdf

Please note that only selected categories are listed below, please refer to the full list using the link above for other toxicities.

Table 31

Adverse Event	1	2	3	4	5
			Blood and Lymphatic Disorders		
**Anaemia**	Hemoglobin (Hgb) <LLN - 10.0 g/dL; <LLN - 6.2 mmol/L; <LLN - 100 g/L	Hgb <10.0 - 8.0 g/dL; <6.2 - 4.9 mmol/L; <100 - 80g/L	Hgb <8.0 g/dL; <4.9 mmol/L; <80 g/L; transfusion indicated	Life-threatening consequences; urgent intervention indicated	Death
**Febrile neutropenia**	--	--	ANC <1000/mm^3 with a single temperature of >38.3°C (101°F) or a sustained temperature of ≥38°C (100.4°F) for more than one hour	Life-threatening consequences; urgent intervention indicated	Death
			Cardiac Disorders		
**Acute coronary syndrome**	--	Symptomatic, progressive angina; cardiac enzymes normal; hemodynamically stable	Symptomatic, unstable angina and/or acute myocardial infarction, cardiac enzymes abnormal, hemodynamically stable	Symptomatic, unstable angina and/or acute myocardial infarction, cardiac enzymes abnormal, hemodynamically unstable	Death
**Myocardial infarction**	--	Asymptomatic and cardiac enzymes minimally abnormal and no evidence of ischemic ECG changes	Severe symptoms; cardiac enzymes abnormal; hemodynamically stable; ECG changes consistent with infarction	Life-threatening consequences; hemodynamically unstable	Death
			Gastrintestinal Disorders		

ADVERSE EVENT	1	2	3	4	5
**Constipation**	Occasional or intermittent symptoms; occasional use of stool softeners, laxatives, dietary modification, or enema	Persistent symptoms with regular use of laxatives or enemas; limiting instrumental ADL	Obstipation with manual evacuation indicated; limiting self care ADL	Life-threatening consequences; urgent intervention indicated	Death
**Diarrhea**	Increase of <4 stools per day over baseline; mild increase in ostomy output compared to baseline	Increase of 4 - 6 stools per day over baseline; moderate increase in ostomy output compared to baseline	Increase of ≥7 stools per day over baseline; incontinence; hospitalization indicated; severe increase in ostomy output compared to baseline; limiting self care ADL	Life- threatening consequences; urgent intervention indicated	Death
**Gastrintestinal Fistula**	Asymptomatic; clinical or diagnostic observations only; intervention not indicated	Symptomatic; altered GI function	Severely altered GI function; tube feeding, TPN or hospitalization indicated	Life-threatening consequences; urgent operative intervention indicated	Death
**Mucositis oral**	Asymptomatic or mild symptoms; intervention not indicated	Moderate pain; not interfering with oral intake; modified diet indicated	Severe pain; interfering with oral intake	Life-threatening consequences; urgent intervention indicated	Death
**Nausea**	Loss of appetite without alteration in eating habits	Oral intake decreased without significant weight loss, dehydration or malnutrition	Inadequate oral caloric or fluid intake; tube feeding, TPN, or hospitalization indicated	--	--
**Vomiting**	1 – 2 episodes (separated by 5 minutes) in 24 hrs	3 - 5 episodes (separated by 5 minutes) in 24 hrs	≥6 episodes (separated by 5 minutes) in 24 hrs; tube feeding, TPN or hospitalization indicated	Life-threatening consequences; urgent intervention indicated	Death
GENERAL DISORDERS AND ADMINISTRATIO SITE CONDITIONS					
**Fatigue**	Fatigue relieved by rest	Fatigue not relieved by rest; limiting instrumental ADL	Fatigue not relieved by rest, limiting self care ADL	--	--

ADVERSE EVENT	1	2	3	4	5
**Infusion site extravasation**	--	Erythema with associated symptoms (eg. edema, pain, induration, phlebitis)	Ulceration or necrosis; severe tissue damage; operative intervention indicated	Life-threatening consequences; urgent intervention indicated	Death
**Injection site reaction**	Tenderness with or without associated symptoms (eg. warmth, erythema, itching)	Pain; lipodystrophy; edema; phlebitis	Ulceration or necrosis; severe tissue damage; operative intervention indicated	Life-threatening consequences; urgent intervention indicated	Death
IMMUNE SYSTEM DISORDERS					
**Allergic reaction**	Transient flushing or rash, drug fever <38 ºC (<100.4ºF); intervention not indicated	Intervention or infusion interruption indicated; responds promptly to symptomatic treatment (eg. antihistamines, NSAIDS, narcotics); prophylactic medications indicated for ≤24 hrs	Prolonged (eg., not rapidly responsive to symptomatic medication and/or brief interruption of infusion); recurrence of symptoms following initial improvement; hospitalization indicated for clinical sequelae (eg. renal impairment, pulmonary infiltrates)	Life-threatening consequences; urgent intervention indicated	Death
**Anaphylaxis**	--	--	Symptomatic bronchospasm, with or without urticaria; parenteral intervention indicated; allergy-related edema/ angioedema; hypotension	Life-threatening consequences; urgent intervention indicated	Death
INJURY, POISONING AND PROCEDURAL COMPLICATIONS					
**Wound Complication**	Incisional separation of <=25% of wound, no deeper than superficial fascia	Incisional separation >25% of wound; local care indicated	Hernia without evidence of strangulation; fascial disruption/dehiscence; primary wound closure or revision by operative intervention indicated	Hernia with evidence of strangulation; major reconstruction flap, grafting, resection, or amputation indicated	Death

Adverse Event	1	2	3	4	5
**Wound Dehiscence**	Incisional separation of<br><br><=25% of wound, no deeper than superficial fascia	Incisional separation >25% of wound with local care;<br><br>asymptomatic hernia or symptomatic hernia without evidence of strangulation	Fascial disruption or<br><br>dehiscence without evisceration; primary wound closure or revision by operative intervention indicated	Life-threatening<br><br>consequences; symptomatic hernia with evidence of strangulation; fascial disruption with evisceration; major reconstruction flap, grafting, resection, or amputation indicated	Death
			Investigations		
**ALT increased**	>ULN - 3.0 x ULN	>3.0 - 5.0 x ULN	>5.0 - 20.0 x ULN	>20.0 x ULN	--
**AST increased**	>ULN - 3.0 x ULN	>3.0 - 5.0 x ULN	>5.0 - 20.0 x ULN	>20.0 x ULN	--
**Blood bilirubin increased**	>ULN - 1.5 x ULN	>1.5 - 3.0 x ULN	>3.0 - 10.0 x ULN	>10.0 x ULN	--
**Creatinine increased**	>1 - 1.5 x baseline; >ULN - 1.5 x ULN	>1.5 - 3.0 x baseline; >1.5 - 3.0 x ULN	>3.0 baseline; >3.0 - 6.0 x ULN	>6.0 x ULN	--
**Neutrophil count decreased**	<LLN - 1500/mm3; <LLN - 1.5 x 10^9/L	<1500 - 1000/mm^3; <1.5 - 1.0 x 10^9/L	<1000 - 500/mm^3; <1.0 - 0.5 x 10^9/L	<500/mm^3; <0.5 x 10^9/L	--
**Platelet count decreased**	<LLN - 75,000/mm^3; <LLN - 75.0 x10^9/L	<75,000 -50,000/mm^3; <75.0 - 50.0 x10^9/L	<50,000 -25,000/mm^3; <50.0 - 25.0 x10^9/L	<25,000/mm^3; <25.0 x 10^9/L	--
**White blood cell decreased**	<LLN - 3000/mm^3; <LLN - 3.0 x 10^9/L	<3000 - 2000/mm^3; <3.0 - 2.0 x 10^9/L	<2000 - 1000/mm^3; <2.0 - 1.0 x 10^9/L	<1000/mm^3; <1.0 x 10^9/L	--
			Metabolism and Nutrition Disorders		
**Anorexia**	Loss of appetite without alteration in eating habits	Oral intake altered without significant weight loss or malnutrition; oral nutritional supplements indicated	Associated with significant weight loss or malnutrition (eg. inadequate oral caloric and/or fluid intake); tube feeding or TPN indicated	Life-threatening consequences; urgent intervention indicated	Death
			Muscoskeletal and connective tissue disorders		

Adverse Event	1	2	3	4	5
**Arthralgia**	Mild pain	Moderate pain; limiting instrumental ADL	Severe pain; limiting self care ADL	--	--
**Myalgia**	Mild pain	Moderate pain; limiting instrumental ADL	Severe pain; limiting self care ADL	--	--
**Osteonecrosis of jaw**	Asymptomatic; clinical or diagnostic observations only; intervention not indicated	Symptomatic; medical intervention indicated (e.g., topical agents); limiting instrumental ADL	Severe symptoms; limiting self care ADL; elective operative intervention indicated	Life-threatening consequences; urgent intervention indicated	Death
		Nervous System Disorders			
**Peripheral motor neuropathy**	Asymptomatic; clinical or diagnostic observations only; intervention not indicated	Moderate symptoms; limiting instrumental ADL	Severe symptoms; limiting self care ADL; assistive device indicated	Life-threatening consequences; urgent intervention indicated	Death
**Peripheral sensory neuropathy**	Asymptomatic; loss of deep tendon reflexes or paresthesia	Moderate symptoms; limiting instrumental ADL	Severe symptoms; limiting self care ADL	Life-threatening consequences; urgent intervention indicated	Death
**Reversible posterior leukoencephalopathy syndrome**	Asymptomatic; clinical or diagnostic observations only; intervention not indicated	Moderate symptoms; abnormal imaging studies; limiting instrumental ADL	Severe symptoms; very abnormal imaging studies; limiting self care ADL	Life-threatening consequences; urgent intervention indicated	Death
		Renal and Urinary Disorders			
**Proteinuria**	1+ proteinuria; urinary protein <1.0 g/24 hrs	Adults: 2+ proteinuria; urinary protein 1.0 - 3.4 g/24 hrs;	Adults: urinary protein >=3.5g/24 hrs;	--	--
		Skin and Subcutaneous Disorders			
**Alopecia**	Hair loss of <50% of normal for that individual that is not obvious from a distance but	Hair loss of ≥50% normal for that individual that is readily apparent to others; a wig or	--	--	--

ADVERSE EVENT	1	2	3	4	5
	only on close inspection; a different hair style may be required to cover the hair loss but it does not require a wig or hair piece to camouflage	hair piece is necessary if the patient desires to completely camouflage the hair loss; associated with psychosocial impact			
VASCULAR DISORDERS					
**Thromboembolic event**	Venous thrombosis (eg. superficial thrombosis)	Venous thrombosis (eg. uncomplicated deep vein thrombosis), medical intervention indicated	Thrombosis (eg. uncomplicated pulmonary embolism [venous], non-embolic cardiac mural [arterial] thrombus), medical intervention indicated	Life-threatening (eg. pulmonary embolism, cerebrovascular event, arterial insufficiency); hemodynamic or neurologic instability; urgent intervention indicated	Death
**Hypertension**	Prehypertension (systolic BP 120 - 139 mm Hg or diastolic BP 80 - 89 mm Hg)	Stage 1 hypertension (systolic BP 140 - 159 mm Hg or diastolic BP 90 - 99 mm	Stage 2 hypertension (systolic BP >=160 mm Hg or diastolic BP >=100 mm Hg);	Life-threatening	Death

ADVERSE EVENT	1	2	3	4	5
		Hg); medical intervention indicated;<br><br>recurrent or persistent (>=24 hrs); symptomatic increase by >20 mm Hg (diastolic) or to >140/90 mm Hg if previously WNL; monotherapy indicated.	medical intervention indicated; more than one drug or more intensive therapy than previously used indicated<br><br>Pediatric: Same as adult	consequences (e.g., malignant hypertension, transient or permanent neurologic deficit, hypertensive crisis); urgent intervention indicated<br>Pediatric: Same as adult	

APPENDIX 8
EVALUATION OF RESIDUAL DISEASE, EVALUATION OF AND DEFINITION OF PROGRESSION

APPENDIX 8.1 ASSESSMENT OF RESIDUAL DISEASE

An assessment of residual disease remaining after surgery is required[52]. This should be based on details in the surgical report. Residual disease will be classified as:

- Debulked to no visible residual disease
- Debulked to ≤1.0cm visible residual disease
- Debulked to >1.0cm visible residual disease.

APPENDIX 8.2 ASSESSMENT OF MEASURABLE VS. NON-MEASURABLE DISEASE

APPENDIX 8.2.1 METHOD OF TUMOUR ASSESSMENT

CT scan of the pelvis and abdomen is the preferred method of evaluation, but MRI is allowed. The same method of assessment should be used at baseline and for all scans during follow-up. If there are known lesions outside the pelvis and abdomen, they should be included in all tumour assessments.

APPENDIX 8.2.2 ASSESSMENT OF BASELINE DISEASE

An assessment of baseline disease is required.

For patients who have undergone IPS prior to entry into the trial this should be performed at least 4 weeks post-operatively and less than 2 weeks after starting chemotherapy. If chemotherapy is started earlier than 2 weeks after surgery, the CT scan should be performed 4 weeks +/- 7 days post-operatively.

For patients who have DPS (or IDS), the baseline assessment will be the post-operative CT scan which should be performed 4 weeks +/- 7 days after surgery.

For patients who were planned to have DPS but in whom surgery was not appropriate, the baseline assessment will be taken to be the initial CT scan which was performed within 4 weeks of randomisation.

Patients who undergo debulking surgery after 6 cycles of chemotherapy will require an additional radiological assessment of disease 4 weeks (+/- 7 days) after their delayed surgery to act as a new baseline assessment for progression-free survival. This is anticipated to be a small number of patients, for further information in ICON8 see **section A3.3.2: Delayed Primary Surgery**, in ICON8NB see **section B3.3.2: Delayed Primary Surgery.**

A patient is not required to have measurable disease as per RECIST for entry, and will be classified as having measurable or non-measurable disease at baseline and at each imaging assessment thereafter.

APPENDIX 8.2.3 FURTHER TUMOUR ASSESSMENTS

APPENDIX 8.2.3A ICON8 FURTHER TUMOUR ASSESSMENTS

In the ICON8 cohort, all patients should undergo tumour re-assessment at the end of treatment, this should be performed 6 weeks (+/- 2 weeks) after day 1 of the last cycle of first line treatment.

For the Stage 2 analysis, tumour re-assessment is required at 9 months post-randomisation. This will mean that approximately the first 186 patients will require a single protocol-mandated CT/MRI scan during the follow-up period.

Further routine re-assessment imaging is not otherwise required. At subsequent follow-up visits tumour assessments should only be performed if clinical symptoms are suggestive of recurrence or there is CA125 progression by GCIG criteria. Radiological tumour assessment should occur within 3 weeks of the date of suspected clinical or CA125 progression. If there is CA125 progression but no radiological documentation of disease progression then imaging should be repeated 3-monthly until protocol defined progression occurs.

APPENDIX 8.2.3B ICON8B FURTHER TUMOUR ASSESSMENTS

In the ICON8B cohort, all patients should undergo tumour re-assessment at the end of of the chemotherapy phase, this should be performed 3 weeks (+/- 2 weeks) after day 1 of the last cycle of first line chemotherapy.

At subsequent follow up visits until week 66, tumour assessments should only be performed if clinical symptoms are suggestive of recurrence or there is CA125 progression by GCIG criteria. Radiological tumour assessment should occur within 3 weeks of the date of suspected clinical or CA125 progression. If there is CA125 progression but no radiological documentation of disease progression then imaging should be repeated 3-monthly until protocol defined progression occurs.

All patients should have a CT scan at week 66 (+/- 2 weeks) post-randomisation.

Followimg the week 66 visit, further routine re-assessment imaging is not otherwise required. At subsequent follow-up visits tumour assessments should only be performed if clinical symptoms are suggestive of recurrence or there is CA125 progression by GCIG criteria. Radiological tumour assessment should occur within 3 weeks of the date of suspected clinical or CA125 progression. If there is CA125 progression but no radiological documentation of disease progression then imaging should be repeated 3-monthly until protocol defined progression occurs.

APPENDIX 8.2.4 DEFINITION OF MEASURABLE DISEASE LESIONS

Measurable disease is defined as at least one lesion that can be accurately measured in at least one dimension (longest diameter (LD) to be recorded).

- Each lesion must be ≥10mm when assessed by CT and MRI (if the slice thickness is ≤5mm), and clinical examination; or ≥20mm when measured by plain X-ray. (For CT and MRI scans where the slice thickness is >5mm, measurability is defined as a lesion with LD ≥ 2x the slice thickness)
- For malignant lymph nodes, the short axis diameter should be recorded and must by ≥15mm to be considered pathological and measurable[53].

APPENDIX 8.2.5 DEFINITION OF NON-MEASURABLE LESIONS

These are all other lesions, including small lesions (longest diameter < the criteria defined above), eg. bone lesions; leptomeningeal disease; ascites; pleural/pericardial effusions; inflammatory breast disease; lymphangitis; cystic lesions; and also abdominal masses that are identified by physical examination but not measurable by imaging techniques.

APPENDIX 8.2.6 BASELINE DOCUMENTATION OF "TARGET" AND "NON-TARGET" LESIONS

1. All measurable lesions, up to a maximum of five in total, representative of all involved organs should be identified as target lesions and recorded and measured at baseline

2. Target lesions should be selected on the basis of their size (lesions with the longest diameter) and their suitability for accurate repeated measurements (either by imaging techniques or clinically)

3. All other lesions (or sites of disease) should be identified as non-target lesions and should also be recorded at baseline. Measurements of these lesions are not required, but the presence or absence of each should be noted throughout follow-up .

APPENDIX 8.3 DEFINITION OF PROGRESSION

APPENDIX 8.3.1 MEASURABLE DISEASE

For patients with measurable disease, progression is defined as ANY of the following:

1. At least a 20% increase in the sum of LD target lesions taking as reference the smallest sum LD recorded since the baseline assessment

2. In the case where the ONLY target lesion is a solitary pelvic mass measured by physical exam which is not radiographically measurable, a 50% increase in the LD is required taking as reference the smallest LD recorded since trial entry

3. The appearance of one or more new lesions

4. Unequivocal progression of existing non-target lesions, other than pleural effusions without cytological proof of neoplastic origin, in the opinion of the treating physician (in this circumstance an explanation must be provided)

5. Global deterioration in health status attributable to the disease requiring a change in therapy without objective evidence of progression

6. Death due to disease without prior objective documentation of progression.

APPENDIX 8.3.3 NON-MEASURABLE DISEASE

For patients with non-measurable disease, progression is defined as increasing clinical, radiological or histological evidence of disease since baseline assessment.

All patients, including those with measurable disease whose progression is classified as 'global deterioration without objective evidence of progression and those with non-measurable disease at randomisation, are expected to have a CT or MRI scan at the time of progression to formally document radiological disease status.

APPENDIX 8.3.4 ROLE OF SERUM CA125 IN ASSESSMENT OF PROGRESSION

In the ICON8 Trials Programme progression is not defined in terms of CA125, and it is not expected that a decision to start treatment for progression will be taken on the basis of a rising CA125 alone. However, regular serum CA125 measurements should be taken while on treatment and during follow-up. If there is isolated CA125 progression, tumour assessments by repeat imaging should be performed 3 monthly until documented radiological, or clinical, progression as described above.

GCIG criteria for CA125 progression[54]:

1. Patients with elevated CA125 pre-treatment and normalisation of CA125 must show evidence of CA125 greater than or equal to two times the upper normal limit on two occasions at least one week apart or

2. Patients with elevated CA125 pre-treatment, which never normalises must show evidence of CA125 greater than or equal to two times the nadir value on two occasions at least one week apart or

3. Patients with CA125 in the normal range pre-treatment must show evidence of CA125 greater than or equal to two times the upper normal limit on two occasions at least one week apart.

APPENDIX 9 PATIENT INFORMATION SHEETS AND INFORMED CONSENT FORMS GUIDANCE

The guidance that follows encompasses the ICH GCP guidelines.

Potential recruits to ICON8 and ICON8B must be given sufficient information to allow them to decide whether or not they want to take part.

Generic versions of the ICON8 and ICON8B Patient Information Sheet (PIS), Informed Consent Form (ICF) and a Supplementary ICF for Biological Samples have been developed and provided to each GCIG group. Wording that will require tailoring for each GCIG group is given in square brackets [like this]. It should be written in simple, non-technical terms using short words, sentences and paragraphs, which should be easily understood by a lay person with the average reading ability of a 12-year old.

A generic General Practitioner Information Letter (GPIL) for the patient's GP/Family Doctor has also been prepared. This should be provided to all patients' GPs unless they have not given consent for their GP to be informed of their participation in ICON8 or ICON8B.

Each participating GCIG group must produce an English language version of the PIS, ICFs and GPIL which should include any country specific-changes to the generic documents. The MRC must be given the opportunity to review and comment on these country-specific versions before the clinical trial application is submitted in that country.

In countries where English is not the local language, the participating GCIG group will produce a local language translation of the country-specific English language version. This local language version must be provided to the MRC, accompanied by a signed statement by a representative of the GCIG group confirming that the local language version is correct and that it provides patients with the same information as that included in the country-specific English language version.

All PISs, ICFs and GPILs submitted to a national or main research ethics committee should be headed simply 'Hospital/Institution headed paper'.

Local PISs, ICFs and GPILs should be printed on headed paper of the hospital/institution where the research is being carried out, with local contact names and telephone numbers, before being submitted to the local research ethics committee. **Paper without a heading is not acceptable.**

APPENDIX 10 QUALITY OF LIFE - INFORMATION SHEET FOR CLINICIANS

Background

A QoL analysis is being performed to assess the potential impact of dose-fractionated chemotherapy on functioning and well-being in patients undergoing 1st line treatment for ovarian cancer. It will enable the exploration of hypotheses about the functional and symptomatic consequences of weekly chemotherapy using validated patient self-reporting measures.

In addition, information will be obtained about the impact of timing of surgery on QoL in combination with different chemotherapy schedules.

QoL Questionnaire Information

The EORTC QLQ OV-28 scale (which includes the EORTC QLQ C-30) has been selected to measure QoL in the ICON8 Trials Programme. The measures of abdominal symptoms, physical and social functioning scales it includes have better face validity to examine hypotheses relevant to the ICON8 Trials Programme than other instruments, notably the FACT-O. The questionnaire will assess patients' QoL within the last seven days of each time point.

The OV-28 instrument has 58 items. It is clear and easy to understand, acceptable to patients and usually completed in less than 15 minutes without assistance. The questionnaire has currently been translated into 14 languages: English, Croatian, Danish, Dutch, French, German, Italian, Portuguese, Spanish, Swedish, Taiwan-Chinese, Finnish, Norwegian and Polish.

The generic EQ-5D instrument has also been included in the QoL questionnaires. This is a standardised measure of health status that can be used to express HRQL in terms of 'utilities'. These can then be used to estimate patients' quality-adjusted survival duration (usually expressed in terms of quality adjusted life-years (QALYs)), which provides meaningful information in both QoL and Health Economics analyses. It records patients' self-assessed health status on the day of completion.

EQ-5D has 5 questions and a visual analogue scale (EQ-VAS) to record the patients' overall assessment of their QoL. It is designed for self-completion by the patients, is clear and easy to understand, and should take only a few minutes to complete. It has been translated into 102 languages.

Each QoL form should be completed before medical assessment, chemotherapy is given or blood tests are performed. It should be completed by the patient without input from health professionals. The first QoL form needs to be completed by the patient at her screening visit. The QoL form should be completed as per the ICON8 and ICON8B table of assessments schedules (**Section A6.2** and **Section B6**).

APPENDIX 11 TRICON8

TRICON8 is the translational research sub-study of the ICON8 trial. Its aim is to establish a large, comprehensive biobank comprising tumour tissue, blood and serial plasma samples with associated clinical data which will be an invaluable resource for high-quality translational research in ovarian cancer.

Specimens collected as part of TRICON8 will be held at the University of Cambridge. Access to tissue samples for future translational research projects will be via application to the ICON8 Trial Management Group and require approval of the Trial Steering Committee. A Translational Research sub-group will be established to assess applications (and will include TRICON8 investigators, members of the ICON8 TMG, MRC CTU senior scientific staff and independent experts).

APPENDIX 11.1 LEVELS OF PARTICIPATION IN TRANSLATIONAL RESEARCH

TRICON8 will be conducted at 3 levels as it is recognised that a variety of clinical centres with access to different levels of research support will enter patients into the trial. All centres are encouraged to participate if local R&D and logistical support allows. The majority of centres are expected to participate at levels 2 and 3. **Further detailed information on all levels of participation is given in the TRICON8 Manual.**

APPENDIX 11.1.1 LEVEL 1 (TUMOUR SPECIMENS)

All clinical centres are expected to participate in the translational research programme at this level. It is appropriate for centres with no additional facilities/support for sample collection/preparation. Formalin-fixed paraffin-embedded tissue taken at the time of IPS will be collected; and for DPS patients paired samples from the original biopsy specimen and at DPS will be collected.

APPENDIX 11.1.2 LEVEL 2 (TUMOUR SPECIMENS AND WHOLE BLOOD SAMPLE)

All clinical centres are encouraged to participate in the translational research programme at this level. It is appropriate for centres with no additional facilities/support for sample collection/preparation. Tissue specimens and peripheral blood samples for DNA will be collected.

APPENDIX 11.1.3 LEVEL 3 (TUMOUR SPECIMENS, WHOLE BLOOD SAMPLE AND SERIAL PLASMA SAMPLES)

This level is appropriate for clinical centres with access to a centrifuge and -70/80°C freezer facilities, and the expertise to process blood samples at site to form plasma & buffy coat layer aliquots. In addition to level 2 sampling, four blood samples (pre-cycle 1, pre-cycle 2, pre-cycle 6 and at protocol defined disease progression, or at 5 years from the start of treatment if the disease has not recurred) are required for potential immunoassay, proteomic and genomic analysis.

Appendix 11.2 Patient Consent for Translational Research

In order to collect the samples for TR, patients will be required to sign a specific consent form. Although most patients are expected to consent to participate, the wishes of patients who do not want to be involved in the additional TR sample collection will be respected and they will be able to enter the clinical trial protocol only.

As the patient's participation in the sample collection is entirely voluntary, they may choose to withdraw consent for collection of further samples or use of samples already collected at any time. Although the patient is not required to give a reason for withdrawing consent, a reasonable effort should be made to establish this reason while fully respecting the patient's rights. If consent for use of already collected samples is withdrawn, the MRC CTU should be informed in writing.

If consent is withdrawn, specimens held at the trial biobank will be returned to the referring centre. However, specimens that have already undergone processing, for example in the formation of tissue microarrays, will be retained.

Appendix 11.3 Sample Collection, Storage and Processing

Detailed description of the samples required and instructions for the processing, labelling, handling, storage and shipment of specimens for translational research are provided in the TRICON8 manual.

A brief overview of specimen requirements is given here.

Appendix 11.3.1 Tumour specimens

Formalin-fixed paraffin-embedded surgical/biopsy specimens of the primary tumour, metastatic sites and an area of normal tissue will be collected for formation of tissue microarrays and cores taken for nucleic acid extraction. All tumour blocks will be returned to the referring centre once required samples have been taken.

Appendix 11.3.2 Peripheral blood for DNA analysis (pharmacogenomics and single nucleotide polymorphisms)

Two peripheral blood samples will be collected at baseline. Samples should be sent directly to the biobank after collection. DNA aliquots will be prepared centrally from these samples when required.

Appendix 11.3.3 Plasma sampling for additional translational research studies

In level 3 plasma and buffy coat samples will be taken prior to administration of cycle 1, before cycle 2, before cycle 6 and at protocol defined disease progression or at 5 years from the start of treatment if there has been no disease progression by this point. Samples will be stored locally at -80°C until requested for transfer to the biobank.

APPENDIX 12 TRICON8B

TRICON8B is the translational research sub-study of the ICON8B trial. Its aim is to establish a large, comprehensive biobank comprising tumour tissue, blood and serial plasma samples with associated clinical data which will be an invaluable resource for high-quality translational research in ovarian cancer. For detailed information on the collection of samples in TRICON8B please refer to the TRICON8B Translational Research Manual.

During site activation a Translational Research Level of Participation Form will be completed by site and sent to the MRC CTU indicating the level of participation in translational research.

For sites that opened prior to the commencement of TRICON8B a site level form will be requested at the launch of TRICON8B.

Specimens collected as part of TRICON8B will be held at the University of Cambridge. Access to tissue samples for future translational research projects will be via application to the ICON8 Trial Management Group and require approval of the Trial Steering Committee. A Translational Research sub-group will be established to assess applications (and will include TRICON8B investigators, members of the ICON8 TMG, MRC CTU senior scientific staff and independent experts).

APPENDIX 12.1 LEVELS OF PARTICIPATION IN TRANSLATIONAL RESEARCH

TRICON8B will be conducted at 3 levels as it is recognised that a variety of clinical centres with access to different levels of research support will enter patients into the trial. All centres are encouraged to participate if local R&D and logistical support allows. The majority of centres are expected to participate at levels 1 & 2. Selected centres will be invited to participate at level 3. Further detailed information on all levels of participation is given in the TRICON8B Manual.

APPENDIX 12.1.1 LEVEL 1 (TUMOUR SPECIMENS)

All clinical centres are expected to participate in the translational research programme at this level. It is appropriate for centres with no additional facilities/support for sample collection/preparation. Formalin-fixed paraffin-embedded tissue taken at the time of IPS will be collected; and for DPS patients paired samples from the original biopsy specimen and at DPS will be collected.

APPENDIX 12.1.2 LEVEL 2 (TUMOUR SPECIMENS AND WHOLE BLOOD SAMPLE)

All clinical centres are encouraged to participate in the translational research programme at this level. It is appropriate for centres with no additional facilities/support for sample collection/preparation. Tissue specimens and peripheral blood samples for DNA will be collected.

APPENDIX 12.1.3 LEVEL 3 (TUMOUR SPECIMENS, WHOLE BLOOD SAMPLE AND SERIAL PLASMA SAMPLES)

This level is appropriate for clinical centres with access to a centrifuge and -70/80oC freezer facilities, and the expertise to process blood samples at site to form plasma & buffy coat layer aliquots. In addition to level 2 sampling, up to 26 serial plasma samples are required for potential immunoassay, proteomic and genomic analysis.

APPENDIX 12.2 PATIENT CONSENT FOR TRANSLATIONAL RESEARCH

In order to collect the samples for TR, patients will be required to sign a supplementary consent form for biological studies. Although most patients are expected to consent to participate, the wishes of patients who do not want to be involved in the additional TR sample collection will be respected and they will be able to enter the ICON8B clinical trial protocol only.

As the patient's participation in the sample collection is entirely voluntary, they may choose to withdraw consent for collection of further samples or use of samples already collected at any time. Although the patient is not required to give a reason for withdrawing consent, a reasonable effort should be made to establish this reason while fully respecting the patient's rights. If consent for use of already collected samples is withdrawn, the MRC CTU should be informed in writing.

If consent is withdrawn, specimens held at the trial biobank will be returned to the referring centre. However, specimens that have already undergone processing, for example in the formation of tissue microarrays, will be retained.

APPENDIX 12.3 SAMPLE COLLECTION, STORAGE AND PROCESSING

Detailed description of the samples required and instructions for the processing, labelling, handling, storage and shipment of specimens for translational research are provided in the TRICON8B manual.

A brief overview of specimen requirements is given here.

APPENDIX 12.3.1 TUMOUR SPECIMENS

Formalin-fixed paraffin-embedded surgical/biopsy specimens of the primary tumour, metastatic sites and an area of normal tissue will be collected for formation of tissue microarrays and cores taken for nucleic acid extraction. All tumour blocks will be returned to the referring centre once required samples have been taken.

APPENDIX 12.3.2 PERIPHERAL BLOOD FOR DNA ANALYSIS (PHARMACOGENOMICS AND SINGLE NUCLEOTIDE POLYMORPHISMS)

Two peripheral blood samples will be collected at baseline. Samples should be sent directly to the biobank after collection. DNA aliquots will be prepared centrally from these samples when required.

APPENDIX 12.3.3 PLASMA SAMPLING FOR ADDITIONAL TRANSLATIONAL RESEARCH STUDIES

In level 3 plasma and buffy coat samples will be collected at serial timepoints to facilitate assessment of circulating biomarkers for treatment response and resistance, and circulating DNA. Up to 26 samples will be collected in total at the following timepoints:

- Baseline (following consent, prior to cycle 1 day 1 treatment administration)
- At each chemotherapy cycle 2-6 (up to 72 hours prior to day 1 of the cycle)

- At the end of chemotherapy visit (should coincide with cycle 7 for arm B1 and B3 patients)
- **For arm B1 and B3 patients:** at cycles 7, 8, 9, 11, 13, 15 and 17 of bevacizumab maintenance
- **For arm B1 and B3 patients:** at the end of bevacizumab visit
- **For arm B1 and B3 patients:** at every 6-weekly follow up visit until week 66
- **For arm B2 patients:** at every 6-weekly follow up visit until week 66
- Week 66
- Week 78
- Week 90
- Week 102
- Month 30
- Month 36
- Month 42
- Month 48
- Month 54
- Month 60
- Month 66 or at Progression (whichever occurs first)

Samples will be stored locally at -80°C until requested for transfer to the biobank.

APPENDIX 13 CHEMOTHERAPY RESPONSE SCORE

The chemotherapy response scoring system in **Table 32** is being incorporated into the new International Collaboration on Cancer Reporting Guidelines. This assessment is currently recommended but not mandated. At trial sites where this assessment has been incorporated into routine pathology reporting practice please ensure that this information is recorded on the Surgery CRF (for DPS patients only).

Table 32

Score	Criterion	Tumour Regression Grading
1	Mainly viable tumour with minimal regression-associated fibro-inflammatory changes* limited to a few foci.	No or minimal tumour response
2	Multifocal or diffuse regression-associated fibro-inflammatory changes, with viable tumour ranging from diffuse sheets, streaks or nodules, to extensive regression with multifocal but easily identifiable residual tumour.	Partial tumour response
3	Mainly regression, with few irregularly scattered individual tumour cells or cell groups (all measuring less than 2 mm), or no residual tumour identified.	Complete or near-complete response

* Regression-associated fibro-inflammatory changes: fibrosis associated with macrophages, including foam cells, mixed inflammatory cells and psammoma bodies; to be distinguished from tumour-related inflammation or desmoplasia.

APPENDIX 14 MULTI-OMICS IMAGING ANALYSIS

The ICON8 Trial Management Group (TMG) have identified a need for a research imaging proposal within the ICON8 Trial with the aims of improving patient stratification to determine who most benefit from debulking surgery in ovarian cancer, and to develop non-invasive methods to predict patients who may have less benefit from current therapy by correlation of imaging features with somatic genomic markers. Previous studies have shown some evidence that volumetric analysis of computed tomography (CT) images may have improved utility for assessment of response to chemotherapy and patient outcomes in ovarian cancer compared to standard RECIST evaluation. Computational texture

analysis of standard CT images has also been shown in a small retrospective study to correlate with patient outcomes and tumour heterogeneity in advanced hgih grade ovarian carcinoma.

A feasibility assessment will be carried out at 3 UK ICON8 centres (The Christie, Addenbrookes and the Beatson) to evaluate whether;

1. We can collect the required images from the sites: these would be CT scans that were conducted during the ICON8 study.
2. We can conduct volumetric and computational texture analysis on these images and correlate with clinical and translational data.
3. We can collaborate with CRUK's current imaging research programme.
4. We can advance the sub-study from a pilot to a full grant proposal.
